# Targeted Mass Spectrometry Enables Quantification of Novel Pharmacodynamic Biomarkers of ATM Kinase Inhibition

**DOI:** 10.3390/cancers13153843

**Published:** 2021-07-30

**Authors:** Jeffrey R. Whiteaker, Tao Wang, Lei Zhao, Regine M. Schoenherr, Jacob J. Kennedy, Ulianna Voytovich, Richard G. Ivey, Dongqing Huang, Chenwei Lin, Simona Colantonio, Tessa W. Caceres, Rhonda R. Roberts, Joseph G. Knotts, Jan A. Kaczmarczyk, Josip Blonder, Joshua J. Reading, Christopher W. Richardson, Stephen M. Hewitt, Sandra S. Garcia-Buntley, William Bocik, Tara Hiltke, Henry Rodriguez, Elizabeth A. Harrington, J. Carl Barrett, Benedetta Lombardi, Paola Marco-Casanova, Andrew J. Pierce, Amanda G. Paulovich

**Affiliations:** 1 Fred Hutchinson Cancer Research Center, Clinical Research Division, Seattle, WA 98109, USA; jwhiteak@fredhutch.org (J.R.W.); twang234@fredhutch.org (T.W.); leizhao@fredhutch.org (L.Z.); rschoenh@fredhutch.org (R.M.S.); jkennedy@fredhutch.org (J.J.K.); uvoytovi@fredhutch.org (U.V.); rivey@fredhutch.org (R.G.I.); dhuang@fredhutch.org (D.H.); clin@fredhutch.org (C.L.); 2 Cancer Research Technology Program, Antibody Characterization Lab, Frederick National Laboratory for Cancer Research, Frederick, MD 21701, USA; colantos@mail.nih.gov (S.C.); tessa.caceres@nih.gov (T.W.C.); rhonda.roberts@nih.gov (R.R.R.); joseph.knotts@nih.gov (J.G.K.); kaczmarczykj@mail.nih.gov (J.A.K.); josip.blonder2@nih.gov (J.B.); joshua.reading@nih.gov (J.J.R.); christopher.richardson2@astrazeneca.com (C.W.R.); stephanie.garcia-buntley@nih.gov (S.S.G.-B.); william.bocik@nih.gov (W.B.); 3 Experimental Pathology Laboratory, Laboratory of Pathology, Center for Cancer Research, National Cancer Institute, National Institute of Health, Bethesda, MD 20892, USA; hewitts@mail.nih.gov; 4 Office of Cancer Clinical Proteomics Research, National Cancer Institute, Bethesda, MD 20892, USA; hiltket@mail.nih.gov (T.H.); rodriguezh@mail.nih.gov (H.R.); 5 Translational Sciences, Oncology, AstraZeneca, Cambridge CB4 0WG, UK; liz.harrington@astrazeneca.com (E.A.H.); carl.barrett@astrazeneca.com (J.C.B.); benedetta.lombardi@astrazeneca.com (B.L.); paola.marco-casanova@astrazeneca.com (P.M.-C.); apierce@crescendobiologics.com (A.J.P.)

**Keywords:** multiple reaction monitoring, DNA damage response, immuno-MRM, targeted proteomics

## Abstract

**Simple Summary:**

Functionality of the cellular DNA damage response (DDR) network affects risk for developing cancer, and the DDR is also a target of cancer therapies. Thus, it is important that we have reliable laboratory methods for determining the activity of this network. We describe the development and analytical validation of a targeted mass spectrometry-based 51-plex assay (DDR-2) for measuring proteins and post-translational modifications related to the DDR. The findings demonstrate identification of potential novel pharmacodynamic biomarkers.

**Abstract:**

The ATM serine/threonine kinase (HGNC: ATM) is involved in initiation of repair of DNA double-stranded breaks, and ATM inhibitors are currently being tested as anti-cancer agents in clinical trials, where pharmacodynamic (PD) assays are crucial to help guide dose and scheduling and support mechanism of action studies. To identify and quantify PD biomarkers of ATM inhibition, we developed and analytically validated a 51-plex assay (DDR-2) quantifying protein expression and DNA damage-responsive phosphorylation. The median lower limit of quantification was 1.28 fmol, the linear range was over 3 orders of magnitude, the median inter-assay variability was 11% CV, and 86% of peptides were stable for storage prior to analysis. Use of the assay was demonstrated to quantify signaling following ionizing radiation-induced DNA damage in both immortalized lymphoblast cell lines and primary human peripheral blood mononuclear cells, identifying PD biomarkers for ATM inhibition to support preclinical and clinical studies.

## 1. Introduction

The DNA damage response (DDR) is a highly regulated signal transduction network that orchestrates the temporal and spatial organization of protein complexes required to detect and repair (or tolerate) DNA damage (e.g., nucleotide excision repair, base excision repair, homologous recombination, non-homologous end joining, post-replication repair) [1]. The DDR is of critical significance in oncology, as genetic instability caused by dysregulation of the DDR is a key feature of cancer cells [2], and chemotherapy and radiation therapy induce DNA damage to promote cell death. Thus, components of the DDR network are potential therapeutic targets, and being able to quantify and understand activity in the DDR network has clinical implications for cancer patients and those at risk [3]. 

The Ataxia-telangiectasia-mutated (ATM) gene encodes a serine/threonine protein kinase that initiates the DDR to DNA double-stranded breaks (DSB). ATM is activated through dissociation of the ATM homodimer to active monomers via autophosphorylation at several sites [4], including Ser1981 [5], Ser367 [6], and Ser2996 [7]. Activated ATM phosphorylates downstream components to mediate DNA repair and cell cycle regulation. Cells lacking ATM, as seen in patients with ataxia telangiectasia, are sensitive to DNA damage [8]. Three ATM inhibitors developed by KuDOS (Cambridge, UK) and AstraZeneca (Cambridge, UK), KU-55933, AZD0156, AD1390, display potent and exquisite inhibition of ATM with no meaningful capacity to inhibit other phosphatidylinositol 3-kinase-related kinases (PIKKs), such as ATR and DNA-PK [9,10,11]. AZD0156 is a particularly potent inhibitor of ATM and is in clinical trials (NCT02588105) in combination with DNA damage-inducing agents, where it is expected to enhance cell death in tumor cells by reducing their capacity to resolve DNA damage. 

Quantifying proteins and protein networks for pharmacokinetic (PK), pharmacodynamic (PD), and proof of mechanism (POM) studies is critical for translating novel therapies, such as ATM inhibitors. Indeed, AstraZeneca recently revised its drug development framework [12,13] with a view to increasing success rates of drugs in pivotal Phase 3 studies. Key components are POM studies and determining PK/PD relationships in early phase clinical trials. Conventional immunoassay platforms (e.g., ELISA, immunohistochemistry) are generally used for these studies; however, these platforms are critically dependent on the absolute specificity of individual antibodies, and establishing this specificity is costly in terms of time and resource. As a result, only a handful of fully validated assays can be developed for each drug project, where the choice of which assays to develop is largely done on the basis of “best educated guess” arising from orthogonal preclinical methods such as Western blotting. Additionally, in many cases an assay for a given target cannot be quickly developed because there are no fit-for-purpose antibodies available. In such cases a bespoke reagent antibody generation campaign may be initiated, adding to the time and cost of drug development. Adding to the issue, proteins act as interconnected “networks”, and the effects of cancer driver mutations spread throughout the network; thus, ideally we would have assays to quantify panels of multiple proteins in early phase clinical trials to assess the activity of pathways/networks that determine treatment responses [14], and this developmental effort is not practical using conventional platforms. Thus, new tools are needed for quantifying proteins and post-translational modifications to aid discovery of new pharmacodynamic markers, enhance mechanistic studies, and translate research findings to clinical implementation.

To address this need, we applied targeted multiple reaction monitoring (MRM) mass spectrometry (MS) to develop quantitative assays to proteins and post-translational modifications [15,16]. MRM is a targeted mode of MS in which the full analytical capacity of the instrument is focused on a set of specific analytes, maintaining a high degree of specificity and enhancing the sensitivity of detection [17,18]. By using spiked-in isotopically labeled internal standards, precise relative quantification is achieved, and results can be harmonized across laboratories [19,20]. To analyze low abundance proteins and post-translation modifications, including phosphorylation, we coupled an antibody enrichment step to MRM [21]. These “immuno-MRM” assays enable precise quantification of low abundance proteins [22], high multiplexing capability while maintaining specificity for individual analytes [23,24], and standardization/harmonization across laboratories [25]. 

We recently established the feasibility of configuring highly multiplex, MRM-based proteomic assays to quantify DDR network signaling [7,26,27,28] and applied the “DDR-1” MRM assay to identify a novel pharmacodynamic biomarker for ATM inhibition [29] to support early phase clinical development of AZD0156, a potent, selective inhibitor of the ATM kinase. In the current study, we extend our prior work with a novel multiplex “DDR-2” immuno-MRM assay that substantially expands our coverage of DDR network signaling. Specifically, we describe development, bioanalytical validation, and application of a novel immuno-MRM assay targeting 51 peptides (including 33 phosphopeptides) representing 31 proteins associated with the DDR. Targets were identified through a combination of literature search and empirical phosphoprofiling of primary peripheral blood mononuclear cells (PBMC) and immortalized lymphoblast cell lines (LCL) cells exposed to DNA damage. The DDR-2 assay was analytically characterized according to best practices to determine working figures of merit (e.g., linear range, repeatability, selectivity, and stability). Proof-of-principle demonstration of the assay for measuring cellular phosphosignaling dynamics was conducted using lymphoblast cell lines exposed to ionizing radiation. Furthermore, to demonstrate the utility of the assay for pharmacodynamic profiling and discovery of pharmacodynamic biomarkers, we profiled the DDR in primary human PBMCs exposed to ionizing radiation in the presence of DDR kinase inhibitors.

## 2. Materials and Methods

### 2.1. Materials and Reagents

Urea (#U0631), Trizma base (#T2694), citric acid (#C0706), dimethyl sulfoxide (DMSO, #D2438), and iodoacetamide (IAM, #A3221) were obtained from Sigma (St. Louis, MO, USA). Acetonitrile (MeCN, #A955), water (#W6, LCMS Optima^®^ grade), trifluoroacetic acid (TFA, LC-MS grade, #85183), tris(2-carboxyethyl)phosphine (TCEP, #77720), phosphate buffered saline (PBS, #BP-399-20), and (3-[(3-cholamidopropyl) dimethylammonio]-1- propanesulfonate) (CHAPS, #28300) detergent were obtained from Thermo Fisher Scientific (Waltham, MA, USA). Formic acid (#1.11670.1000) was obtained from EMD Millipore (Billerica, MA, USA). Lys-C (Wako, #129-02541) and sequencing grade trypsin (#V5111, Promega, Madison, WI, USA) were used for digestion of samples. The ATM kinase inhibitor KU-55933 was from Selleckchem (#S1092, Houston, TX, USA). The ATM kinase inhibitor AZD0156, DNAPK kinase inhibitor AZD7648, and the ATR kinase inhibitor AZD6738 were supplied by AstraZeneca and dissolved at 10 mM in DMSO. Rabbit monoclonal antibodies were produced with Excel Biopharm (Burlingame, CA, USA). Light (unlabeled) synthetic peptides were obtained from New England Peptide (NEP, Gardner, MA, USA) as crude (flash purified) grade. Stable isotope-labeled (heavy) peptides from NEP corresponding to the tryptic analyte sequence were purified >95% by HPLC, labeled with [^13^C and ^15^N] at the C-terminal Arg or Lys, and quantified by amino acid analysis (AAA). Aliquots were stored in 3% acetonitrile/0.1% formic acid at −80 °C until use.

### 2.2. Cells and Culture Conditions

Cell lines were obtained from the following sources: human lymphoblast cell lines GM07057 (*ATM+/+*) and GM01526 (*ATM−/−*) were obtained from the Coriell Institute (Camden, NJ, USA). The HeLa cell line was obtained from American Type Culture Collection (ATCC #CCL-2, Manassas, VA, USA), and empty vector-transfected MCF10A-EV cells were a gift from James A Wells (Department of Pharmaceutical Chemistry, University of California, San Francisco, CA, USA). 

LCL were grown in RPMI1640 (Gibco #11875-093, Waltham, MA, USA) plus 15% heat-inactivated FBS (HyClone #SH30071.03, Logan, UT, USA) and 1% Penicillin-Streptomycin (Gibco #15140-122). HeLa were cultured in Eagle’s Minimum Essential Medium (Invitrogen, #10370, Waltham, MA, USA) supplemented with 10% heat-inactivated FmBS (Hyclone, #SH30071.03HI), 1 mM sodium pyruvate (Invitrogen, #11360), 2 mM L-glutamine (Invitrogen, #25030), and 100 units/mL of penicillin-streptomycin (Invitrogen, #15140). MCF10A-EV cells were cultured in DMEM/F-12 (Thermo Fisher Scientific #11320033) supplemented with 5% Horse Serum (Thermo Fisher Scientific #16050130), 20 ng/mL Epidermal Growth Factor (PeptroTech #AF-100-15, Cranbury, NJ, USA), 10 µg/mL Insulin (Millipore Sigma #I6634, Burlington, MA, USA), 0.5 µg/mL Hydrocortisone (Millipore Sigma #H0888) and 100 ng/mL Cholera Toxin (Millipore Sigma #C8052) at 37 °C in a humidified 5% carbon dioxide incubator.

LCL were collected by centrifugation and diluted to 1 million cells/mL in fresh growth medium for 36 h prior to treatment with ionizing radiation (IR). Irradiation was performed in a JL Shepherd Mark I irradiator using a ^137^Cs source delivering a dose rate of 4.7 Gy/min; mock (sham)-irradiated cells were handled in precisely the same manner as the irradiated cells, but the irradiator was not turned on.

For inhibitor studies, LCL were treated for 1 h prior to DNA damage by the addition of 1 μL of 10 mM KU-55933 per mL of culture medium (final concentration 10 μM). For controls, mock-treated cells received 1 μL DMSO (vehicle) per mL of culture medium.

### 2.3. Human Samples

Leukocyte cones were supplied by NHS Blood and Transplant Service (NHSBT, Cambridge, UK) as anonymized samples from consenting donors and were commercially acquired from NHS-BT Cambridge. Human PBMC were isolated from leukocyte cones by density gradient centrifugation (Lymphoprep, STEMCELL technologies #07801, Vancouver, BC, Canada). To prepare PBMCs, leukocyte cones resulting from plateletpheresis were diluted with four volumes of PBS, layered over 0.75 volumes of Lymphoprep at room temperature, and centrifuged at 800× *g* for 30 min with no brake. PBMCs were harvested from the Lymphoprep-plasma interface and re-suspended in RPMI medium (SIGMA R8758-500ML) supplemented with 10% heat-inactivated FBS (Hyclone #SH30071.03HI), 100 units/mL of penicillin, 100 units/mL streptomycin (Gibco #15070-063), 2 mM L-Glutamine (Gibco #25030-081). The diluted PBMC preparation was spun down and treated with Red Blood Cell Lysis Buffer (Gibco #A10492-01) for 3 min, washed and re-suspended in supplemented RPMI medium. The T-cell population was activated and expanded using SEB (SIGMA, #S4881) at a final 100 ng/mL concentration. SEB+ and SEB− treated PBMCs were cultured in supplemented RPMI medium at 1.5 and 3 × 10^6^ cells/mL, respectively. Cells were cultured for 4 days at 37 °C and 5% CO_2_ in T175 flasks with fresh growth medium in presence of DMSO, 30 nM AZD0156, 3 μM AZD6738 or 1 μM AZD7648. On day 4, cells were subjected to ionizing radiation (5 Gy cumulative dose, Faxitron X-ray instrument) or mock-irradiated and incubated at 37 °C, 5% CO_2_ for 1 h prior to harvesting.

### 2.4. Cell Lysate Generation

LCL and PBMCs were lysed at 5 × 10^7^ cells/mL in freshly prepared ice-cold urea lysis buffer (6M Urea, 25 mM Tris pH 8.0, 1 mM EDTA, 1 mM EGTA containing protease and phosphatase inhibitors (Sigma, #P0044, #P5726, and #P8340)). Lysates were transferred to cryo-vials, stored in liquid nitrogen, and thawed on ice. Protein concentrations of lysates were measured in triplicate using Micro BCA Protein Assay Kit (Thermo Fisher Scientific #23235). HeLa and MCF10A-EV cells were trypsizined, centrifuged at 1500 rpm for 6 min and the supernatant was removed and discarded. Cells were washed with 1× PBS, centrifuged as before, resuspended in 1× PBS and counted. Cells were split according to desired cell number in 15 mL centrifuge tubes and centrifuged as before. Centrifuge tubes with pellets were stored at −80 °C until lysis. Cell pellets were lysed using RIPA lysis and extraction buffer (Thermo Fisher Scientific #89900) following the manufacturer’s protocol. Mammalian Protease Inhibitor (VWR #97063-010) was added according to instructions.

### 2.5. Phosphoproteomics Discovery Using SILAC and Immobilized Metal Affinity Chromatography

For SILAC labeling, LCL were cultured as described above in RPMI 1640 SILAC basal medium (Thermo Fisher Scientific #89984) supplemented with 15% heat-inactivated (30 min at 56 °C) dialyzed FBS (Thermo Fisher Scientific #88440), 1% Penicillin/Streptomycin (Gibco #15140-122), 0.1 mg/mL L-Arginine (13C6, 15N4), and 0.1 mg/mL L-Lysine (^13^C_6_, ^15^N_2_) (Cambridge Isotope Laboratories, #CNLM-539-H-0.1 and CNLM-291-H-0.1, Tewksbury, MA, USA). For “Forward” SILAC experiments, labels were Light (ATM−/−, mock), Medium (ATM+/+, mock), and Heavy (ATM+/+, IR). For “Reverse” SILAC experiments, labels were Light (ATM+/+, IR), Medium (ATM−/−, mock), and Heavy (ATM−/−, IR). Cells were suspended at 10^6^ cells/mL in fresh medium, and 20 mL of each cell line was transferred to T25 flasks and allowed to equilibrate at 37 °C, 5% CO_2_ for 40 h. Cells were treated with ionizing radiation and harvested, and protein lysates were generated as described above.

Lysates were reduced, alkylated with iodoacetamide, and digested by the addition of Lys-C at a 1:100 trypsin:protein ratio (by mass). After 2 h, an aliquot of trypsin was added at a 1:50 trypsin:protein ratio and incubated overnight at 37 °C with shaking. After 16 h, the reaction was quenched with formic acid (final concentration 1% by volume). The desalted tryptic digest was fractionated by high-pH reverse phase (RP) liquid chromatography using 4 mg of the protein digest loaded onto a LC system consisting of an Agilent 1200 HPLC (Agilent, Santa Clara, CA, USA) with mobile phases of 5 mM NH_4_HCO_3_, pH 10 (A) and 5 mM NH_4_HCO_3_ in 90% MeCN, pH 10 (B). The peptides were separated by a 4.6 mm × 250 mm Zorbax Extend-C18, 3.5 μm, column (Agilent #770953-902) over 96 min at a flow rate of 1.0 mL/min by the following timetable: hold 0% B for 9 min, gradient from 0 to 10% B for 4 min, 10 to 28.5% B for 50 min, 28.5 to 34% B for 5.5 min, 34 to 60% B for 13 min, hold at 60% B for 8.5 min, 60 to 0% B for 1 min, re-equilibrate at 0% B for 5 min. Then 1-min fractions were collected from 0–96 min by the shortest path by row in a 1 mL deep well plate (Thermo Fisher Scientific #95040450). The high pH RP fractions were concatenated into 24 samples by every other plate column starting at minute 15 (e.g., sample 1 contained fractions from wells B10, D10, F10, etc.). The remaining fractions were combined such that fractions from 12 to 14 min were added to sample 1, all fractions after 86 min were added to sample 24, and all fractions from 0 to 11 min were combined into sample “A”. 95% of every 12th fraction of the 24 samples was combined (1,13; 2,14; …) to generate 12 samples, which were dried down and stored at −80 °C prior to phosphopeptide enrichment. 

Immobilized metal affinity chromatography (IMAC) enrichment was performed using Ni-NTA-agarose beads (Qiagen, Valencia, CA, USA, #36113) stripped with EDTA and incubated in a 10 mM FeCl_3_ solution to prepare Fe^3+^-NTA-agarose beads. Peptide enrichment was performed using 500 μg of fractionated lysate digest reconstituted in 400 μL of 0.1% TFA in 80% MeCN and incubated for 30 min with 75 μL of the 5% bead suspension, mixing at 1400 rpm at room temperature. After incubation, the beads were washed 3 times each with 150 μL of 0.1% TFA in 80% MeCN and then once with 150 μL of 0.1% TFA. Phosphorylated peptides were eluted from the beads twice using 150 μL of 500 mM potassium phosphate, pH 7, after incubating for 3 min. Samples were desalted using StageTips (Thermo Fisher Scientific #SP301) loaded with reverse phase material [30], dried down, and re-suspended in 0.1% FA, 3% MeCN. The samples were frozen at −80 °C until analysis. 

### 2.6. Liquid Chromatography—Tandem Mass Spectrometry for Shotgun Analysis

Phosphopeptide-enriched samples were analyzed by LC-MS/MS on a nanoAcquity HPLC (Waters, Milford, MA, USA) with mobile phases of 0.1% FA in water (A) and 0.1% FA in MeCN (B). Each IMAC-enriched fraction was directly injected and separated by a 75 µm × 250 mm C18, 130 Å, 1.7 µm, column (Waters #186003545) by the following method: gradient from 3 to 40% B for 120 min, gradient from 40 to 90% B for 2 min, hold 90% B for 10 min, re-equilibrate at 3% B for 23 min. The flow rate was 300 nL/min. The HPLC was coupled to an LTQ-Orbitrap Velos (Thermo Fisher Scientific) hybrid mass spectrometer using an Advance CaptiveSpray source (Michrom Bioresources, Auburn, CA, USA) operated in positive ion mode. A spray voltage of 1700 V was applied to the nanospray tip. MS/MS analysis consisted of 1 full scan MS from 300–2000 *m*/*z* at resolution 30,000 followed by 15 data dependent MS/MS scans. Dynamic exclusion parameters included repeat count 1, exclusion list size 500, and exclusion duration 15 s.

### 2.7. Tandem Mass Spectrometry Data Analysis

Raw MS/MS spectra from the analysis of the basic reverse phase/IMAC lysate digest were searched using MaxQuant/Andromeda [31] against version 3.68 of the human International Protein Index (IPI) with tryptic enzyme constraint set for up to two missed cleavages, oxidized methionine and phosphorylated serine, threonine and tyrosine set as a variable modification, and carbamidomethylated cysteine set as a static modification. Peptide MH+ mass tolerances were set at 20 ppm. The overall FDR was set at ≤1% based on a decoy database search. Any localization with a probability greater than 0.8 was reported as being localized; below that was reported as an ambiguous localization. Quantification of Heavy:Medium:Light ratios was performed using MaxQuant. To quantify peptides with the most reliable signals, a functional limit of quantification was defined as the intensity where the median CV was equal to 20% (calculated by plotting the median CV of the peak area ratios as a function of the minimum peak area (minimum of the heavy and light peaks)). The following requirements were applied to identify reproducibly responsive phosphopeptides: (i) identified in both the Forward and Reverse experiments, (ii) intensity above the functional limit of quantification (defined as intensity with median CV = 20%), (iii) relative ratio (±DNA damage) of ≥1.4 or ≤0.7 fold (log2 ratio +/− 0.5).

### 2.8. Targeted MRM Proteomics Sample Preparation

LCL and PBMC samples were processed in a blinded fashion. Biological replicates of LCL were conducted by independent cultures processed on separate days. Lysates were reduced, alkylated with iodoacetamide, and digested by the addition of Lys-C at a 1:100 trypsin:protein ratio (by mass). After 2 h, an aliquot of trypsin was added at a 1:50 trypsin:protein ratio and incubated overnight at 37 °C with shaking. After 16 h, the reaction was quenched with formic acid (final concentration 1% by volume). A mix of stable isotope-labeled peptide standards was added to the digest at 200 fmol/mg. The mixture was desalted using Oasis HLB 96-well plates (Waters #WAT058951) and a positive pressure manifold (Waters #186005521). The eluates were aliquoted by volume, lyophilized, and stored at −80 °C.

Enrichment was performed as previously described [7,32], with the following modifications. The final assay consisted of a mixture of 55 antibodies. Antibodies were crosslinked on protein G beads (GE Sepharose, #28-9513-79), and peptide enrichment was performed using 1 μg antibody—protein G magnetic beads for each target. Unless indicated, 500 μg of trypsin-digested lysate resuspended in 200 μL PBS + 0.03% CHAPS (pH was adjusted to 8.0 with 5 μL of 1 M Tris) was inputted to each enrichment. Beads were mixed in the incubation plate, washed twice in PBS buffer + 0.03% CHAPS, washed once in 1/10 × PBS, and peptides were eluted in 26 μL of 5% acetic acid/3% acetonitrile/50 mM citrate. The elution plate was covered with adhesive foil and frozen at −80 °C until analysis.

### 2.9. Liquid Chromatography Multiple Reaction Monitoring (MRM) Mass Spectrometry

LC-MS was performed with an Eksigent 425 nanoLC system with a nano autosampler and chipFLEX system (Eksigent Technologies, Dublin, CA, USA) coupled to a 5500 QTRAP mass spectrometer (SCIEX, Foster City, CA, USA). Peptides were loaded on a trap chip column (Reprosil C18-AQ, 0.5 mm × 200 μm, SCIEX, #804-00016) at 5 μL/min for 3 min using mobile phase A (0.1% formic acid in water). The LC gradient was delivered at 300 nL/min and consisted of a linear gradient of mobile phase B (90% acetonitrile and 0.1% formic acid in water) developed from 3–14% B in 1 min, 14–34% B in 20 min, 34–90% B in 2 min, and re-equilibration at 3% B on a 15 cm × 75 μm chip column (ChromXP 3C18-CL particles, 3 μm, SCIEX, #804-00001). The nano electrospray interface was operated in the positive ion MRM mode. Parameters for declustering potential (DP) and collision energy (CE) were taken from optimized values in Skyline [33]. Scheduled MRM transitions used a retention time window of 150 s and a desired cycle time of 1.5 s, enabling sufficient points across a peak for quantitation. A minimum four transitions per peptide pair, including endogenous and spiked heavy peptides, were recorded for each light and heavy peptide. 

MRM data acquired on the 5500 QTRAP were analyzed by Skyline [33]. Peak integrations were reviewed manually, and transitions from analyte peptides were confirmed by the same retention times of the light synthetic peptides and heavy stable isotope-labeled peptides, and with equivalent relative areas of recorded transitions. Transitions with detected interferences were not used in the data analysis. Integrated raw peak areas were exported from Skyline [33] and total intensity was calculated using Peak Area + Background. Transitions were summed for each light/heavy pair and peak area ratios were obtained by dividing peak areas of light peptides by that of the corresponding heavy peptides. Peak area ratios were log (base 2) transformed for statistical analysis. 

### 2.10. Fit-for-Purpose Assay Validation

Four experiments (described below) were performed to characterize the analytical performance of the assays: (i) response curves, (ii) repeatability, (iii) stability, (iv) sequential enrichment. 

#### 2.10.1. Response Curve

Response curves were generated in a background matrix consisting of an equal mixture of protein lysate from LCL GM07057 + 10 Gy IR (1 h) and LCL GM07057 + mock IR (1 h). The pooled lysate was digested by trypsin, and the heavy stable isotope-labeled peptides were added to aliquots by serial dilution covering the concentrations 2000, 200, 50, 12.5, 4.25, 1.4, 0.7, 0.35 fmol/mg. Light peptide was also spiked into the cell lysate pool at 200 fmol/mg. Blanks were prepared using background matrix with light peptide (no heavy spike). All points were analyzed by immuno-capture and mass spectrometry in four replicates. Curves were analyzed using Skyline. Linear regression was performed using log transformed data on all points above the lower limit of quantification. The Lower Limit of Quantifications (LLOQs) was obtained by empirically finding the lowest point on the curve that had CV < 20% in the curve replicates. All measurements were filtered by the LLOQ (i.e., all measurements were required to be above the LLOQ). The upper limit of quantification (ULOQ) was determined by the highest concentration point of the response curve that was maintained in the linear range of the response. For curves that maintained linearity at the highest concentration measured, the ULOQ is a minimum estimate.

#### 2.10.2. Repeatability

Repeatability was determined using the same pooled lysate matrix used to generate the response curves. Heavy peptides were spiked in at three concentrations (0.8, 80, 800 fmol/mg). All light peptides were added at 200 fmol/mg. Complete process triplicates (including digestion, capture, and mass spectrometry) were prepared and analyzed on five independent days. Intra-assay variation was calculated as the mean CV obtained within each day. Inter-assay variation was the CV calculated from the mean values of the five days. 

#### 2.10.3. Peptide Stability

Stability of the enriched peptides was determined by analyzing aliquots of the medium spike level sample used in repeatability studies after storage at 4 °C in the autosampler for approximately 24 h and after 2 freeze-thaw cycles. 

### 2.11. Sequential Enrichment of Multiple Assay Panels

Sequential enrichment refers to using the flow-through from immunoaffinity enrichment as the input for a second enrichment targeting different analytes in the same sample [23]. Thus, sequential enrichment allows an increase in the capacity to analyze more targets from an aliquot of a sample. Sequential enrichment of the multiplexed assay was evaluated by using the pooled LCL background lysate from above. Separate aliquots were prepared using 500 μg of the protein lysate. Heavy peptides were added to the aliquots at 200 fmol/mg. The multiplexed assay was used to analyze the target peptides from the lysate and compared to results using the flow-through of previously enriched samples (separate aliquots of the same lysate first enriched using the DDR-1 panel [7]). All captures were performed as described above; flow-through samples were immediately processed using antibodies coupled to magnetic beads (i.e., no freeze-thaw occurred between captures).

### 2.12. Further Characterization of Novel Monoclonal Antibody Reagents Generated in This Study

#### 2.12.1. Immunoblot

Recombinant proteins were obtained from Origene (Rockville, MD, USA) or Novus Biologicals (Littleton, CO, USA), see Supplemental Table S2 for catalog numbers. Western immunoassays were performed using traditional immunoblotting techniques according to the designed protocol described in the antibodies.cancer.gov (accessed on 23 July 2021) SOP M-103 with the following modifications. Validation of proper protein sample loading was done using Bio-Rad (Hercules, CA, USA) Stain-Free precast gel confirmation. Traditional immunoblotting for recombinant protein was conducted at a concentration of 10 μg/mL in reducing conditions (200 ng total protein). Whole cell lysates were diluted to 2.5 mg/mL in reducing conditions (50 μg total protein). Transfer of protein from Bio-Rad precast stain free gels was performed by Bio-Rad Turbo-Blot at “High MW” setting for 10 min. Blocking of the membrane was performed using Bio-Rad Blotting Grade Blocker at 5% in 1× PBS/0.5% Tween-20. Primary antibodies (1 mg/mL) were diluted in 1× PBS/0.5% Tween-20 to a dilution of 1:5000 at a total volume of 25 mL. Washing of membrane was conducted using 1× PBS/0.5% Tween-20 three times. Secondary HRP-linked rabbit specific antibody (Jackson ImmunoResearch, West Grove, PA, USA) was diluted at 1:5000 in 1× PBS/0.5% Tween-20 at a volume of 25 mL. Immuno detection was performed using colorimetric substrate Opti-4-CN (Bio-Rad) at 1 mL per blot. Development of immunoblot was captured using Bio-Rad ChemiDoc MP imaging system.

#### 2.12.2. WES System

The Simple Western (Wes, ProteinSimple, San Jose, CA, USA) system was used to detect primary antibody binding to a target protein in cell lysates (MCF10A, LCL57). The Simple Westerns were performed following the procedures detailed in the antibodies.cancer.gov (accessed on 23 July 2021) SOP M-134 with the following modifications. Cell lysates were run using the 12–230 kDa separation module, 8 × 25 capillary cartridges (ProteinSimple) and detected with Anti-Rabbit Detection Module (ProteinSimple). Irradiated and phosphatase treated cell lysates were run at a concentration of 200 μg/mL and incubated with primary antibodies diluted 1:500.

#### 2.12.3. Immunofluroescence (IF) Staining

The Human Protein Atlas Cell Atlas Standard Immunostaining Protocol was followed with minor modifications. Briefly, before starting, adherent cells were cultured overnight at 10,000 cells/well in 100 µL cell culture media in a 12.5 µg/mL superfibronectin (Sigma) coated 96-well glass bottom microplate (Sigma) and suspension cells were cultured in a Shi-fix™ coated 96-well microplate (Everest Biotech, Bicester, UK) according to manufacturer’s instructions. The following day, cell culture media were removed from cells and cells were washed with 1× Phosphate Buffered Saline (PBS, Thermo Fisher Scientific), fixed with 4% paraformaldehyde (VWR)/1× PBS, permeabilized with 0.1% Triton X-100 (Sigma)/1× PBS, washed with 1× PBS and incubated with diluted primary antibodies (2 µg/mL unless otherwise stated) with 1 µg/mL mouse anti-alpha tubulin (Abcam) in blocking buffer (4% Fetal Bovine Serum (Thermo Fisher Scientific)/1× PBS)) overnight at 4 °C. The next day, primary antibodies were removed from cells and cells were washed with 1× PBS, incubated with diluted secondary antibodies (2.5 µg/mL anti-rabbit Alexa Fluor 488 (Thermo Fisher Scientific) with 2.5 µg/mL anti-mouse Alexa Fluor 555 (Thermo Fisher Scientific) in blocking buffer) for 1.5 h at room temperature (RT) in darkness. With minimum light present, secondary antibodies were removed from cells and cells were stained with 0.2 µg/mL DAPI (VWR)/1× PBS, washed with 1× PBS, and wells were filled with glycerol (Thermo Fisher Scientific)/10× PBS (Thermo Fisher Scientific). The 96 well microplate was sealed with an adhesive aluminum PCR plate seal (VWR) and stored at 4 °C until IF analysis (Invitrogen™ EVOS™ M 5000 Imaging System (Thermo Fisher Scientific)).

#### 2.12.4. Single Cell Western Blot (SCWB)

The Standard scWest Kit (ProteinSimple) was used and the kit protocol was followed. Briefly, cells were diluted to 100,000 cells/mL with 1× Suspension Buffer (SB)(ProteinSimple) and 1 mL cells was added to the chip to allow cells to settle for 15–20 min. Chip with cells was rinsed with 1× SB, ran on Milo (ProteinSimple) where cells are lysed, separated, and captured, washed with 1× Wash Buffer (WB), incubated with primary antibody for 2 h at RT, washed with 1× WB, incubated with fluorescently labeled secondary antibody (R&D System, Inc.) for 1 h at RT in darkness, with minimal light present, washed with 1× WB, rinsed with deionized water, spun dry on a Microarray Slide Spinner (Sigma Aldrich), and imaged in a microarray scanner (ProteinSimple). The image was analyzed using Scout software (ProteinSimple). 

#### 2.12.5. Immunoprecipitation Mass Spectrometry (IP-MS)

The antibodies were tested by IP-MS using recombinant proteins and lysates of selected cell lines by grouping the antibodies into two multiplexed groups with 6 to 8 mAbs per group. Both recombinant proteins (Supplemental Table S2) and monoclonal antibodies were diluted in 1× PBS/0.05% CHAPS at a concentration of 50 mg/mL. Equal volumes of antibodies and antigens (100 μL) were mixed in a 96 deep well plate with 5 μL of Pierce™ Protein G Magnetic Beads that were previously washed with 1× PBS/0.05% CHAPS. The plate was incubated overnight at 4 °C with shaking in an Eppendorf Thermomixer. Bead washes (1× PBS/0.05% CHAPS) and elution (8M Urea/50 mM Tris/100 mM DTT elution plate) from the plate were performed automatically in a Thermo Fisher Scientific KingFisher Flex Magnetic Particle Processor. Eluates were alkylated with iodoacetamide (final concentration 50 mM) for 30 min at RT in the dark and desalted using Amicon^®^ Ultra Centrifugal Filters, MW CO 3 KDa (Millipore Sigma) until nominal urea concentration was <1 M. The spin filters were transferred to a clean collection tube, and the solution in the filters was spiked with 1 mg of Trypsin/Lys-C Mix, Mass Spec Grade (Promega). Negative controls (beads and recombinant protein, without antibody) were treated exactly the same as the IP samples. Protein and trypsin mixture were incubated overnight at 37 °C in water vapor saturated incubator with shaking. Digested samples were spun, and tryptic peptides were recovered in the collection tube by spinning. Samples were desalted with a SOLAµ™ SPE HRP Plate according to vendor instructions, dried in a speed-vac device and reconstituted in 0.1% formic acid. The peptide digests were separated using a nano-flow LC system (EASY-nLC 1200, Thermo Fisher Scientific) coupled on-line to a hybrid ion trap-orbitrap mass spectrometer (Orbitrap Elite, Thermo Fisher Scientific). Samples were injected onto 20 mm long, 75 μm inner diameter, C18, 3 μm particle size trapping column (EASY-Spray, Thermo Fisher Scientific) and separated using in line, 150 mm long, 75 μm inner diameter, C18, 2 μm particle size, analytical column (EASY-Spray, Thermo Fisher Scientific). The linear gradient for separation was 5–40% mobile phase B over 62 min at 300 nl/min flow rate, where mobile phase A was 0.1% formic acid in water, and mobile phase B was 0.1% formic acid in 80% acetonitrile. Mass spectrometer was operated in a data dependent mode scanning, using the ion mass to charge range of 350–1650, monitored at the resolution level of 60,000 at *m*/*z* 400. Each MS1 scan was followed by MS2 scan, wherein the 20 most abundant precursor ions were dynamically selected for collision-induced dissociation using normalized collision energy of 35%. Proteins were identified applying the SEQUEST HT algorithm search against the non-redundant human proteome database (i.e., SwissProt release v57.15) utilizing the software Proteome Discoverer 1.4 (Thermo Fisher Scientific). The database search thresholds included: for the monoisotopic peptide precursor ions (i.e., MS1 spectra) mass tolerance was set at 10 ppm and for the fragment ions (i.e., data-dependent MS2 spectra) mass tolerance was set at 0.6 Da. Dynamic amino acid modifications were added for the detection of the following: +57.021 Da for cysteine. IP experiments were also performed with LCL-57, HeLa and MCF10A cell lysates. Cell were lysed as described above. Lysate protein concentration was estimated with a BCA assay (Pierce). Each cell lysate was incubated with 5 μg of antibody and 5 mL of Protein G beads. Pull-down, proteolytic digestion and analysis were performed as described above for the recombinant protein experiment. IP data were compared with negative controls (beads and lysate digests, without antibody).

#### 2.12.6. Immunohistochemistry Pancreatic Cancer Tissue Micro-Array

IHC was used to test the specificity of the antibodies using NCI60 protein array as well as a pancreatic cancer tissue micro array (TMA). Tissues were sectioned into 4 µm sections and adhered to positively charged glass microscope slides. Slides were deparaffinized using the Leica Autostainer model XL (Leica, Nussloch, GmbH) following the protocol detailed in the antibodies.cancer.gov (accessed on 23 July 2021) SOP M-106. A 1× Antigen Retrieval buffer (Dako, Santa Clara, CA, USA) was prepared and slides were placed into the buffer and placed in a pressure cooker at 123 °C for 30 min. When the pressure cooker was finished, the slides were removed and allowed to cool to room temperature. Slides were then loaded on to the Dako Autostainer Model LV-1 (Agilent) and rinsed with IHC wash buffer (Dako). The slides were incubated in a hydrogen peroxide block (Cell Marque, Rocklin, CA, USA) for 10 min at room temperature. The slides were then rinsed with IHC wash buffer. The slides were then incubated with a protein block (Dako) for 10 min at room temperature. Primary antibody was diluted using antibody diluent (Dako). Primary Antibody solution was added to slides and incubated for 1 h at room temperature. Slides were rinsed with IHC wash buffer. Rabbit HRP secondary antibody (Dako) was added and incubated for 30 min at room temperature. Slides were rinsed with IHC wash buffer (Dako). The DAB substrate was prepared by mixing 1mL of substrate buffer (Cell Marque) with 1 drop of DAB chromogen (Cell Marque). DAB solution was added to the slides and incubated for 10 min at room temperature. The slides were rinsed with IHC wash buffer. The slides were then removed from Autostainer and placed back on the Leica Autostainer model XL for the hematoxylin counterstain. Slides were removed from Leica Autostainer and a coverslip was applied using cytoseal XYL mounting medium (Richard Allan Scientific, San Diego, CA, USA). 

#### 2.12.7. Immunohistochemistry NCI-60 Protein Array

IHC staining protocol above was used on the NCI60 protein array using the optimal conditions selected by the pathologist. Once staining was completed slides were scanned using a Hamamatsu NanoZoomer-XR digital slide scanner (Hamamatsu, Bridgewater, NJ, USA). Image analysis was completed to determine the staining intensity of each core on the array. Staining intensity ranged from 0–3, 0 being negative or no stain and 3 being the most intense. 

### 2.13. Public Availability of Data and Antibodies

Raw data for LCL phosphoprofiling experiments have been deposited to the ProteomeXchange Consortium [34] via the PRIDE [35] partner repository with the dataset identifier PXD026103, accessed on 28 July 2021. Targeted mass spectrometry data are available in the supplemental tables and Panorama Public [36], a public repository of targeted proteomics experiments (https://panoramaweb.org/DDR2_ATMprofiling.url, accessed on 28 July 2021). Characterization data for assays can be found via the CPTAC Assay Portal (https://assays.cancer.gov, accessed on 28 July 2021), and antibodies are available through the CPTAC Antibody Portal (https://antibodies.cancer.gov, accessed on 28 July 2021) Portals (see Supplemental Table S2 for IDs).

## 3. Results

### 3.1. Identification of DNA Damage Response Phosphosites for Targeted Assay Development

We sought to develop a novel quantitative assay panel that expanded our capabilities for profiling the DDR and evaluating putative pharmacodynamic markers of ATM kinase inhibition. To identify ATM-dependent, ionizing radiation-responsive phosphosites that are quantifiable by MRM, we performed mass spectrometry (MS)-based phosphoproteomic profiling of protein lysates generated from lymphoblast cell lines GM07057 (‘LCL-57’; ATM+/+) and GM01526 (‘LCL-26’; ATM−/−) harvested 1 h following exposure (or mock-exposure) to ionizing radiation (IR) (10 GY). For relative quantification of phosphorylation, we used the SILAC (Stable Isotope Labeling by/with Amino acids in Cell culture) approach, where the cell culture was supplemented with stable isotopically labeled amino acids [37]. To eliminate any bias in experimental design and provide complete biological replicates, the labeling culture was performed in both directions by swapping the media in a separate experiment (i.e., forward and reverse labeling). LC-MS analyses enabled quantification of 6903 unique phosphopeptides (3222 in the forward and 3681 in the reverse labeled samples; see Supplemental Table S1 for a list of all peptides identified and the quantitative ratios). We found that 682 phosphosites demonstrated increased levels (>2 fold change in both forward and reverse labeled samples) in the irradiated cells, regardless of ATM expression, whereas 60 phosphosites were only IR-induced in the ATM+/+ cells. To identify the subset of IR-responsive phosphosites also detectable in primary human PBMCs, we mined our previously published phosphoprofiles of PBMCs exposed to IR [28], and also phosphopeptides identified in previous literature relevant to the DDR. IR-responsive phosphopeptide candidates for assay development were ranked using established practices [18,38] for observational, chemical, and physical properties to identify those sequences amenable to development of mass spectrometry-based assays. In total, we selected 31 phosphorylation sites for quantitative immuno-MRM assay development (Table 1), including targets exhibiting a robust response to DNA damage in the LCL and PBMC datasets, targets showing ATM-dependent phosphorylation, and highly characterized DDR targets curated from the literature.

### 3.2. DDR-2 Immuno-MRM Assay Development

**Figure 1:**
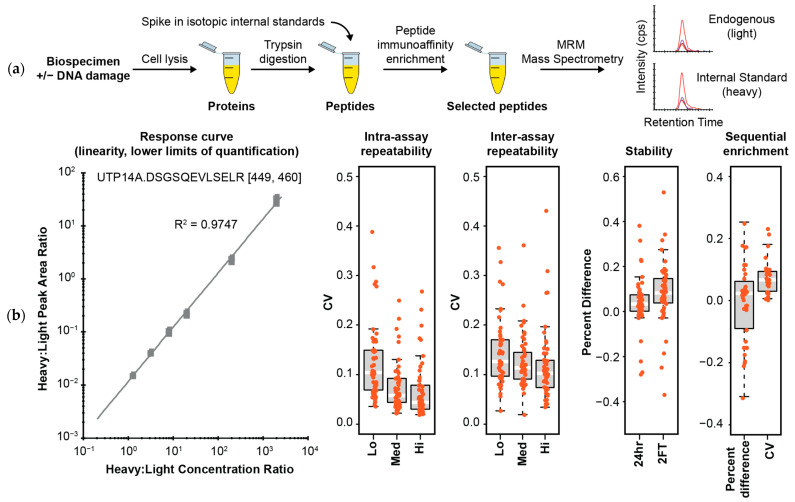
Immuno-MRM enables highly multiplexed protein quantification. (**a**) Assay workflow consists of generation of a protein lysate followed by enzymatic digestion (e.g., trypsin). Stable isotope labeled standards unique to each targeted peptide sequence are spiked to the sample at a known concentration. Custom monoclonal antibodies coupled to magnetic beads are used to enrich the endogenous peptides and labeled standards. The eluate is analyzed by multiple reaction monitoring mass spectrometry, where analyte peptides and internal standards coelute with equivalent relative areas of monitored transitions. High specificity is maintained through optimal selection of fragment ion transitions to monitor. (**b**) Characterization of performance figures of merit of the assay. A representative response curve for the heavy peptide DSGSQEVLSELR spiked into cell lysate shows a typical linear range. Repeatability is characterized by the distribution of CV values for intra- (within day) and inter- (between day) assay repeatability. Each point corresponds to the average CV for a peptide measured at three concentrations, Low (Lo), Medium (Med), and High (Hi) in triplicate over five days (n = 15 at each concentration for a peptide). Stability shows the distribution of percent difference for samples stored at 24 h at 4 °C and after two freeze-thaw cycles relative to immediate analysis. Sequential enrichment shows the distribution of percent difference and CV values following enrichment using the flow-through of a sample from another immuno-MRM assay compared to direct enrichment. For box plots, the white line shows the median value, boxes show the inner quartiles, and the whiskers show 5–95% of data.

To enrich phosphopeptides from cell lysates, we developed a novel panel of monoclonal antibodies to support immuno-MRM assay generation, as previously described [7,26]. The workflow is depicted in Figure 1a. Using established protocols [39], we obtained 35 rabbit monoclonal anti-peptide antibodies for inclusion in the multiplexed assay. In addition to antibodies for the targeted phosphosites, we included custom antibodies for GAPDH, actin, and tubulin to be used as potential sample loading/normalization markers. Notably, antibody clones are screened against both the phosphorylated and the non-phosphorylated versions of the peptide antigens, and where possible, high affinity antibodies are selected that recognize both proteoforms (annotated in Table 1). In addition to their incorporation into immuno-MRM assays, all novel monoclonal antibodies generated in this study were characterized for use in a variety of platforms (Supplemental Table S2 and Supplemental PDF Document S1), and the reagents are offered as a resource to the research community via antibodies.cancer.gov, accessed on 28 July 2021.

To develop the immuno-MRM method, we optimized mass spectrometry conditions for targeted MRM of the phosphopeptides. In the MRM assay, specific precursor and fragment ions pairs (i.e., transitions) are selected in a triple quadrupole MS, resulting in high specificity and sensitivity. We used synthetic peptides to identify the top transitions of each targeted peptide, determine the retention time of each analyte, and determine the optimal collision energy parameters. The antibodies were cross-linked to magnetic beads and were used to enrich the target peptides and stable isotope labeled internal standards. 

### 3.3. Fit-for-Purpose Method Validation of the DDR-2 Immuno-MRM Assay

Performance of the multiplexed DDR-2 immuno-MRM assay was characterized according to published guidelines [40,41] to establish figures of merit for linearity, limits of quantification, repeatability, stability, and use in combination with the previously described DDR-1 multiplexed MRM assay [7] (e.g., sequential enrichment). The linear range and limits of quantification (LOQ) were determined by response curves using a pooled background matrix of protein lysates from LCL-57 cells (+/− 10 Gy IR). Pooled lysates were digested, and 500 µg aliquots were spiked with synthetic peptides; light peptides were added at constant concentration of 200 fmol/mg, and the heavy stable isotope-labeled peptides were serially diluted at concentrations of 2000, 200, 20, 8, 3.2, 1.28, 0.512, 0.2048, and 0 fmol/mg. The monoclonal antibodies were coupled to magnetic beads and used to enrich peptides, following which the eluates were analyzed by MRM. All concentration points were analyzed by four replicates. Peptide specificity was confirmed by equivalent retention time and relative transition areas of light and heavy peptides. Figure 1b shows an example response curve. The peak area ratios (heavy:light) were plotted as a function of analyte concentration to determine the linear range. Lower limits of quantification (LLOQ) were determined by the lowest point with CV < 20%. Figures of merit are reported for each peptide in Supplemental Table S3. Median linear dynamic range was ≥3.2 orders of magnitude with median LLOQ 1.28 fmol/mg (range 0.5–200 fmol/mg). 

Intra-assay (within day) and inter-assay (between day) repeatability were determined by performing complete process triplicate measurements for the multiplexed assay at three concentrations of spiked peptides over 5 multiple days. Heavy peptides were spiked into 500 µg aliquots of the pooled cell lysate background matrix at three concentrations (8, 80, 800 fmol/mg; low, medium, high) with addition of equal amounts of light peptides (200 fmol/mg) to each aliquot. Specificity was confirmed using the same criteria as described above. The median intra-assay variability was 10.4%, 5.8%, 4.1% from low to high concentration samples, and the median inter-assay variability was 12.2%, 11.1% and 10.1% from low to high concentration samples (Figure 1b and Supplemental Table S3). Seven assays showed intra- or inter-assay variability greater than 25% in the low concentration samples due to low signal-to-noise at the lowest concentration values. Two assays (CHK2.TLCG and TP53BP1.pT543pS552.IDED) failed to validate due to consistently high inter-assay variability (range 25–45%). For the CHK2 peptide, this is likely due to instability (see below). For the TP53BP1 peptide, this is likely due to poor detection (e.g., ionization efficiency) of the doubly phosphorylated peptide sequence. 

Peptide stability was evaluated by spiking heavy peptide (200 fmol) into 500 µg digested aliquots of the pooled background lysate. The samples were analyzed after storage at 4 °C on the autosampler for 24 h and after two freeze-thaw cycles; control samples were analyzed immediately. The comparison of peak area ratio between control samples and samples with different handling conditions was used to evaluate peptide stability. The median percent difference relative to the fresh sample was 3.1% after 24 h storage and 7.1% after 2 freeze-thaw cycles, indicating acceptable overall stability for the peptides (Figure 1b and Supplemental Table S3). Seven peptides (CHK2.pT387.TLCG, CHK2.TLCG, CDC25C.SPSM, TP53B.pT543pS552.IDED, TP53B.pT543.IDED, TP53BP1.pS552.IDED, MCM6.pS762.EIES) showed a relative difference greater than 20% after storage at 4 °C for 24 h, indicating these peptides should be analyzed immediately. Four of those peptides were also unstable after 2 freeze-thaws, with an additional 3 peptides (CASP3.pS26.IIHG, ACT.AVFP, SAAL1.NGAA) showing percent difference >20% after 2 freeze-thaws (see Figure 1b and Supplemental Table S3). 

One advantage to immuno-MRM assays is the ability to increase the capacity to analyze more targets by using the flow-through from the antibody-capture step to perform enrichment using another panel of antibodies to different targets (i.e., sequential captures) [23]. We sought to validate the performance of the DDR-2 assay in the flow-through of samples first enriched for other DDR targets using the DDR-1 assay panel [7]. We hypothesized that the order of enrichment would not affect the measured value or the precision of the assay. Using separate aliquots of a common lysate, we compared results from direct enrichment of DDR-2 analytes to those from sequential capture using the flow-through from the DDR-1 panel. All enrichments were performed in triplicate and analyzed by MRM. In total, we detected 35 peptides above the LLOQ in the pooled lysates. Figure 1b shows the distribution of bias (percent difference) for enrichments performed on the flow-through compared to primary enrichments (see Supplemental Table S3 for all data). The median percent difference was 2.2%, showing little difference between the primary DDR-2 enrichments and those conducted on the DDR-1 flow-through samples. Two peptides, CDC25B.SPSM (−30.9%) and TP53BP1.IDED (25%), had a percent difference higher than 25%, likely due to low endogenous signal in the lysate. The distribution of CVs for triplicate analysis in the sequential enrichment format is also shown in Figure 1b. The median CV was 6.7%, and no analytes had CV greater than 25%. These data validate using sequential enrichments with both DDR-1 and DDR-2 MRM assays to increase the number of analytes that can be measured from a single sample aliquot, which is critical in the setting of clinical studies where biospecimens are available in limited amounts.

### 3.4. Quantitative Profiling of Cell Signaling Dynamics in Immortalized Cells Using the DDR-2 Assay

**Figure 2:**
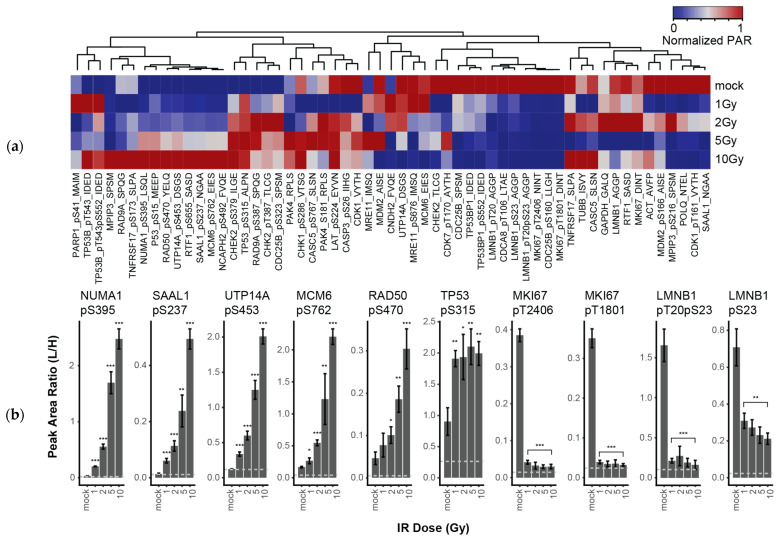
The DDR-2 immuno-MRM assay detects changes in phosphorylation in response to ionizing radiation. (**a**) Heatmap showing unsupervised clustering of analytes detected in LCL-57 exposed to increasing levels of irradiation (1,2,5,10 Gy); control samples were mock-irradiated. All samples were harvested at 1 h. Peak area ratios (light:heavy) were normalized for each peptide analyte. Peptide analyte labels indicated gene symbol, followed by modification site and the first four letters of the peptide sequence. Nonmodified peptides are indicated by “pan”. (**b**) Bar plots showing peak area ratio (light:heavy) of analytes with greater than 2-fold change in concentration detected by immuno-MRM. Error bars show the standard deviation of biological triplicate analysis. In t-test between irradiated and mock results, one asterisk (*) indicates *p* value smaller than 0.05 (*p* < 0.05); two asterisks (**) indicate *p* value smaller than 0.01 (*p* < 0.01); three asterisks (***) indicate *p* value smaller than 0.001 (*p* < 0.001).

To demonstrate proof-of-principle application of the DDR-2 assay to multiplexed measurement of phospho-signaling in response to DNA damage, we used a model system (human LCL-57; ATM +/+) to profile the response to DNA damage after exposure to IR. Independent biological triplicate preparations of LCL-57 cells were treated with increasing doses of IR (1, 2, 5, 10 Gy) and harvested at 1 h; the control cells were mock-irradiated and harvested at 1 h. 500 µg aliquots of lysates were analyzed using the immuno-MRM panel targeting DDR-2 analytes (Table 1). Specificity was confirmed by equivalent retention time and relative transition areas of internal standards. Overall, we detected 43 peptides (including 26 phosphopeptides and 17 nonmodified peptides) above LLOQ in the lysates. Figure 2a shows a heatmap of the responses measured in the dose curve experiment (raw data are available in Supplemental Table S4). Phosphorylation response was seen to go in both directions, with two notable clusters in the heatmaps for down-regulated (i.e., decreasing with irradiation) and up-regulated (i.e., increasing with irradiation) phosphopeptides. Six phosphopeptides showed a greater than 2-fold increase in response to DNA damage (no nonmodified peptides showed >2-fold change). NUMA1 pS395, SAAL1 pS237, UTP14A pS453, MCM6 pS762, RAD50 pS470, and TP53 pS315 were elevated after IR and show noticeable dose-dependence (Figure 2b). NUMA1 phosphorylation has been identified in phosphoprofiling of the DDR [42] and was linked to ATM signaling in response to IR [43], in agreement with our results. There is also evidence that NUMA1 phosphorylation by ATM is required for proper bipolar mitotic spindle formation, providing further evidence for a causal relationship between ATM and NUMA1 [44]. Finally, there were four phosphopeptides showing decreased levels in response to DNA damage (plotted in Figure 2b). Interestingly, unlike the sites with increased phosphorylation levels post-IR, the sites showing decreased levels did not appear to show any dependence on the dose of radiation. 

**Figure 3:**
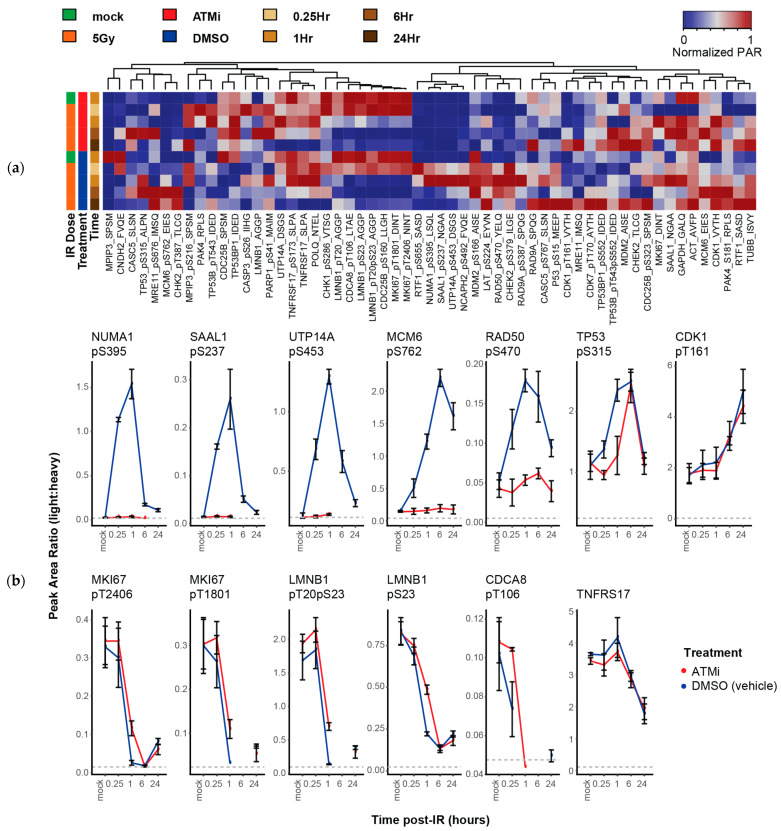
The DDR-2 immuno-MRM assay detects changes in phosphorylation dynamics due to ATM kinase inhibition. (**a**) Heatmap showing unsupervised clustering of analytes in LCL-57 exposed to ionizing radiation in the presence of ATM inhibitor (or control vehicle, DMSO). Peak area ratios (light:heavy) were normalized for each peptide analyte. (**b**) Bar plots showing peak area ratios (light:heavy) of analytes with greater than 2-fold change quantified by immuno-MRM. Blue lines show control (DMSO vehicle) and red lines show samples treated in the presence of ATM inhibitor. Error bars the standard deviation of biological triplicate analysis.

Having established that the DDR-2 assay can profile quantitative changes in phosphorylation in response to DNA damage, we next tested use of the assay to quantify the effects of pharmacological inhibition of DDR kinase activities. We hypothesized that the multiplexed assay could profile changes in signaling dynamics, especially for ATM-dependent phosphosites. LCL-57 (Atm+) cells were treated with or without ATM-kinase inhibitor (KU-55933) prior to 5 GY IR induction; control samples were treated with DMSO vehicle. Cells were prepared in biological triplicate using separate aliquots in the presence/absence of the inhibitor and harvested over a time-course of 0.25, 1, 6, and 24 h post-irradiation; controls were harvested 1 h following mock-irradiation. Lysates were analyzed by the DDR-2 multiplexed immuno-MRM assay. Overall, we detected 42 peptides (25 phosphopeptides and 17 nonmodified peptides) above LLOQ in the lysates. Figure 3a shows the heatmap of peak area ratios detected above LLOQ (raw data are available in Supplemental Table S4). There were three notable clusters in the heatmap. The first two were attributed to analytes that show an increase or decrease over time following IR. The third cluster was a large cluster of phosphopeptides that showed increased levels following DNA damage in the absence but not the presence of the ATM inhibitor. 

There were 13 peptides (12 phosphopeptides, 1 nonmodified) that showed a greater than 2-fold change in either direction in response to DNA damage (plotted in Figure 3b). In addition to the phosphosites measured to increase with DNA damage in the dose-curve experiment above, we detected an increase of CDK1 pT161 at 6 h and 24 h post-irradiation. Of the phosphopeptides that showed an increase in phosphorylation with DNA damage, only CDK1 pT161 was not ATM-dependent (TP53 pS315 showed an increase with ATM inhibition at later timepoints). Phosphorylation of NUMA1 pS395, SAAL1 pS237, UTP14A pS453, MCM6 pS762, and RAD50 pS470 was affected by presence of the ATM inhibitor, indicating these phosphosites were dependent on ATM activity. The time-course data showed some interesting trends, where RAD50 pS470, NUMA1 pS395, UPT14A pS453 and SAAL1 pS237 phosphorylation reached a maximum at the 1 h timepoint post-IR induction, whereas MCM6 pS762 phosphorylation kept increasing until 6 h post-exposure to IR in the absence of ATM inhibitor. Except for MCM6 pS762, phosphosites induced post-IR recovered (or approached) their basal levels after 24 h. In contrast, targets that decreased after DNA damage (MKI67, LMNB1) were cell cycle-related, i.e., they were not directly involved in DNA damage repair, and these levels did not recover after 24 h.

### 3.5. Pharmacodynamic Profiling of Kinase Inhibition in Primary Human Cells

As discussed above, analysis of biomarkers in peripheral blood is becoming increasingly important in clinical trials for POM and PD studies, to help guide dose and scheduling of therapeutics. From a single blood draw, peripheral blood mononuclear cells can be isolated and processed to analyze and quantify protein markers [45]. We sought to evaluate the utility of the DDR-2 assay in pharmacodynamic profiling in both proliferating and non-proliferating primary human PBMCs in the presence/absence of DDR kinase inhibitors. In addition to ATM inhibition, we used the assay to profile the response to inhibitors of ATR serine/threonine kinase (HGNC: ATR), AZD6738 [46], and DNAPK (DNAPK; HGNC: PRKDC), AZD7648 [47]; control samples were treated with vehicle (DMSO) only.

PBMCs were isolated from 3 donors and split into two aliquots; one aliquot was expanded in culture with Staphylococcal Enterotoxin B (SEB+) activation, and the other aliquot had no stimulation (SEB−). For each set of cells, DNA damage was induced by 5 Gy IR, controls were mock-irradiated, and cells were harvested 1 h post-IR. Having validated (above) the sequential use of our DDR-1 [7,26] and DDR-2 (this study) assays, we applied the DDR-1 assay [7,26] to 500 µg aliquots of PBMC lysates and applied the DDR-2 assay to analyze the flow-through. For the ATM inhibitor and no inhibitor samples, lysates from three individual donors were used as “biological replicates”. 

**Figure 4:**
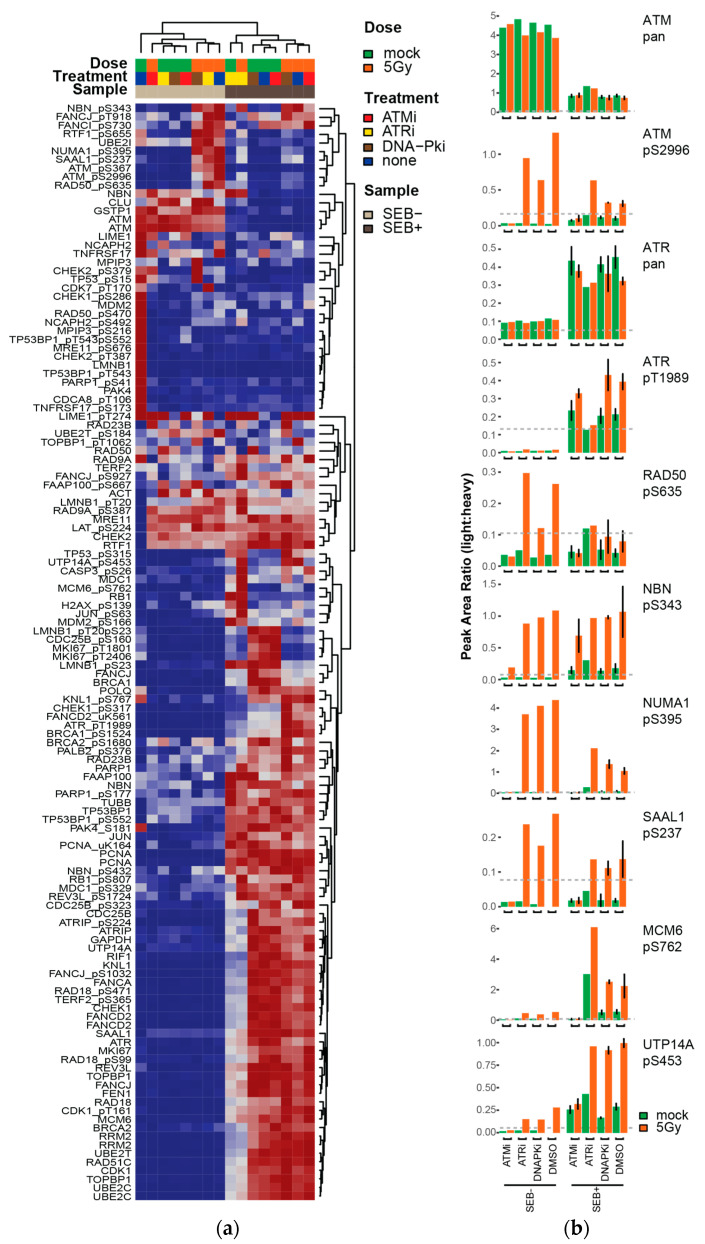
Pharmacodynamic profiling of cell signaling dynamics. (**a**) Heatmap showing unsupervised clustering of analytes and samples for PBMCs exposed to ionizing radiation in the presence of ATM, ATR, or DNA-PK kinase inhibitors. Peak area ratios (light:heavy) were normalized for each peptide analyte. (**b**) Bar plots showing peak area ratios (light:heavy) for selected analytes. For each kinase inhibitor, the pair of mock-treated (green) and irradiated (orange) cells are plotted. Error bars show the standard deviation of triplicate analysis (SEB+, ATMi and DMSO samples) or the range of duplicate analysis (SEB+, DNAPKi samples). To meet the assay sample requirements, two individuals (i.e., biological duplicate reps) were used for DNAPKi samples, and a single individual sample was used (i.e., singlicate analysis) for the ATRi samples. For the unstimulated cells (SEB−), lysates from two individuals were pooled to obtain sufficient material (i.e., singlicate technical analysis).

Overall, 101 peptides (41 phosphorylated, 60 unmodified) were detected above LOQ in the PBMCs (Supplemental Table S5). The samples primarily clustered (Figure 4a) according to cell culture conditions (e.g., SEB+/−), and the heatmap shows a clear cluster of proteins expressed in stimulated PBMCs (SEB+) that were not detected, or detected at low expression levels, in the unstimulated cells (SEB−). Interestingly, within the SEB+/− clusters, the PBMCs were predominantly grouped into two main clusters according to DNA damage (e.g., 5 Gy IR vs. mock).

The expression of individual proteins and their phosphorylation levels help to elucidate the pharmacodynamics of treatment with the kinase inhibitors in these PBMCs (see Figure 4b). Autophosphorylation of ATM is associated with the activation of the DDR [5]. Overall expression levels of nonmodified ATM were found to be higher in non-stimulated (SEB−) cells and, as expected, we detected increased autophosphorylation of ATM pS2996 following irradiation (Figure 4b). In the presence of ATM inhibitor, ATM pS2996 phosphorylation was not detected after irradiation, consistent with expectations [6,48] and confirming that ATM activity is inhibited upon treatment. Similarly, Figure 4b shows ATR expression was detected in both groups of cells, with higher expression levels in the stimulated SEB+ cells. Auto-phosphorylation of ATR pT1989 [49,50] was detected in all irradiated SEB+ cells, but not in the presence of ATRi. Unlike ATM, phosphorylation of ATR was not detected in SEB- cells, and non-modified ATR was detected at very low levels, possibly reflecting higher expression in proliferating cells [51]. These measurements confirm activity of the kinase inhibitors.

Consistent with expectations [29], RAD50 pS635 and NBN pS343 show reduced levels in the presence of ATM inhibitor (Figure 4b). Likewise, changes observed in the LCL demonstration experiments (above) were recapitulated in the PBMCs. For example, NUMA1 pS395, SAAL1 pS237, MCM6 pS762, and UPT14A pS453 phosphorylation all significantly increased with DNA damage compared to mock, but showed a marked decrease in activity in the presence of the ATM inhibitor (Figure 4b; note RAD50 pS470 was not detected above LLOQ in the PBMC samples). In contrast, these phosphorylation sites showed increased levels in the presence of ATRi and DNAPKi, suggesting that they were dependent upon ATM activation but not ATR/DNAPK.

**Figure 5:**
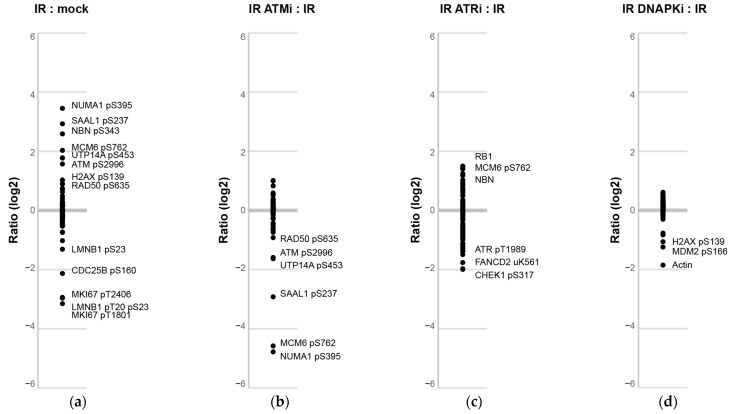
Pharmacodynamic markers of kinase inhibition identified by comparing protein and phosphorylation levels in PBMCs. The ratio of concentration levels in PBMCs (SEB+) determined by the immuno-MRM assay is plotted for four comparisons: (**a**) 5 Gy versus mock-irradiation, (**b**) irradiated in the presence of ATM inhibitor versus vehicle (DMSO), (**c**) irradiated in the presence of ATR inhibitor versus vehicle (DMSO), and (**d**) irradiated in the presence of DNAPK inhibitor versus vehicle (DMSO). The log(2) ratio is plotted for each comparison. Large changes are labeled with the analyte and phosphorylation site (if applicable).

Potential pharmacodynamic markers of kinase inhibition can be examined by evaluating the fold change (log_2_) of peptide and phosphopeptide levels quantified in irradiated PBMC (SEB+) samples in the presence of inhibitors compared to the absence of inhibitors (Figure 5). Changes seen in irradiated versus mock-irradiated cells are plotted in Figure 5a. As observed above, large increases were evident in phosphorylation activity, including increases in NUMA1 pS395, SAAL1 pS237, NBN pS343, ATM pS2996, and RAD50 pS635, as well as large decreases in the phosphosites characterized above (e.g., MKI67 pT1801/pT2406 and LMNB1 pT20/pS23). The effects of inhibition on the ATM kinase are shown in Figure 5b. The largest difference is seen for NUMA1 pS395. Using the DDR-1 assay [7], we previously identified RAD50 pS635 as a novel PD biomarker of ATM inhibition [29]. It is notable that NUMA1 pS395 shows a 14.5-fold greater difference (compared to RAD50 pS635) in this experiment and presents a good candidate for further study as a putative more robust PD marker of ATM inhibition. Finally, differences due to ATRi and DNAPKi are plotted in Figure 5c,d. For ATRi, a decrease in ATR pT1989 levels was seen, as well as decreases in CHEK1 pS317, a target of ATR [52]. Decreased levels of H2AX pS139 and MDM2 pS166 were seen in the presence of the DNAPKi. Since we targeted phosphosites dependent on activity of the ATM kinase, it was not surprising that we found the largest effects on phosphorylation levels in the samples with ATM kinase inhibitor (Figure 5b).

## 4. Discussion

Quantitative targeted proteomics using immuno-MRM provides a powerful tool for profiling changes in cell signaling dynamics. We previously demonstrated the utility of multiplexed MS-based assays for quantitative profiling of the cell signaling responses to DNA damage [7,16,26,27]. The DDR-1 assay [7] was successfully deployed to identify RAD50 pS635 as a novel PD biomarker for ATM inhibition [29]. This success prompted us to expand the approach to include assays for quantifying additional phosphorylation events in the DDR. We describe a new multiplexed assay panel (DDR-2) that provides quantitative assays to 51 peptides (33 phosphopeptides) representing 31 proteins. Analytical validation of the assay showed it can be used in conjunction with the DDR-1 assay without requiring additional sample material. All assay protocols, validation data, and 35 monoclonal antibodies are accessible to the community via the NCI’s assay (https://assays.cancer.gov, accessed on 28 July 2021) and antibody portal (https://antibodies.cancer.gov, accessed on 28 July 2021) [41,53], providing a resource to the research community for quantitative studies in cell signaling, pharmacodynamic profiling, and mechanism of action studies.

Traditional antibody-based immunoassays have several shortcomings, such as detecting one target at a time, producing semi-quantitative results (limiting quantitative comparisons across different assays and epitopes), and being susceptible to interferences. The immuno-MRM assay overcomes these limitations and enables robust (specific, precise, multiplex) quantification of DNA damage biomarkers. Unlike in traditional immunoassays, where detection is based on a surrogate signal (e.g., fluorescent tag), the mass spectrometer is used directly to measure the target analyte. The combination of retention time and relative intensity of multiple transition (i.e., fragment) ions between the analyte and internal standard allows for near-absolute specificity and allows for some relaxed requirements in antibody specificity. The assay was characterized in a human cell lysate, which is the matrix of intended use. Historically, assay performance is consistent across sample types, but fit-for-purpose validation in the intended sample of use is recommended [54]. Interferences can be detected in analytical validation studies and removed or avoided. The use of stable isotope labeled internal standards enables quantification over several orders of magnitude dynamic range and provides a manner for standardization across multiple laboratories, making the approach suitable for clinical implementation. The high specificity of MRM coupled to the large linear range means that multiplexing can be readily achieved.

The proof-of-principle demonstration of this new DDR-2 assay for quantifying the DNA damage response in non-stimulated PBMCs isolated from individuals shows the potential for use of the assay in providing pharmacodynamic markers for kinase inhibitors in clinical trials [45]. To be successful in this setting, there are some challenges that must be overcome. One challenge is the availability of clinical material. For this experiment we used 500 µg of protein for input to the assay, which requires roughly 8 mL of blood collection (i.e., >10^6^ cells), which is unlikely to be applicable in samples with low cell counts (e.g., material obtained from circulating tumor cells). Improvements in assay sensitivity (including optimization of recovery, using the highest affinity antibodies, optimizing addition-only single reaction vessel protocols, and optimizing chromatographic conditions for specific subsets of peptides (e.g., phosphopeptides)) are ongoing and could accommodate lower amounts of sample input. In addition to obtaining sufficient material, inducing DNA damage in the primary cells appears necessary for detection of some responses in the pathway and could pose challenges in a clinical assay. To overcome this hurdle, DNA damaging agents could be introduced following collection, prior to fixation or freezing, in a manner similar to that performed for immune profiling experiments [55]. Another challenge is overcoming preanalytical variables during sample collection. Notably, some phosphosignaling is susceptible to ischemic effects in tissue collection [56], potentially introducing preanalytical variations [45]. Translation of quantitative phosphorylation assays would require strong validation studies to ensure stability of the phosphopeptides. Despite the challenges, progress is being made in advancing MRM proteomics to the clinic. For example, MRM-based assays have been used clinically for thyroglobulin [57,58], and standards for protein quantification by MRM are in the process of being developed [40].

## 5. Conclusions

The multiplex DDR-2 immuno-MRM assay described in this report enables precise and highly specific quantification of 51 analytes in the DDR, including potential PD biomarkers of ATM kinase inhibition. Based on the proof-of-concept data presented herein, this assay has many potential applications in basic and translational research.

## Figures and Tables

**Table 1:** Peptides targeted for the multiplexed DDR-2 immuno-MRM assay panel. Modifications of “(ph)” indicate phosphorylation. Site of phosphorylation refers to the amino acid position in the protein.

Gene	Protein Acc ID	Peptide Modified Sequence	Phosphorylation Site
CHEK1	sp|O14757|	VTSGGVSES(ph)PSGFSK	pS286
LAT	sp|O43561|	EYVNVS(ph)QELHPGAAK	pS224
POLQ	sp|O75417|	NTELNEEQEVISNLETK	
PAK4	sp|O96013|	RPLS(ph)GPDVGTPQPAGLASGAK	S181
CHEK2	sp|O96017|	ILGETS(ph)LMR	pS379
CHEK2	sp|O96017|	TLCGT(ph)PTYLAPEVLVSVGTAGYNR	pT387
CHEK2	sp|O96017|	TLCGTPTYLAPEVLVSVGTAGYNR	
GAPDH	sp|P04406|	GALQNIIPASTGAAK	
TP53	sp|P04637|	ALPNNTSSS(ph)PQPK	pS315
TP53	sp|P04637|	MEEPQSDPSVEPPLS(ph)QETFSDLWK	pS15
CDK1	sp|P06493|	VYT(ph)HEVVTLWYR	pT161
CDK1	sp|P06493|	VYTHEVVTLWYR	
TUBB	sp|P07437|	ISVYYNEATGGK	
PARP1	sp|P09874|	MAIMVQS(ph)PMFDGK	pS41
LMNB1	sp|P20700|	AGGPTT(ph)PLSPTR	pT20
LMNB1	sp|P20700|	AGGPTTPLS(ph)PTR	pS23
LMNB1	sp|P20700|	AGGPTT(ph)PLS(ph)PTR	pT20pS23
CDC25B	sp|P30305|	LLGHS(ph)PVLR	pS160
CDC25B	sp|P30305|	SPS(ph)MPCSVIRPILK	pS323
CDC25B	sp|P30305|	SPSMPCSVIRPILK	
CDC25C	sp|P30307|	SPSMPENLNRPR	
CASP3	sp|P42574|	IIHGSES(ph)MDSGISLDNSYK	pS26
MKI67	sp|P46013|	DINTFLGT(ph)PVQK	pT1801
MKI67	sp|P46013|	DINTFLGTPVQK	
MKI67	sp|P46013|	NINTFVET(ph)PVQK	pT2406
MRE11	sp|P49959|	IMSQSQVSK	
CDK7	sp|P50613|	AYT(ph)HQVVTR	pT170
ACT	sp|P68133|	AVFPSIVGRPR	
MDM2	sp|Q00987|	AIS(ph)ETEENSDELSGER	pS166
TNFRSF17	sp|Q02223|	SLPAALS(ph)ATEIEK	pS173
TNFRSF17	sp|Q02223|	SLPAALSATEIEK	
TP53BP1	sp|Q12888|	IDEDGENT(ph)QIEDTEPMS(ph)PVLNSK	pT543pS552
TP53BP1	sp|Q12888|	IDEDGENT(ph)QIEDTEPMSPVLNSK	pT543
TP53BP1	sp|Q12888|	IDEDGENTQIEDTEPMS(ph)PVLNSK	pS552
TP53BP1	sp|Q12888|	IDEDGENTQIEDTEPMSPVLNSK	
MCM6	sp|Q14566|	EIESEIDS(ph)EEELINK	pS762
MCM6	sp|Q14566|	EIESEIDSEEELINK	
NUMA1	sp|Q14980|	LSQLEEHLS(ph)QLQDNPPQEK	pS395
CDCA8	sp|Q53HL2|	LTAEAIQT(ph)PLK	pT106
NCAPH2	sp|Q6IBW4|	FVQETELS(ph)QR	pS492
KNL1	sp|Q8NG31|	SLS(ph)NPTPDYCHDK	pS767
KNL1	sp|Q8NG31|	SLSNPTPDYCHDK	
RTF1	sp|Q92541|	SASDLS(ph)EDLFK	pS655
RTF1	sp|Q92541|	SASDLSEDLFK	
RAD50	sp|Q92878|	YELQQLEGS(ph)SDR	pS470
SAAL1	sp|Q96ER3|	NGAAQPLDQPQEES(ph)EEQPVFR	pS237
SAAL1	sp|Q96ER3|	NGAAQPLDQPQEESEEQPVFR	
RAD9A	sp|Q99638|	SPQGPSPVLAEDS(ph)EGEG	pS387
RAD9A	sp|Q99638|	SPQGPSPVLAEDSEGEG	
UTP14A	sp|Q9BVJ6|	DSGS(ph)QEVLSELR	pS453
UTP14A	sp|Q9BVJ6|	DSGSQEVLSELR	

## Data Availability

Shotgun proteomics data have been deposited to the ProteomeXchange Consortium via the PRIDE partner repository with the dataset identifier PXD026103, accessed on 28 July 2021. Assay characterization data are available via the CPTAC Assay Portal (assays.cancer.gov, accessed on 28 July 2021) and Antibody Portal (antibodies.cancer.gov, accessed on 28 July 2021). Raw peak area ratios for all targeted peptides are provided in the Supplemental Tables and Panorama Public (https://panoramaweb.org/DDR2_ATMprofiling.url, accessed on 28 July 2021).

## Supplementary Materials

The following are available online at https://www.mdpi.com/article/10.3390/cancers13153843/s1, Table S1: Phosphoprofiling results of LCL-57 and LCL-26 cells +/− IR, Table S2: Antibody characterization data for immunoassay applications, Table S3: Fit-for-purpose bioanalytical validation of the immuno-MRM assay, Table S4: Immuno-MRM results for LCL experiments, Table S5: Immuno-MRM results for PBMC experiments, PDF Document S1: Results for extensive antibody characterization.
