# Supplementary material for: Targeted Mass Spectrometry Enables Quantification of Novel Pharmacodynamic Biomarkers of ATM Kinase Inhibition
Source: Cancers (Basel). 2021 Jul 30;13(15):3843. doi: 10.3390/cancers13153843 (PMC8345163; doi:10.3390/cancers13153843)
Supplement: Supplementary file 1 [file cancers-13-03843-s001.zip › SupplementaryMaterials/Supplemental_Material_Antibody_Characterization_Results.pdf]

Antibody characterization supplemental information

Index

A. Project Description……………………………………………………………..……Page 3.
B. Methods…………………………………………………………………………….…...Page 6.
C. Tables………………………………………………………………………………..….Page 11.
D. Results……………………………………………………………………………..……Page 26.
E. Summary……………………………………………………………………………..….Page 179.
F. References……………………………………………………………………………Page 183.

A. Project Description

A panel of 39 rabbit anti-human antibodies were characterized in the Antibody Characterization Laboratory (ACL) at the Frederick National Laboratory for Cancer Research facility in Frederick, Md. The testing strategy that was used in this characterization is depicted in Figure 1A and Figure 1B. Briefly, each antibody was first screened by immunoblot assay against commercial purified recombinant protein. Clones that were positive in this primary screen were subsequently tested for their ability to detect endogenous protein in three distinct human cell lines: HeLa, MCF10A, and LCL57 by immunoblot assay. These positive clones were further characterized in immunofluorescence (IF), single cell western blot (SCWB), and immunoprecipitation (IP) assays. Clones that were negative in the primary screen were evaluated for potential phospho-specificity. All 39 clones were also screened by immunohistochemistry. Clones that were positive in this screen were subsequently tested in 59 of the 60 cancer cell lines in the NCI-60 protein array. This report describes the methods that were used to characterize the antibodies and summarizes the results that were obtained for each test as positive, negative, weak, uncertain, not tested, or to be determined accordingly.

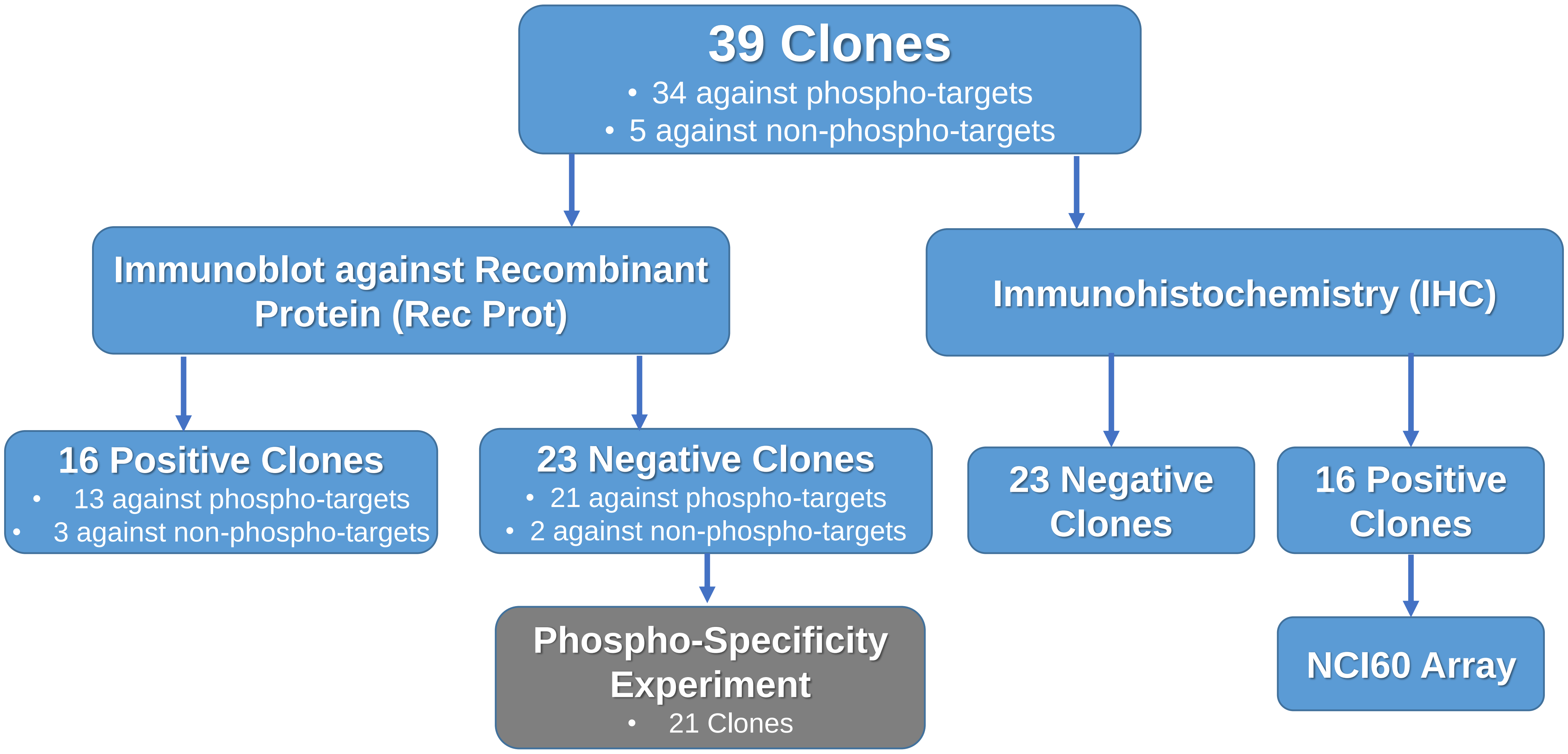

Figure 1A. Flow chart for NCI56 antibody characterization primary screen

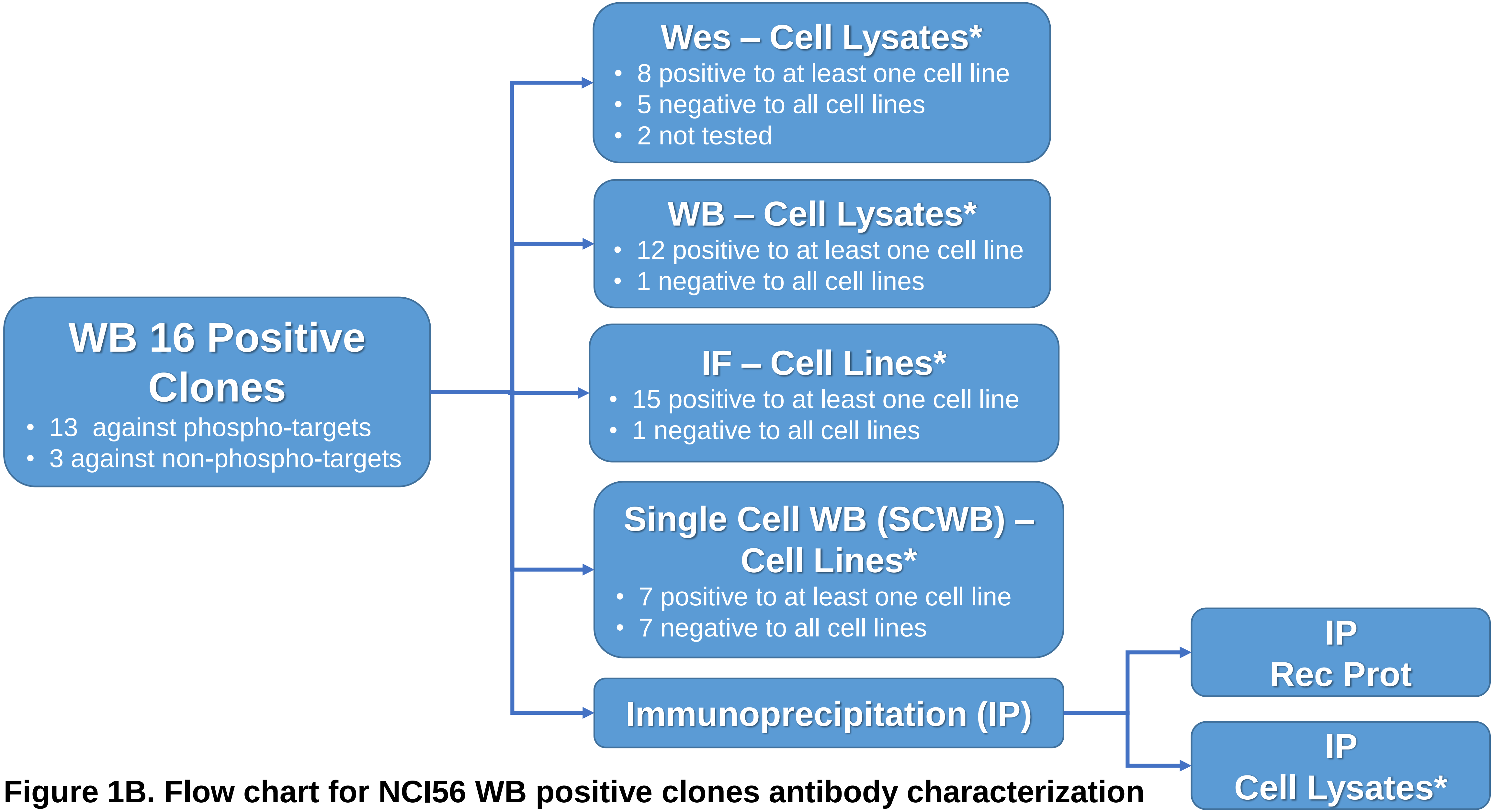

Figure 1B. Flow chart for NCI56 WB positive clones antibody characterization

*** Cell lines - HeLa, MCF10A, LCL57**

B. Methods

Cell Culture and Lysis

Cell lines LCL57 (Coriell Institute) and HeLa (American Type Culture Collection ,ATCC) were cultured according to manufacturers' specifications. Empty vector transfected MCF10A-EV cells were a kind gift from James A. Wells (Department of Pharmaceutical Chemistry, University of California, San Francisco, CA) and were cultured according to Dr. Wells' specifications. Briefly, MCF10a-EV cells were cultured in DMEM/F-12 (Thermo Fisher Scientific) supplemented with 5% Horse Serum (Thermo Fisher Scientific), 20 ng/mL Epidermal Growth Factor (Thermo Fisher Scientific), 10 µg/mL Insulin (Sigma), 0.5 µg/mL Hydrocortisone (Sigma) and 100 ng/mL Cholera Toxin (Sigma) at 37°C in a humidified 5% carbon dioxide incubator. LCL57, HeLa and MCF10A-EV cells were cultured and harvested according to manufacturers' specifications to obtain cell pellets. Briefly, once cells were subcultured and after trypsinization, cells were centrifuged at 1500 rpm for 6 minutes and supernatant was removed and discarded. Cells were washed with 1X PBS, centrifuged as before, resuspended in 1X PBS and counted. Cells were split according to desired cell number in 15 mL centrifuge tubes and centrifuged as before. Centrifuge tubes with pellets were stored at -80°C until lysis. Cell pellets were lysed using RIPA lysis and extraction buffer (Thermo Fisher Scientific) following the manufacturer's protocol. Mammalian Protease Inhibitor (VWR) was added according to instructions. Lysate protein concentration was estimated with a BCA assay (Pierce) as described by the vendor.

Immunoblot

Recombinant proteins were obtained from Origene (Rockville, MD) or Novus Biologicals (Littleton, CO); see "Western blot data and table" for catalog numbers. Western immunoassays were performed using traditional immunoblotting techniques according to the designed protocol described in the antibodies.cancer.gov SOP M-103, with the following modifications. Validation of proper protein sample loading was done using Bio-Rad Stain-Free precast gel confirmation. Traditional immunoblotting for recombinant protein were conducted at a concentration of 10 ug/ml in reducing conditions (200 ng total protein). Whole cell lysates were diluted to 2.5 mg/ml in reducing conditions (50 ug total protein). Transfer of protein from Bio-Rad pre-cast stain free gels were performed by Bio-Rad Turbo-Blot at "High MW" setting for 10 minutes. Blocking of the membrane was performed using Bio-Rad Blotting Grade Blocker at 5% in 1X PBS-0.5% Tween-20. Primary antibodies (1 mg/mL) were diluted in 1X PBS-0.5% Tween-20 to a dilution of 1:5000 at a total volume of 25 mL. Washing of membrane was conducted using 1X PBS-0.5% Tween-20 three times. Secondary HRP-linked rabbit specific antibody (Jackson ImmunoResearch, West Grove, PA) was diluted at 1:5000 in 1X PBS -0.5%Tween-20 at a volume of 25 mL. Immuno-detection was performed using colorimetric substrate Opti-4CN (Bio-Rad) at 1 mL per blot. Development of immunoblot was captured using Bio-Rad ChemiDoc MP imaging system.

WES System

The Simple Western (Wes-ProteinSimple, USA) system was used to detect primary antibody binding to a target protein in cell lysates (HeLa, MCF10A, LCL57). The Simple Westerns were performed following the procedures detailed in the antibodies.cancer.gov SOP M-134 with modifications. Cell lysates were run using the 12-230 kDa separation module, 8 x 25 capillary cartridges (ProteinSimple) and detected with Anti-Rabbit Detection Module (ProteinSimple). Irradiated and phosphatase treated cell lysates were run at a concentration of 200 ug/mL and incubated with primary antibodies diluted 1:500.

Phospho-Specificity Experiment

Monoclonal antibodies raised against phosphorylated peptides that did not recognize the recombinant protein in WB were tested for their phospho-specificity. For this experiment non-treated cells (HeLa, MCF10A, and LCL57) versus cells treated with irradiation as previously described (Whiteaker JR, Zhao L, Saul R, et al. A Multiplexed Mass Spectrometry-Based Assay for Robust Quantification of Phosphosignaling in Response to DNA Damage. *Radiat Res*. 2018;189(5):505-518. doi:10.1667/RR14963.1) were used. Cell pellets and irradiated cell pellets (positive controls) were lysed in ice cold lysis buffer (50 mM Tris, 10 mM NaCl, 5.0 mM $MgCl_2$, 0.1 mM $ZnCl_2$, 1% Triton X-100, 1.0 mM DTT, pH 8.0) for 20 minutes. Lysis buffer was supplemented with Mammalian Protease Inhibitor (VWR) on the day of lysis. The lysates were clarified by centrifugation at 14,000 rpm for 10 minutes at 4°C. Clarified lysate was removed and the protein concentration was quantified with the BCA protein assay kit (Pierce). Phosphatase treated lysates (negative controls) were treated with alkaline phosphatase enzyme from bovine intestinal mucosa (Sigma) for 1 hour at 37°C. Reactions were stopped by placing samples at -80°C until immunoblot analysis.

Immunofluorescence (IF) Staining

The Human Protein Atlas Cell Atlas Standard Immunostaining Protocol was followed with minor modifications. Briefly, before starting, adherent cells were cultured overnight at 10,000 cells/well in 100 µL cell culture media in a 12.5 µg/mL superfibronectin (Sigma) coated 96-well glass bottom microplate (Sigma) and suspension cells were cultured in a Shi-fix™ coated 96-well microplate (Everest Biotech) according to manufacturer's instructions. The following day, cell culture media was removed from cells and cells were washed with 1X Phosphate Buffered Saline (PBS) (Fisher Scientific), fixed with 4% paraformaldehyde (VWR)/1X PBS, permeabilized with 0.1 % Triton X-100 (Sigma)/1X PBS, washed with 1X PBS and incubated with diluted primary antibodies (2 µg/mL unless otherwise stated) with 1 µg/mL mouse anti-alpha tubulin (Abcam) in blocking buffer (4% Fetal Bovine Serum (Thermo Fisher Scientific)/1X PBS)) overnight at 4°C. The next day, primary antibodies were removed from cells and cells were washed with 1X PBS, incubated with diluted secondary antibodies (2.5 µg/mL anti-rabbit Alexa Fluor 488 (Thermo Fisher Scientific) with 2.5 µg/mL anti-mouse Alexa Fluor 555 (Thermo Fisher Scientific) in blocking buffer) for 1.5 hours at room temperature (RT) in darkness. With minimum light present, secondary antibodies were removed from cells and cells were stained with 0.2 µg/mL DAPI (VWR)/1X PBS, washed with 1X PBS and wells were filled with glycerol (Thermo Fisher Scientific)/10X PBS (Fisher Scientific). The 96-well microplate was sealed with an adhesive aluminum PCR plate seal (VWR) and stored at 4°C until IF analysis (Invitrogen™ EVOS™ M5000 Imaging System, (Thermo Fisher Scientific)).

Single-Cell Western Blot (SCWB)

The Standard scWest Kit (ProteinSimple) was used and the kit protocol was followed. Briefly, cells were diluted to 100,000 cells/mL with 1X Suspension Buffer (SB)(ProteinSimple) and 1 mL cells was added to the chip to allow cells to settle for 15-20 mins. Chip with cells was rinsed with 1X SB, ran on Milo (ProteinSimple) where cells are lysed, separated and captured, washed with 1X Wash Buffer (WB) (ProteinSimple), incubated with primary antibody for 2 hours at RT, washed with 1X WB, incubated with fluorescently labeled secondary antibody (R&D System, Inc.) for 1 hour at RT, washed with 1X WB, rinsed with deionized water, spun dry on a Microarray Slide Spinner (Sigma Aldrich), imaged in a microarray scanner (ProteinSimple) and resulted image analyzed using Scout software (ProteinSimple).

Immuno-Precipitation Mass Spectrometry (IP-MS)

The antibodies were tested by IP-MS against recombinant proteins and against lysates of selected cell lines by grouping the antibodies into two multiplexed groups with six to 8 mAbs per group. Both recombinant proteins (table 1) and monoclonal antibodies were diluted in 1X PBS/0.05% CHAPS at a concentration of 50 mg/mL. Equal volumes of antibodies and antigens (100 uL) were mixed in a 96 deep well plate with 5 uL of Pierce™ Protein G Magnetic Beads that were previously washed with 1X PBS/0.05% CHAPS. The plate was incubated overnight at 4°C with shaking in an Eppendorf Thermomixer. Bead washes (1X PBS/0.05% CHAPS) and elution (8M Urea/50 mM Tris/100 mM DTT elution plate) from the plate was performed automatically in a Thermo Fisher Scientific KingFisher Flex Magnetic Particle Processor. Eluates were alkylated with iodoacetamide (final concentration 50 mM) for 30 min at RT in the dark and desalted using Amicon® Ultra Centrifugal Filters, MW CO 3KDa (Millipore Sigma) until nominal urea concentration was <1M. The spin filters were transferred to a clean collection tube and the solution in the filters was spiked with 1 mg of Trypsin / Lys-C Mix, Mass Spec Grade (Promega). Negative controls (beads and recombinant protein, without antibody) were treated exactly the same as the IP samples. Protein and trypsin mixture were incubated overnight at 37°C in water vapor saturated incubator with shaking. Digested samples were spun and tryptic peptides were recovered in the collection tube by spinning. Samples were desalted with a SOLAμ™ SPE HRP Plate according to vendor instructions, dried in a speed-vac device and reconstituted in 0.1% formic acid. The peptide digests were resolved using a nano-flow LC system (EASY-nLC 1200, Thermo Scientific) coupled on-line to a hybrid ion trap-orbitrap mass spectrometer (Orbitrap Elite, Thermo Scientific). Samples were injected onto 20 mm long, 75 μm inner diameter, C18, 3 μm particle size trapping column (EASY-Spray, Thermo Scientific) and separated using in line, 150 mm long, 75 μm inner diameter, C18, 2 μm particle size, analytical column (EASY-Spray, Thermo Scientific). The linear gradient for separation was 5–40% mobile phase B over 62 min at 300 nl/min flow rate, where mobile phase A was 0.1% formic acid in water, and mobile phase B was 0.1% formic acid in 80% acetonitrile. Mass spectrometer was operated in a data dependent mode scanning, using the ion mass to charge range of 350 - 1650, monitored at the resolution level of 60000 at m/z 400. Each MS1 scan was followed by MS2 scan, wherein the 20 most abundant precursor ions were dynamically selected for collision-induced dissociation using normalized collision energy of 35%. Proteins were identified applying the SEQUEST HT algorithm search against the non-redundant human proteome database (i.e., SwissProt release v57.15) utilizing the software Proteome Discoverer 1.4 (Thermo Scientific). The database search thresholds included: for the monoisotopic peptide precursor ions (i.e., MS1 spectra) mass tolerance was set at 10 ppm and for the fragment ions (i.e., data-dependent MS2 spectra) mass tolerance was set at 0.6 Da. Dynamic amino acid modifications were added for the detection of the following: +57.021 Da for cysteine. IP experiments were also performed with LCL57, HeLa and MCF10A cell lysates. Cell were lysed as described above. Lysate protein concentration was estimated with a BCA assay (Pierce). Each cell lysate was incubated with 5 ug of antibody and 5 mL of Protein G beads. Pull-down, proteolytic digestion and analysis were performed as described above for the recombinant protein experiment. IP data were compared with negative controls (beads and lysate digests, without antibody).

Immunohistochemistry Pancreatic Cancer Tissue Micro-array (TMA)

IHC was used to test the specificity of the antibodies using NCI60 protein array as well as a pancreatic cancer tissue micro array (TMA). Tissues were sectioned into 4µm sections and adhered to positively charged glass microscope slides. Slides were deparaffinized using the Leica Autostainer model XL (Leica) following the protocol detailed in the antibodies.cancer.gov SOP M-106. A 1x Antigen Retrieval buffer (Dako) was prepared and slides were placed into the buffer and placed in a pressure cooker at 123°C for 30 minutes. When the pressure cooker was finished, the slides were removed and allowed to cool to room temperature. Slides were then loaded on to the Dako Autostainer Model LV-1 (Agilent Technologies) and rinsed with IHC wash buffer (Dako). The slides were incubated in a hydrogen peroxide block (Cell Marque) for 10 mins at room temperature. The slides were then rinsed with IHC wash buffer. The slides were then incubated with a protein block (Dako) for 10 minutes at room temperature. Primary antibody was diluted using antibody diluent (Dako). Primary Antibody solution was added to slides and incubated for 1 hour at room temperature. Slides were rinsed with IHC wash buffer. Rabbit HRP secondary antibody (Dako) was added and incubated for 30 minutes at room temperature. Slides were rinsed with IHC wash buffer (Dako). The DAB substrate was prepared by mixing 1mL of substrate buffer (Cell Marque) with 1 drop of DAB chromogen (Cell Marque). DAB solution was added to the slides and incubated for 10 minutes at room temperature. The slides were rinsed with IHC wash buffer. The slides were then removed from Autostainer and placed back on the Leica Autostainer model XL for the hematoxylin counterstain. Slides were removed from Leica Autostainer and a coverslip was applied using cytoseal XYL mounting medium (Richard Allen Scientific).

Immunohistochemistry NCI60 Protein Array

IHC was performed on the NCI60 protein array using the NCI56 antibodies that were selected by the pathologist. IHC staining protocol above was used on the NCI60 protein array using the optimal conditions selected by the pathologist. Once staining was completed slides were scanned using a Hamamatsu NanoZoomer-XR digital slide scanner (Hamamatsu). Image analysis was completed to determine the staining intensity of each core on the array. Staining intensity ranged from 0-3, 0 being negative or no stain and 3 being the most intense.

C. Tables

Table 1: Western blot (WB) against recombinant protein screening results (Part A)

CPTC Antibody name	Gene	Sequence	Modification site	Antigen Vendor	Antigen Cat. #	WB results
CPTC-ACT Group-1	ACT	AVFPSIVGRPR		Origene	TP303643	Negative
CPTC-CASP3-1	CASP3	IIHGSES(ph)MDSGISLDNSYK	S26	Origene	TP304444	Negative
CPTC-CDC25B-1	CDC25B	LLGHS(ph)PVLR	S160	Origene	LY402883	Positive
CPTC-CDC25B-2	CDC25B	SPS(ph)MPC(Cam)SVIRPILK	S323	Origene	LY402883	Negative
CPTC-CDC25C-1	CDC25C	SPS(ph)MPENLNRPR	S216	Origene	TP305641	Negative
CPTC-CDCA8-1	CDCA8	LTAEAIQT(ph)PLK	T106	Origene	TP760482	Negative
CPTC-CDK1-1	CDK1	VYT(ph)HEVVTLWYR	T161	Origene	TP300495	Positive
CPTC-CDK7-1	CDK7	AYT(ph)HQVVTR	T170	Origene	TP302736	Positive
CPTC-CHEK1-6	CHEK1	VTSGGVSES(ph)PSGFSK	S286	Origene	TP325807	Positive
CPTC-CHEK2-1	CHEK2	ILGETS(ph)LM(ox)R	S379	Origene	TP301278	Negative
CPTC-CHEK2-2	CHEK2	TLC(Cam)GT(ph)PTYLAPEVLV SVGTAGYNR	T387	Origene	TP301278	Positive
CPTC-GAPDH-1	GAPDH	GALQNIIPASTGAAK		Origene	TP302309	Positive
CPTC-KNL1-1	CASC5	SLS(ph)NPTPDYC(Cam)HDK	S767	Novus Biologicals	H00057082-Q01-25ug	Negative

Table 1: Western blot (WB) against recombinant protein screening results (Part B)

CPTC Antibody name	Gene	Sequence	Modification site	Antigen Vendor	Antigen Cat. #	WB results
CPTC-KRT Group A-1	KRTA	LAADDFR		Origene	TP309707	Positive
CPTC-KRT Group B-1	KRTB	ELQSQISDTSVVLSMDNSR		Origene	TP309570	Positive
CPTC-LAT-1	LAT	EYVNVS(ph)QELHPGAAK	S224	Novus Biologicals	NBP1-72479-50ug	Positive
CPTC-LIME1-1	LIME1	SSTC(Cam)GAGT(ph)PPASSC(Cam)PSLGR	T274	Origene	LY413516	Negative
CPTC-LMNB1-1	LMNB1	AGGPTT(ph)PLS(ph)PTR	S23;T20	Origene	TP301604	Negative
CPTC-LMNB1-2	LMNB1	AGGPTT(ph)PLS(ph)PTR	S23;T20	Origene	TP301604	Positive
CPTC-MCM6-1	MCM6	EIESEIDS(ph)EEELINK	S762	Origene	TP307097	Negative
CPTC-MDM2-1	MDM2	AIS(ph)ETEENSDELSGER	S166	Origene	TP761916	Positive
CPTC-MKI67-3	MKI67	DINTFLGT(ph)PVQK	T1801	Origene	TP710117	Negative
CPTC-MKI67-4	MKI67	NINTFVET(ph)PVQK	T2406	Origene	TP710117	Negative
CPTC-MRE11A-1	MRE11A	IMS(ph)QSQVSK	S676	Origene	TP309414	Negative
CPTC-NCAPH2-1	NCAPH2	FVQETELS(ph)QR	S492	Origene	TP317604	Negative

Table 1: Western blot (WB) against recombinant protein screening results (Part C)

CPTC Antibody name	Gene	Sequence	Modification site	Antigen Vendor	Antigen Cat. #	WB results
CPTC-NUMA1-1	NUMA1	LSQLEEHLS(ph)QLQDNPPQEK	S395	Origene	LY416823	Negative
CPTC-PAK4-1	PAK4	RPLS(ph)GPDVGTPQPAGLASG AK	S181	Origene	TP302302	Positive
CPTC-PARP1-2	PARP1	MAIMVQS(ph)PMFDGK	S41	Origene	TP307085	Negative
CPTC-RAD50-2	RAD50	YELQQLEGS(ph)SDR	S470	Novus Biologicals	NBL1-15116	Negative
CPTC-RAD9A-1	RAD9A	SPQGPSPVLAEDS(ph)EGEG	S387	Origene	TP304439	Negative
CPTC-RTF1-1	RTF1	SASDLS(ph)EDLFK	S655	Origene	LY432360	Negative
CPTC-RTF1-2	RTF1	SASDLS(ph)EDLFK	S655	Origene	LY432360	Negative
CPTC-SAAL1-1	SAAL1	NGAAQPLDQPQEES(ph)EEQP VFR	S237	Origene	TP304551	Positive
CPTC-TNFRSF17-1	TNFRSF17	SLPAALS(ph)ATEIEK	S173	Origene	TP723029	Negative
CPTC-TP53-1	TP53	ALPNNTSSS(ph)PQPK	pS315	Origene	TP300003	Positive
CPTC-TP53-2	TP53	MEEPQSDPSVEPPLS(ph)QETF SDLWK	pS15	Origene	TP300003	Positive
CPTC-TP53BP1-1	TP53BP1	IDEDGENT(ph)QIEDTEPMS(p h)PVLNSK	T543	Origene	TP318016	Positive
CPTC-TUBB-1	TUBB	ISVYYNEATGGK		Origene	TP303629	Negative
CPTC-UTP14A-1	UTP14A	DSGS(ph)QEVLSELR	S453	Origene	TP710267	Negative

Table 2: Western blot (WB) against cell lysates screening results

**CPTC Antibody name**	**LCL57**	**HeLa**	**MCF10A**
CPTC-CDC25B-1	Positive	Positive	Positive
CPTC-CDK1-1	Positive	Positive	Positive
CPTC-CDK7-1	Positive	Positive	Positive
CPTC-CHEK1-6	Positive	Positive	Positive
CPTC-CHEK2-2	Uncertain	Uncertain	Uncertain
CPTC-GAPDH-1	Positive	Positive	Positive
CPTC-KRT Group A-1	Negative	Positive	Positive
CPTC-KRT Group B-1	Negative	Positive	Positive
CPTC-LAT-1	Positive	Positive	Positive
CPTC-LMNB1-2	Positive	Positive	Positive
CPTC-MDM2-1	Positive	Positive	Positive
CPTC-PAK4-1	Negative	Negative	Negative
CPTC-SAAL1-1	Positive	Negative	Negative
CPTC-TP53-1	Uncertain	Uncertain	Uncertain
CPTC-TP53-2	Positive	Positive	Positive
CPTC-TP53BP1-1	Uncertain	Uncertain	Uncertain

Table 3: Simple Western (Wes) against cell lysates screening results

**CPTC Antibody name**	**LCL57**	**HeLa**	**MCF10A**
CPTC-CDC25B-1	Negative	Negative	Negative
CPTC-CDK1-1	Positive	Positive	Positive
CPTC-CDK7-1	Positive	Positive	Positive
CPTC-CHEK1-6	Negative	Negative	Positive
CPTC-CHEK2-2	Positive	Positive	Positive
CPTC-GAPDH-1	Positive	Positive	Positive
CPTC-KRT Group A-1	*not tested*	*not tested*	*not tested*
CPTC-KRT Group B-1	Negative	Positive	Positive
CPTC-LAT-1	Negative	Negative	Negative
CPTC-LMNB1-2	Negative	Negative	Negative
CPTC-MDM2-1	Uncertain	Uncertain	Uncertain
CPTC-PAK4-1	Positive	Positive	Positive
CPTC-SAAL1-1	Positive	Uncertain	Positive
CPTC-TP53-1	Negative	Positive	Negative
CPTC-TP53-2	Positive	Positive	Positive
CPTC-TP53BP1-1	*not tested*	*not tested*	*not tested*

Table 4: Phospho-specificity experiment – Simple Western (Wes) against cell lysates screening results

CPTC Antibody name	LCL57	HeLa	MCF10A
CPTC-CASP3-1	Negative	Negative	Negative
CPTC-CDC25B-2	Negative	Negative	Negative
CPTC-CDC25C-1	Negative	Negative	Negative
CPTC-CDCA8-1	Negative	Negative	Negative
CPTC-CHEK2-1	Negative	Negative	Negative
CPTC-KNL1-1	Negative	Negative	Negative
CPTC-LIME1-1	Negative	Negative	Negative
CPTC-LMNB1-1	Negative	Negative	Negative
CPTC-MCM6-1	Negative	Negative	Negative
CPTC-MKI67-3	Negative	Uncertain	Uncertain
CPTC-MKI67-4	Negative	Negative	Negative
CPTC-MRE11A-1	Negative	Negative	Negative
CPTC-NCAPH2-1	Negative	Negative	Negative
CPTC-NUMA1-1	Positive	Positive	Positive
CPTC-PARP1-2	Negative	Negative	Negative
CPTC-RAD50-2	Negative	Negative	Negative
CPTC-RAD9A-1	Negative	Positive	Negative
CPTC-RTF1-1	Negative	Negative	Negative
CPTC-RTF1-2	Negative	Negative	Negative
CPTC-TNFRSF17-1	Negative	Negative	Uncertain
CPTC-UTP14A-1	Negative	Negative	Uncertain

Table 5: Phospho-specificity experiment – Western Blot (WB) against cell lysates confirmation results

CPTC Antibody name	LCL57	HeLa	MCF10A
CPTC-MKI67-3	Negative	Negative	Negative
CPTC-NUMA1-1	Positive	Uncertain	Uncertain
CPTC-RAD9A-1	Uncertain	Uncertain	Negative
CPTC-TNFRSF17-1	Negative	Negative	Negative
CPTC-UTP14A-1	Negative	Negative	Negative

Table 6: Immuno-Fluorescence (IF) in cell lines screening results

CPTC Antibody name	LCL57	HeLa	MCF10A
CPTC-CDC25B-1	Positive	Negative	Negative
CPTC-CDK1-1	Positive	Positive	Positive
CPTC-CDK7-1	Positive	Positive	Negative
CPTC-CHEK1-6	Negative	Negative	Positive
CPTC-CHEK2-2	Positive	Negative	Negative
CPTC-GAPDH-1	Positive	Positive	Positive
CPTC-KRT Group A-1	Positive	Positive	Positive
CPTC-KRT Group B-1	Negative	Positive	Positive
CPTC-LAT-1	Negative	Negative	Negative
CPTC-LMNB1-2	Positive	Positive	Positive
CPTC-MDM2-1	Positive	Positive	Positive
CPTC-PAK4-1	Positive	Negative	Negative
CPTC-SAAL1-1	Positive	Negative	Negative
CPTC-TP53-1	Positive	Positive	Positive
CPTC-TP53-2	Positive	Positive	Positive
CPTC-TP53BP1-1	Positive	Positive	Positive

Table 7: Single Cell Western blot (SCWB) in cell lines screening results

CPTC Antibody name	LCL57	HeLa	MCF10A
CPTC-CDC25B-1	Negative	Negative	Negative
CPTC-CDK1-1	Positive	Positive	Positive
CPTC-CDK7-1	Positive	Positive	Positive
CPTC-CHEK1-6	Negative	Negative	Negative
CPTC-CHEK2-2	Negative	Negative	Negative
CPTC-GAPDH-1	*not tested*	*not tested*	*not tested*
CPTC-KRT Group A-1	Negative	Positive	Positive
CPTC-KRT Group B-1	Negative	Positive	Positive
CPTC-LAT-1	Negative	Negative	Negative
CPTC-LMNB1-2	Positive	Positive	Positive
CPTC-MDM2-1	Positive	Positive	Positive
CPTC-PAK4-1	Negative	Negative	Negative
CPTC-SAAL1-1	Negative	Negative	Negative
CPTC-TP53-1	Negative	Negative	Negative
CPTC-TP53-2	Positive	Positive	Positive
CPTC-TP53BP1-1	*not tested*	*not tested*	*not tested*

Table 8: Immuno-precipitation (IP) with recombinant protein and cell lysates screening results

CPTC Antibody name	Recombinant Protein	LCL57	HeLa	MCF10A
CPTC-CDC25B-1	*not tested*	Negative	Negative	Negative
CPTC-CDK1-1	Weak	Negative	Negative	Negative
CPTC-CDK7-1	Weak	Negative	Negative	Negative
CPTC-CHEK1-6	Weak	Negative	Negative	Negative
CPTC-CHEK2-2	Positive	Negative	Negative	Negative
CPTC-GAPDH-1	Positive	Negative	Weak	Negative
CPTC-KRT Group A-1	*not tested*	Weak	Weak	Negative
CPTC-KRT Group B-1	Positive	Negative	Positive	Negative
CPTC-LAT-1	Positive	Negative	Negative	Negative
CPTC-LMNB1-2	Positive	Negative	Negative	Negative
CPTC-MDM2-1	*not tested*	Negative	Negative	Negative
CPTC-PAK4-1	Positive	Negative	Negative	Negative
CPTC-SAAL1-1	Positive	Negative	Negative	Negative
CPTC-TP53-1	Weak	Negative	Negative	Negative
CPTC-TP53-2	Positive	Negative	Negative	Negative
CPTC-TP53BP1-1	*not tested*	Positive	Weak	Negative

ID	Antigen Accession #	CPTC Ab name	Coverage	# Proteins	# Unique Peptides	# Peptides	# PSMs	D peptide	D PSMs	PSM ratio (IP/Neg Ctrl)
Negative Control	P06493	CPTC-CDK1-1	**53.20**	28	13	**13**	**16**	2	3	**1.2**
IP	P06493	CPTC-CDK1-1	**50.17**	27	14	**15**	**19**			
Negative Control	P50613	CPTC-CDK7-1	**42.20**	3	19	**19**	**28**	3	10	**1.4**
IP	P50613	CPTC-CDK7-1	**52.31**	3	22	**22**	**38**			
Negative Control	O14757	CPTC-CHEK1-6	**35.29**	1	15	**15**	**16**	2	3	**1.2**
IP	O14757	CPTC-CHEK1-6	**36.55**	1	17	**17**	**19**			
Negative Control	O96017	CPTC-CHEK2-2	**39.78**	1	16	**16**	**17**	11	49	**3.9**
IP	O96017	CPTC-CHEK2-2	**50.28**	1	27	**27**	**66**			
Negative Control	P04406	CPTC-GAPDH-1	**22.39**	2	6	**6**	**10**	13	49	**5.9**
IP	P04406	CPTC-GAPDH-1	**66.87**	2	19	**19**	**59**			
Negative Control	P05787	CPTC-KRT Group B-1	**20.50**	14	3	**7**	**9**	42	109	**13.1**
IP	P05787	CPTC-KRT Group B-1	**71.43**	16	41	**49**	**118**			
Negative Control	O43561	CPTC-LAT-1	**6.11**	1	1	**1**	**4**	5	6	**2.5**
IP	O43561	CPTC-LAT-1	**50.00**	1	6	**6**	**10**			
Negative Control	P20700	CPTC-LMNB1-2	**16.89**	1	9	**9**	**10**	10	12	**2.2**
IP	P20700	CPTC-LMNB1-2	**30.20**	1	19	**19**	**22**			
Negative Control	O96013	CPTC-PAK4-1	**10.49**	3	5	**5**	**6**	10	16	**3.7**
IP	O96013	CPTC-PAK4-1	**32.49**	3	15	**15**	**22**			
Negative Control	Q96ER3	CPTC-SAAL1-1	**5.91**	1	2	**2**	**2**	15	31	**16.5**
IP	Q96ER3	CPTC-SAAL1-1	**37.97**	1	17	**17**	**33**			
Negative Control	P04637	CPTC-TP53-1	**41.22**	1	16	**16**	**31**	5	16	**1.5**
IP	P04637	CPTC-TP53-1	**49.36**	1	21	**21**	**47**			
Negative Control	P04637	CPTC-TP53-2	**47.58**	1	18	**18**	**30**	5	39	**2.3**
IP	P04637	CPTC-TP53-2	**49.11**	1	23	**23**	**69**			

Table 9: Immuno-precipitation (IP) with recombinant protein screening results – details

Note: PSM ratio (IP/negative Control) below 1 has been indicated as negative; values of PMS ratio between 1 and 2 are considered weak; PSM ratio values above 2 have been assigned as positive

ID	Antigen Accession #	CPTC Ab name	Coverage	# Proteins	# Unique Peptides	# Peptides	# PSMs	D peptide	D PSMs	PSM ratio (IP/Neg Ctrl)
Negative Control	P30305	CPTC-CDC25B-1	**0.00**	0	0	**0**	**0**	0	0	**N/A**
IP	P30305	CPTC-CDC25B-1	**0.00**	0	0	**0**	**0**			
Negative Control	P06493	CPTC-CDK1-1	**0.00**	0	0	**0**	**0**	0	0	**N/A**
IP	P06493	CPTC-CDK1-1	**0.00**	0	0	**0**	**0**			
Negative Control	P50613	CPTC-CDK7-1	**0.00**	0	0	**0**	**0**	0	0	**N/A**
IP	P50613	CPTC-CDK7-1	**0.00**	0	0	**0**	**0**			
Negative Control	O14757	CPTC-CHEK1-6	**0.00**	0	0	**0**	**0**	0	0	**N/A**
IP	O14757	CPTC-CHEK1-6	**0.00**	0	0	**0**	**0**			
Negative Control	O96017	CPTC-CHEK2-2	**0.00**	0	0	**0**	**0**	0	0	**N/A**
IP	O96017	CPTC-CHEK2-2	**0.00**	0	0	**0**	**0**			
Negative Control	P04406	CPTC-GAPDH-1	**6.57**	2	2	**2**	**2**	-1	-1	**0.5**
IP	P04406	CPTC-GAPDH-1	**4.48**	1	1	**1**	**1**			
Negative Control	P04264	CPTC-KRT Group A-1	**44.41**	2	23	**27**	**32**	7	12	**1.4**
IP	P04264	CPTC-KRT Group A-1	**50.00**	3	31	**34**	**44**			
Negative Control	P05787	CPTC-KRT Group B-1	**0.00**	0	0	**0**	**0**	0	0	**N/A**
IP	P05787	CPTC-KRT Group B-1	**0.00**	0	0	**0**	**0**			
Negative Control	O43561	CPTC-LAT-1	**0.00**	0	0	**0**	**0**	0	0	**N/A**
IP	O43561	CPTC-LAT-1	**0.00**	0	0	**0**	**0**			
Negative Control	P20700	CPTC-LMNB1-2	**0.00**	0	0	**0**	**0**	1	1	**N/A**
IP	P20700	CPTC-LMNB1-2	**2.05**	1	1	**1**	**1**			
Negative Control	Q00987	CPTC-MDM2-1	**0.00**	0	0	**0**	**0**	0	0	**N/A**
IP	Q00987	CPTC-MDM2-1	**0.00**	0	0	**0**	**0**			
Negative Control	O96013	CPTC-PAK4-1	**0.00**	0	0	**0**	**0**	0	0	**N/A**
IP	O96013	CPTC-PAK4-1	**0.00**	0	0	**0**	**0**			
Negative Control	Q96ER3	CPTC-SAAL1-1	**0.00**	0	0	**0**	**0**	0	0	**N/A**
IP	Q96ER3	CPTC-SAAL1-1	**0.00**	0	0	**0**	**0**			
Negative Control	P04637	CPTC-TP53-1	**0.00**	0	0	**0**	**0**	0	0	**N/A**
IP	P04637	CPTC-TP53-1	**0.00**	0	0	**0**	**0**			
Negative Control	P04637	CPTC-TP53-2	**0.00**	0	0	**0**	**0**	0	0	**N/A**
IP	P04637	CPTC-TP53-2	**0.00**	0	0	**0**	**0**			
Negative Control	Q12888	CPTC-TP53BP1-1	**0.66**	1	1	**1**	**1**	26	31	**32.0**
IP	Q12888	CPTC-TP53BP1-1	**19.32**	1	27	**27**	**32**			

Table 10: Immuno-precipitation (IP) with LCL57 cell lysates screening results – details

Note: PSM ratio (IP/negative Control) below 1 has been indicated as negative; values of PMS ratio between 1 and 2 are considered weak; PSM ratio values above 2 have been assigned as positive

ID	Antigen Accession #	CPTC Ab name	Coverage	# Proteins	# Unique Peptides	# Peptides	# PSMs	D peptide	D PSMs	PSM ratio (IP/Neg Ctrl)
Negative Control	P30305	CPTC-CDC25B-1	**0.00**	0	0	**0**	**0**	0	0	**N/A**
IP	P30305	CPTC-CDC25B-1	**0.00**	0	0	**0**	**0**			
Negative Control	P06493	CPTC-CDK1-1	**3.37**	1	1	**1**	**1**	-1	-1	**0.0**
IP	P06493	CPTC-CDK1-1	**0.00**	0	0	**0**	**0**			
Negative Control	P50613	CPTC-CDK7-1	**0.00**	0	0	**0**	**0**	0	0	**N/A**
IP	P50613	CPTC-CDK7-1	**0.00**	0	0	**0**	**0**			
Negative Control	O14757	CPTC-CHEK1-6	**0.00**	0	0	**0**	**0**	0	0	**N/A**
IP	O14757	CPTC-CHEK1-6	**0.00**	0	0	**0**	**0**			
Negative Control	O96017	CPTC-CHEK2-2	**0.00**	0	0	**0**	**0**	0	0	**N/A**
IP	O96017	CPTC-CHEK2-2	**0.00**	0	0	**0**	**0**			
Negative Control	P04406	CPTC-GAPDH-1	**13.73**	1	4	**4**	**4**	2	3	**1.8**
IP	P04406	CPTC-GAPDH-1	**22.69**	1	6	**6**	**7**			
Negative Control	P04264	CPTC-KRT Group A-1	**37.27**	3	19	**22**	**25**	3	10	**1.4**
IP	P04264	CPTC-KRT Group A-1	**43.17**	3	21	**25**	**35**			
Negative Control	P05787	CPTC-KRT Group B-1	**9.32**	11	1	**5**	**6**	6	6	**2.0**
IP	P05787	CPTC-KRT Group B-1	**21.33**	8	5	**11**	**12**			
Negative Control	O43561	CPTC-LAT-1	**0.00**	0	0	**0**	**0**	0	0	**N/A**
IP	O43561	CPTC-LAT-1	**0.00**	0	0	**0**	**0**			
Negative Control	P20700	CPTC-LMNB1-2	**0.00**	0	0	**0**	**0**	1	1	**N/A**
IP	P20700	CPTC-LMNB1-2	**4.10**	1	1	**1**	**1**			
Negative Control	Q00987	CPTC-MDM2-1	**0.00**	0	0	**0**	**0**	0	0	**N/A**
IP	Q00987	CPTC-MDM2-1	**0.00**	0	0	**0**	**0**			
Negative Control	O96013	CPTC-PAK4-1	**0.00**	0	0	**0**	**0**	1	1	**N/A**
IP	O96013	CPTC-PAK4-1	**3.05**	1	1	**1**	**1**			
Negative Control	Q96ER3	CPTC-SAAL1-1	**0.00**	0	0	**0**	**0**	0	0	**N/A**
IP	Q96ER3	CPTC-SAAL1-1	**0.00**	0	0	**0**	**0**			
Negative Control	P04637	CPTC-TP53-1	**0.00**	0	0	**0**	**0**	0	0	**N/A**
IP	P04637	CPTC-TP53-1	**0.00**	0	0	**0**	**0**			
Negative Control	P04637	CPTC-TP53-2	**0.00**	0	0	**0**	**0**	0	0	**N/A**
IP	P04637	CPTC-TP53-2	**0.00**	0	0	**0**	**0**			
Negative Control	Q12888	CPTC-TP53BP1-1	**8.27**	1	11	**11**	**12**	1	1	**1.1**
IP	Q12888	CPTC-TP53BP1-1	**9.58**	1	12	**12**	**13**			

Table 11: Immuno-precipitation (IP) with HeLa cell lysates screening results – details

Note: PSM ratio (IP/negative Control) below 1 has been indicated as negative; values of PMS ratio between 1 and 2 are considered weak; PSM ratio values above 2 have been assigned as positive

ID	Antigen Accession #	CPTC Ab ID	Coverage	# Proteins	# Unique Peptides	# Peptides	# PSMs	D peptide	D PSMs	PSM ratio (IP/Neg Ctrl)
Negative Control	P30305	CPTC-CDC25B-1	**0.00**	0	0	**0**	**0**	0	0	**N/A**
IP	P30305	CPTC-CDC25B-1	**0.00**	0	0	**0**	**0**			
Negative Control	P06493	CPTC-CDK1-1	**0.00**	0	0	**0**	**0**	0	0	**N/A**
IP	P06493	CPTC-CDK1-1	**0.00**	0	0	**0**	**0**			
Negative Control	P50613	CPTC-CDK7-1	**0.00**	0	0	**0**	**0**	0	0	**N/A**
IP	P50613	CPTC-CDK7-1	**0.00**	0	0	**0**	**0**			
Negative Control	O14757	CPTC-CHEK1-6	**0.00**	0	0	**0**	**0**	0	0	**N/A**
IP	O14757	CPTC-CHEK1-6	**0.00**	0	0	**0**	**0**			
Negative Control	O96017	CPTC-CHEK2-2	**0.00**	0	0	**0**	**0**	0	0	**N/A**
IP	O96017	CPTC-CHEK2-2	**0.00**	0	0	**0**	**0**			
Negative Control	P04406	CPTC-GAPDH-1	**7.46**	2	3	**3**	**3**	-1	-1	**0.7**
IP	P04406	CPTC-GAPDH-1	**6.57**	2	2	**2**	**2**			
Negative Control	P04264	CPTC-KRT Group A-1	**40.68**	1	25	**30**	**34**	-3	-3	**0.9**
IP	P04264	CPTC-KRT Group A-1	**46.27**	1	23	**27**	**31**			
Negative Control	P05787	CPTC-KRT Group B-1	**61.90**	11	34	**40**	**60**	-5	-16	**0.7**
IP	P05787	CPTC-KRT Group B-1	**62.11**	11	29	**35**	**44**			
Negative Control	O43561	CPTC-LAT-1	**0.00**	0	0	**0**	**0**	0	0	**N/A**
IP	O43561	CPTC-LAT-1	**0.00**	0	0	**0**	**0**			
Negative Control	P20700	CPTC-LMNB1-2	**0.00**	0	0	**0**	**0**	0	0	**N/A**
IP	P20700	CPTC-LMNB1-2	**0.00**	0	0	**0**	**0**			
Negative Control	Q00987	CPTC-MDM2-1	**0.00**	0	0	**0**	**0**	0	0	**N/A**
IP	Q00987	CPTC-MDM2-1	**0.00**	0	0	**0**	**0**			
Negative Control	O96013	CPTC-PAK4-1	**0.00**	0	0	**0**	**0**	0	0	**N/A**
IP	O96013	CPTC-PAK4-1	**0.00**	0	0	**0**	**0**			
Negative Control	Q96ER3	CPTC-SAAL1-1	**0.00**	0	0	**0**	**0**	0	0	**N/A**
IP	Q96ER3	CPTC-SAAL1-1	**0.00**	0	0	**0**	**0**			
Negative Control	P04637	CPTC-TP53-1	**0.00**	0	0	**0**	**0**	0	0	**N/A**
IP	P04637	CPTC-TP53-1	**0.00**	0	0	**0**	**0**			
Negative Control	P04637	CPTC-TP53-2	**0.00**	0	0	**0**	**0**	0	0	**N/A**
IP	P04637	CPTC-TP53-2	**0.00**	0	0	**0**	**0**			
Negative Control	Q12888	CPTC-TP53BP1-1	**0.00**	0	0	**0**	**0**	0	0	**N/A**
IP	Q12888	CPTC-TP53BP1-1	**0.00**	0	0	**0**	**0**			

Table 12: Immuno-precipitation (IP) with MCF10A cell lysates screening results – details

Note: PSM ratio (IP/negative Control) below 1 has been indicated as negative; values of PMS ratio between 1 and 2 are considered weak; PSM ratio values above 2 have been assigned as positive

CPTC Antibody name	pH		Antibody Dilutions			
	pH 6	pH 9	50	250	1250	6250
CPTC-ACT Group-1	Negative	Negative	Negative	Negative	Negative	Negative
CPTC-CASP3-1	Negative	Negative	Negative	Negative	Negative	Negative
CPTC-CDC25B-1	Negative	Negative	Negative	Negative	Negative	Negative
CPTC-CDC25B-2	Negative	Negative	Negative	Negative	Negative	Negative
CPTC-CDC25C-1	Not optimal	Positive	Positive	Not optimal	Not optimal	Not optimal
CPTC-CDCA8-1	Negative	Negative	Negative	Negative	Negative	Negative
CPTC-CDK1-1	Negative	Negative	Negative	Negative	Negative	Negative
CPTC-CDK7-1	Not optimal	Positive	Positive	Not optimal	Not optimal	Not optimal
CPTC-CHEK1-6	Negative	Negative	Negative	Negative	Negative	Negative
CPTC-CHEK2-1	Negative	Negative	Negative	Negative	Negative	Negative
CPTC-CHEK2-2	Negative	Negative	Negative	Negative	Negative	Negative
CPTC-GAPDH-1	Negative	Negative	Negative	Negative	Negative	Negative
CPTC-KNL1-1	Negative	Negative	Negative	Negative	Negative	Negative

Table 13. Immunohistochemistry screening results Part A.

CPTC Antibody name	pH		Antibody Dilutions			
	pH 6	pH 9	50	250	1250	6250
CPTC-KRT Group A-1	Negative	Negative	Negative	Negative	Negative	Negative
CPTC-KRT Group B-1	Positive	Not optimal	Not optimal	Positive	Not optimal	Not optimal
CPTC-LAT-1	Not optimal	Positive	Not optimal	Not optimal	Not optimal	Positive
CPTC-LIME1-1	Negative	Negative	Negative	Negative	Negative	Negative
CPTC-LMNB1-1	Not optimal	Positive	Positive	Not optimal	Not optimal	Not optimal
CPTC-LMNB1-2	Negative	Negative	Negative	Negative	Negative	Negative
CPTC-MCM6-1	Negative	Negative	Negative	Negative	Negative	Negative
CPTC-MDM2-1	Negative	Negative	Negative	Negative	Negative	Negative
CPTC-MKI67-3	Positive	Not optimal	Not optimal	Not optimal	Not optimal	Positive
CPTC-MKI67-4	Not optimal	Positive	Not optimal	Not optimal	Positive	Not optimal
CPTC-MRE11A-1	Positive	Not optimal	Not optimal	Positive	Not optimal	Not optimal
CPTC-NCAPH2-1	Not optimal	Positive	Positive	Not optimal	Not optimal	Not optimal

Table 14. Immunohistochemistry screening results Part B.

CPTC Antibody name	pH		Antibody Dilutions			
	pH 6	pH 9	50	250	1250	6250
CPTC-NUMA1-1	Positive	Not optimal	Not optimal	Not optimal	Positive	Not optimal
CPTC-PAK4-1	Positive	Not optimal	Positive	Not optimal	Not optimal	Not optimal
CPTC-PARP1-2	Negative	Negative	Negative	Negative	Negative	Negative
CPTC-RAD50-2	Negative	Negative	Negative	Negative	Negative	Negative
CPTC-RAD9A-1	Negative	Negative	Negative	Negative	Negative	Negative
CPTC-RTF1-1	Not optimal	Positive	Not optimal	Not optimal	Positive	Not optimal
CPTC-RTF1-2	Positive	Not optimal	Positive	Not optimal	Not optimal	Not optimal
CPTC-SAAL1-1	Negative	Negative	Negative	Negative	Negative	Negative
CPTCTNFRSF17-1	Negative	Negative	Negative	Negative	Negative	Negative
CPTC-TP53-1	Negative	Negative	Negative	Negative	Negative	Negative
CPTC-TP53-2	Positive	Not optimal	Not optimal	Positive	Not optimal	Not optimal
CPTC-TP53BP1-1	Not optimal	Positive	Not optimal	Not optimal	Positive	Not optimal
CPTC-TUBB-1	Negative	Negative	Negative	Negative	Negative	Negative
CPTC-UTP14A-1	Not optimal	Positive	Not optimal	Not optimal	Not optimal	Positive

Table 15. Immunohistochemistry screening results Part C.

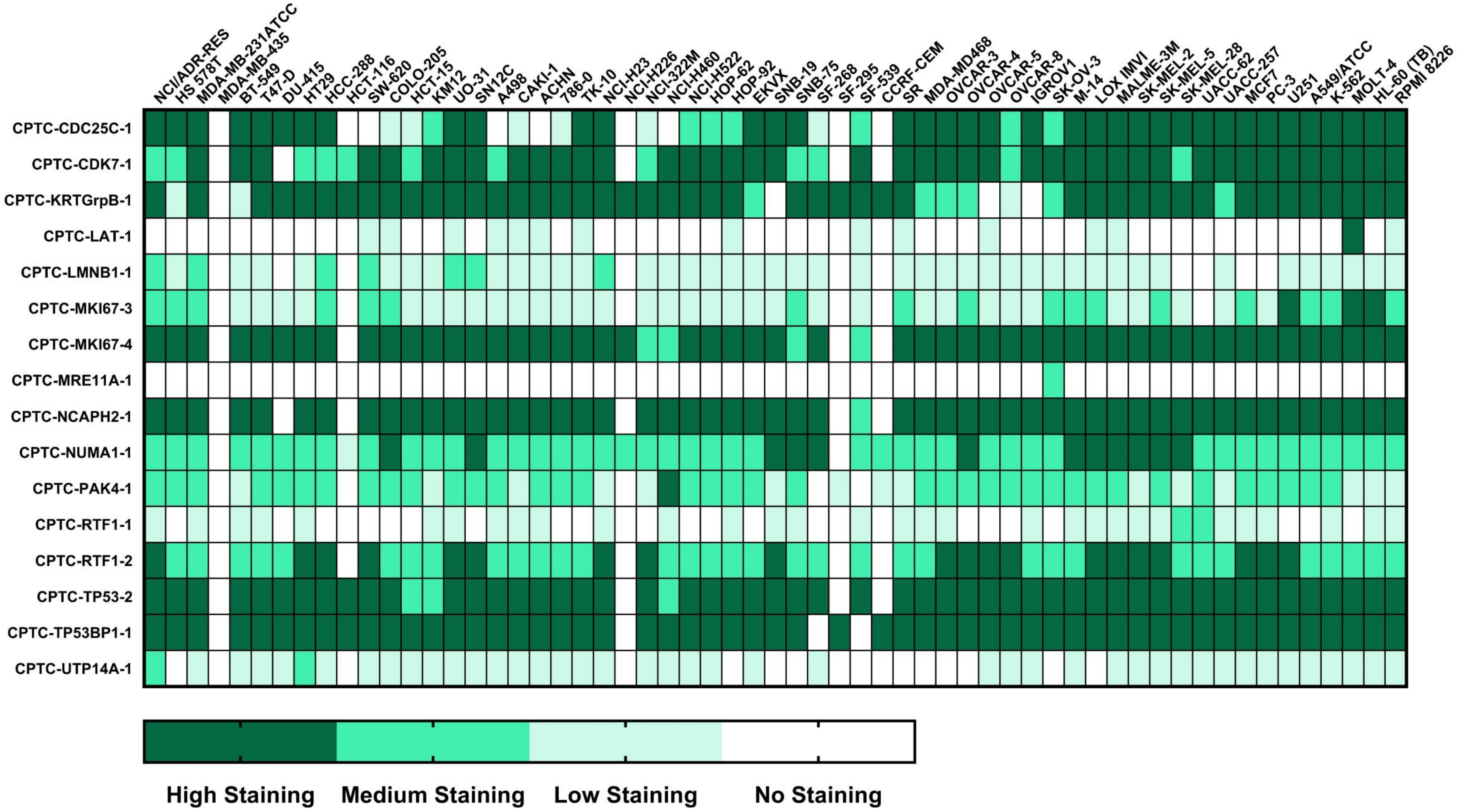

Figure 2. Heatmap showing NCI60 immunohistochemistry staining results.

D. Results

1. CPTC-ACT Group 1……………………………………………………………………….…....Page 29
2. CPTC-CASP3-1……………………………………………………………………………Page 30
3. CPTC-CDC25B-1…………………………………………………………………..…...Page 32
4. CPTC CDC25B-2……………………………………………………………………….....Page 36
5. CPTC-CDC25C-1 ……………………………………………………………………………Page 38
6. CPTC-CDCA8-1 …………………………………………………………………………......Page 41
7. CPTC-CDK1-1 …………………………………………………………………………........Page 43
8. CPTC-CDK7-1 ………………………………………………………………………….. …Page 52
9. CPTC-CHEK1-6 ……………………………………………………………………….... …Page 59
10. CPTC-CHEK2-1 …………………………………………………………….. …….……......Page 63
11. CPTC-CHEK2-2 …………………………………………………………….……….. ...Page 65
12. CPTC-GAPDH-1 …………………………………………………………….………. …Page 69
13. CPTC-KNL1-1 ………………………………………………………………… ……………Page 75
14. CPTC-KRT Group A-1…..…………………………………………………………….......Page 77
15. CPTC-KRT Group B-1…..…………………………………………………………….......Page 84
16. CPTC-LAT-1………………………………………………………………….……….Page 90
17. CPTC-LIME1-1 ………………………………………………………………………….Page 94
18. CPTC-LMNB1-1 ……………………………………………………………...…..……...Page 96
19. CPTC-LMNB1-2 ……………………………………………………………….……...…...Page 99
20. CPTC-MCM6-1 ……………………………………………………………..…………Page 106

D. Results

21. CPTC-MDM2-1……………………………………………………………………..………..…Page 108
22. CPTC-MKI67-3……………………………………………………………………………..…. Page 115
23. CPTC-MKI67-4……………………………………………………………………………...Page 118
24. CPTC-MRE11A-1…………………………………………………………….. ………………Page 121
25. CPTC-NCAPH2-1…………………………………………………………………………..Page 124
26. CPTC-NUMA1-1……………………………………………………………………………….Page 127
27. CPTC-PAK4-1…………………………………………………………………………………….Page 131
28. PTC-PARP1-2…………………………………………………………………………………….Page 136
29. CPTC-RAD50-2 ………………………………………………………………………………….Page 138
30. CPTC-RAD9A-1………………………………………………………………………………….Page 140
31. CPTC-RTF1-1……………………………………………………………………………………..Page 142
32. CPTC-RTF1-2……………………………………………………………………………………..Page 145
33. CPTC-SAAL1-1 ………………………………………………………………………………….Page 148
34. CPTC-TNFRSF17-1 ……………………………………………………………………………….Page 152
35. CPTC-TP53-1 ……………………………………………………………………………………Page 154
36. CPTC-TP53-2 ……………………………………………………………………………………Page 160
37. CPTC-TP53BP1-1 ………………………………………………………………………………Page 168
38. CPTC-TUBB-1 ……………………………………………………………………………………..Page 175
39. CPTC-UTP14A-1 …………………………………………………………………………………..Page 176

Notes

1. The expected molecular weight and subcellular localization data used in this report were derived from The Universal Protein Resource (UniProt) database (uniport.org).

2. The observed molecular weight of the protein may vary from the listed predicted molecular weight due to post translational modifications, post translation cleavages, relative charges, and other experimental factors.

1. CPTC-ACT Group-1

Technique	Analyte	Result
WB	RecProt	**N***
Wes	HeLa	**NT**
	MCF10A	**NT**
	LCL57	**NT**
WB	HeLa	**NT**
	MCF10A	**NT**
	LCL57	**NT**
IF	HeLa	**NT**
	MCF10A	**NT**
	LCL57	**NT**
SCWB	HeLa	**NT**
	MCF10A	**NT**
	LCL57	**NT**
IP	RecProt	**NT**
IP	HeLa	**NT**
	MCF10A	**NT**
	LCL57	**NT**
IHC		**N***
NCI60		**NT**

N – Negative, NT – Not tested, * Data not shown

2. CPTC-CASP3-1

Technique	Analyte	Result
WB	RecProt	**N***
West†	HeLa	**N**
	MCF10A	**N**
	LCL57	**N**
WB	HeLa	**NT**
	MCF10A	**NT**
	LCL57	**NT**
IF	HeLa	**NT**
	MCF10A	**NT**
	LCL57	**NT**
SCWB	HeLa	**NT**
	MCF10A	**NT**
	LCL57	**NT**
IP	RecProt	**NT**
IP	HeLa	**NT**
	MCF10A	**NT**
	LCL57	**NT**
IHC		**N***
NCI60		**NT**

N – Negative, NT – Not Tested, * Data not shown
† Data shown are from the Phospho-specificity Experiment.

Endogenous Wes

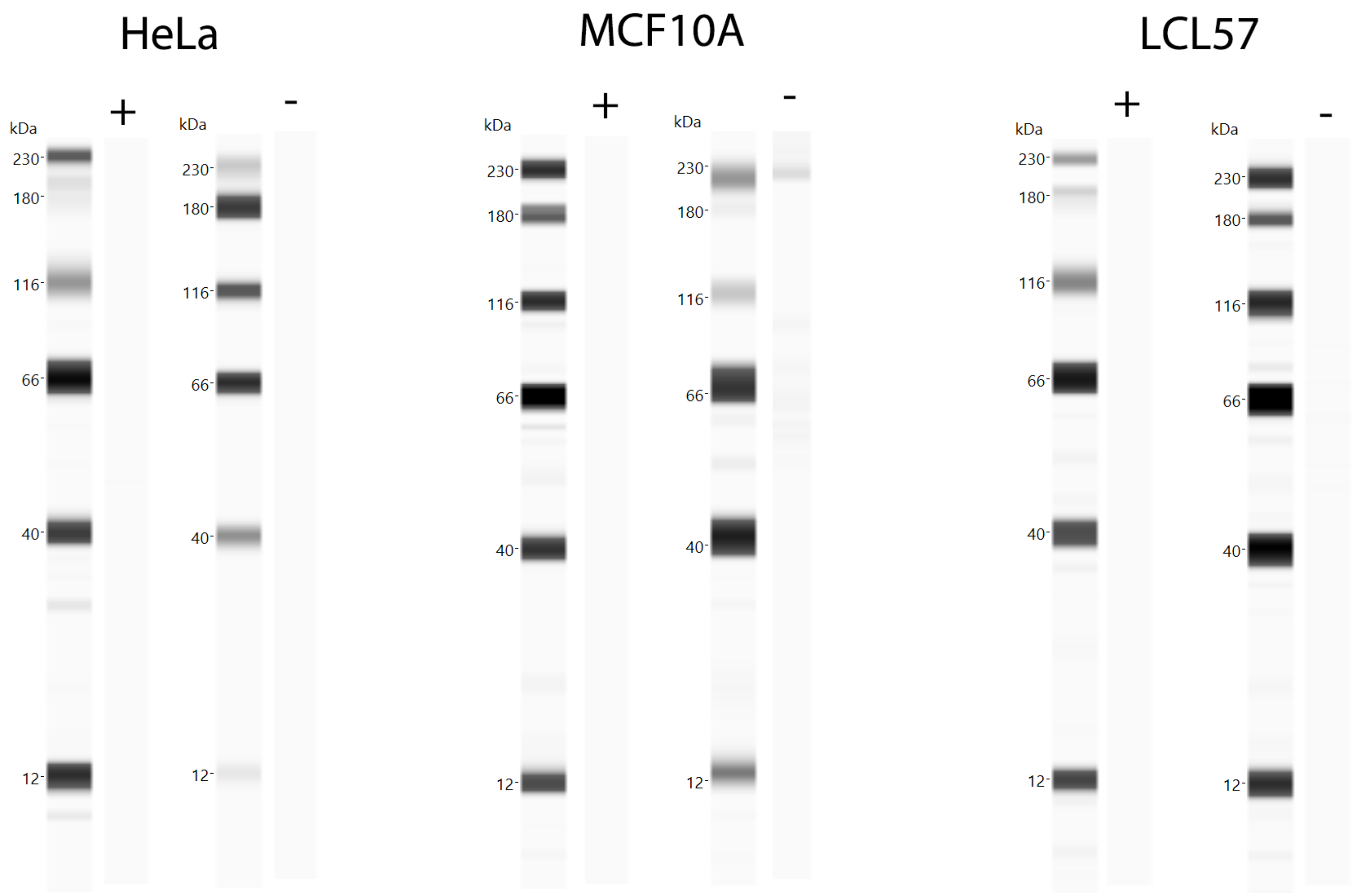

Expected molecular weight: 32 kDa

+ : irradiation treated cells
- : not treated cells

3. CPTC-CDC25B-1

Technique	Analyte	Result
WB	RecProt	**P**
Wes	HeLa	**N**
	MCF10A	**N**
	LCL57	**N**
WB	HeLa	**P**
	MCF10A	**P**
	LCL57	**P**
IF	HeLa	**N†**
	MCF10A	**N†**
	LCL57	**P**
SCWB	HeLa	**N**
	MCF10A	**N**
	LCL57	**N**
IP*	RecProt	**P**
IP*	HeLa	**N**
	MCF10A	**N**
	LCL57	**N**
IHC		**N†**
NCI60		**NT**

P – Positive, N – Negative, * Please refer to tables for results, † Data not shown

WB against commercial recombinant protein

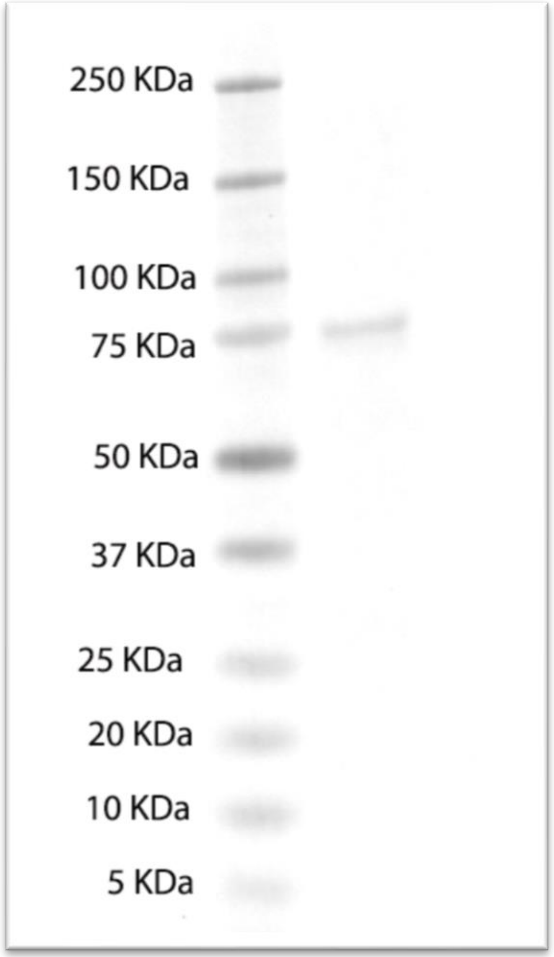

Ag Vendor: Origene

Cat #: LY402883

Description: CDC25B (NM_021873) Human Over-expression Lysate (Transient overexpression lysate of cell division cycle 25 homolog B (S. pombe) (CDC25B), transcript variant 1)

Predicted MW 65 KDa

Endogenous WB

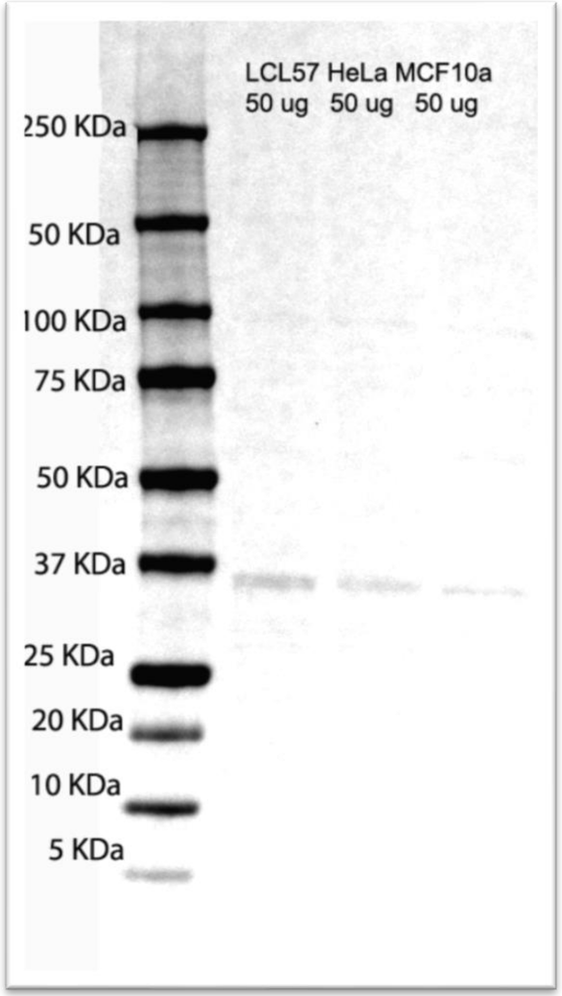

Endogenous Wes

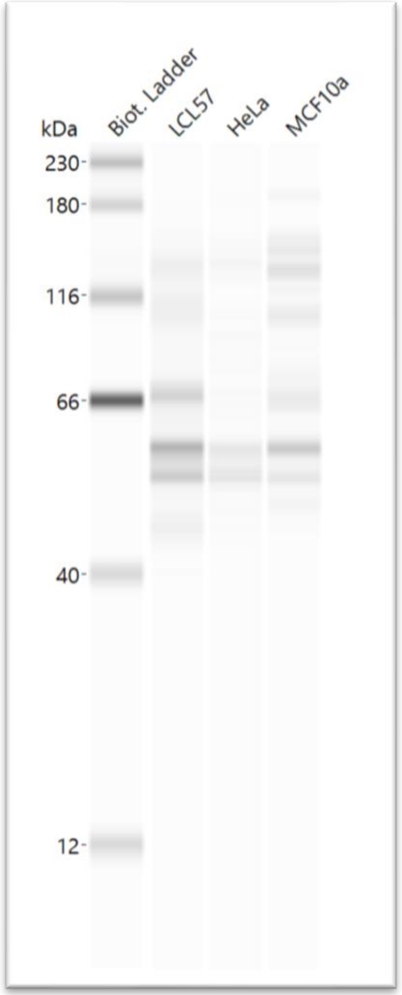

Immunofluorescence – LCL57

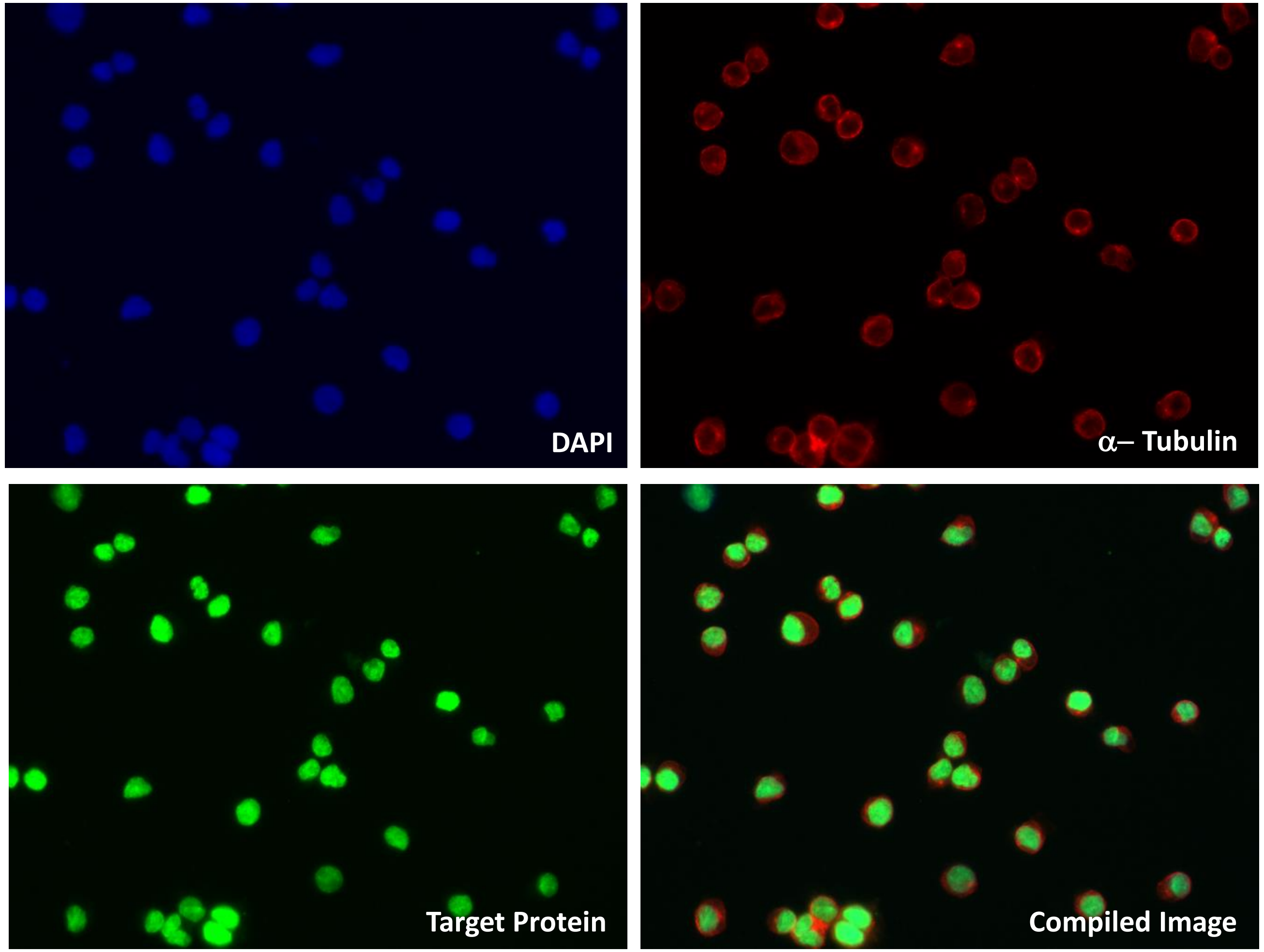

Subcellular location: Cytoskeleton

4. CPTC-CDC25B-2

Technique	Analyte	Result
WB	RecProt	N*
West†	HeLa	N
	MCF10A	N
	LCL57	N
WB	HeLa	NT
	MCF10A	NT
	LCL57	NT
IF	HeLa	NT
	MCF10A	NT
	LCL57	NT
SCWB	HeLa	NT
	MCF10A	NT
	LCL57	NT
IP	RecProt	NT
IP	HeLa	NT
	MCF10A	NT
	LCL57	NT
IHC		N*
NCI60		NT

N – Negative, NT – Not Tested, *Data not shown
†Data shown are from Phospho-specificity Experiment

Endogenous Wes

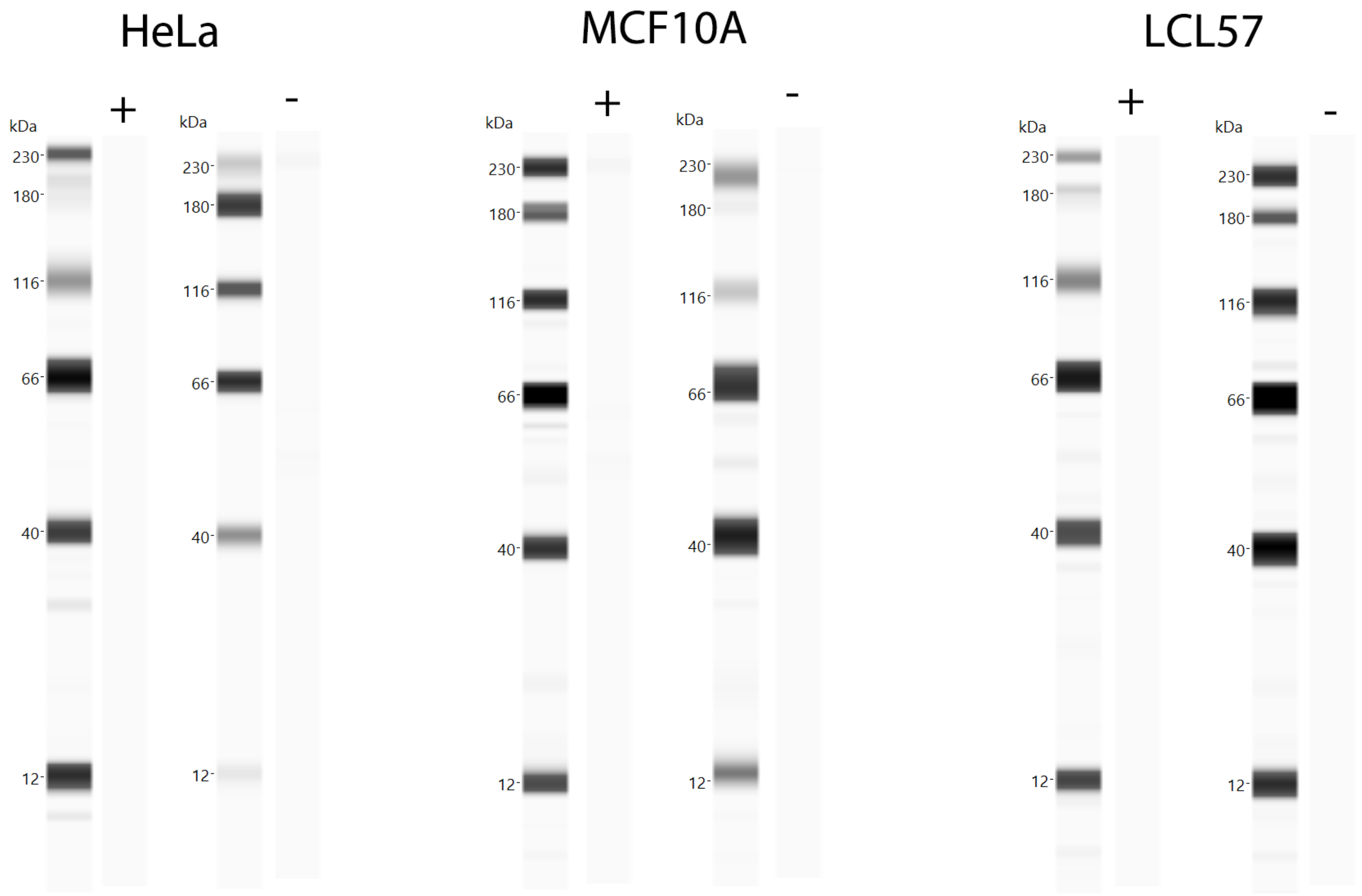

Expected molecular weight: 65, 63, 61, 67 kDa

+ : irradiation treated cells
- : not treated cells

5. CPTC-CDC25C-1

Technique	Analyte	Result
WB	RecProt	N*
West†	HeLa	N
	MCF10A	N
	LCL57	N
WB	HeLa	NT
	MCF10A	NT
	LCL57	NT
IF	HeLa	NT
	MCF10A	NT
	LCL57	NT
SCWB	HeLa	NT
	MCF10A	NT
	LCL57	NT
IP	RecProt	NT
IP	HeLa	NT
	MCF10A	NT
	LCL57	NT
IHC		P
NCI60		P

N – Negative, NT – Not Tested, *Data not shown
†Data shown are from Phospho-specificity Experiment

Endogenous Wes

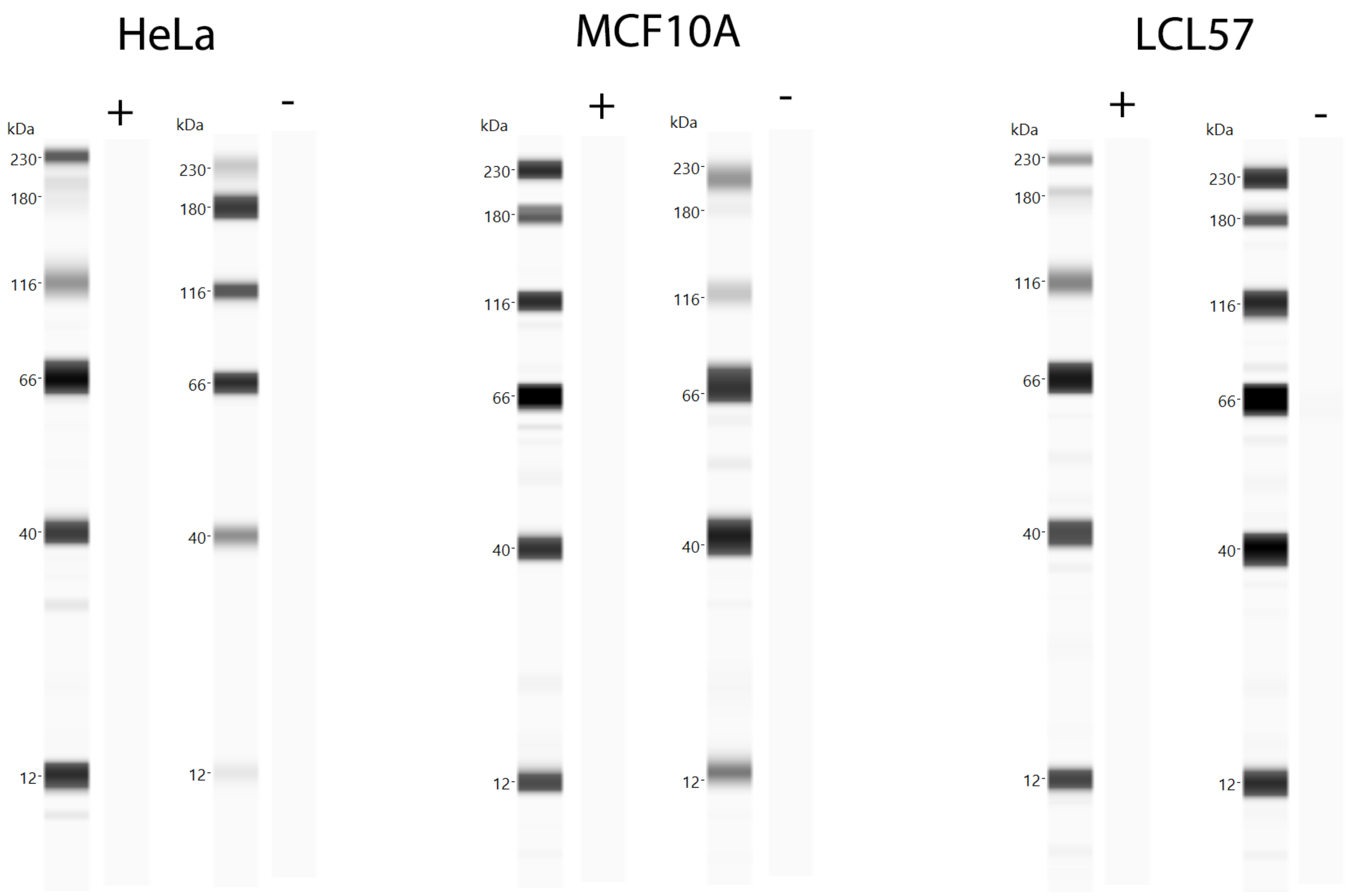

Expected molecular weight: 53, 50, 49, 46 kDa

+ : irradiation treated cells
- : not treated cells

CPTC- CDC25C-1

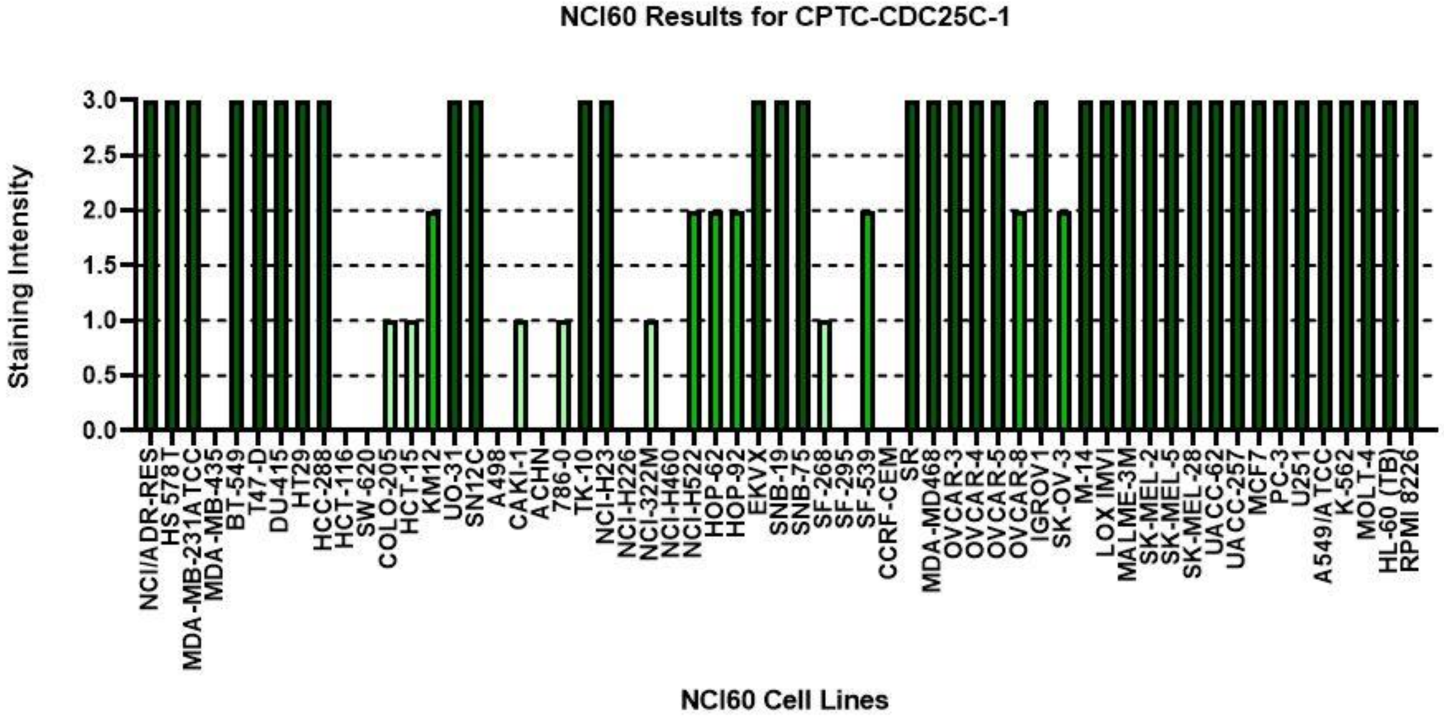

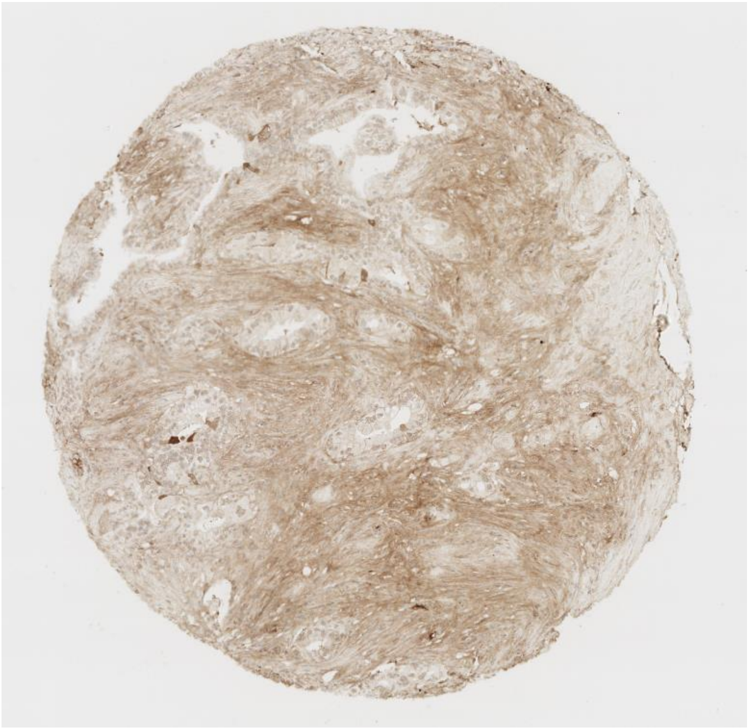

6. CPTC-CDCA8-1

Technique	Analyte	Result
WB	RecProt	N*
West†	HeLa	N
	MCF10A	N
	LCL57	N
WB	HeLa	NT
	MCF10A	NT
	LCL57	NT
IF	HeLa	NT
	MCF10A	NT
	LCL57	NT
SCWB	HeLa	NT
	MCF10A	NT
	LCL57	NT
IP	RecProt	NT
IP	HeLa	NT
	MCF10A	NT
	LCL57	NT
IHC		N*
NCI60		NT

N – Negative, NT – Not Tested, *Data not shown
†Data shown are from Phospho-specificity Experiment

Endogenous Wes

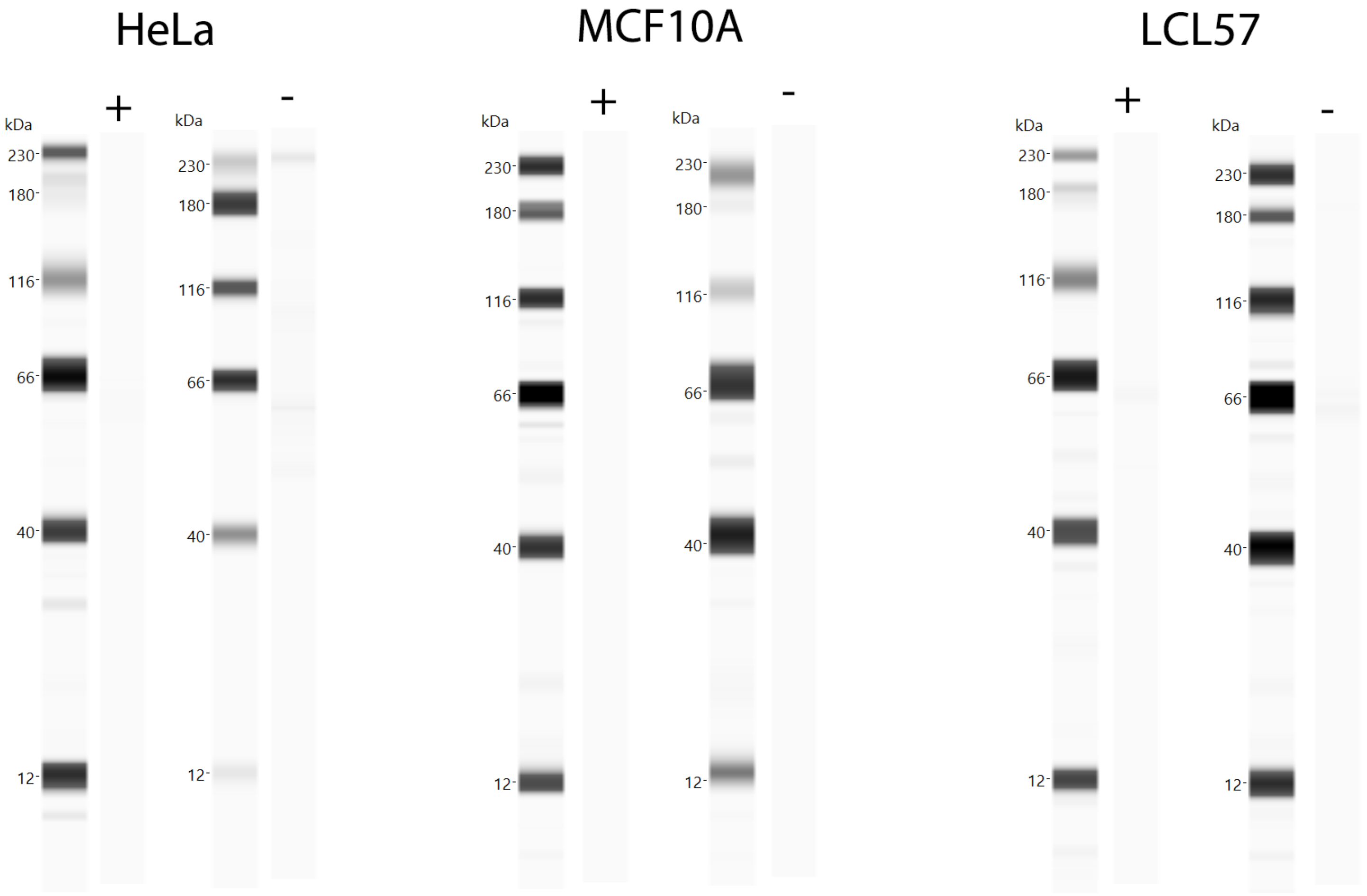

Expected molecular weight: 31 kDa

+ : irradiation treated cells
- : not treated cells

7. CPTC-CDK1-1

Technique	Analyte	Result
WB	RecProt	P
Wes	HeLa	P
	MCF10A	P
	LCL57	P
WB	HeLa	P
	MCF10A	P
	LCL57	P
IF	HeLa	P
	MCF10A	P
	LCL57	P
SCWB	HeLa	P
	MCF10A	P
	LCL57	P
IP*	RecProt	W
IP*	HeLa	N
	MCF10A	N
	LCL57	N
IHC		N†
NCI60		NT

P – Positive, N – Negative, W – Weak, * Please refer to tables for results, †Data not shown

WB against commercial recombinant protein

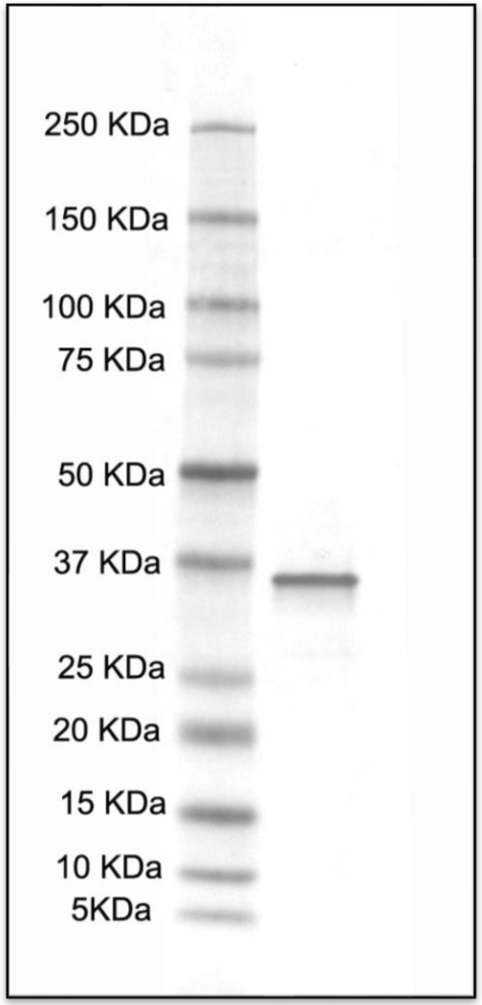

Ag Vendor: Origene

Cat #: TP300495

Description: CDK1 (NM_001786) Human Recombinant Protein
Recombinant protein of human cell division cycle 2, G1 to S and G2 to M (CDC2), transcript variant 1n

Predicted MW 33.9 KDa

Endogenous WB

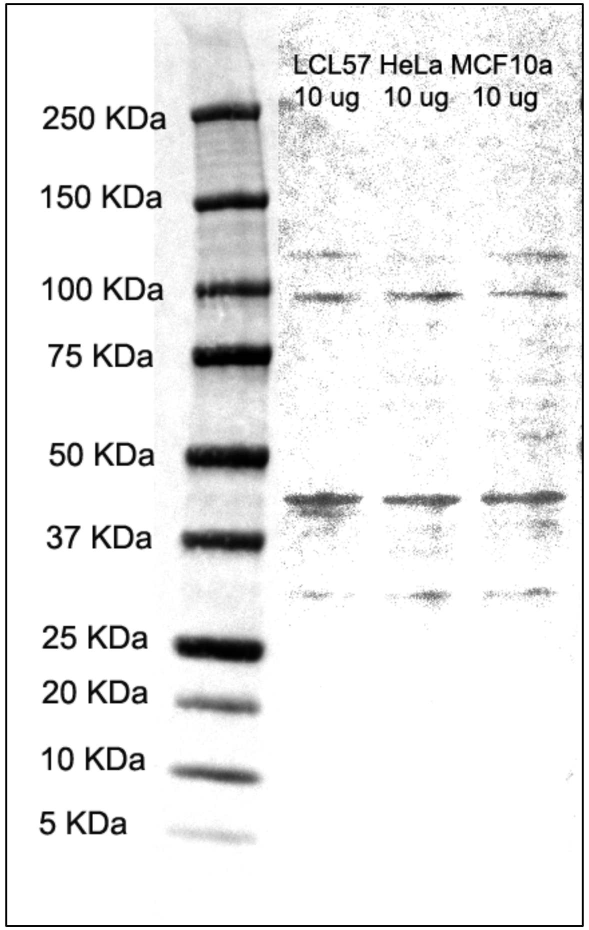

Endogenous Wes

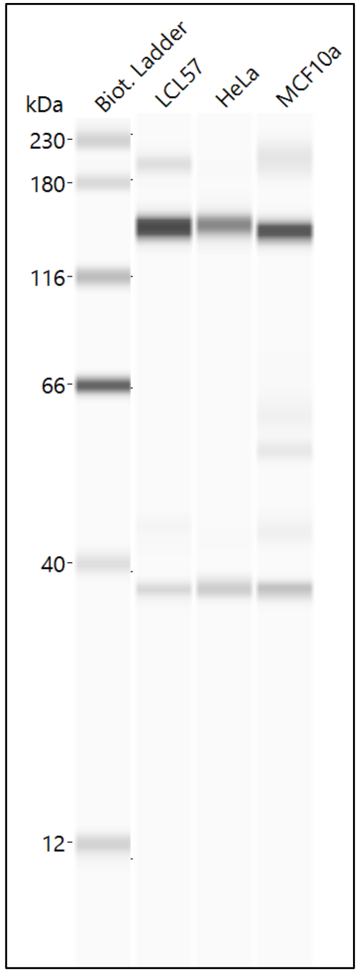

Immunofluorescence – HeLa

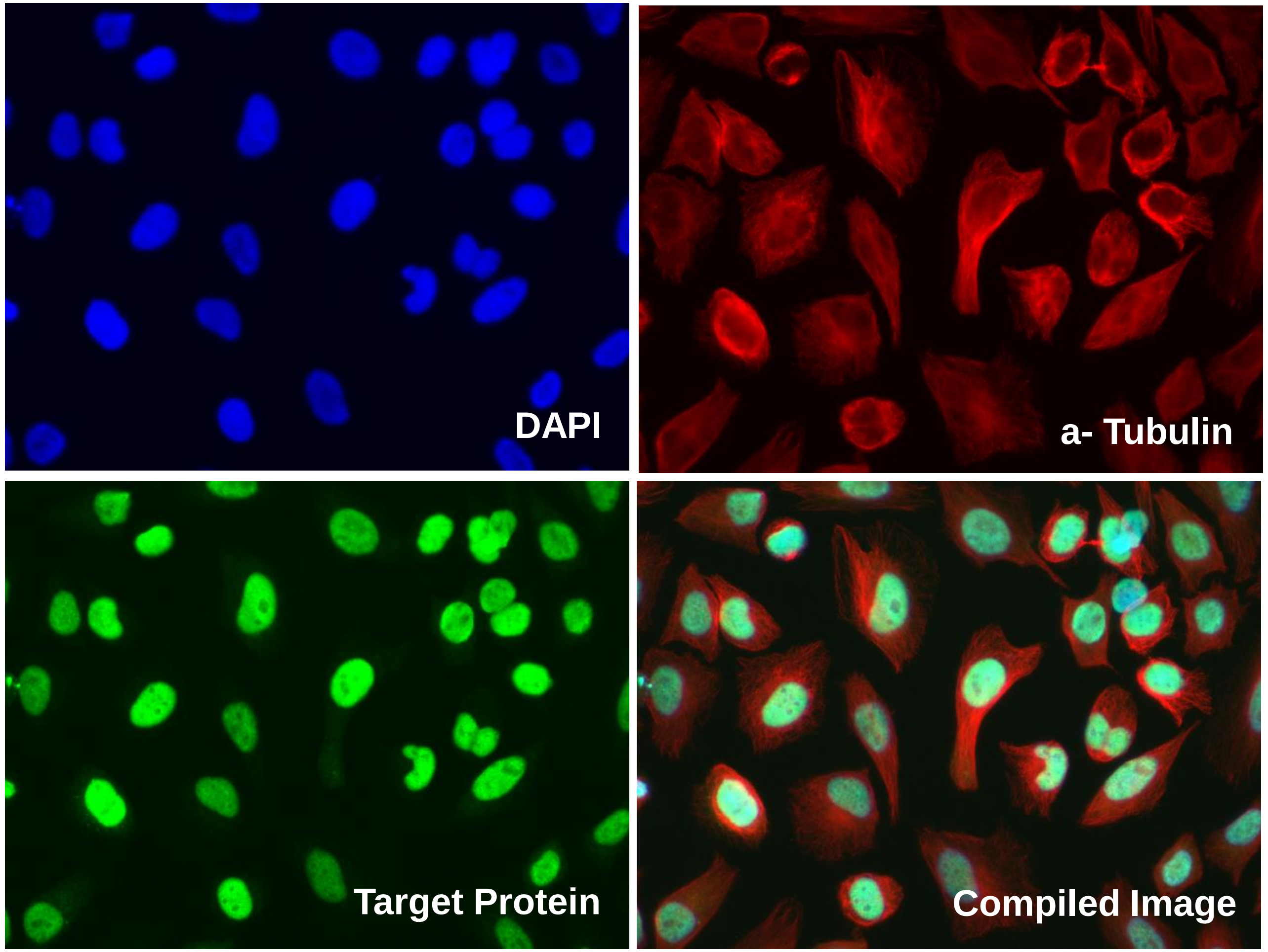

Subcelluar Locations: Mitochondrion, Nucleus, Cytoskeleton, Other

Immunofluorescence – MCF10A

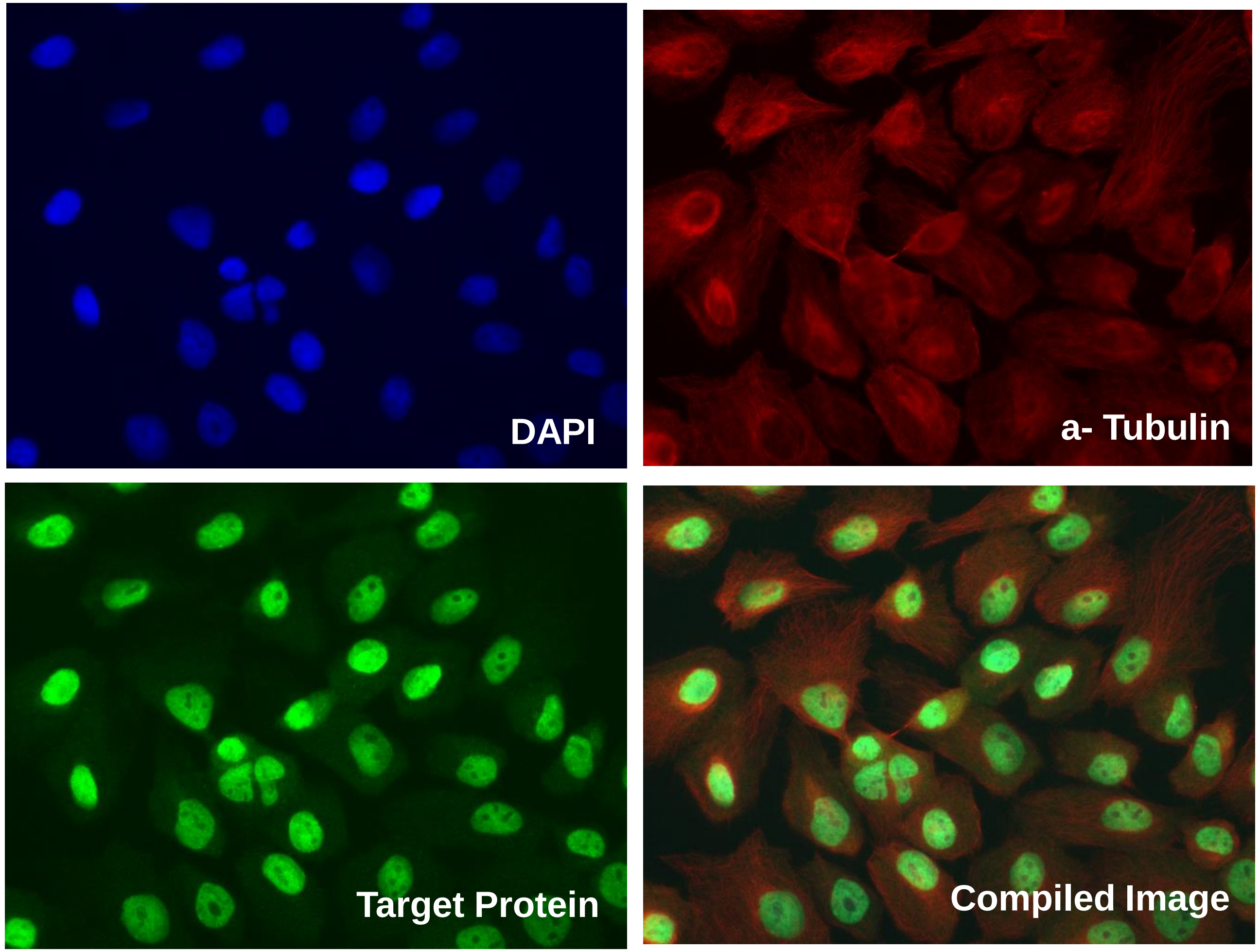

Subcelluar Locations: Mitochondrion, Nucleus, Cytoskeleton, Other

Immunofluorescence – LCL57

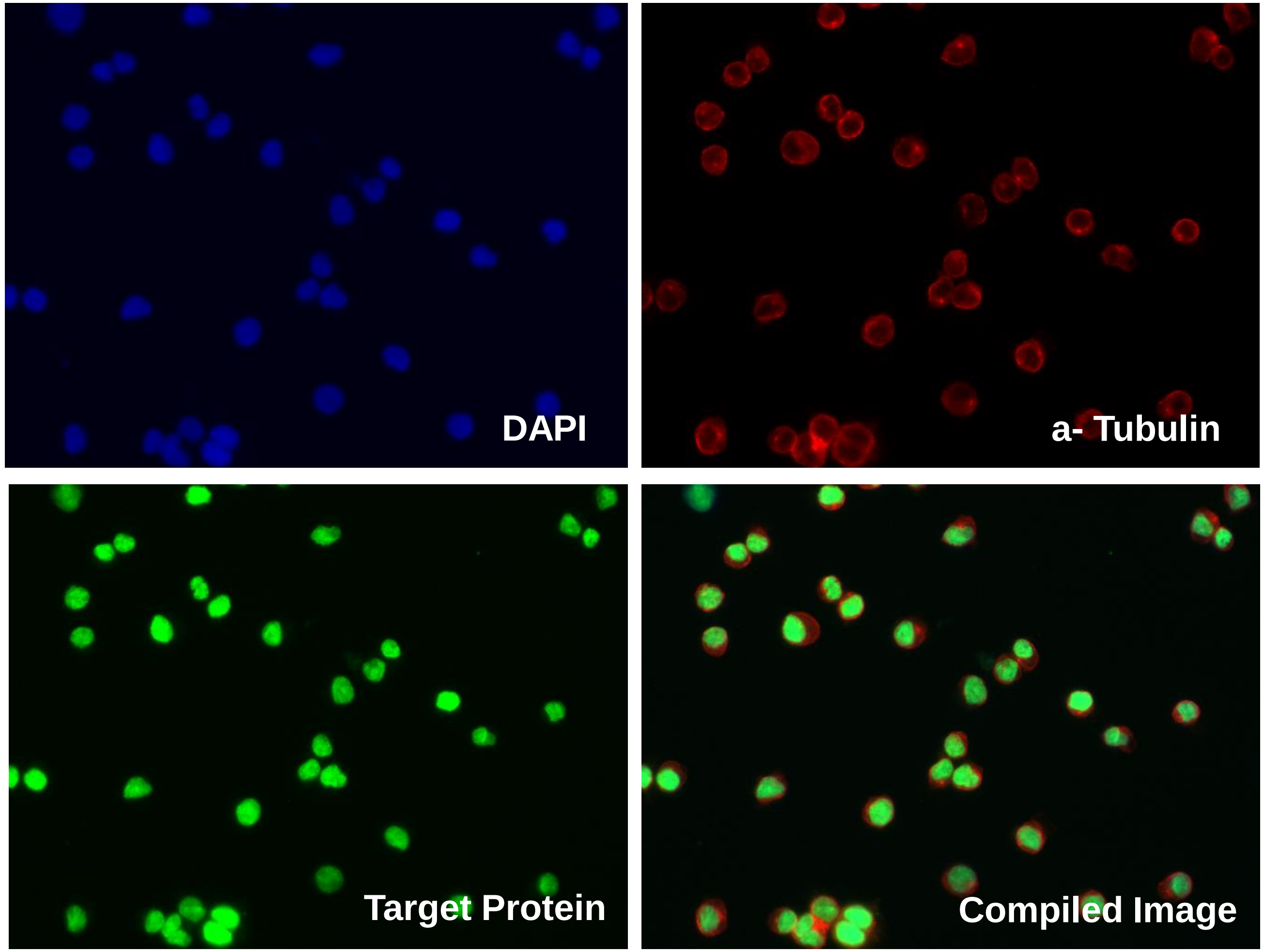

Subcelluar Locations: Mitochondrion, Nucleus, Cytoskeleton, Other

Single Cell Western Blot

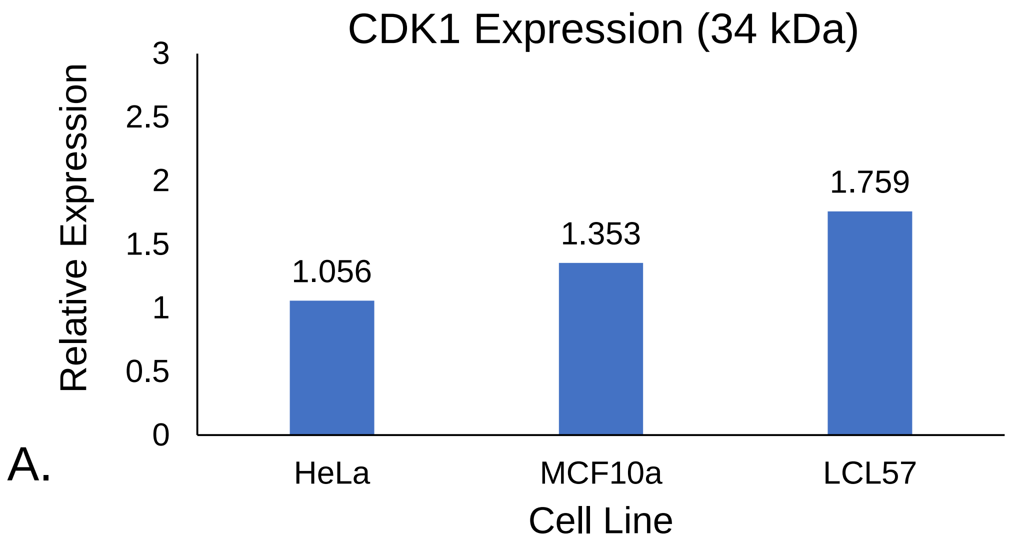

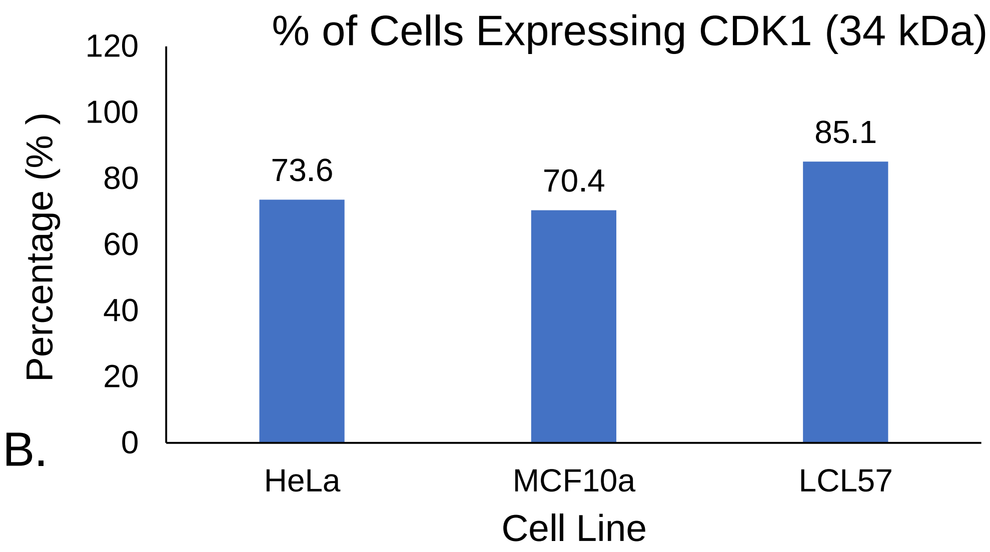

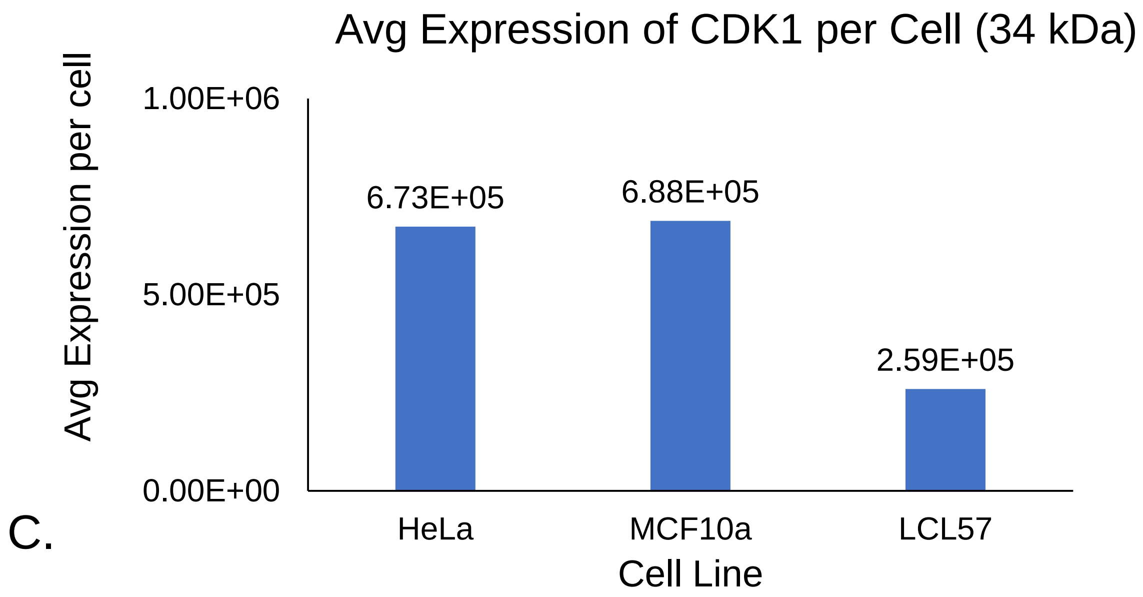

Single cell western blot data for CDK1 (34 kDa band). Relative expression of total CDK1 (34 kDa) in HeLa, MCF10A, and LCL57 cells (A). Percentage of cells that express CDK1 (B). Average expression of CDK1 protein per cell (C). All data is normalized to β-tubulin expression.

Single Cell Western Blot

A.

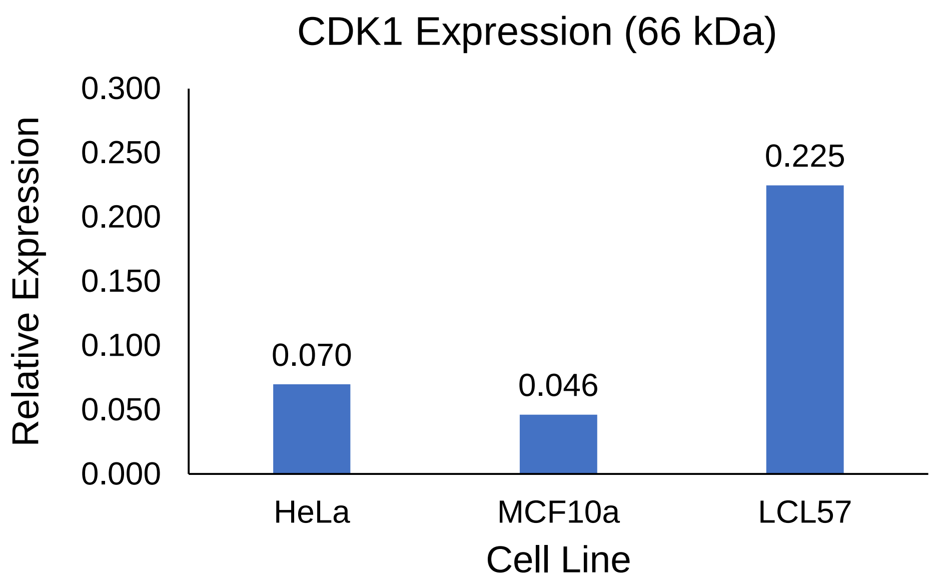

B.

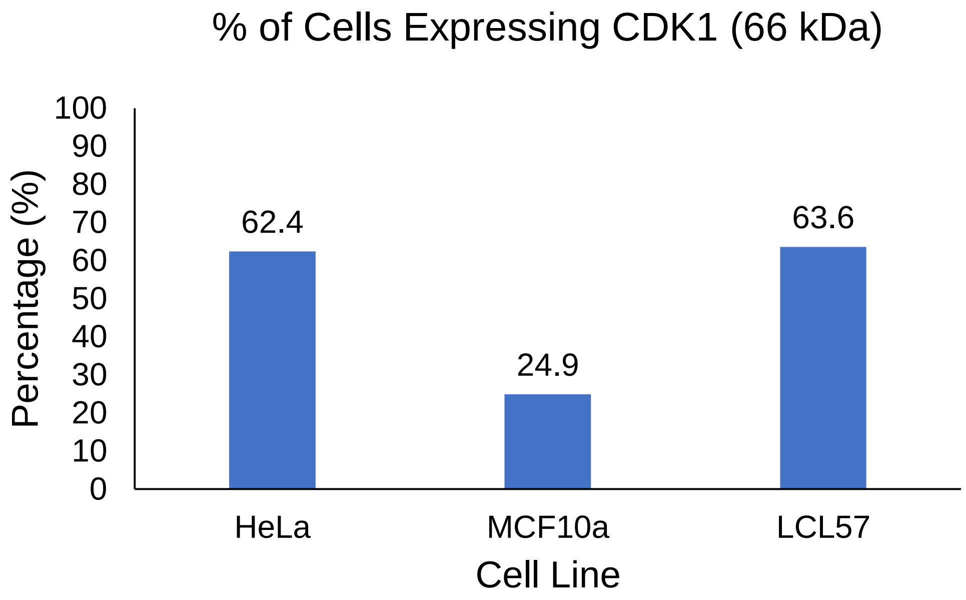

C.

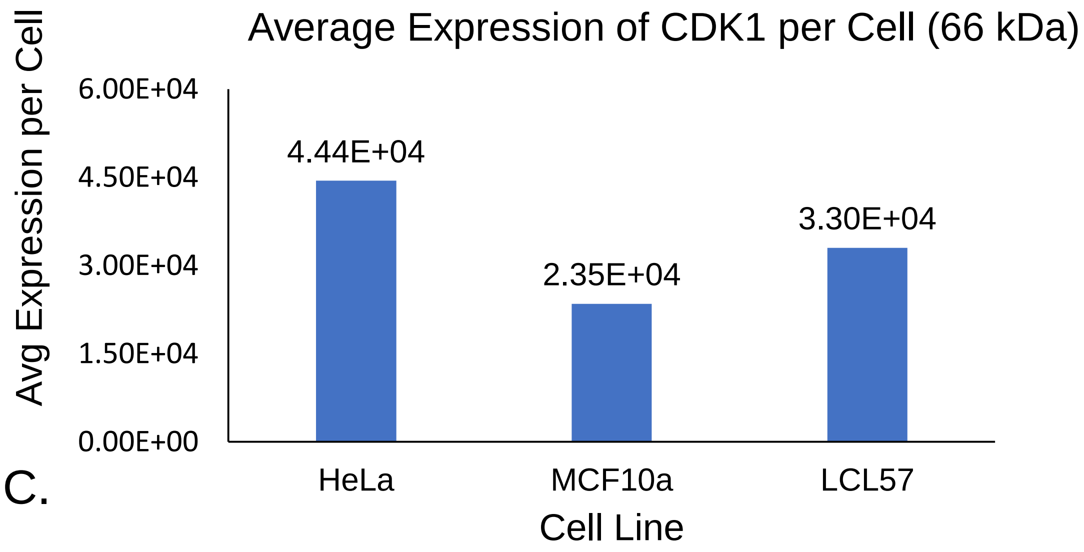

Single cell western blot data for CDK1 (66 kDa band). Relative expression of total CDK1 (66 kDa) in HeLa, MCF10A, and LCL57 cells (A). Percentage of cells that express CDK1 (B). Average expression of CDK1 protein per cell (C). All data is normalized to β-tubulin expression.

Single Cell Western Blot

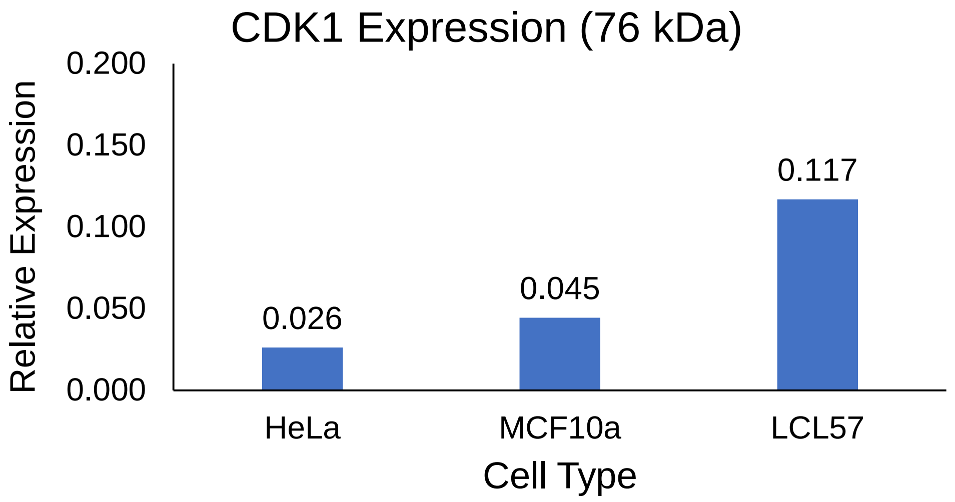

A.

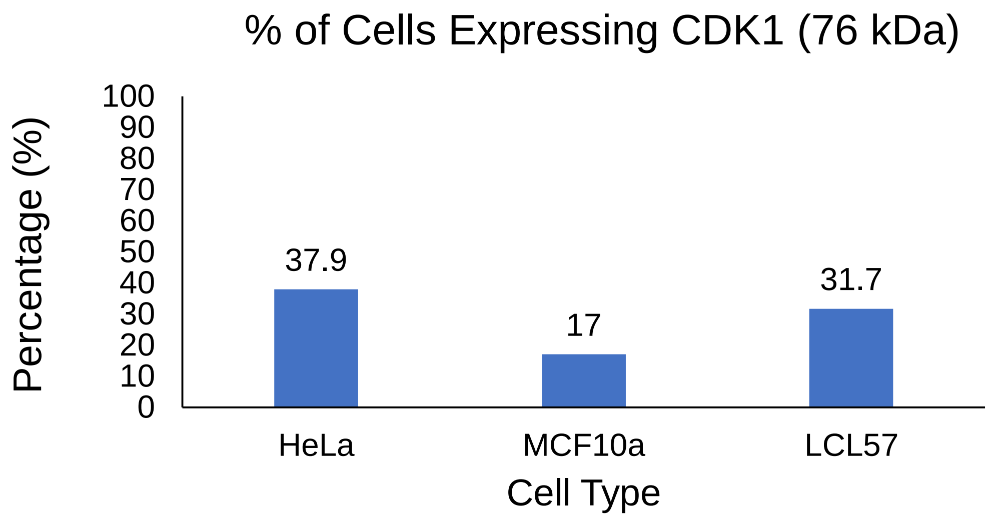

B.

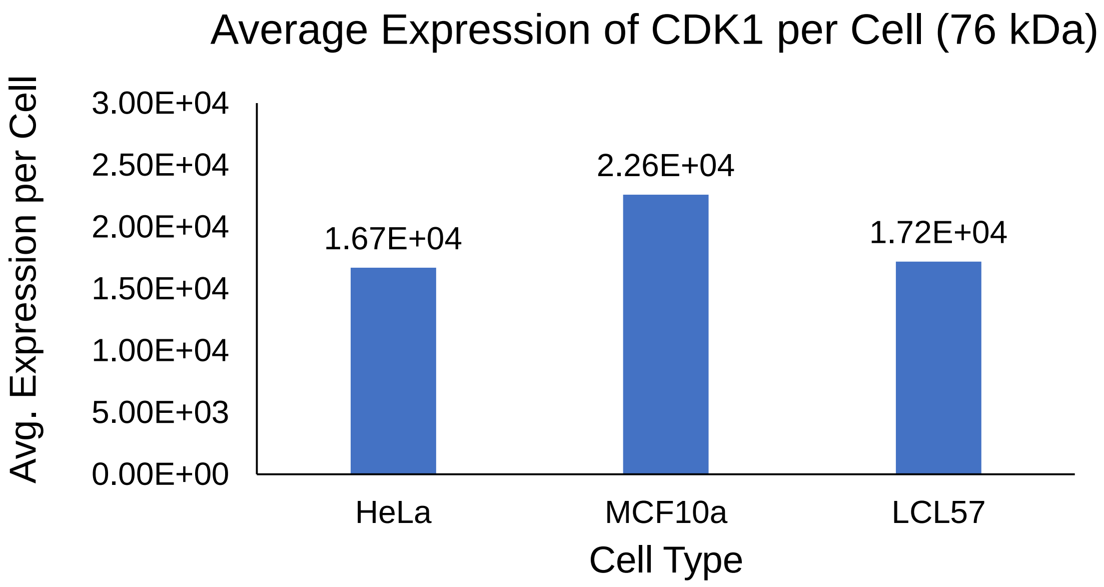

C.

Single cell western blot data for CDK1 (76 kDa band). Relative expression of total CDK1 (76 kDa) in HeLa, MCF10A, and LCL57 cells (A). Percentage of cells that express CDK1 (B). Average expression of CDK1 protein per cell (C). All data is normalized to β-tubulin expression.

8. CPTC-CDK7-1

Technique	Analyte	Result
WB	RecProt	P
Wes	HeLa	P
	MCF10A	P
	LCL57	P
WB	HeLa	P
	MCF10A	P
	LCL57	P
IF	HeLa	P
	MCF10A	N†
	LCL57	P
SCWB	HeLa	P
	MCF10A	P
	LCL57	P
IP*	RecProt	P
IP*	HeLa	N
	MCF10A	N
	LCL57	N
IHC		P
NCI60		P

P – Positive, N – Negative, * Please refer to tables for results, † Data not shown

WB against commercial recombinant protein

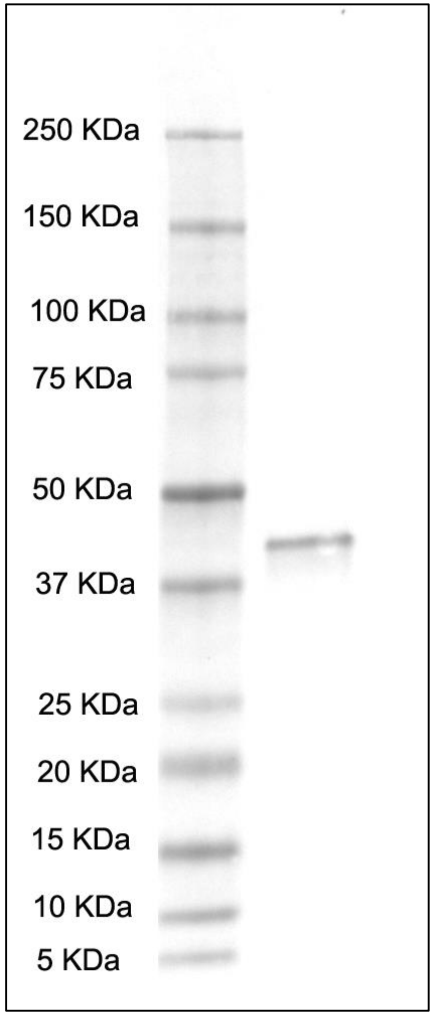

Ag Vendor: Origene

Cat #: TP302736

Description: Recombinant protein of human cyclin-dependent kinase 7 (CDK7)

Predicted MW: 38.9 kDa

Endogenous WB

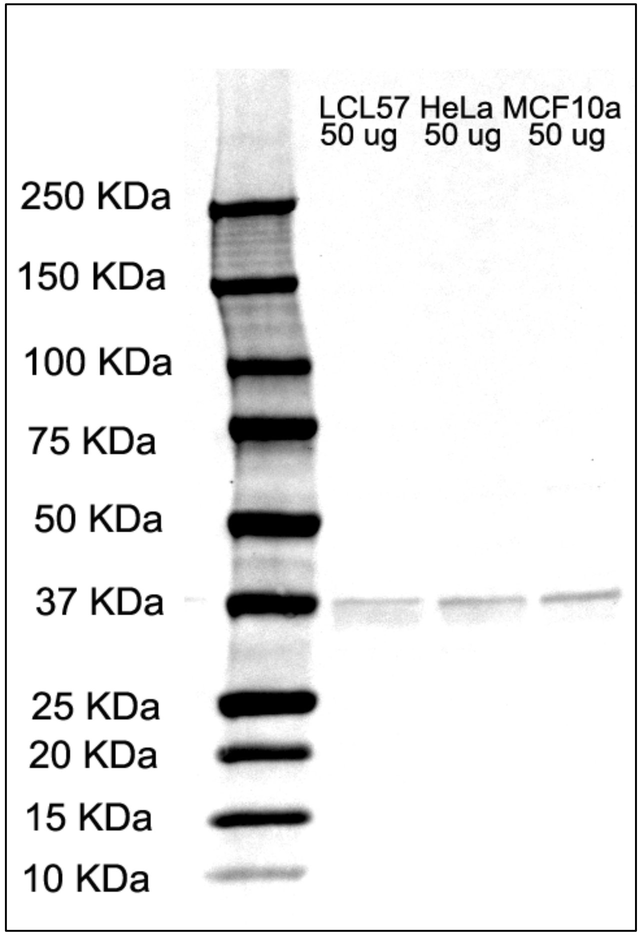

Endogenous Wes

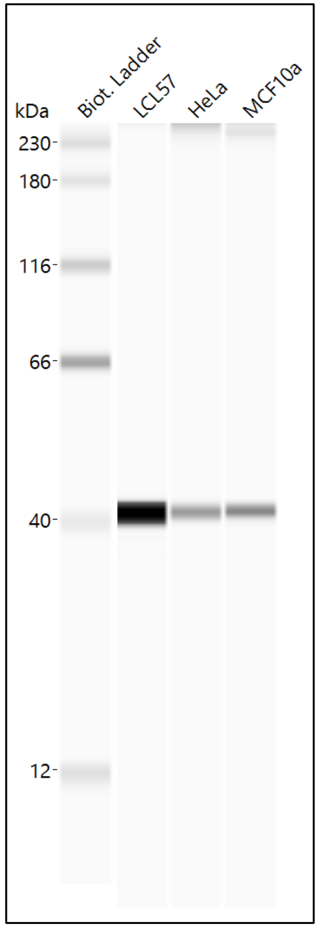

Immunofluorescence - HeLa

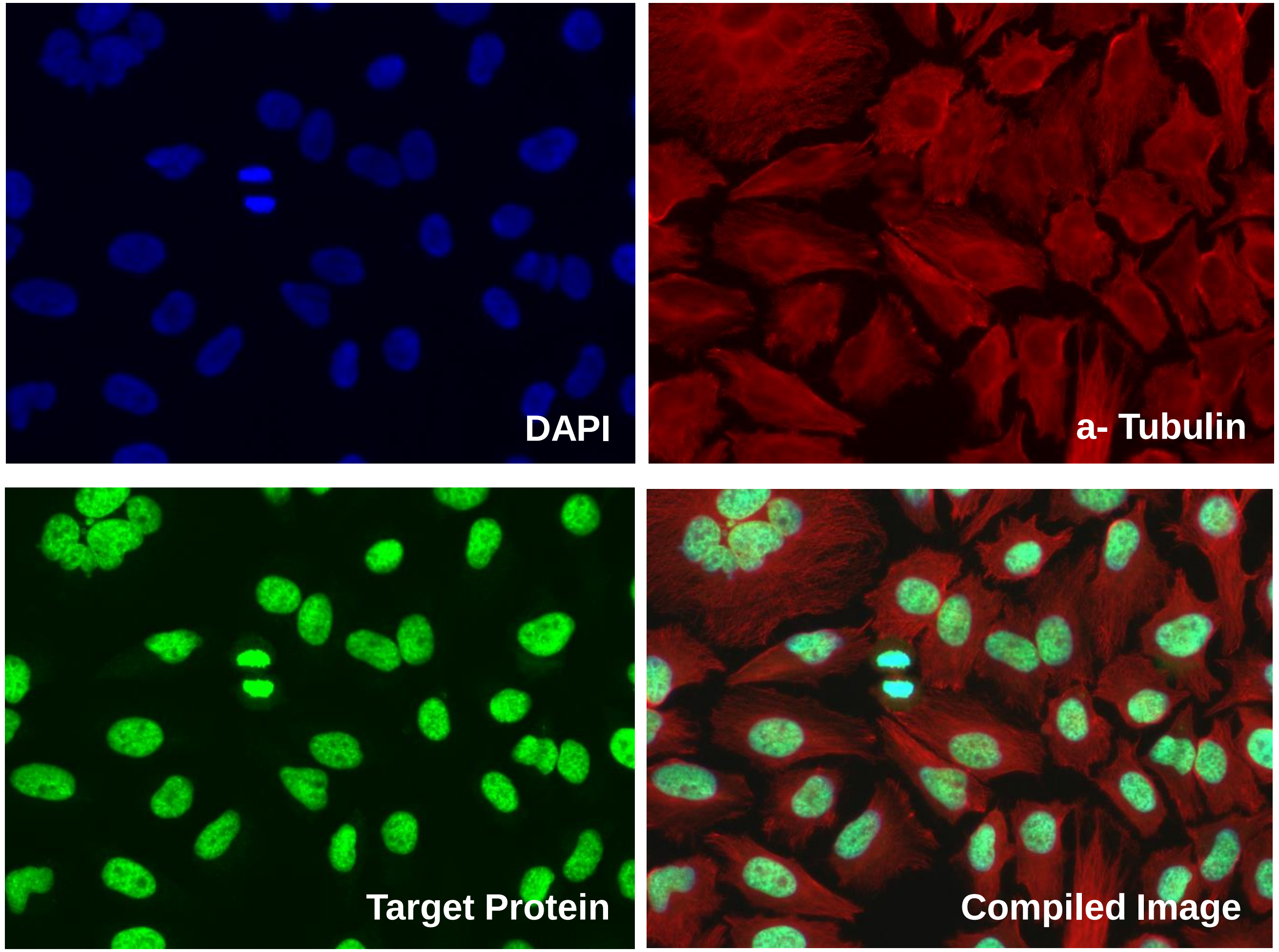

Subcellular Location: Nucleus, Other

Immunofluorescence - LCL57

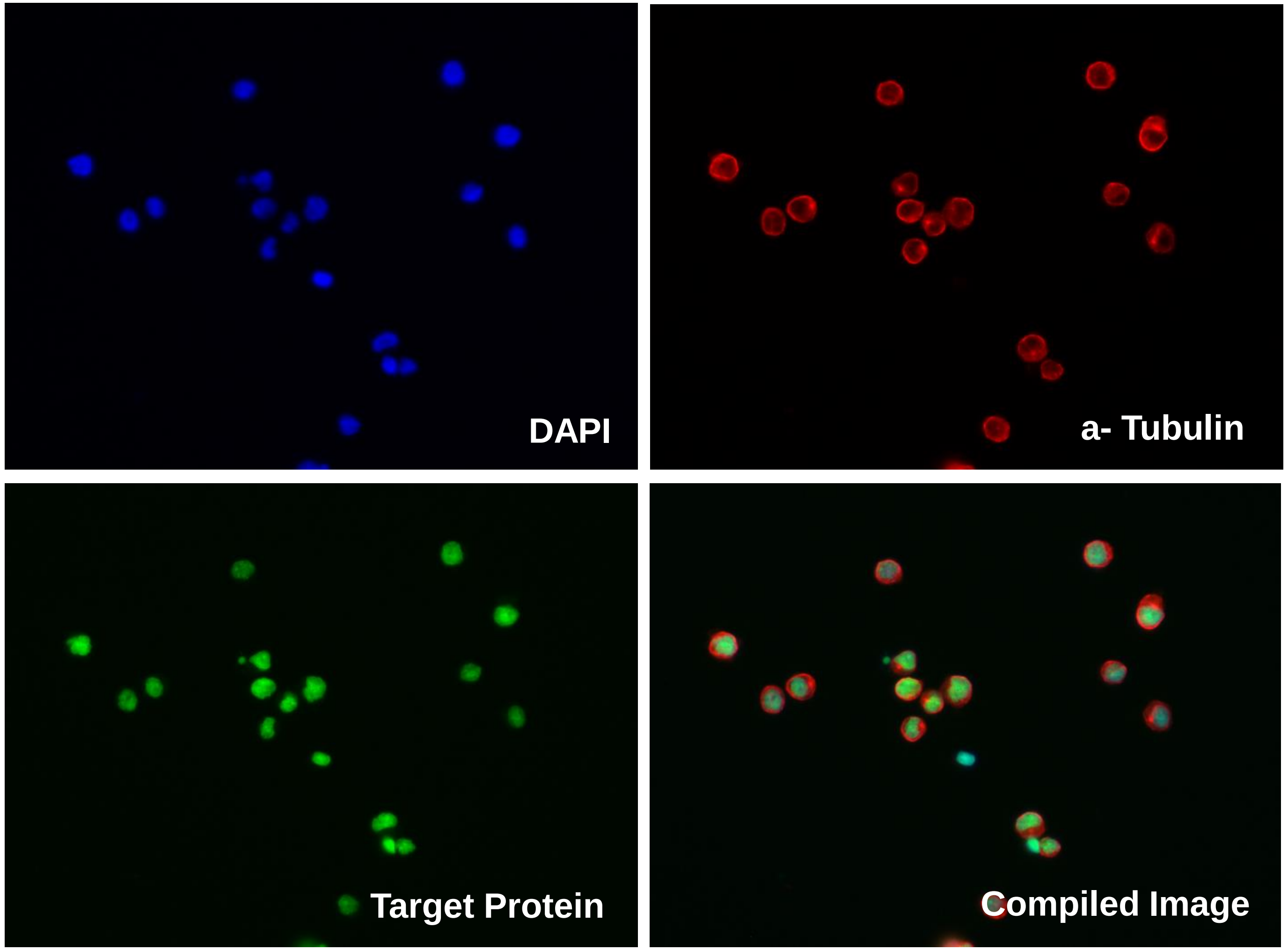

Subcellular Location: Nucleus, Other

Single Cell Western Blot

A.

B.

C.

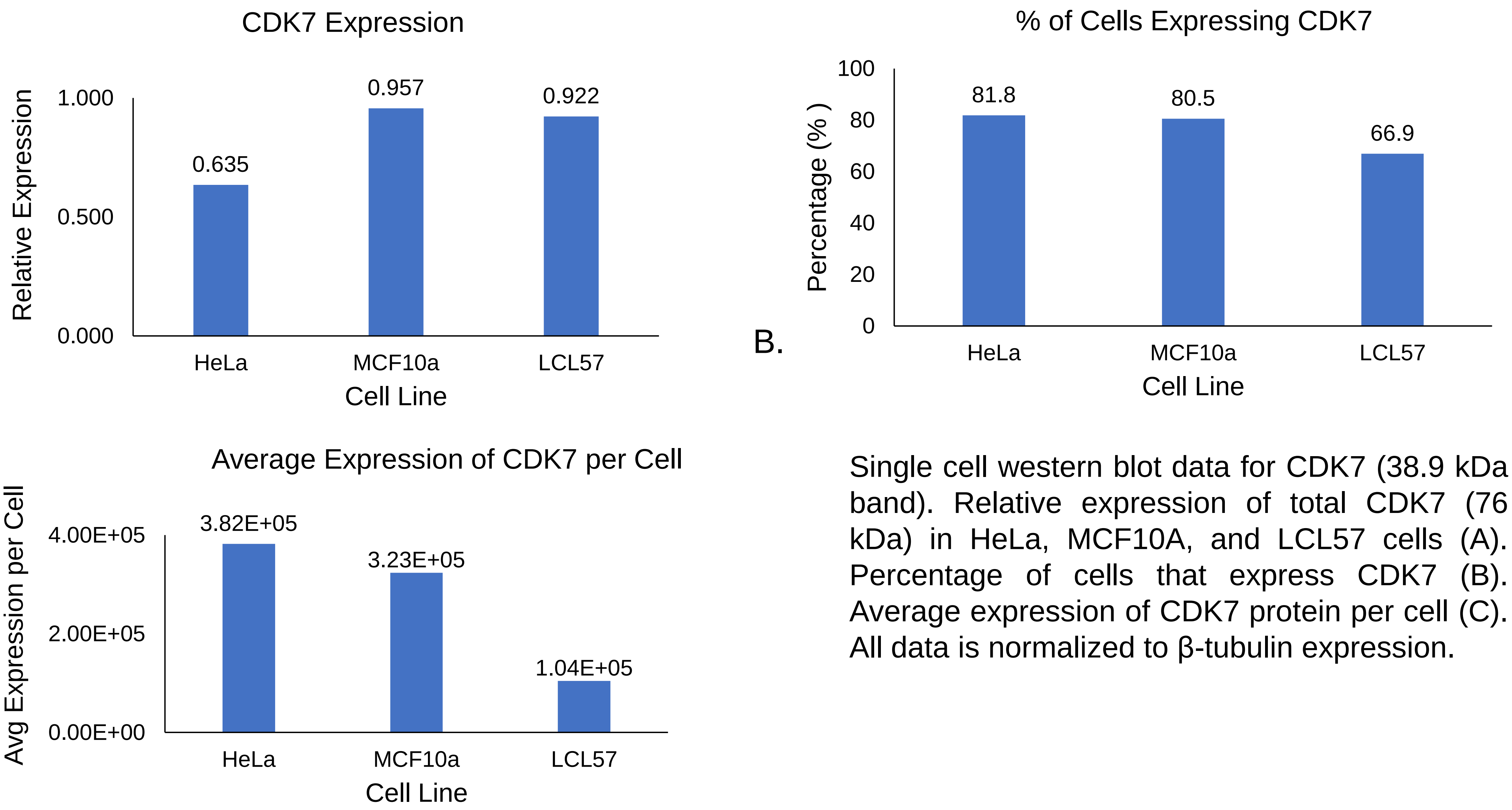

Single cell western blot data for CDK7 (38.9 kDa band). Relative expression of total CDK7 (76 kDa) in HeLa, MCF10A, and LCL57 cells (A). Percentage of cells that express CDK7 (B). Average expression of CDK7 protein per cell (C). All data is normalized to β-tubulin expression.

CPTC-CDK7-1

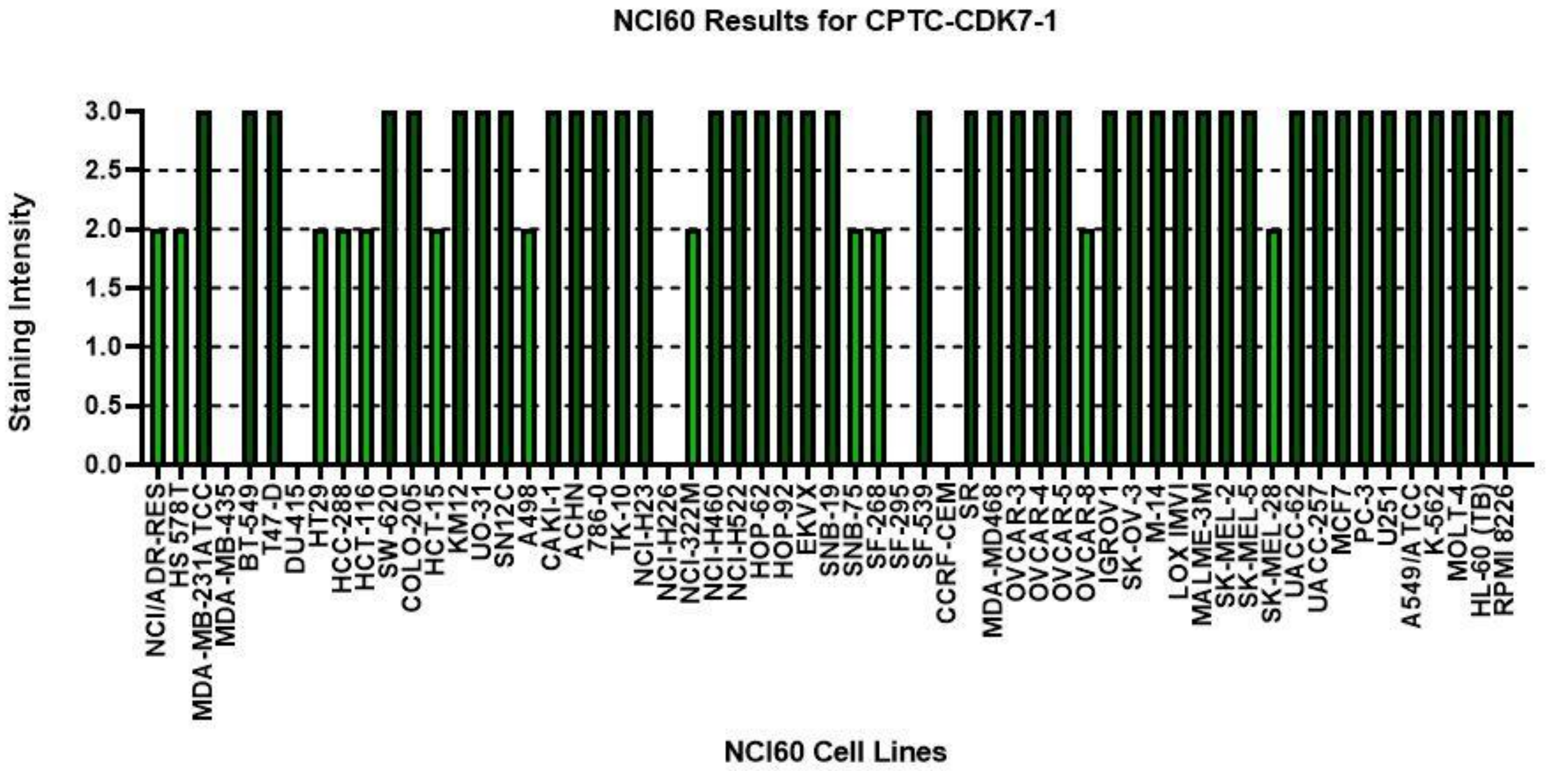

NCI60 Results for CPTC-CDK7-1
Staining Intensity
3.0
2.5
2.0
1.5
1.0
0.5
0.0
NCI/ADR-RES
HS 578T
MDA-MB-231ATCC
MDA-MB-435
BT-549
T47-D
DU-415
HT29
HCC-288
HCT-116
SW-620
COLO-205
HCT-15
KM12
UO-31
SN12C
A498
CAKI-1
ACHN
786-0
TK-10
NCI-H23
NCI-H226
NCI-322M
NCI-H460
NCI-H522
HOP-62
HOP-92
EKVX
SNB-19
SNB-75
SF-268
SF-295
SF-539
CCRF-CEM
SR
MDA-MD468
OVCAR-3
OVCAR-4
OVCAR-5
OVCAR-8
IGROV1
SK-OV-3
M-14
LOX IMVI
MALME-3M
SK-MEL-2
SK-MEL-5
SK-MEL-28
UACC-62
UACC-257
MCF7
PC-3
U251
A549/ATCC
K-562
MOLT-4
HL-60 (TB)
RPMI 8226
NCI60 Cell Lines

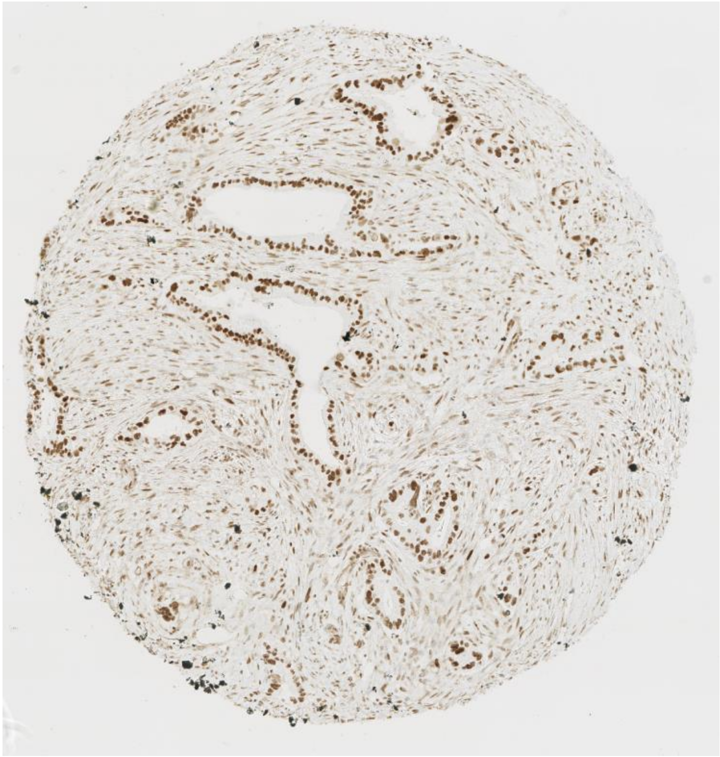

9. CPTC-CHEK1-6

Technique	Analyte	Result
WB	RecProt	P
Wes	HeLa	N
	MCF10A	P
	LCL57	N
WB	HeLa	P
	MCF10A	P
	LCL57	P
IF	HeLa	N†
	MCF10A	P
	LCL57	N†
SCWB†	HeLa	N
	MCF10A	N
	LCL57	N
IP*	RecProt	W
IP*	HeLa	N
	MCF10A	N
	LCL57	N
IHC		N†
NCI60		NT

P – Positive, N – Negative, W – Weak, * Please refer to tables for results, † Data not shown

WB against commercial recombinant protein

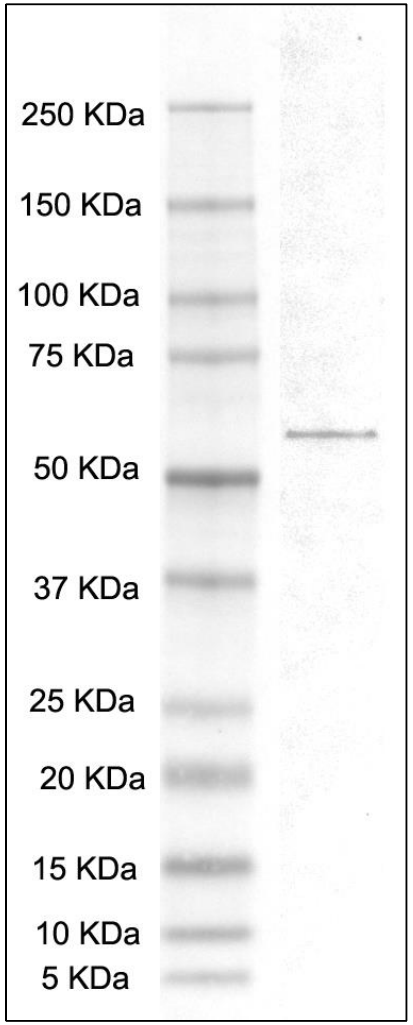

Ag Vendor: Origene

Cat #: TP325807

Description:Purified recombinant protein of Homo sapiens CHK1 checkpoint homolog (S. pombe) (CHEK1), transcript variant 3

Predicted MW: 54.3 kDa

Endogenous WB

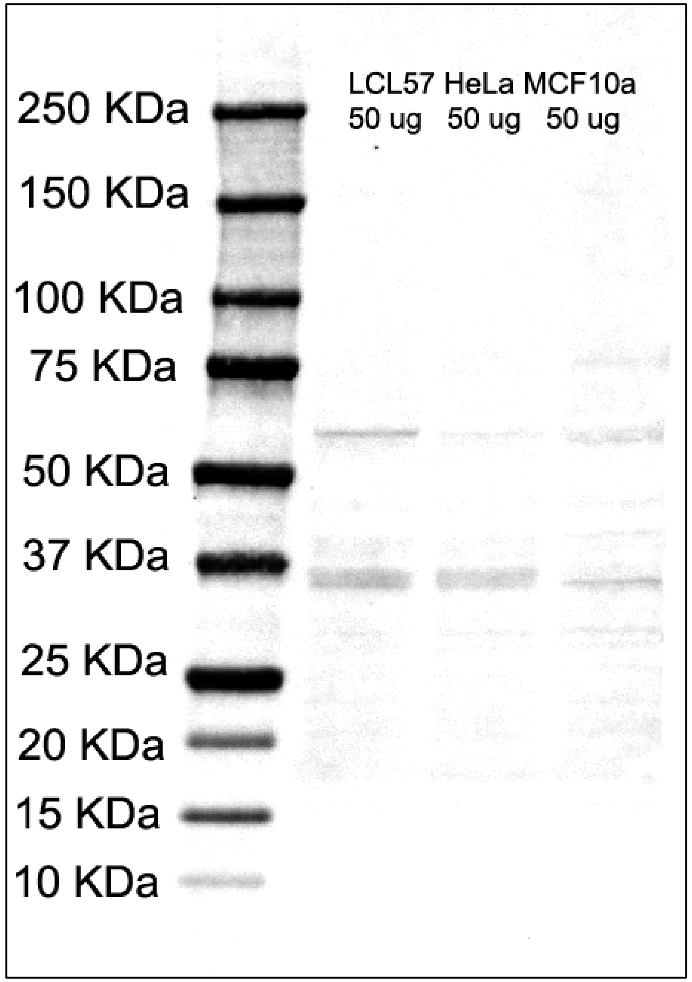

Endogenous Wes

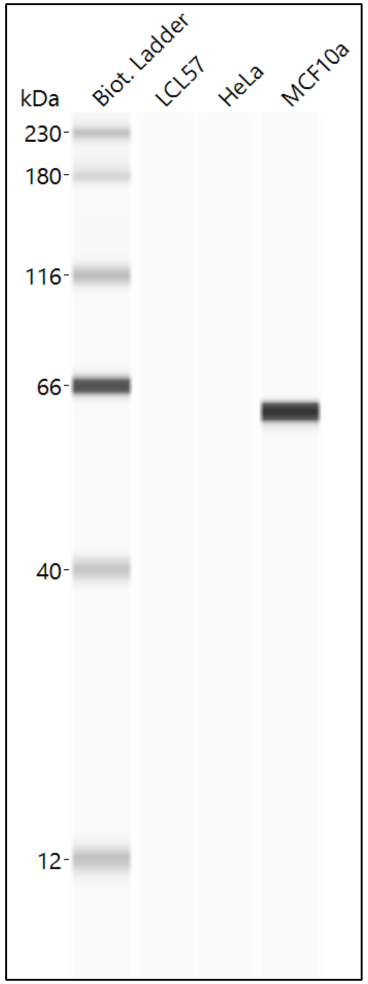

Immunofluorescence - MCF10A

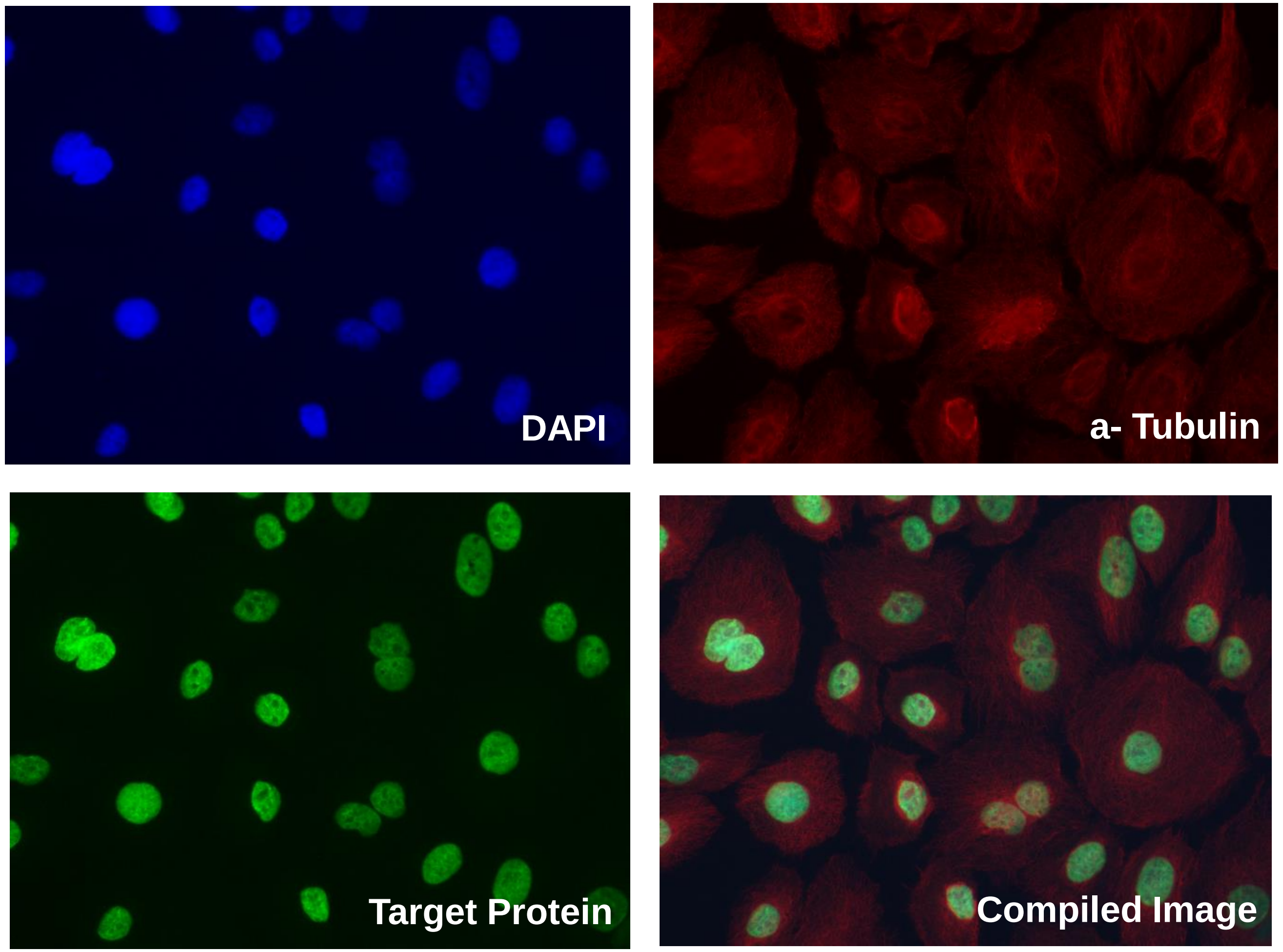

Subcelluar Location: Nucleus, Cytoskeleton, Other

10. CPTC-CHEK2-1

Technique	Analyte	Result
WB	RecProt	N*
West†	HeLa	N
	MCF10A	N
	LCL57	N
WB	HeLa	NT
	MCF10A	NT
	LCL57	NT
IF	HeLa	NT
	MCF10A	NT
	LCL57	NT
SCWB	HeLa	NT
	MCF10A	NT
	LCL57	NT
IP	RecProt	NT
IP	HeLa	NT
	MCF10A	NT
	LCL57	NT
IHC		N*
NCI60		NT

N – Negative, NT – Not Tested, *Data not shown
†Data shown are from Phospho-specificity Experiment

Endogenous Wes

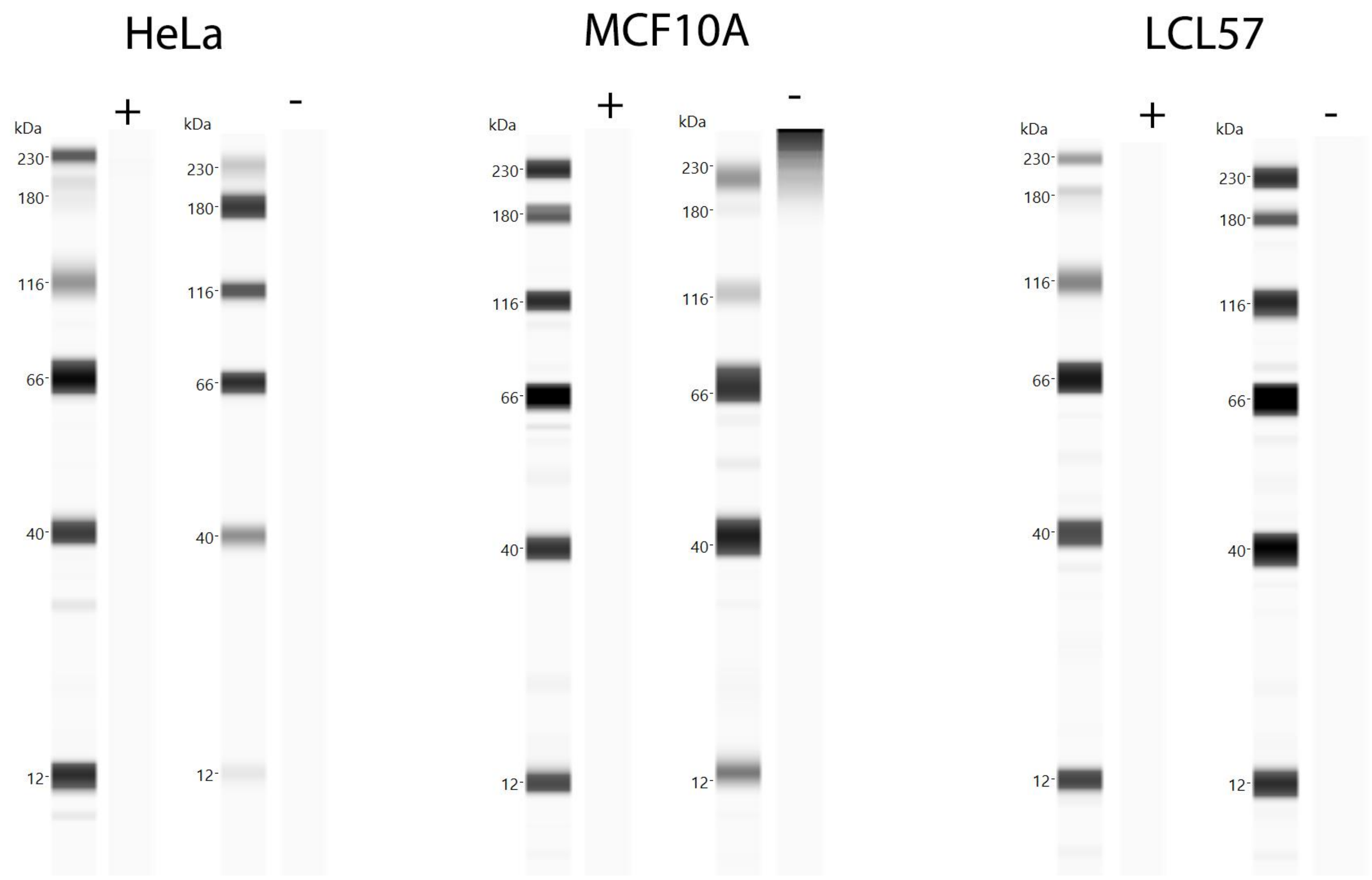

Expected molecular weight: 61 kDa

+ : irradiation treated cells
- : not treated cells

11. CPTC-CHEK2-2

Technique	Analyte	Result
WB	RecProt	P
Wes	HeLa	P
	MCF10A	P
	LCL57	P
WB	HeLa	U
	MCF10A	U
	LCL57	U
IF	HeLa	N†
	MCF10A	N†
	LCL57	P
SCWB†	HeLa	N
	MCF10A	N
	LCL57	N
IP*	RecProt	P
IP*	HeLa	N
	MCF10A	N
	LCL57	N
IHC		N†
NCI60		NT

P – Positive, N – Negative, U – Undetermined, * Please refer to tables for results, † Data not shown

WB against commercial recombinant protein

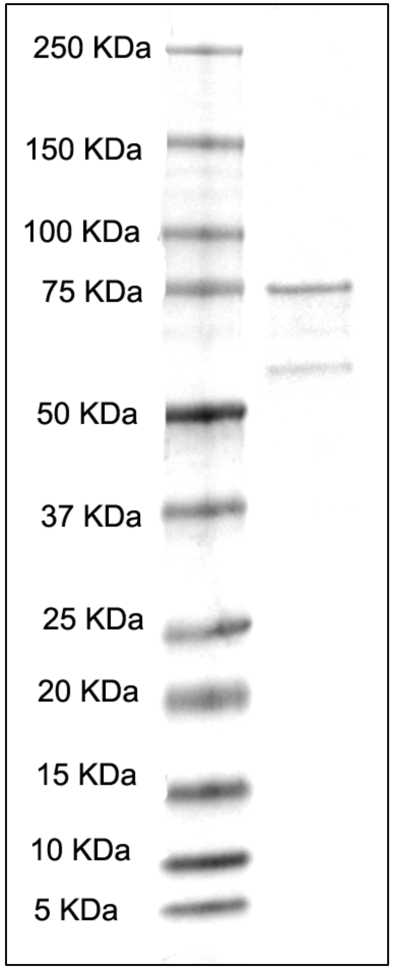

Ag Vendor: Origene

Cat #: TP301278

Description:Recombinant protein of human CHK2 checkpoint homolog (S. pombe) (CHEK2), transcript variant 1

Predicted MW: 60.7 kDa

Endogenous WB

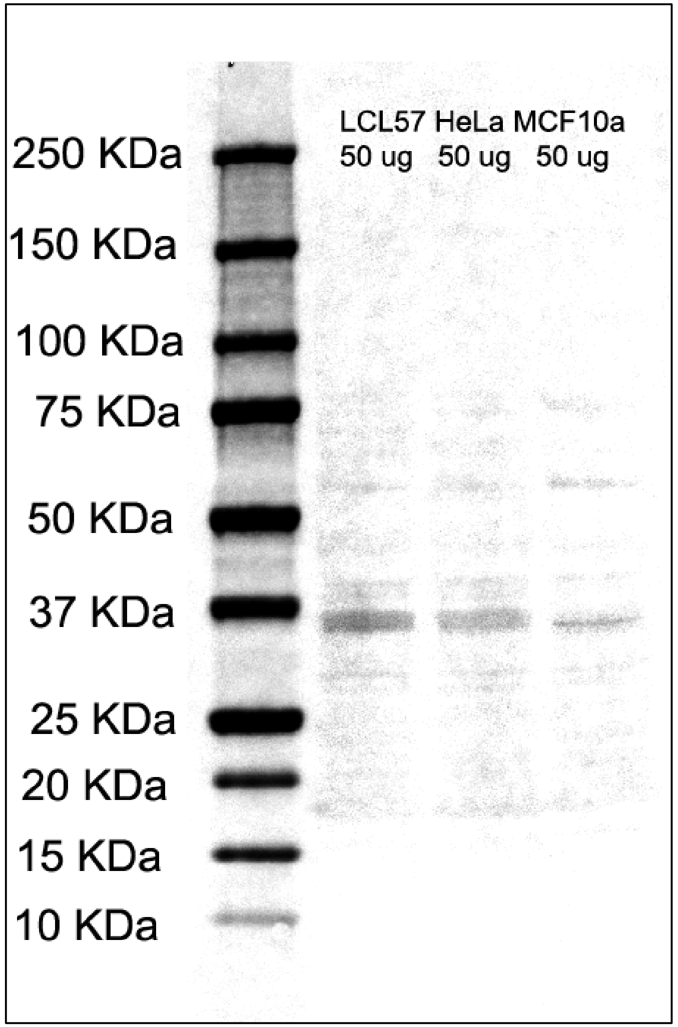

Endogenous Wes

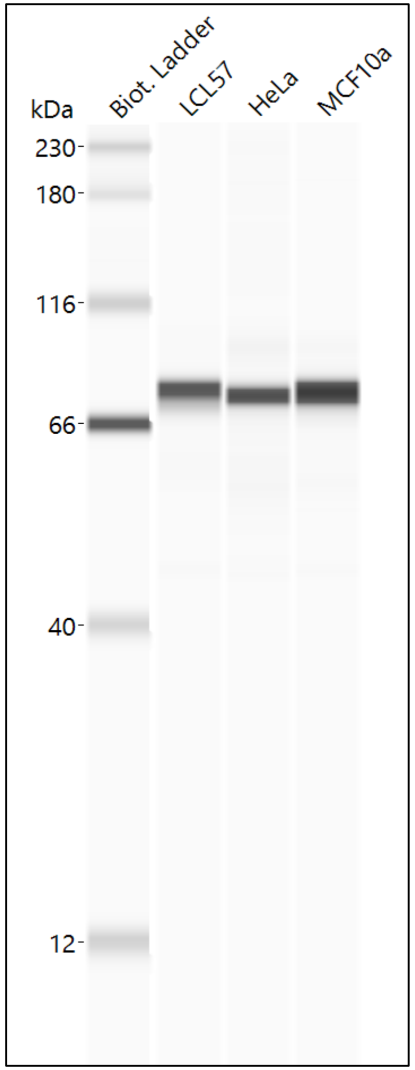

Immunofluorescence - LCL57

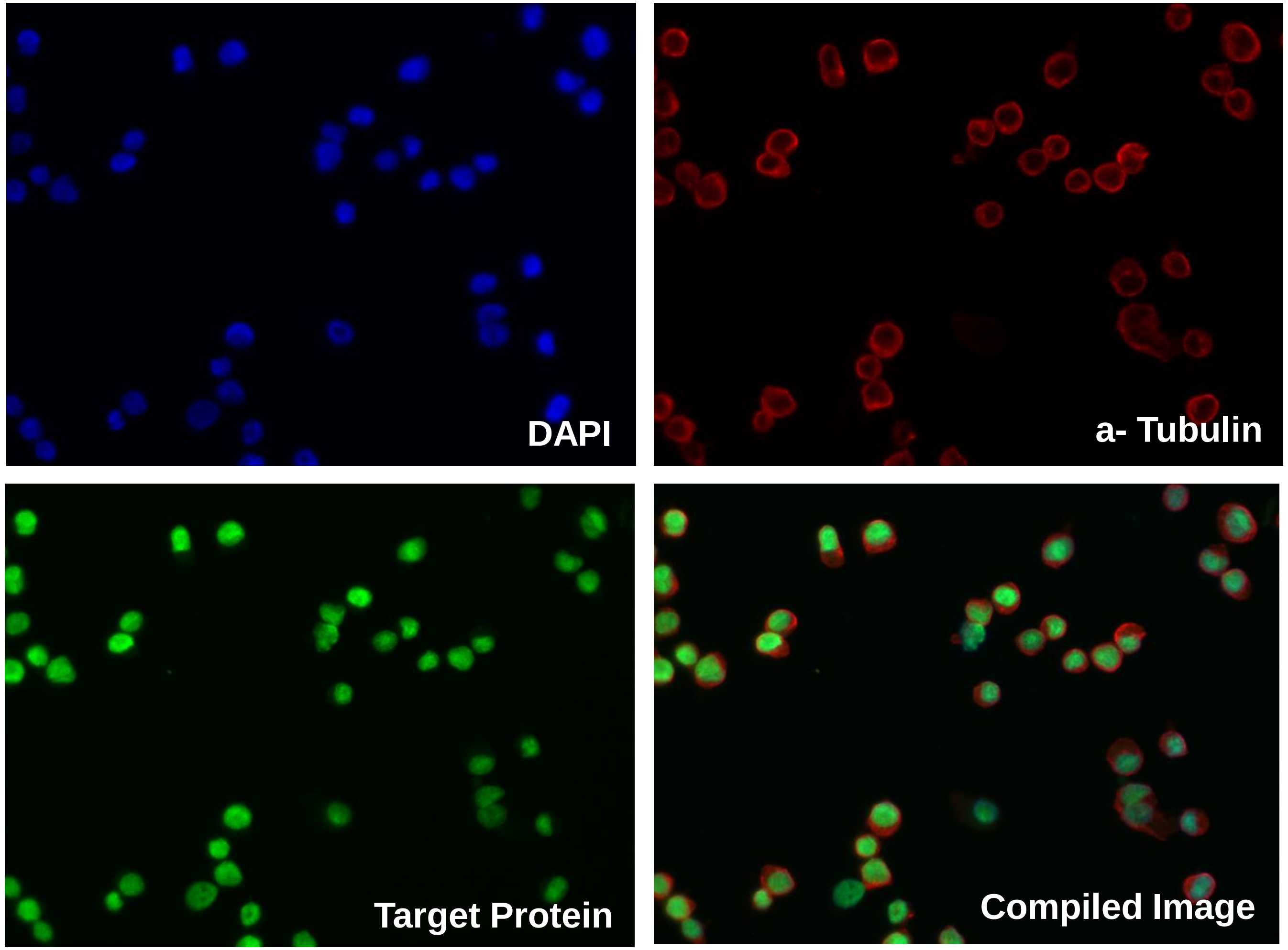

Subcellular Location: Nucleus, Other

12. CPTC-GAPDH-1

Technique	Analyte	Result
WB	RecProt	P
Wes	HeLa	P
	MCF10A	P
	LCL57	P
WB	HeLa	P
	MCF10A	P
	LCL57	P
IF	HeLa	P
	MCF10A	P
	LCL57	P
SCWB†	HeLa	NT
	MCF10A	NT
	LCL57	NT
IP*	RecProt	P
IP*	HeLa	W
	MCF10A	W
	LCL57	N
IHC		N‡
NCI60		NT

P – Positive, N – Negative, NT – Not Tested, W – Weak, * Please refer to tables for results, †The single cell western blot technique used did not provide sufficient denaturing conditions for accurate detection of GAPDH. ‡ Data not shown

WB against commercial recombinant protein

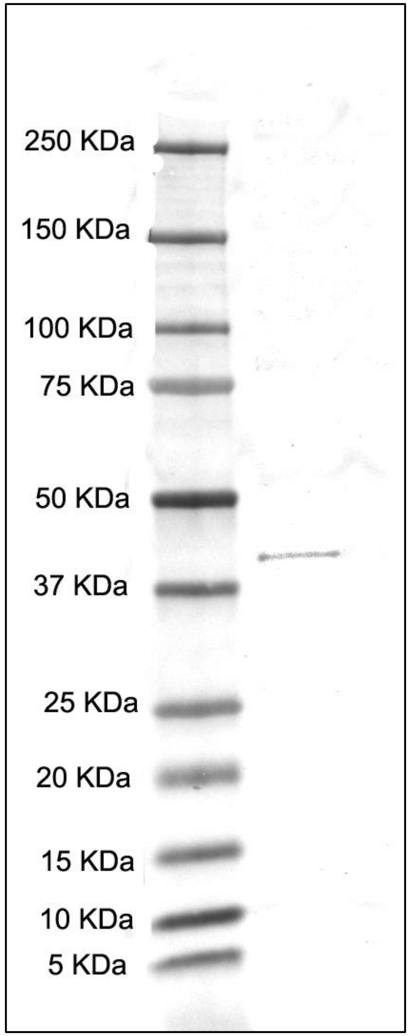

Ag Vendor: Origene

Cat #: TP302309

Description: GAPDH (NM_002046) Human Recombinant Protein

Predicted MW: 36 kDa

Endogenous WB

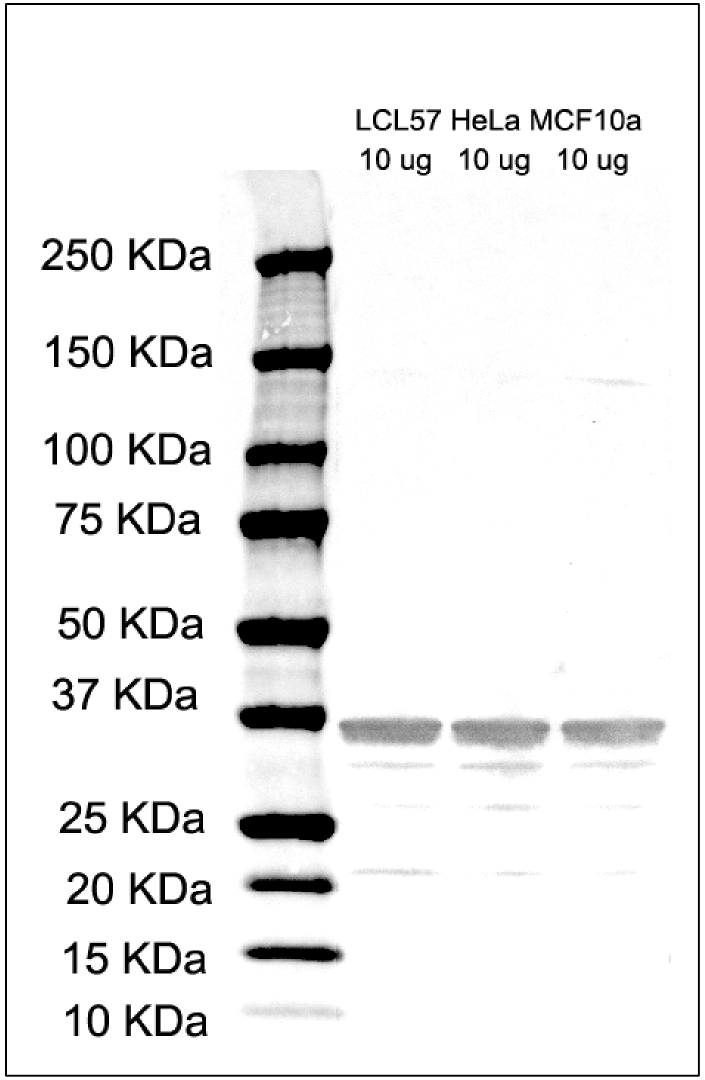

Endogenous Wes

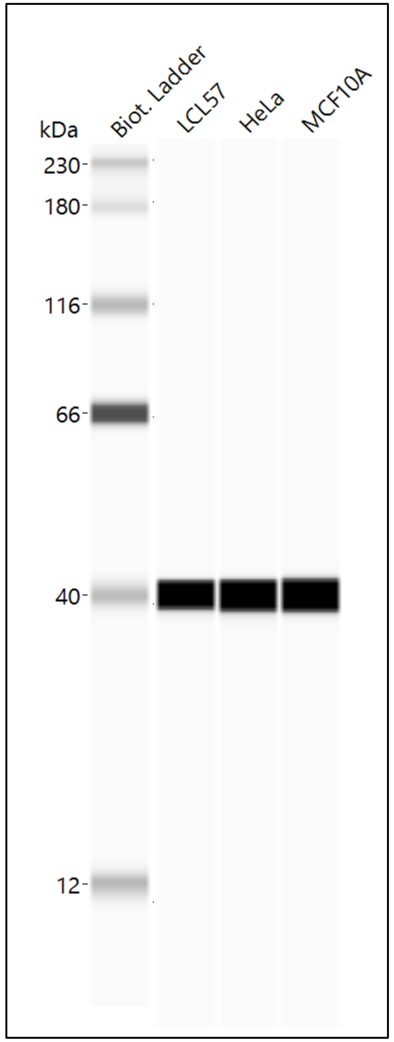

Immunofluorescence - HeLa

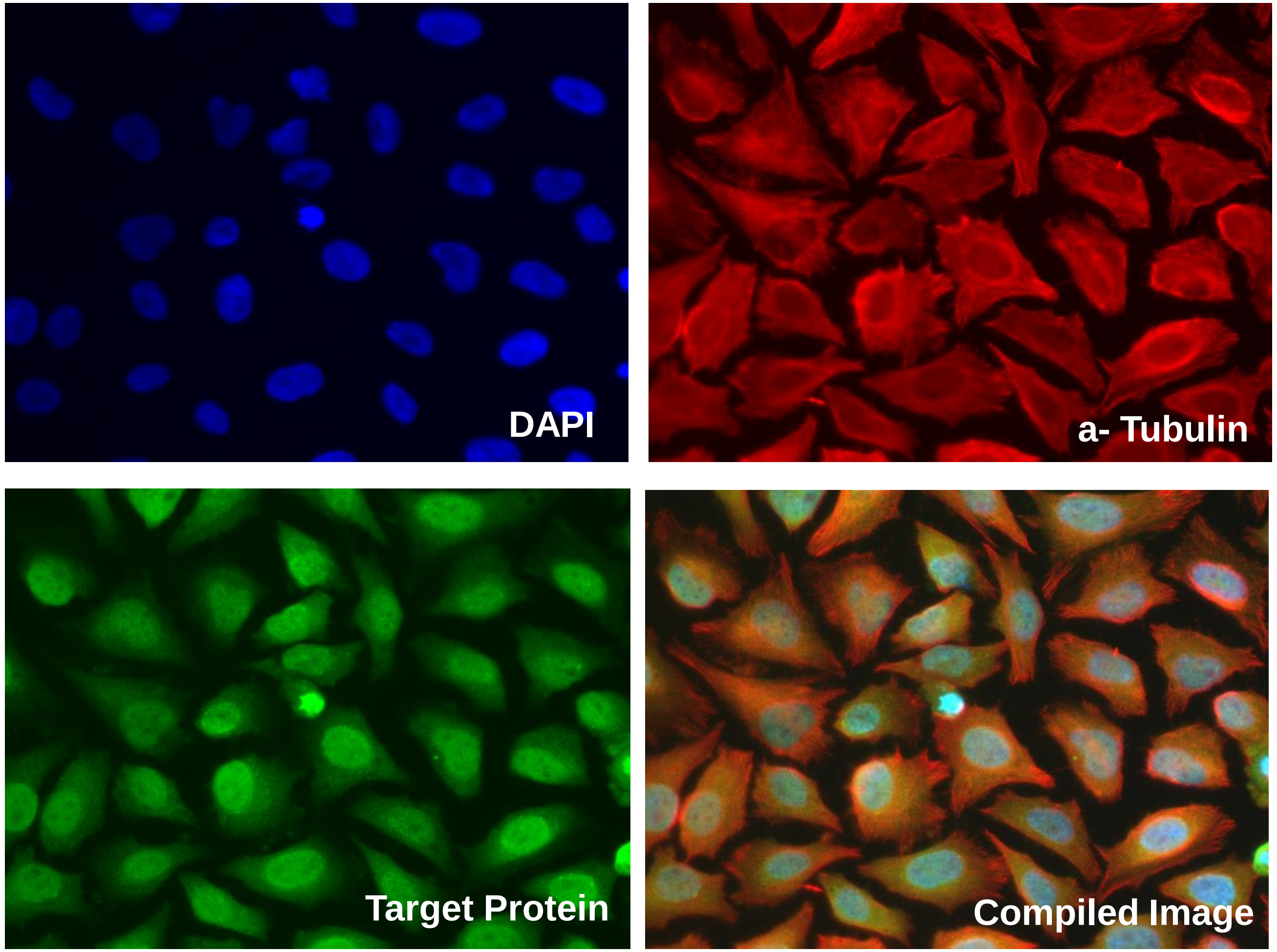

Subcelluar Locations: Cytosol, Cytoskeleton, Nucleus, Other

Immunofluorescence - MCF10A

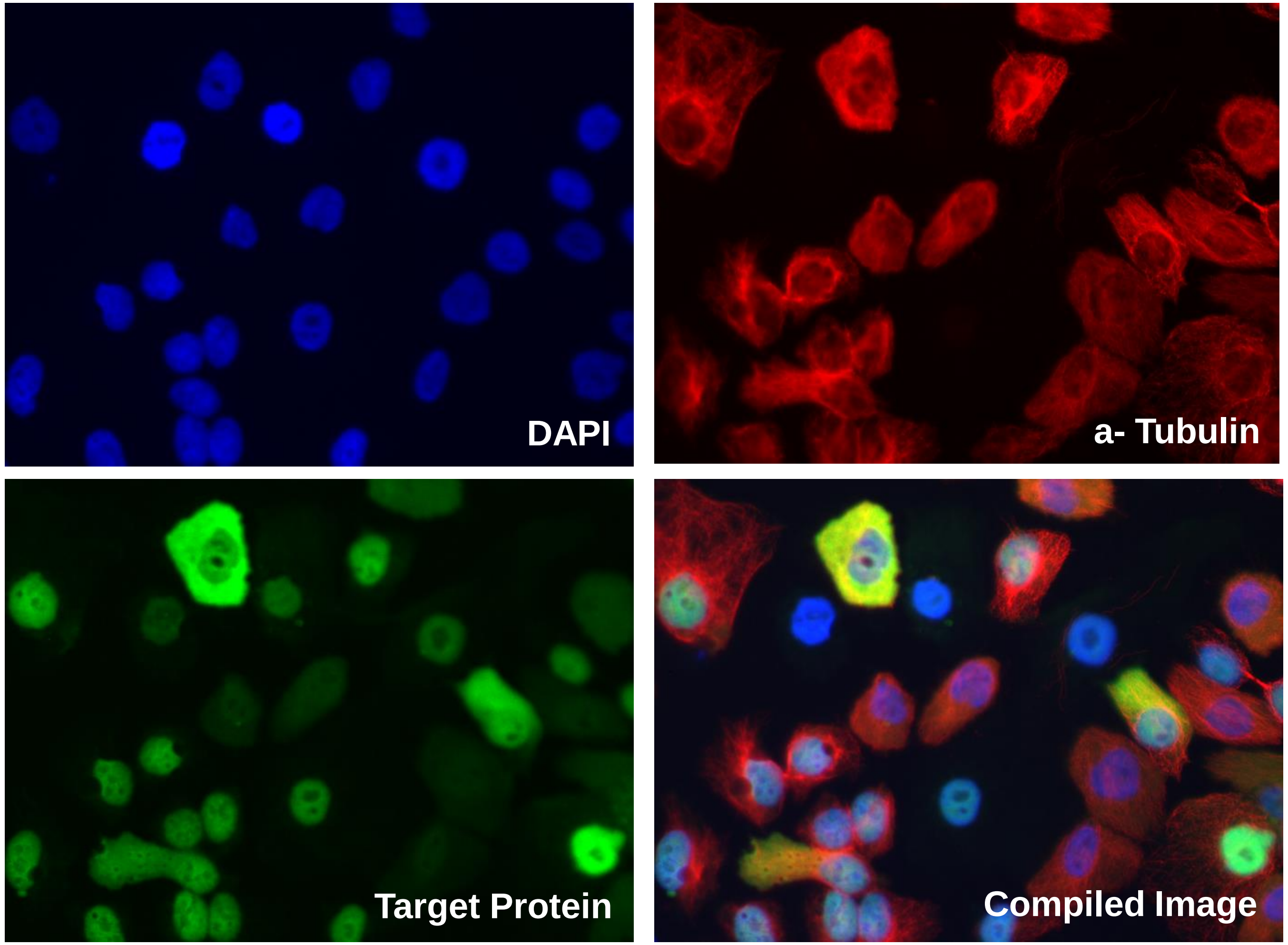

Subcelluar Locations: Cytosol, Cytoskeleton, Nucleus, Other

Immunofluorescence - LCL57

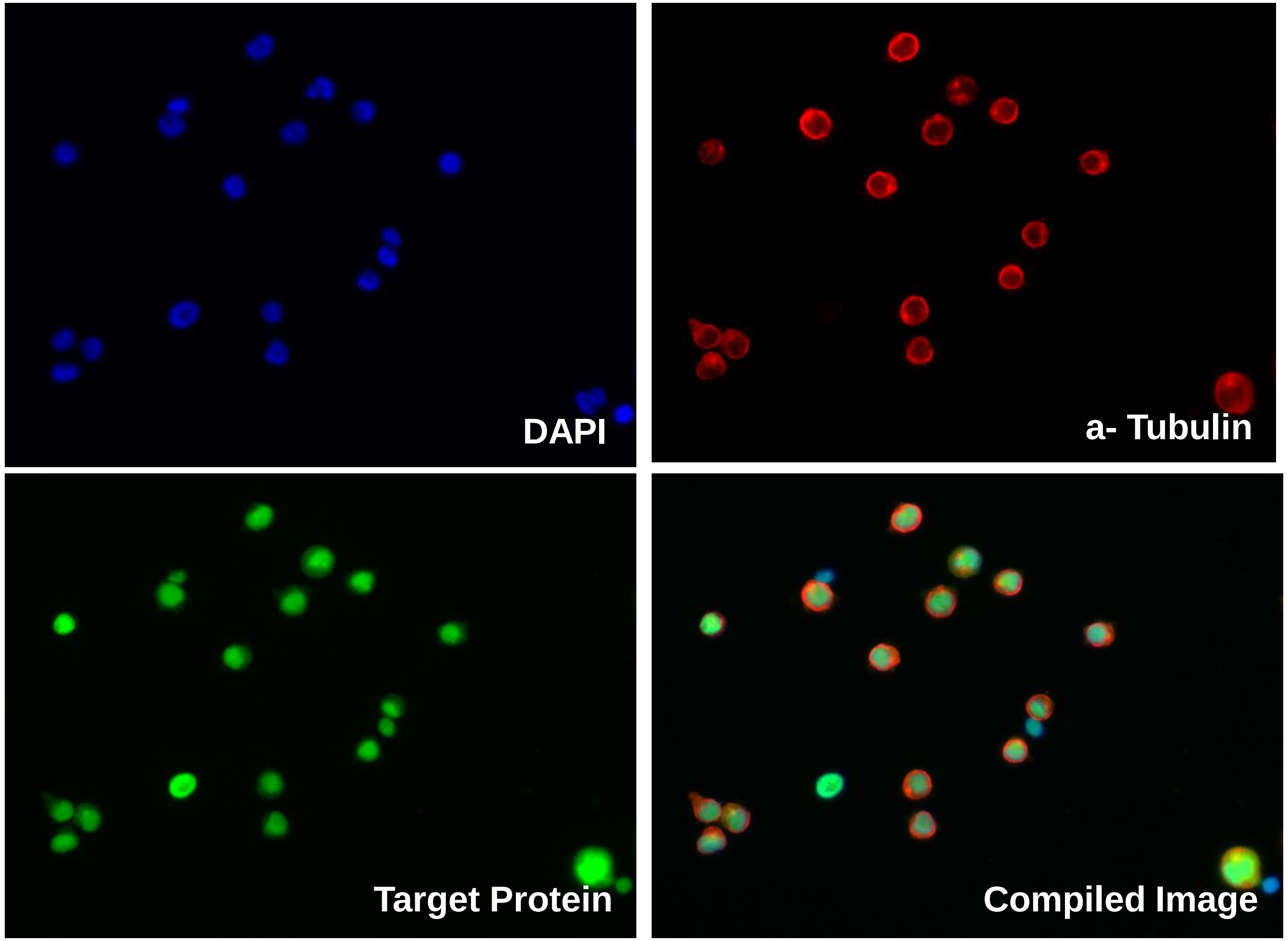

Subcelluar Locations: Cytosol, Cytoskeleton, Nucleus, Other

13. CPTC-KNL1-1

Technique	Analyte	Result
WB	RecProt	N*
West†	HeLa	N
	MCF10A	N
	LCL57	N
WB	HeLa	NT
	MCF10A	NT
	LCL57	NT
IF	HeLa	NT
	MCF10A	NT
	LCL57	NT
SCWB	HeLa	NT
	MCF10A	NT
	LCL57	NT
IP	RecProt	NT
IP	HeLa	NT
	MCF10A	NT
	LCL57	NT
IHC		N*
NCI60		NT

N – Negative, NT – Not Tested, * Data not shown
† Data shown are from Phospho-specificity Experiment.

Endogenous Wes

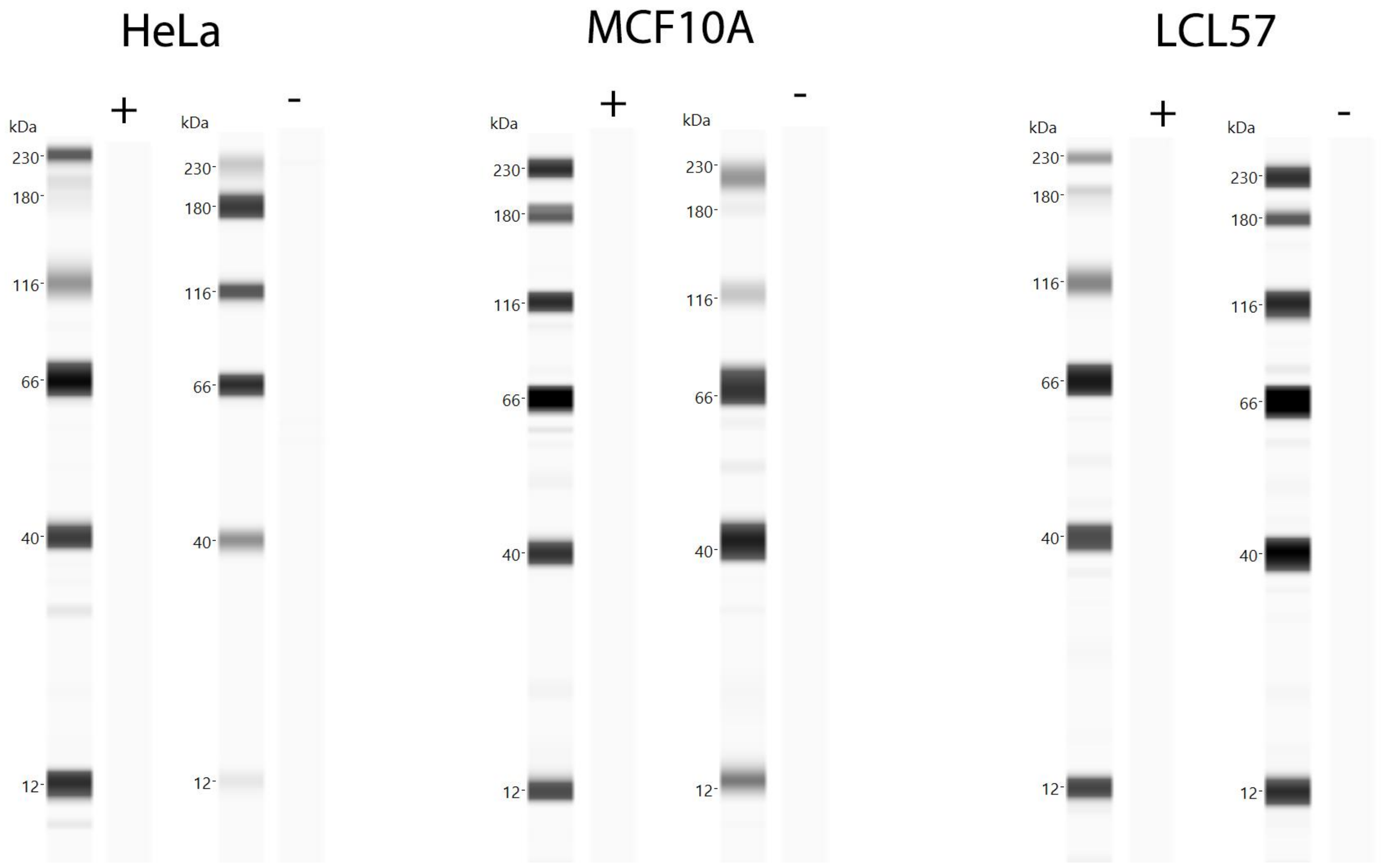

Expected molecular weight: 265, 263, 196, 206 kDa

+ : irradiation treated cells

- : not treated cells

14. CPTC-KRT Group A-1

Technique	Analyte	Result
WB	RecProt	P
Wes	HeLa	NT
	MCF10A	NT
	LCL57	NT
WB	HeLa	P
	MCF10A	P
	LCL57	N
IF	HeLa	P
	MCF10A	P
	LCL57	P
SCWB	HeLa	P
	MCF10A	P
	LCL57	N†
IP*	RecProt	P
IP*	HeLa	P
	MCF10A	W
	LCL57	W
IHC		N†
NCI60		NT

P – Positive, N – Negative, NT – Not Tested, W – Weak, * Please refer to tables for results, † Data not shown

WB against commercial recombinant protein

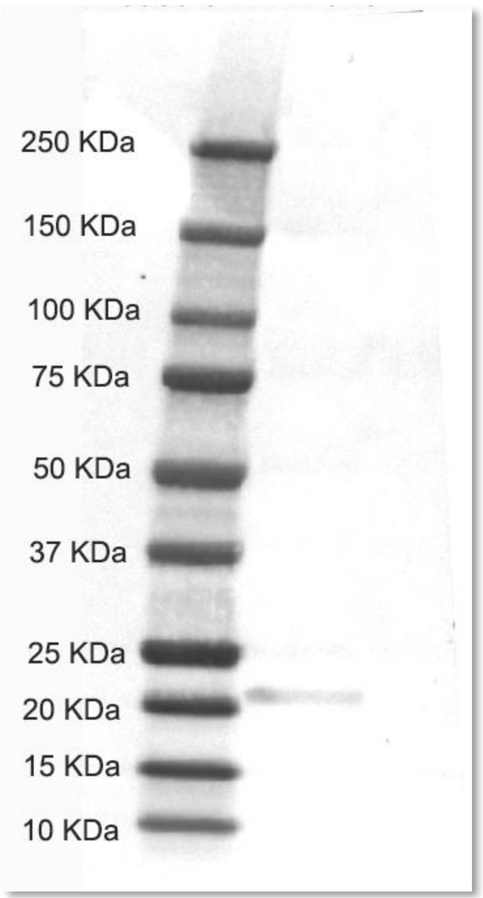

Ag Vendor: Origene

Cat #: TP309707

Description: Cytokeratin 19 (KRT19) (NM_002276) Human Recombinant Protein

Predicted MW: 44 KDa

Endogenous WB

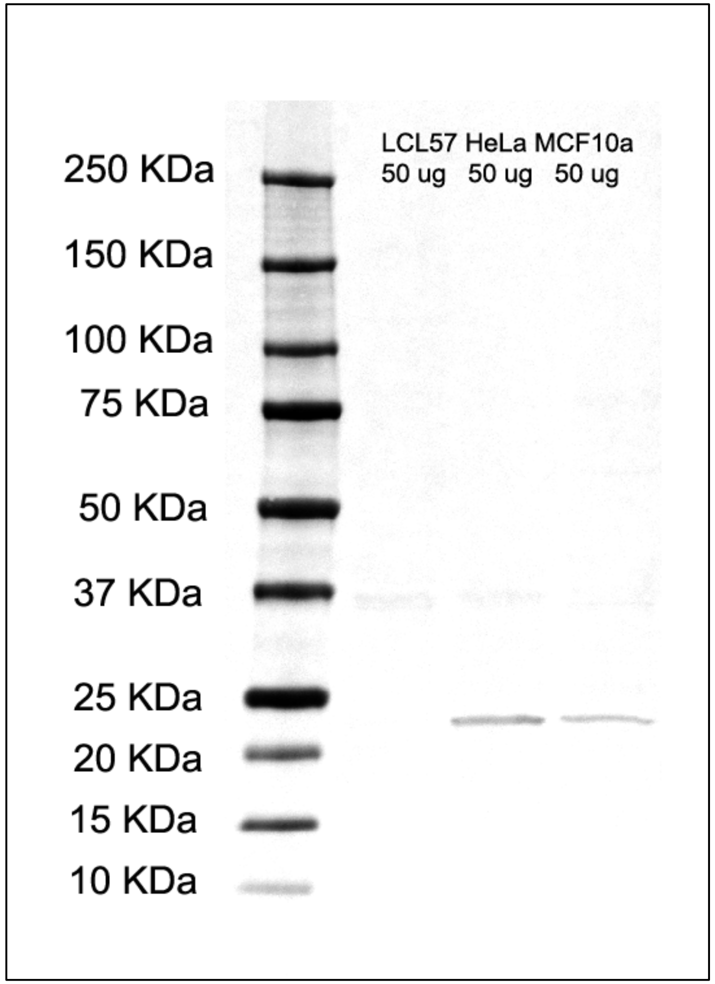

Immunofluorescence - HeLa

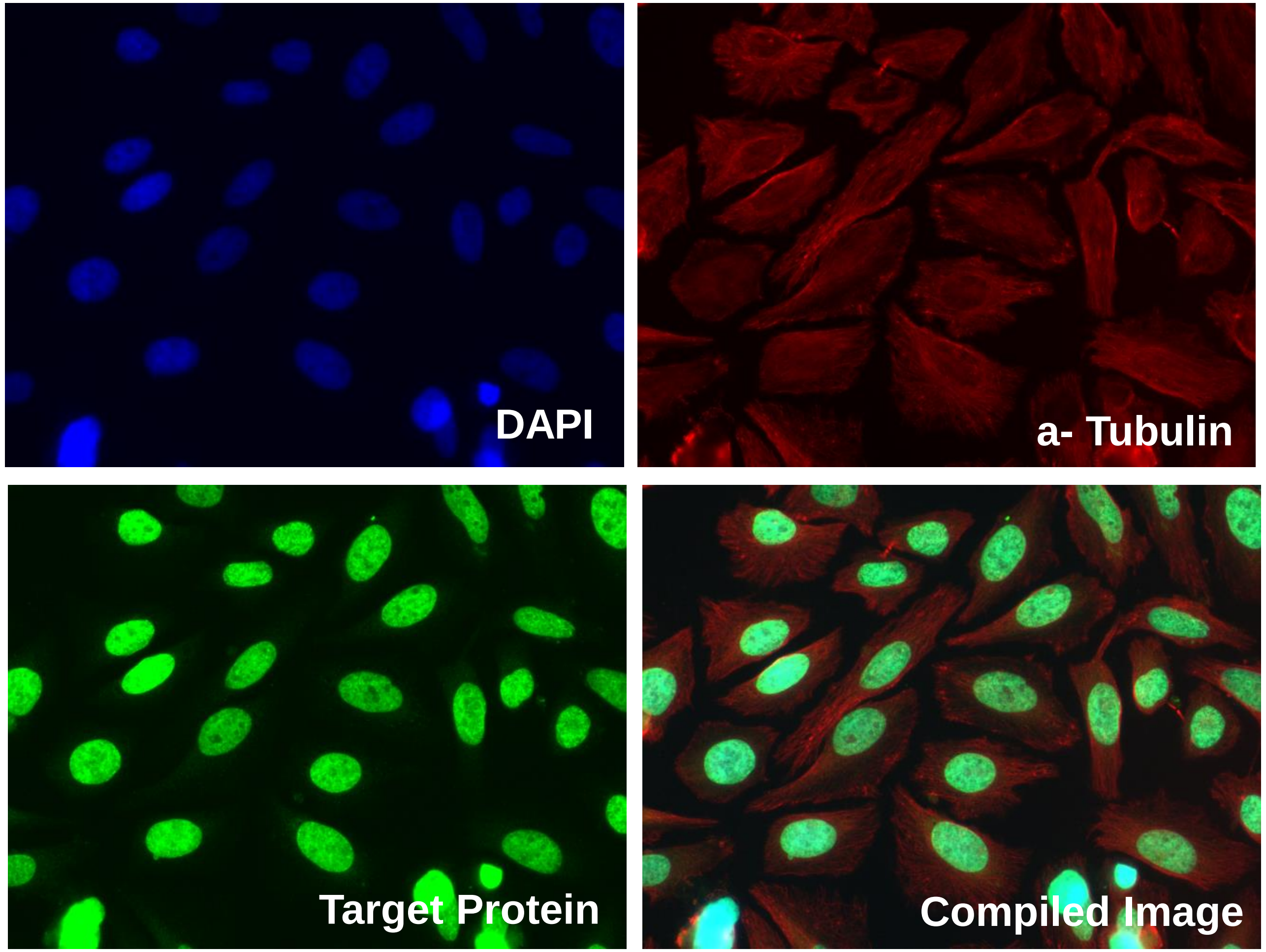

Subcellular Location: Nucleus, Other

Immunofluorescence - MCF10A

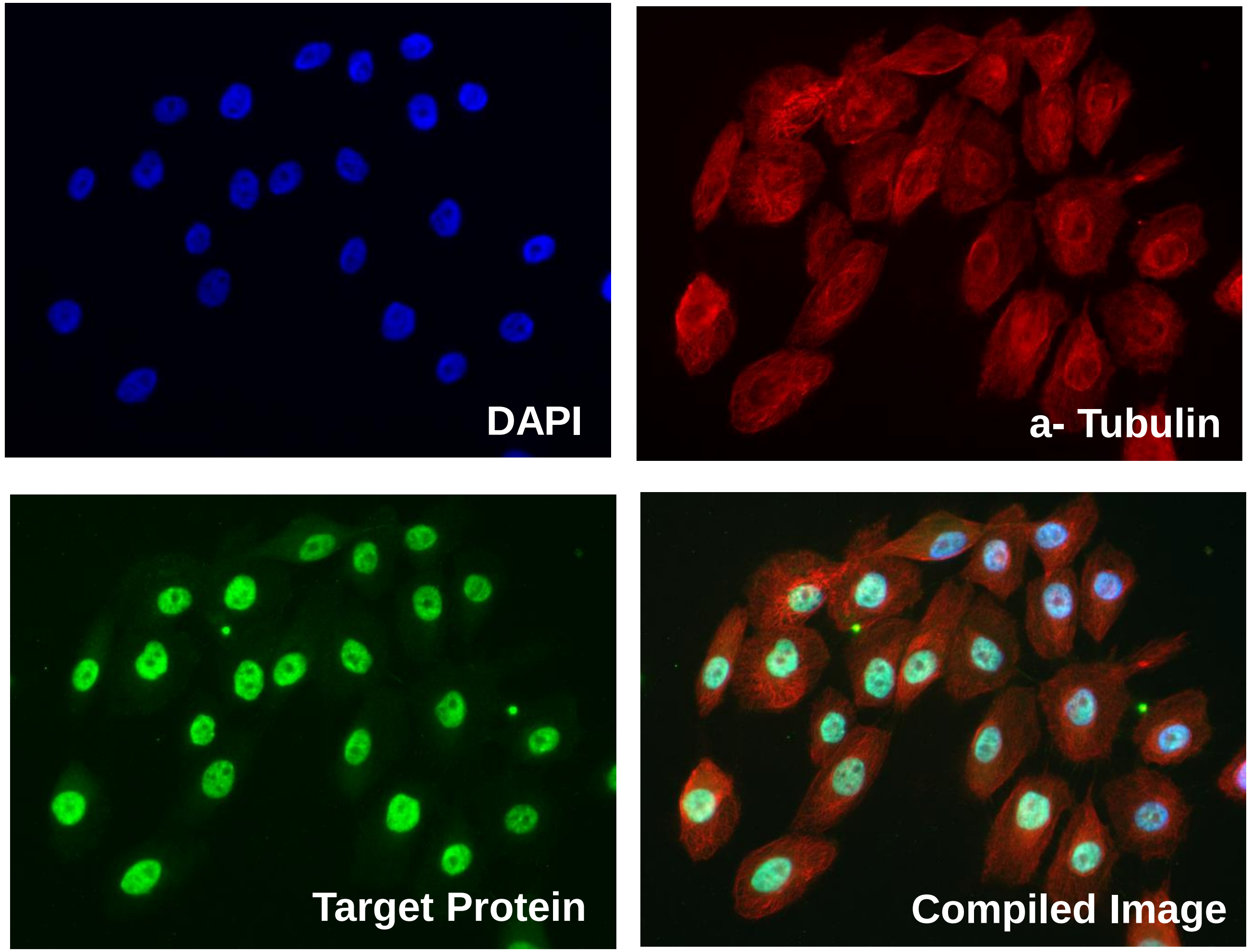

Subcellular Location: Nucleus, Other

Immunofluorescence - LCL57

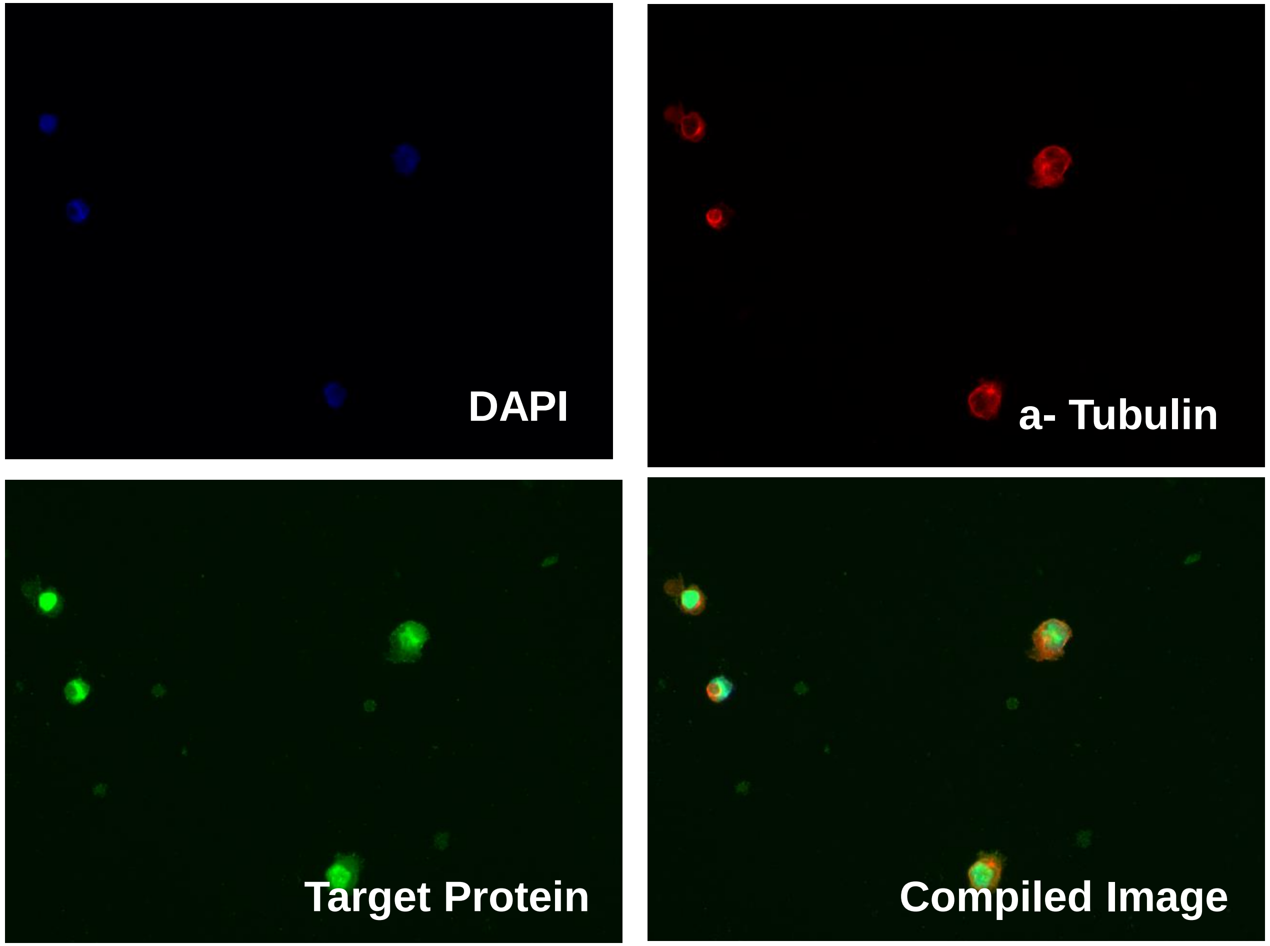

Subcellular Location: Nucleus, Other

Single Cell Western Blot

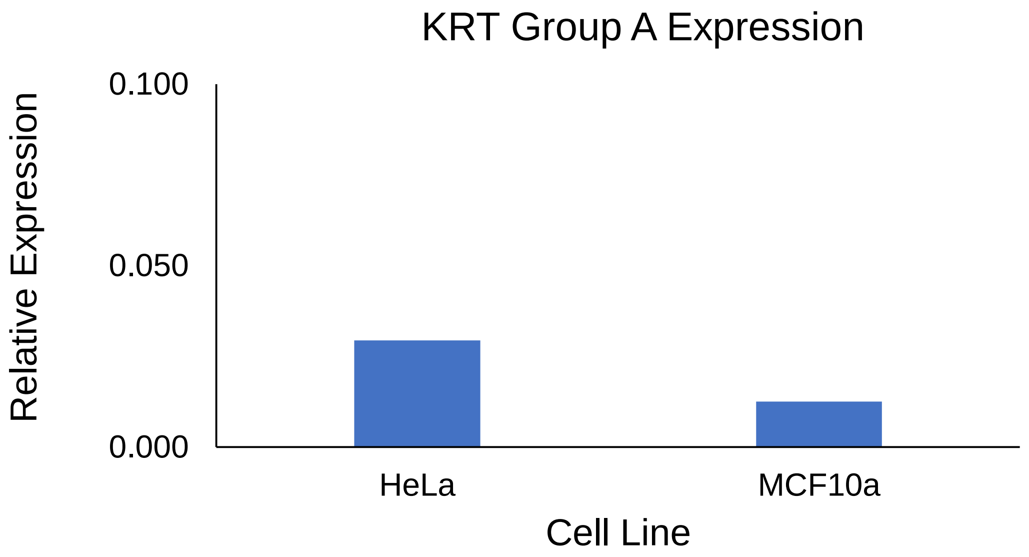

A.

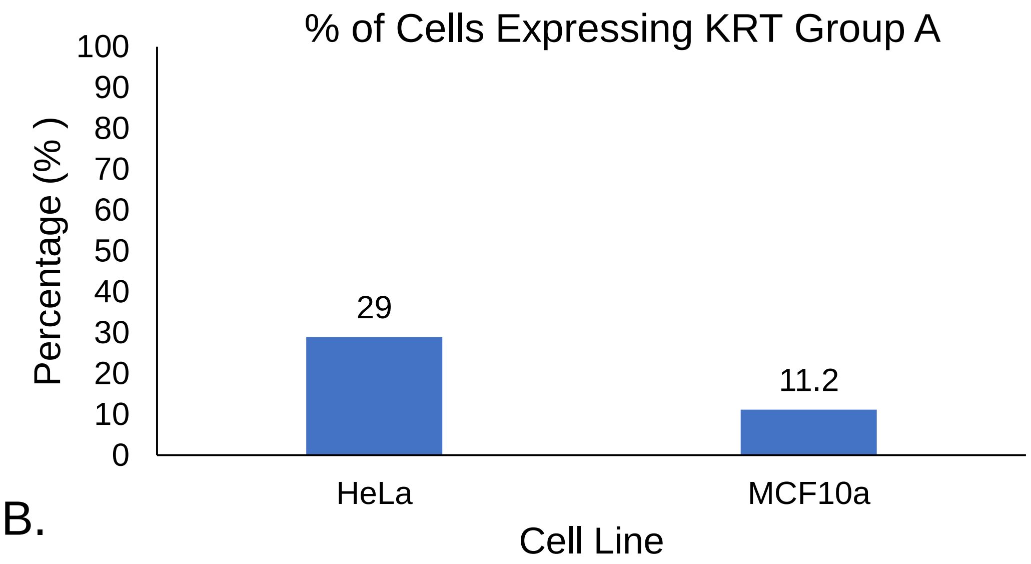

B.

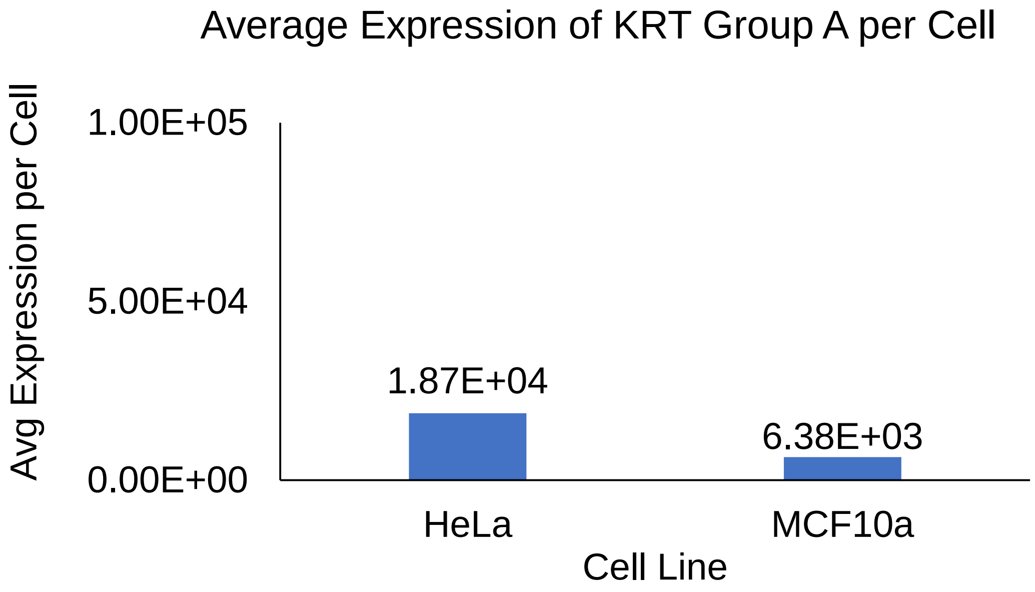

C.

Single cell western blot data for KRT Group A (44 kDa band). Relative expression of total KRT Group A (44 kDa) in HeLa, MCF10A, and LCL57 cells (A). Percentage of cells that express KRT Group A (B). Average expression of KRT Group A protein per cell (C). All data is normalized to β-tubulin expression.

15. CPTC-KRT Group B-1

Technique	Analyte	Result
WB	RecProt	P
Wes	HeLa	P
	MCF10A	P
	LCL57	N
WB	HeLa	P
	MCF10A	P
	LCL57	N
IF	HeLa	P
	MCF10A	P
	LCL57	N†
SCWB	HeLa	P
	MCF10A	P
	LCL57	N†
IP*	RecProt	P
IP*	HeLa	P
	MCF10A	N
	LCL57	N
IHC		P
NCI60		P

P – Positive, N – Negative, * Please refer to tables for results, † Data not shown

WB against commercial recombinant protein

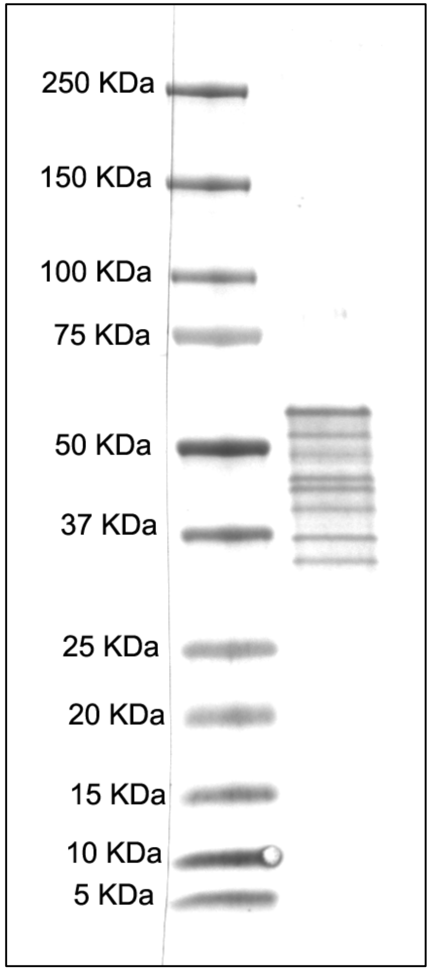

Ag Vendor: Origene

Cat #: TP309570

Description: Recombinant protein of human keratin 8 (KRT8)

Predicted MW 53.7 KDa

Endogenous WB

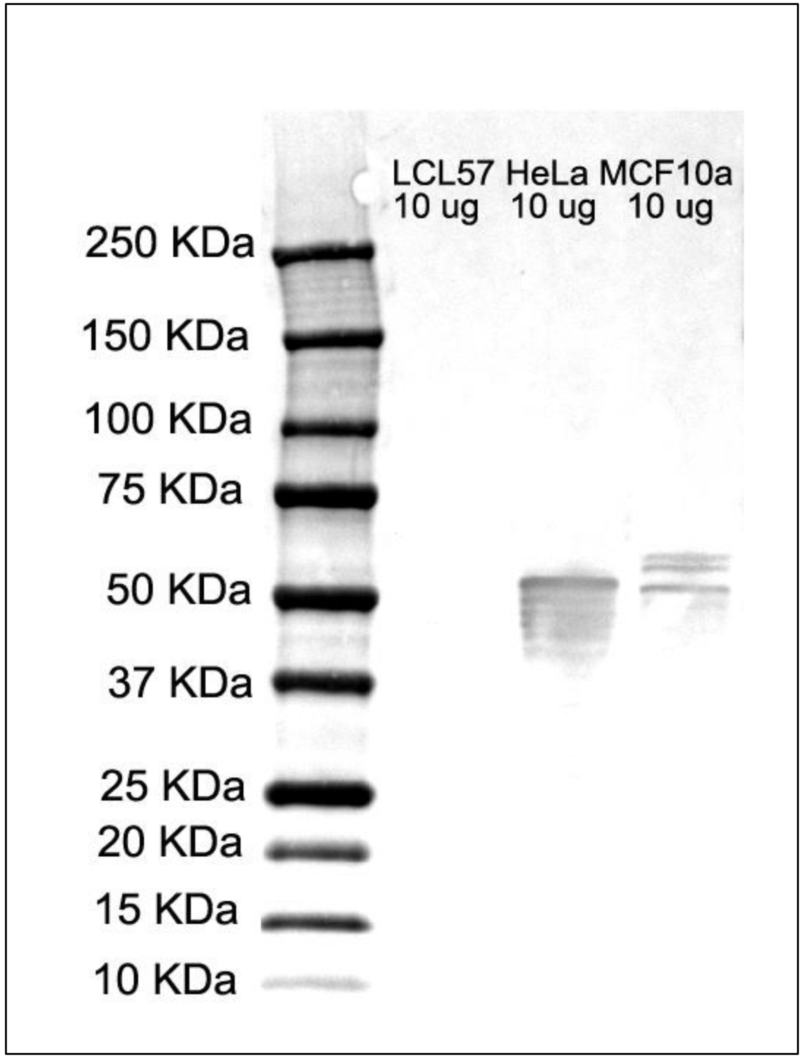

Endogenous Wes

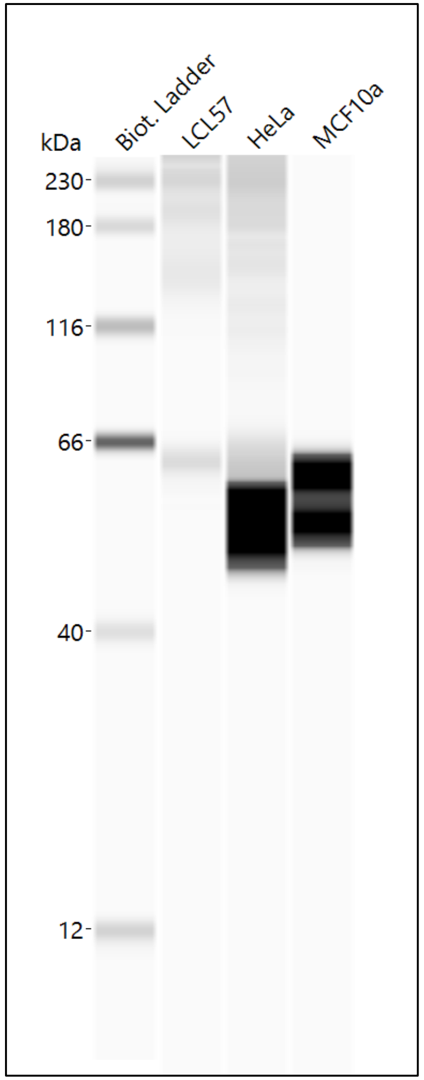

Immunofluorescence - HeLa

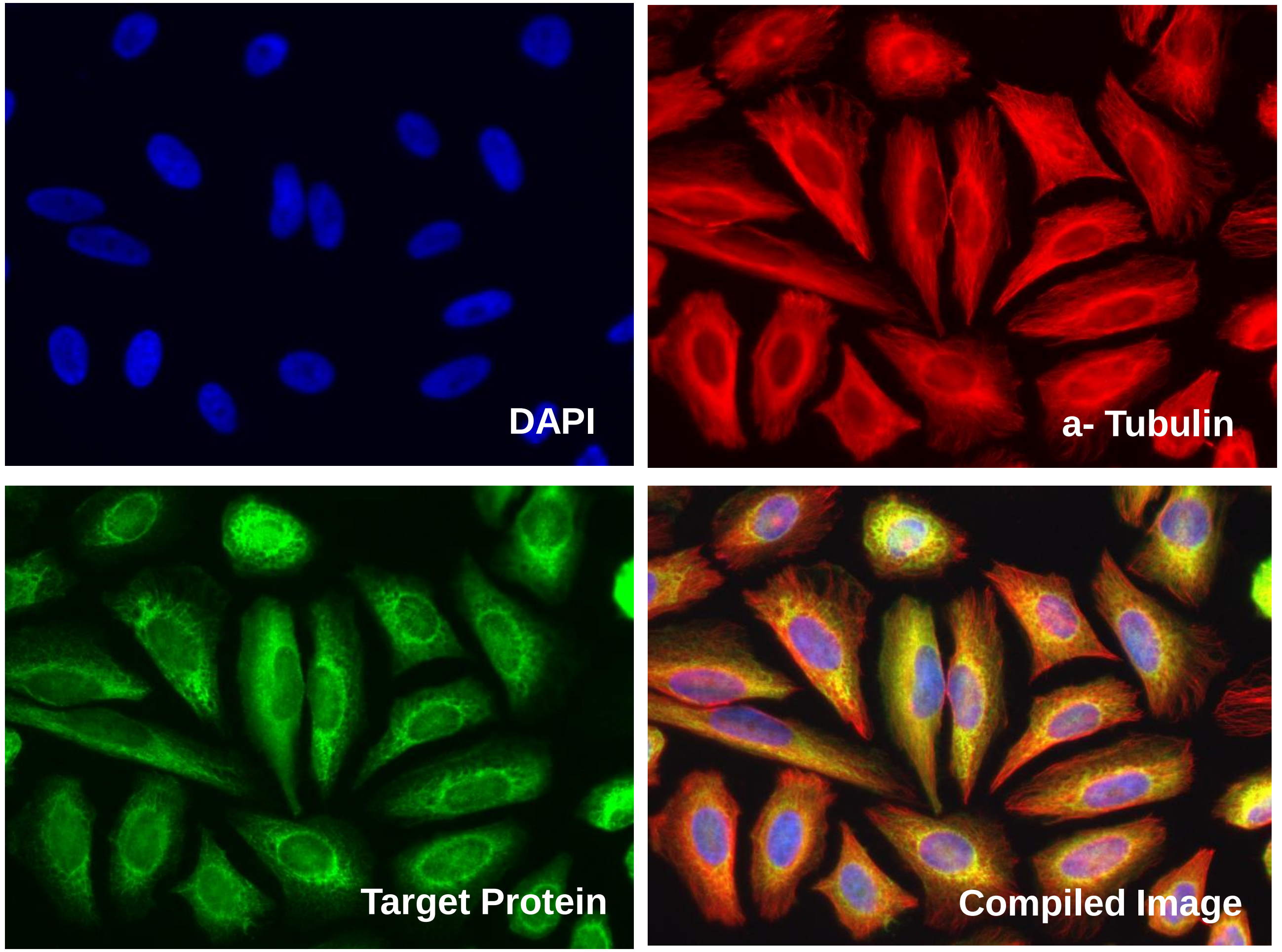

Subcelluar Locations: Cytoskeleton, Cytosol, Extracellular region or secreted, Other

Immunofluorescence - MCF10A

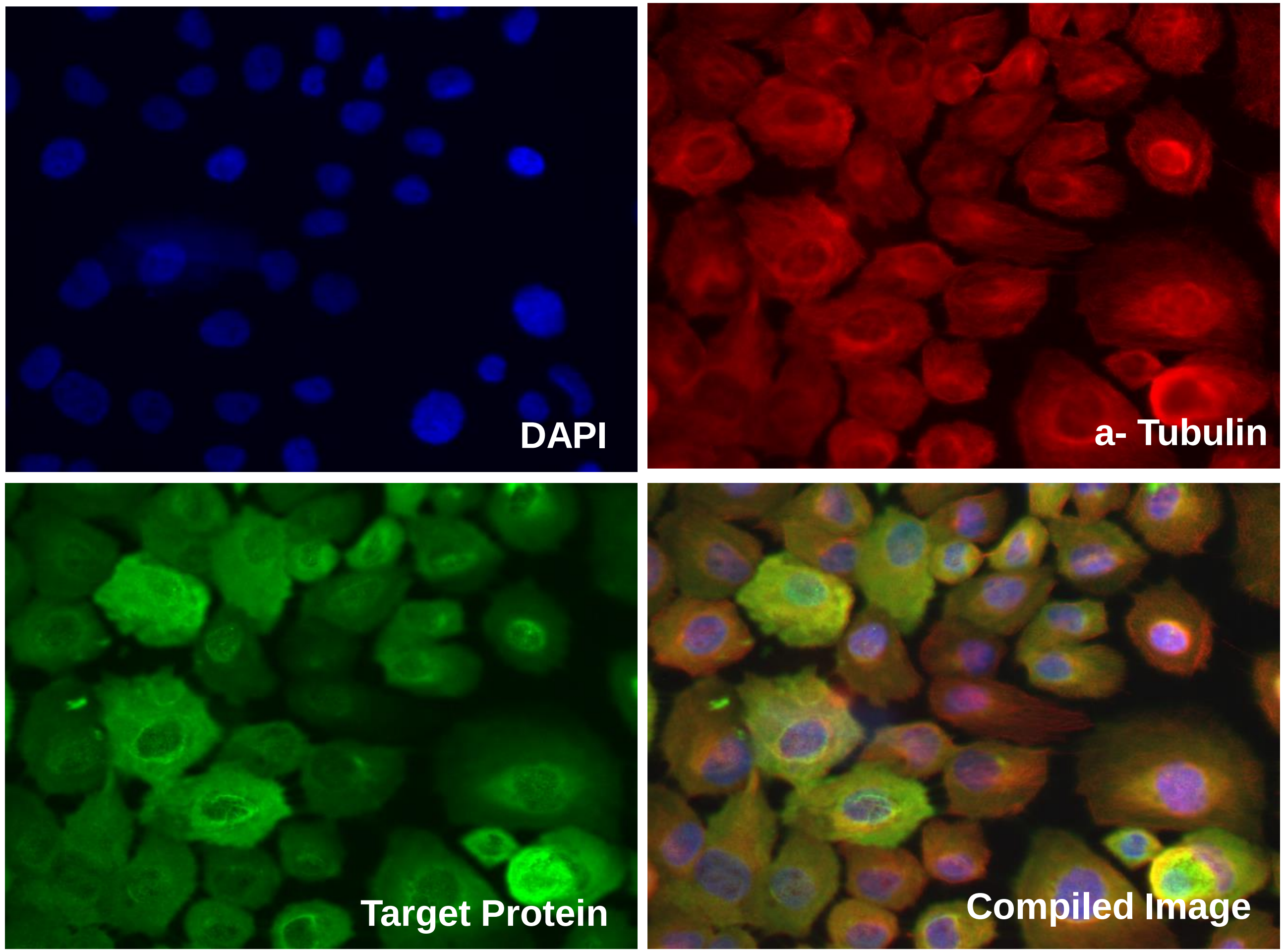

Subcelluar Locations: Cytoskeleton, Cytosol, Extracellular region or secreted, Other

CPTC- KRT Group B-1

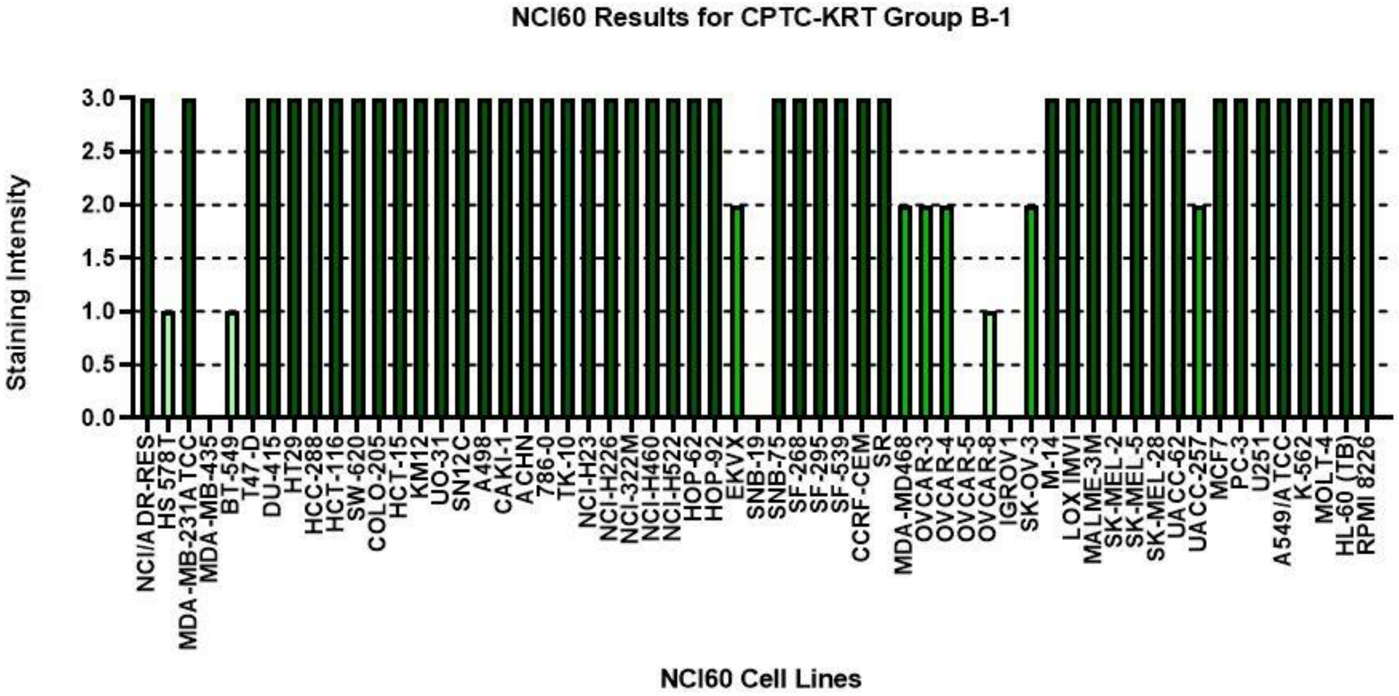

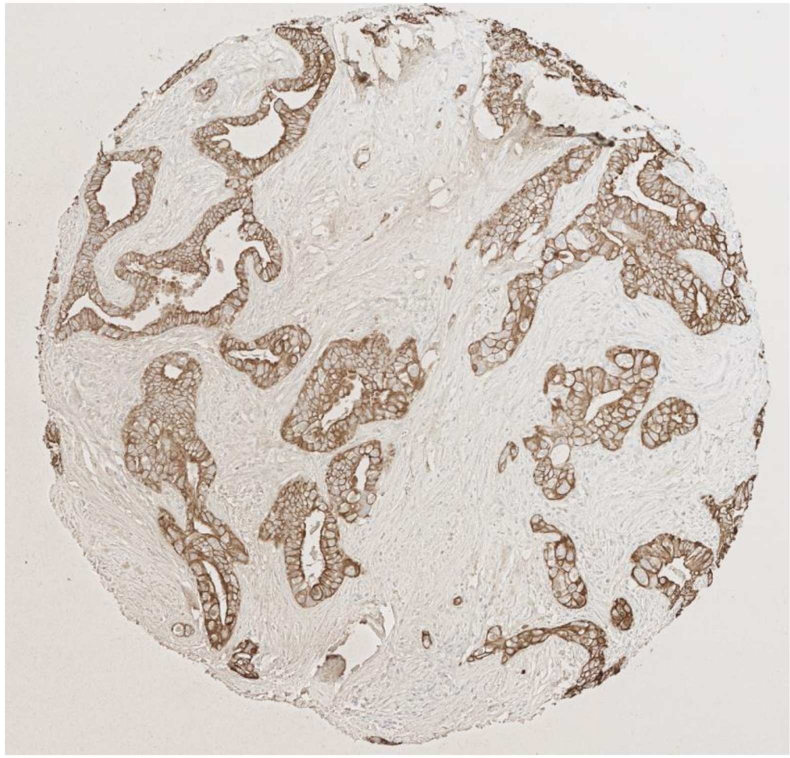

16. CPTC-LAT-1

Technique	Analyte	Result
WB	RecProt	P
Wes	HeLa	N
	MCF10A	N
	LCL57	N
WB	HeLa	P
	MCF10A	P
	LCL57	P
IF†	HeLa	N
	MCF10A	N
	LCL57	N
SCWB†	HeLa	N
	MCF10A	N
	LCL57	N
IP*	RecProt	W
IP*	HeLa	N
	MCF10A	N
	LCL57	N
IHC		P
NCI60		P

P – Positive, N – Negative, W – Weak, * Please refer to tables for results, † Data not shown

WB against commercial recombinant protein

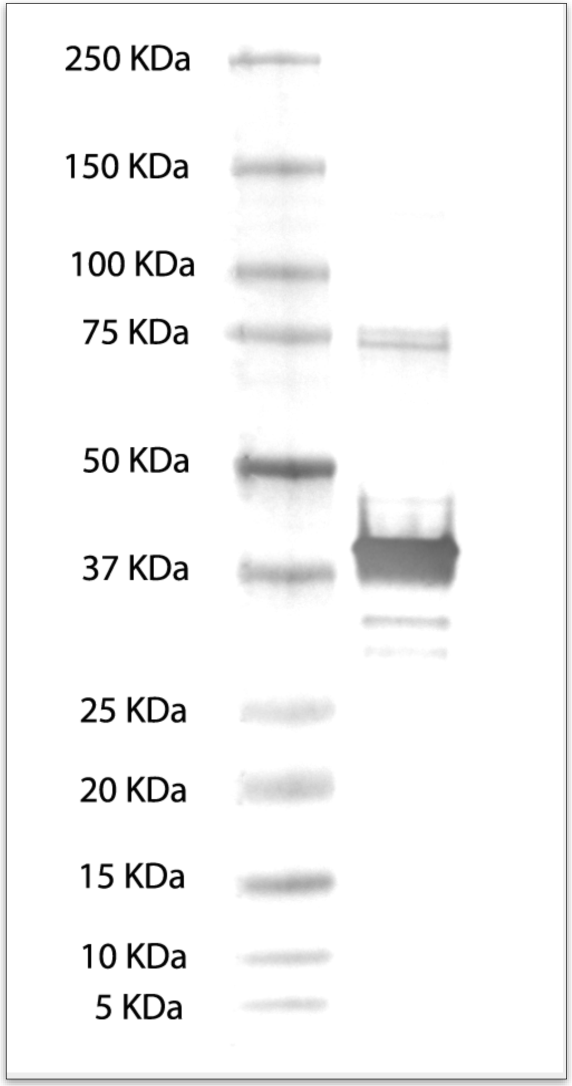

Ag Vendor: Novus Biologicals

Cat #: NBP1-72479

Description: Recombinant Human LAT Protein

Predicted MW 24.4 KDa

Endogenous WB

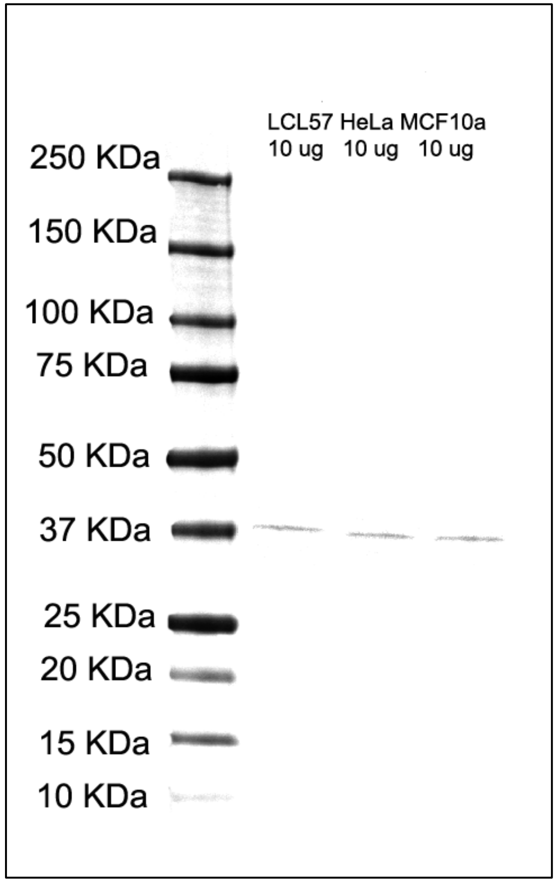

Endogenous Wes

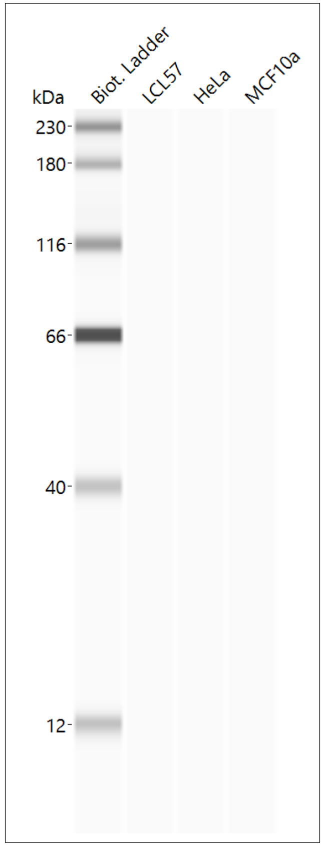

CPTC-LAT-1

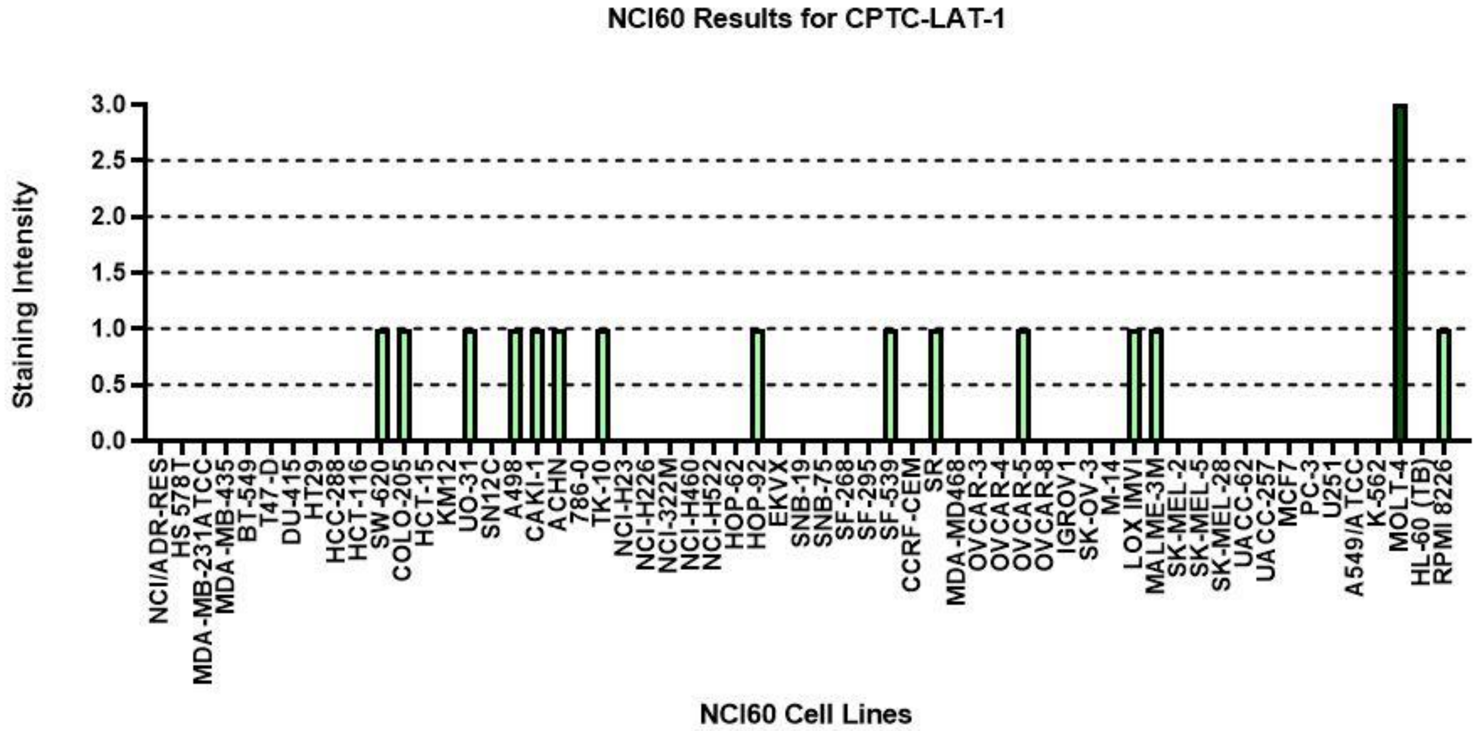

NCI60 Results for CPTC-LAT-1
Staining Intensity
3.0
2.5
2.0
1.5
1.0
0.5
0.0
NCI/ADR-RES
HS 578T
MDA-MB-231ATCC
MDA-MB-435
BT-549
T47-D
DU-415
HT29
HCC-288
HCT-116
SW-620
COLO-205
HCT-15
KM12
UO-31
SN12C
A498
CAKI-1
ACHN
786-0
TK-10
NCI-H23
NCI-H226
NCI-322M
NCI-H460
NCI-H522
HOP-62
HOP-92
EKVX
SNB-19
SNB-75
SF-268
SF-295
SF-539
CCRF-CEM
SR
MDA-MD468
OVCAR-3
OVCAR-4
OVCAR-5
OVCAR-8
IGROV1
SK-OV-3
M-14
LOX IMVI
MALME-3M
SK-MEL-2
SK-MEL-5
SK-MEL-28
UACC-62
UACC-257
MCF7
PC-3
U251
A549/ATCC
K-562
MOLT-4
HL-60 (TB)
RPMI 8226
NCI60 Cell Lines

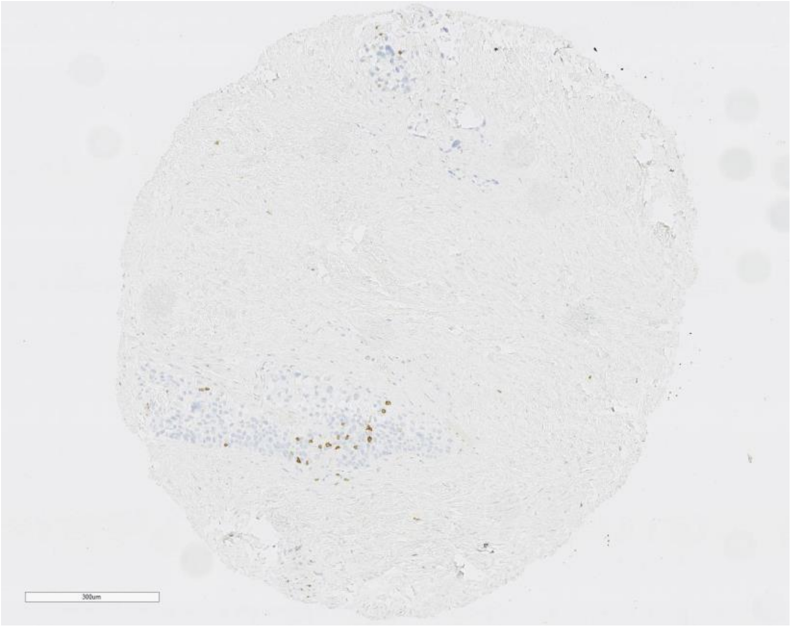

17. CPTC-LIME1-1

Technique	Analyte	Result
WB	RecProt	**N***
Wes†	HeLa	**N**
	MCF10A	**N**
	LCL57	**N**
WB	HeLa	**NT**
	MCF10A	**NT**
	LCL57	**NT**
IF	HeLa	**NT**
	MCF10A	**NT**
	LCL57	**NT**
SCWB	HeLa	**NT**
	MCF10A	**NT**
	LCL57	**NT**
IP	RecProt	**NT**
IP	HeLa	**NT**
	MCF10A	**NT**
	LCL57	**NT**
IHC		**N***
NCI60		**NT**

N – Negative, NT – Not Tested, * Data not shown
† Data shown are from Phospho-specificity Experiment.

Endogenous Wes

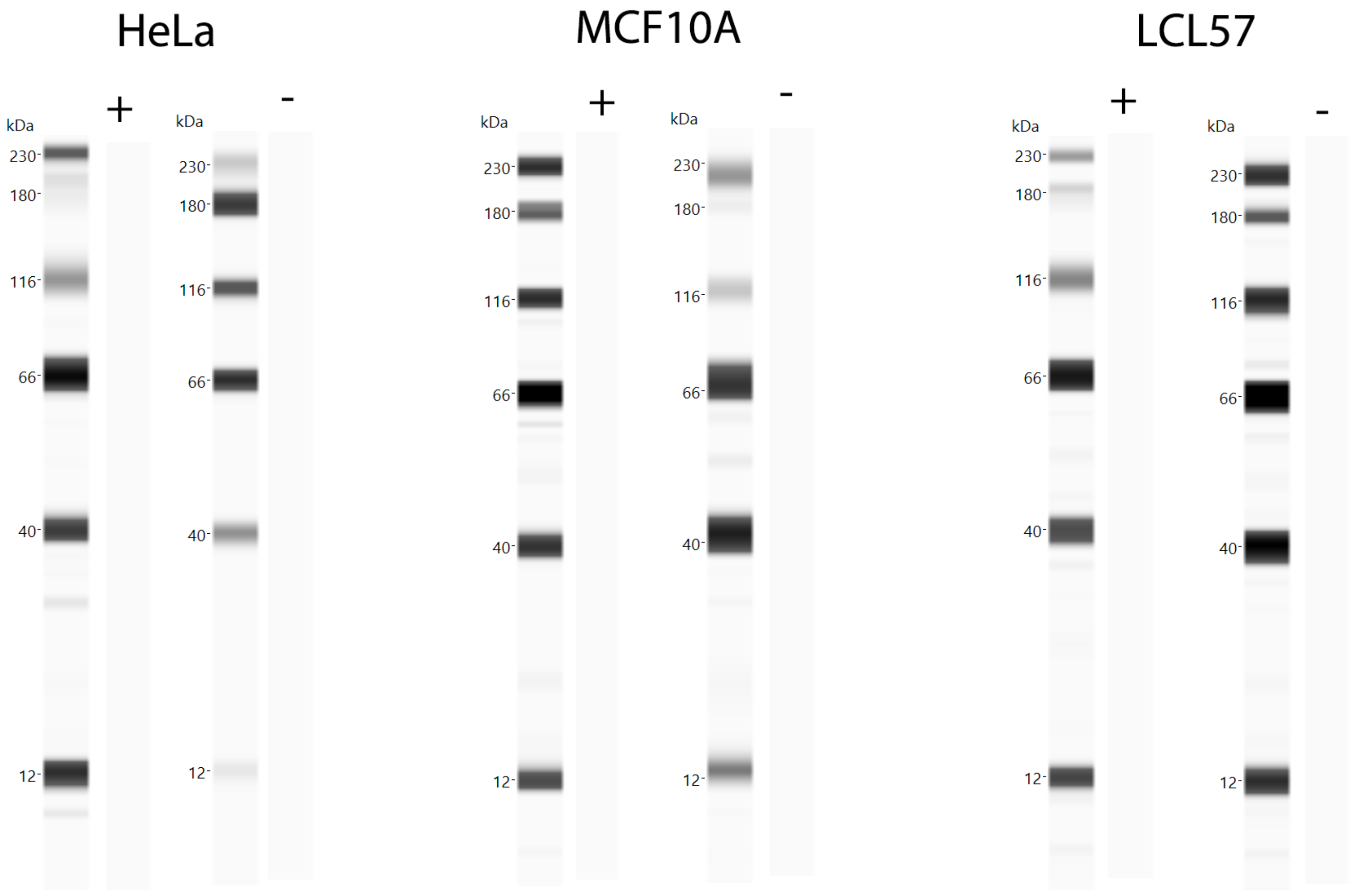

Expected molecular weight: 31, 21 kDa

+ : irradiation treated cells
- : not treated cells

18. CPTC-LMNB1-1

Technique	Analyte	Result
WB	RecProt	N*
Wes†	HeLa	N
	MCF10A	N
	LCL57	N
WB	HeLa	NT
	MCF10A	NT
	LCL57	NT
IF	HeLa	NT
	MCF10A	NT
	LCL57	NT
SCWB	HeLa	NT
	MCF10A	NT
	LCL57	NT
IP	RecProt	NT
IP	HeLa	NT
	MCF10A	NT
	LCL57	NT
IHC		P
NCI60		P

N – Negative, NT – Not Tested, * Data not shown
† Data shown are from Phospho-specificity Experiment.

Endogenous Wes

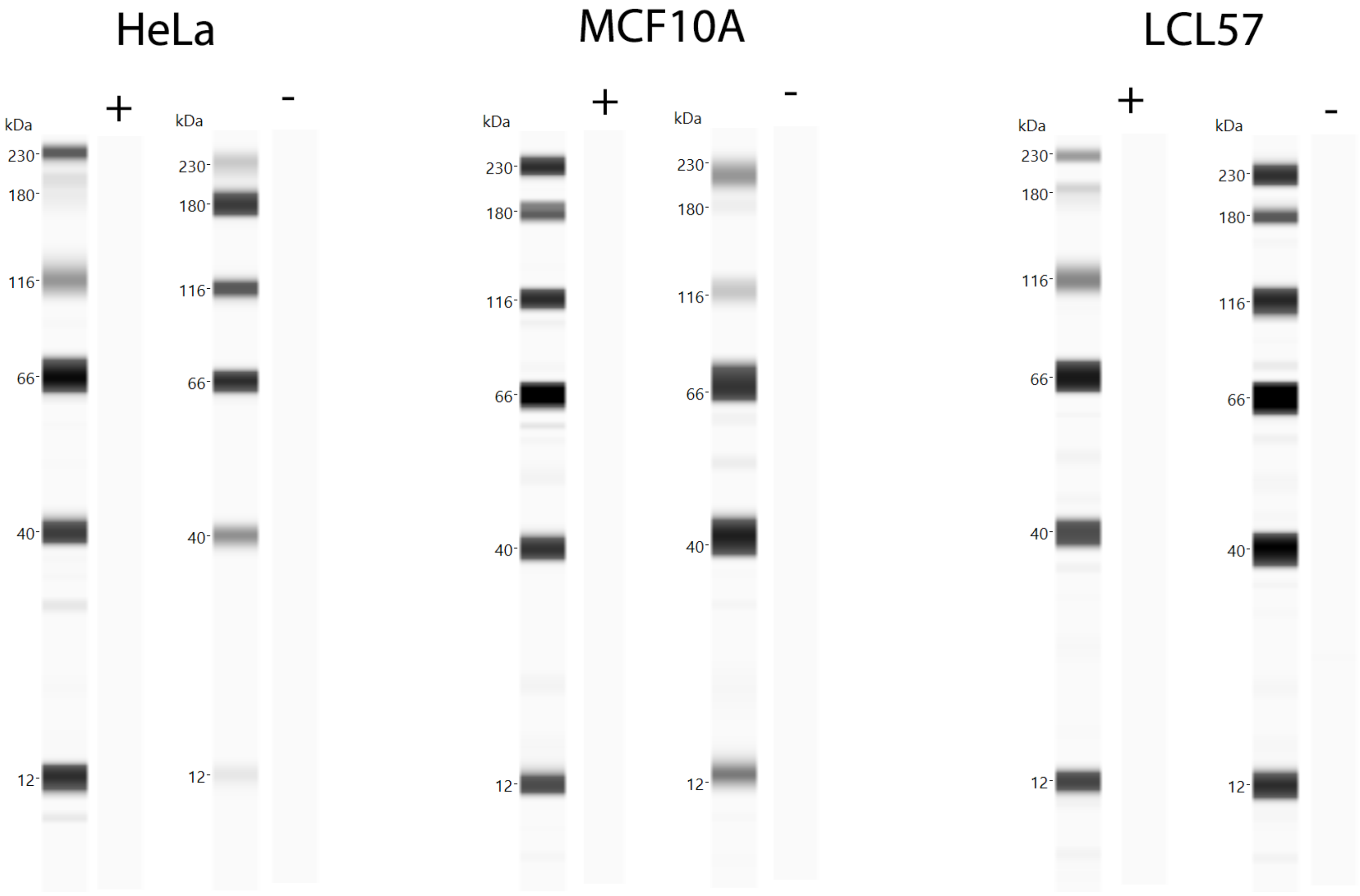

Expected molecular weight: 66 kDa

+ : irradiation treated cells
- : not treated cells

CPTC-LMNB1-1

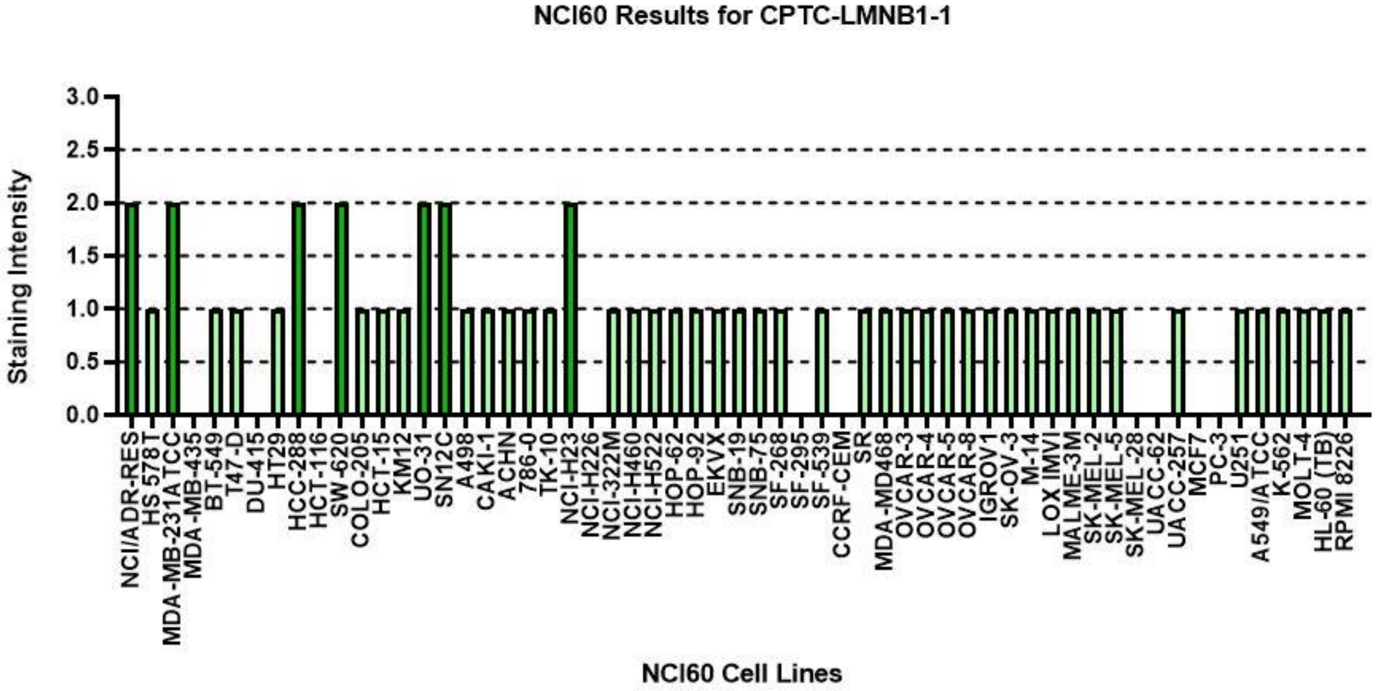
NCI60 Results for CPTC-LMNB1-1
Staining Intensity
3.0
2.5
2.0
1.5
1.0
0.5
0.0
NCI/ADR-RES
HS 578T
MDA-MB-231ATCC
MDA-MB-435
BT-549
T47-D
DU-415
HT29
HCC-288
HCT-116
SW-620
COLO-205
HCT-15
KM12
UO-31
SN12C
A498
CAKI-1
ACHN
786-0
TK-10
NCI-H23
NCI-H226
NCI-322M
NCI-H460
NCI-H522
HOP-62
HOP-92
EKVX
SNB-19
SNB-75
SF-268
SF-295
SF-539
CCRF-CEM
SR
MDA-MD468
OVCAR-3
OVCAR-4
OVCAR-5
OVCAR-8
IGROV1
SK-OV-3
M-14
LOX IMVI
MALME-3M
SK-MEL-2
SK-MEL-5
SK-MEL-28
UACC-62
UACC-257
MCF7
PC-3
U251
A549/ATCC
K-562
MOLT-4
HL-60 (TB)
RPMI 8226
NCI60 Cell Lines

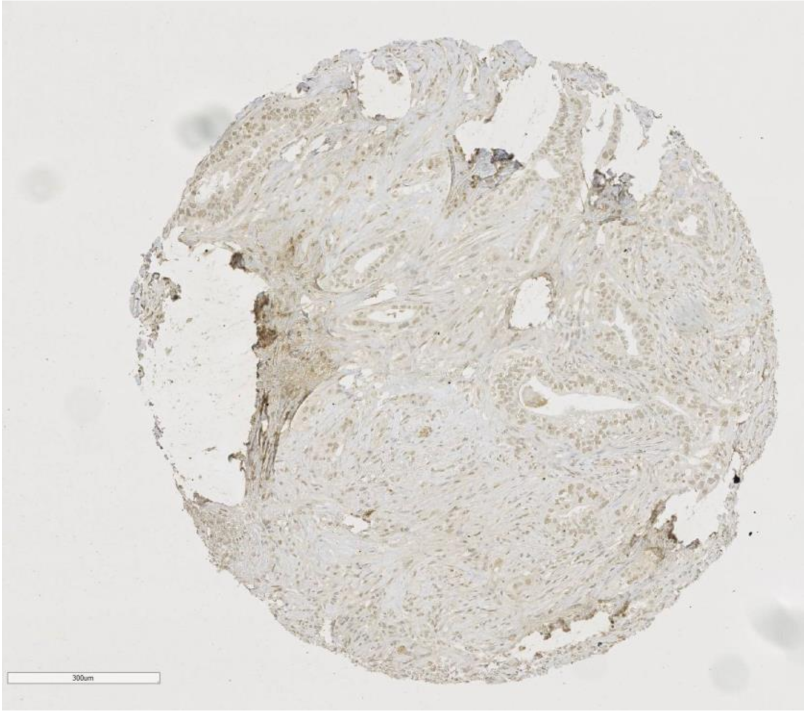

19. CPTC-LMNB1-2

Technique	Analyte	Result
WB	RecProt	P
Wes	HeLa	N
	MCF10A	N
	LCL57	N
WB	HeLa	P
	MCF10A	P
	LCL57	P
IF	HeLa	P
	MCF10A	P
	LCL57	P
SCWB	HeLa	P
	MCF10A	P
	LCL57	P
IP*	RecProt	P
IP*	HeLa	N
	MCF10A	N
	LCL57	N
IHC		N†
NCI60		NT

P – Positive, N – Negative, *Please refer to tables for results, † Data not shown

WB against commercial recombinant protein

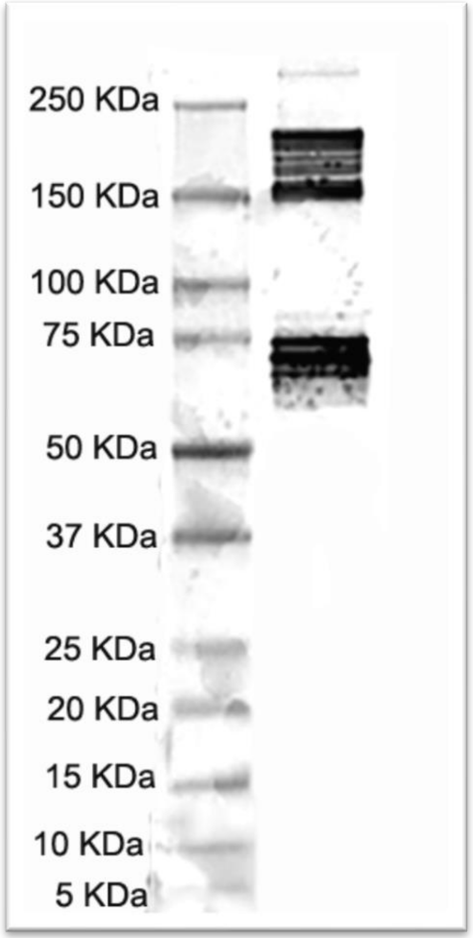

Ag Vendor: Origene

Cat #: TP301604

Description: Lamin B1 (LMNB1) (NM_005573) Human Recombinant Protein

Predicted MW 66.2 KDa

Endogenous WB

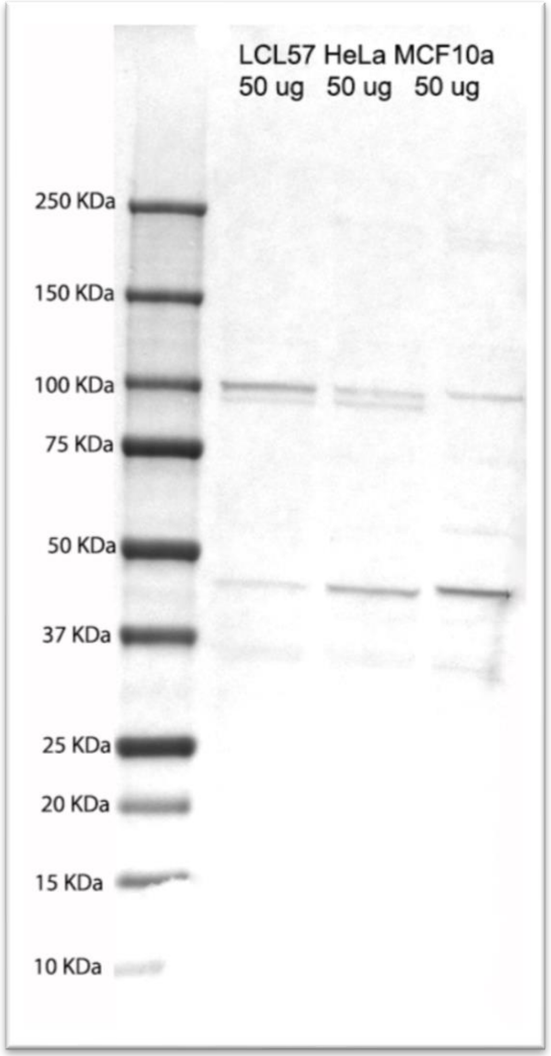

Endogenous Wes

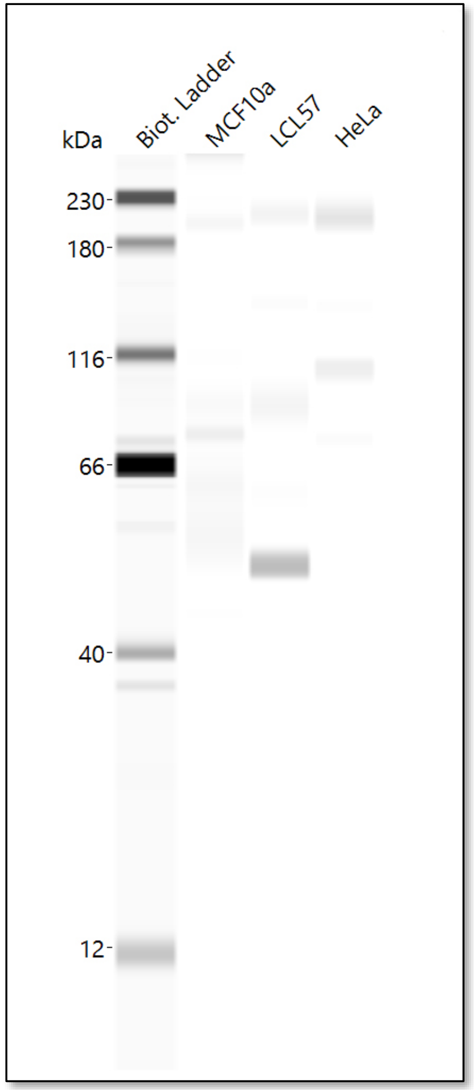

Immunofluorescence - HeLa

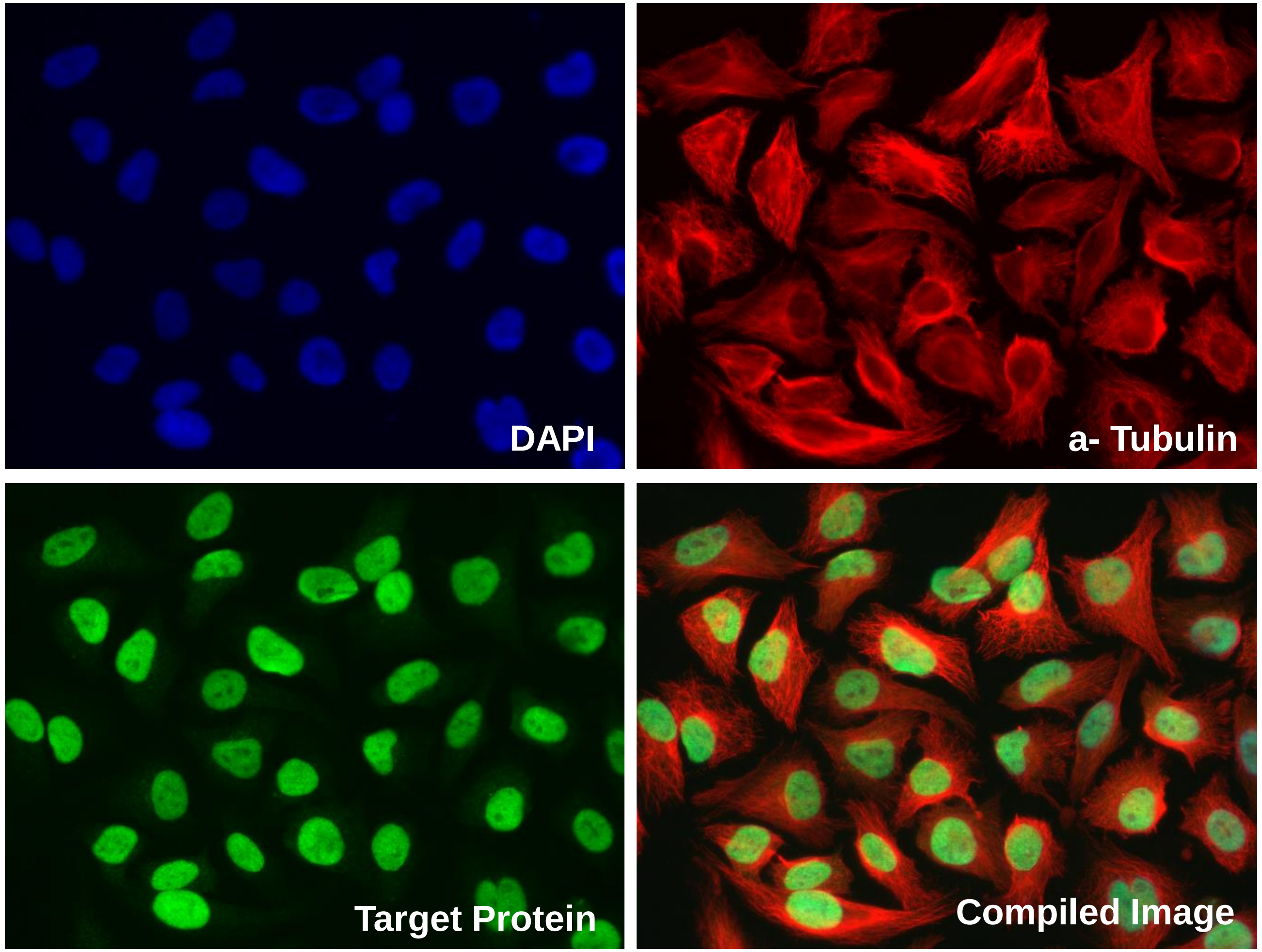

Subcellular location: Nucleus

Immunofluorescence – MCF10A

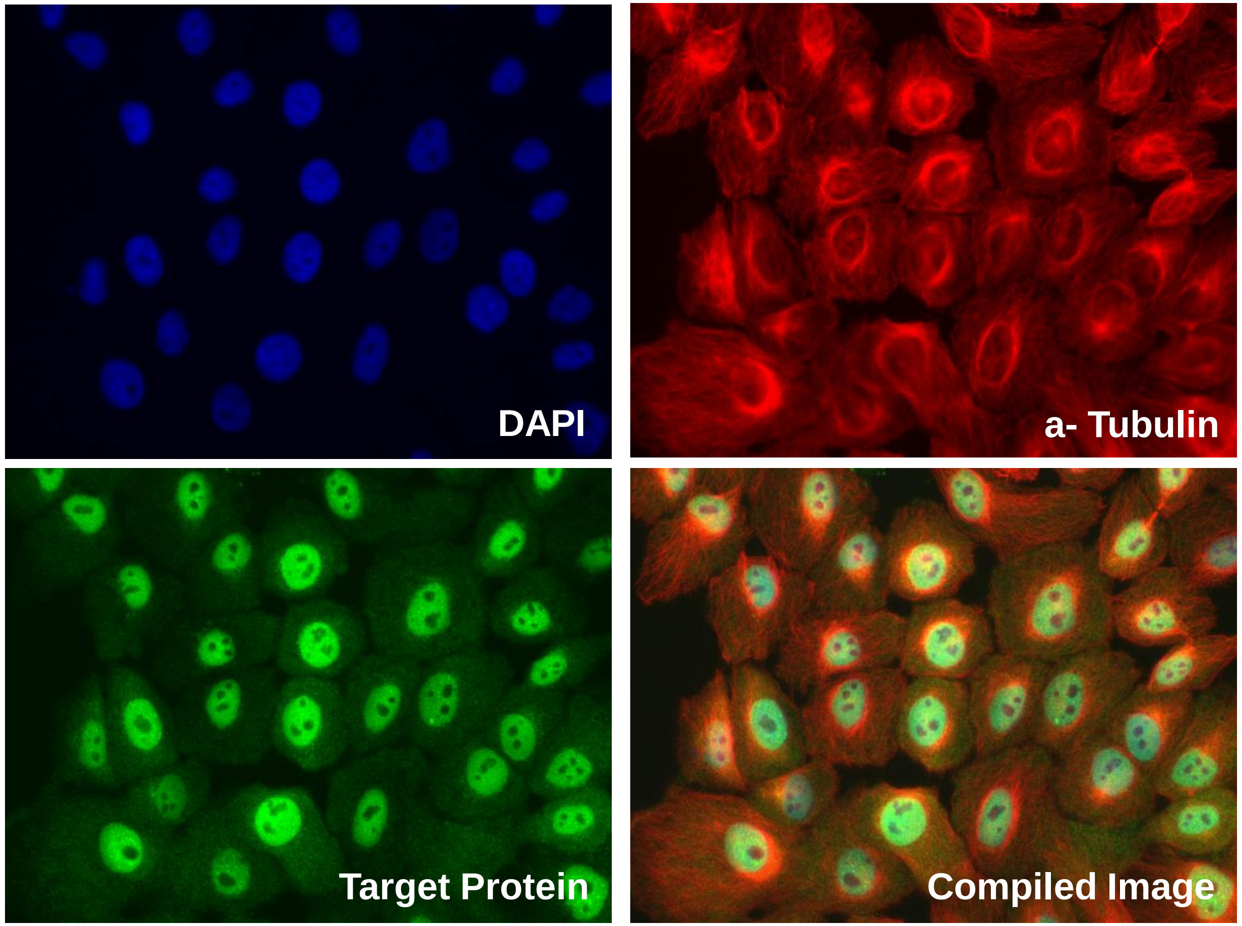

Subcellular location: Nucleus

Immunofluorescence – LCL57

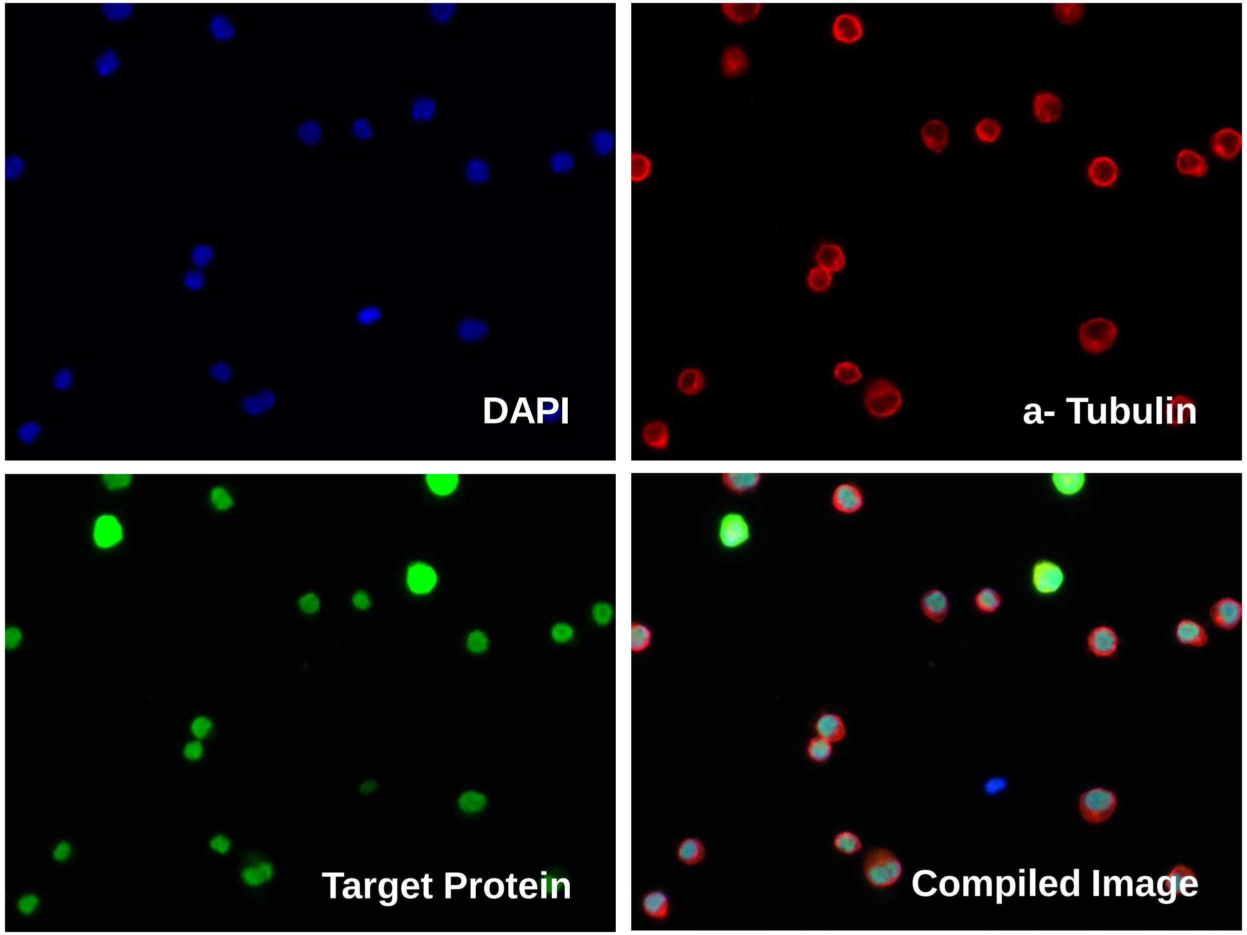

Subcellular location: Nucleus

Single Cell Western Blot

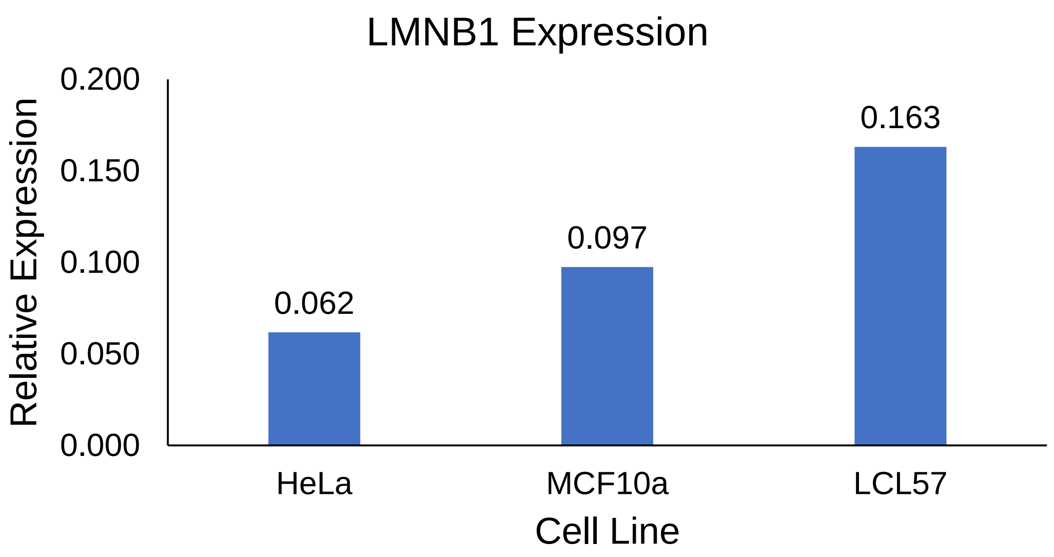

A.

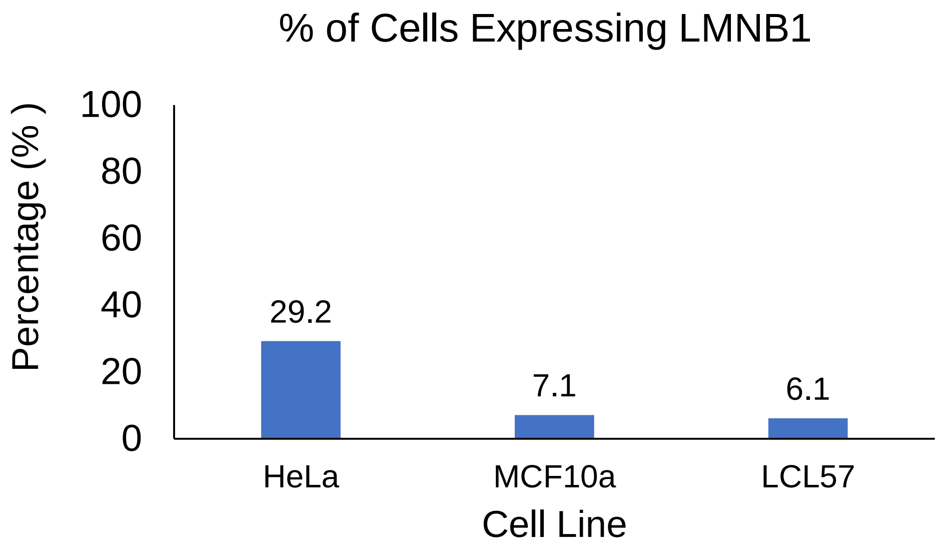

B.

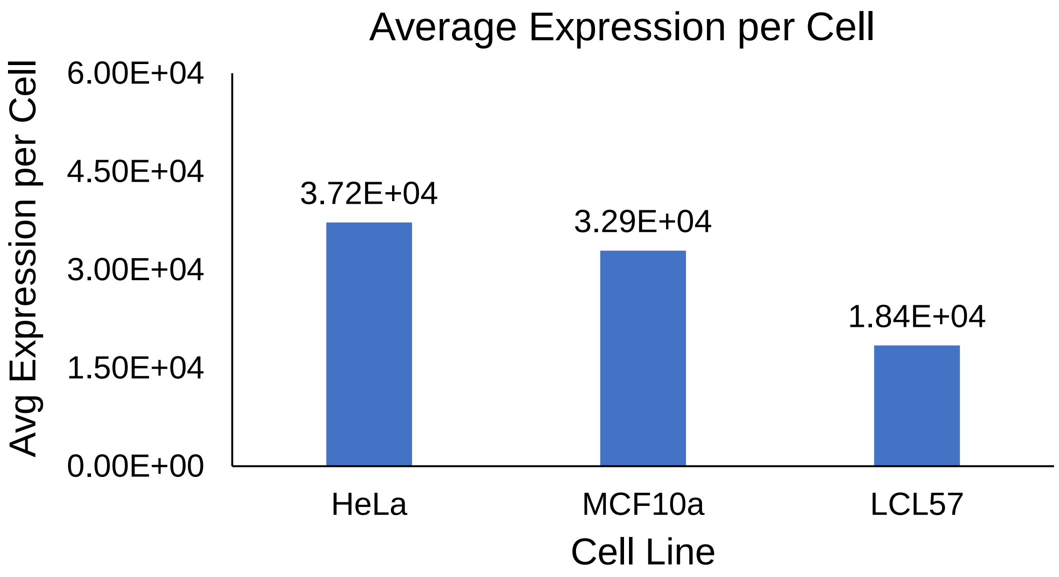

C.

Single cell western blot data for LMNB1 (66 kDa band). Relative expression of total LMNB1 (66 kDa) in HeLa, MCF10A, and LCL57 cells (A). Percentage of cells that express LMNB1 (B). Average expression of LMNB1 protein per cell (C). All data is normalized to β-tubulin expression.

20. CPTC-MCM6-1

Technique	Analyte	Result
WB	RecProt	**N***
West†	HeLa	**N**
	MCF10A	**N**
	LCL57	**N**
WB	HeLa	**NT**
	MCF10A	**NT**
	LCL57	**NT**
IF	HeLa	**NT**
	MCF10A	**NT**
	LCL57	**NT**
SCWB	HeLa	**NT**
	MCF10A	**NT**
	LCL57	**NT**
IP	RecProt	**NT**
IP	HeLa	**NT**
	MCF10A	**NT**
	LCL57	**NT**
IHC		**N***
NCI60		**NT**

N – Negative, NT – Not Tested, * Data not shown
† Data shown are from Phospho-specificity Experiment.

Endogenous Wes

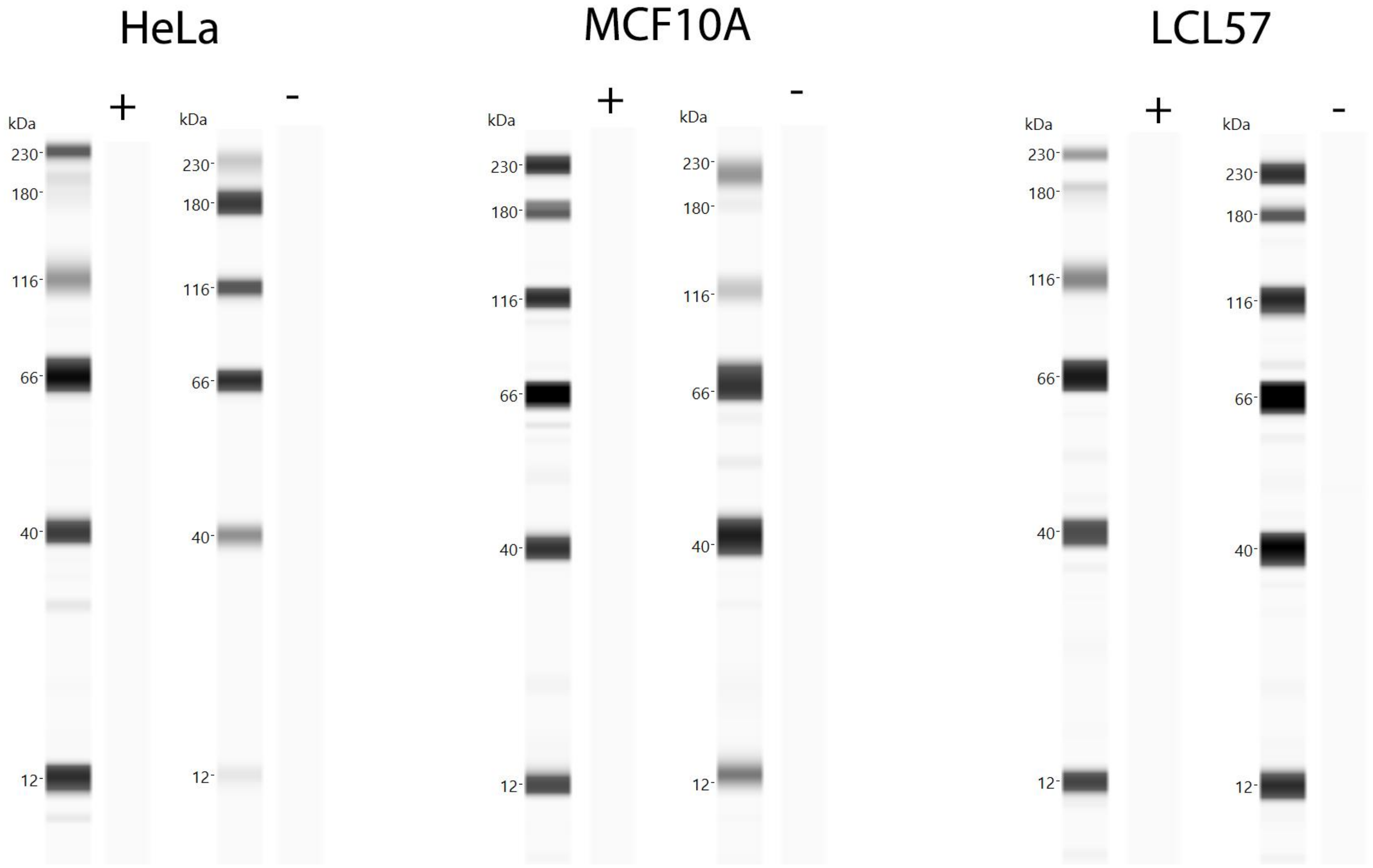

Expected molecular weight: 93 kDa

+ : irradiation treated cells
- : not treated cells

21. CPTC-MDM2-1

Technique	Analyte	Result
WB	RecProt	P
Wes	HeLa	U
	MCF10A	U
	LCL57	U
WB	HeLa	P
	MCF10A	P
	LCL57	P
IF	HeLa	P
	MCF10A	P
	LCL57	P
SCWB	HeLa	P
	MCF10A	P
	LCL57	P
IP	RecProt	NT
IP	HeLa	N
	MCF10A	N
	LCL57	N
IHC		N*
NCI60		NT

N – Negative, NT – Not tested, * Data not shown, U - Undetermined

WB against commercial recombinant protein

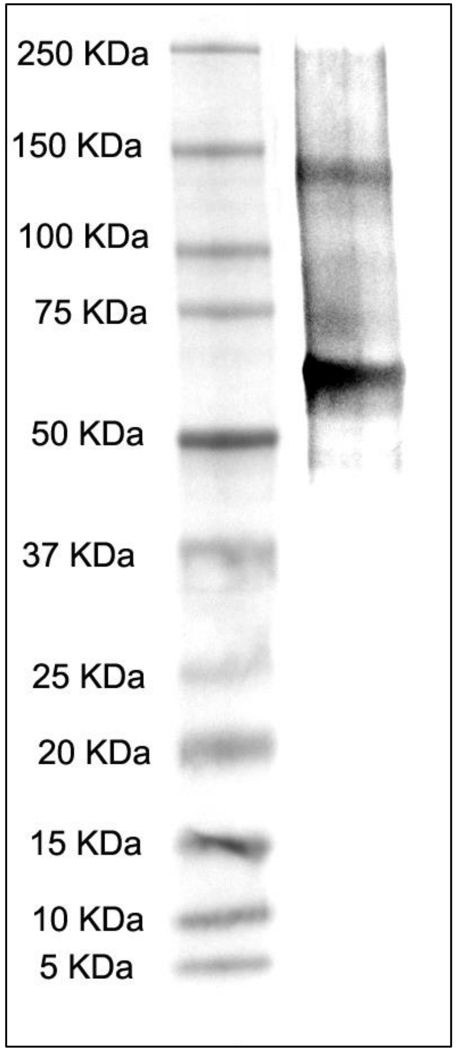

Ag Vendor: Origene

Cat #: TP761916

Description: Purified recombinant protein of Human Mdm2 p53 binding protein homolog (mouse) (MDM2), transcript variant MDM2, Gln119-Leu438, with N-terminal HIS tag, expressed in E. coli

Predicted MW: 35.9 kDa

Endogenous WB

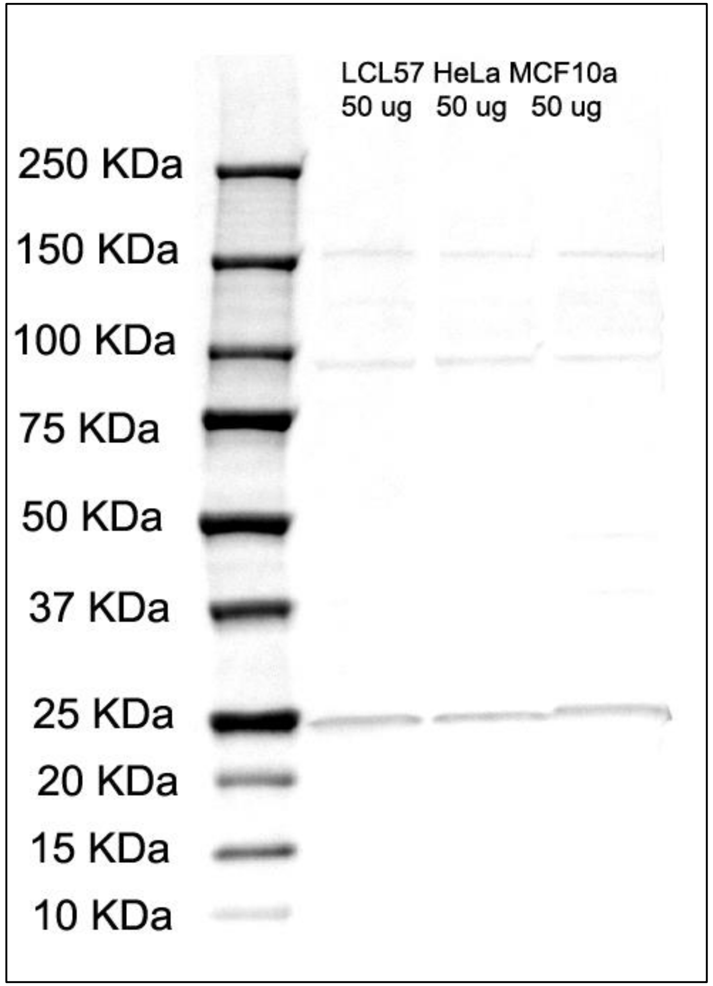

Endogenous Wes

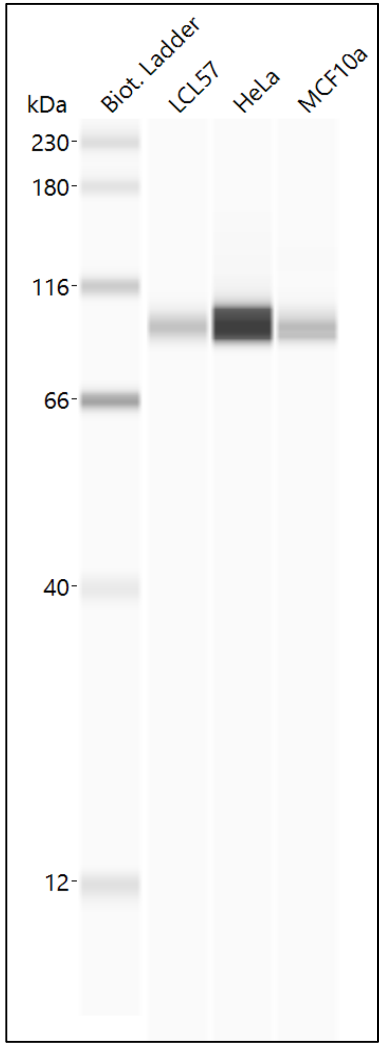

Immunofluorescence - HeLa

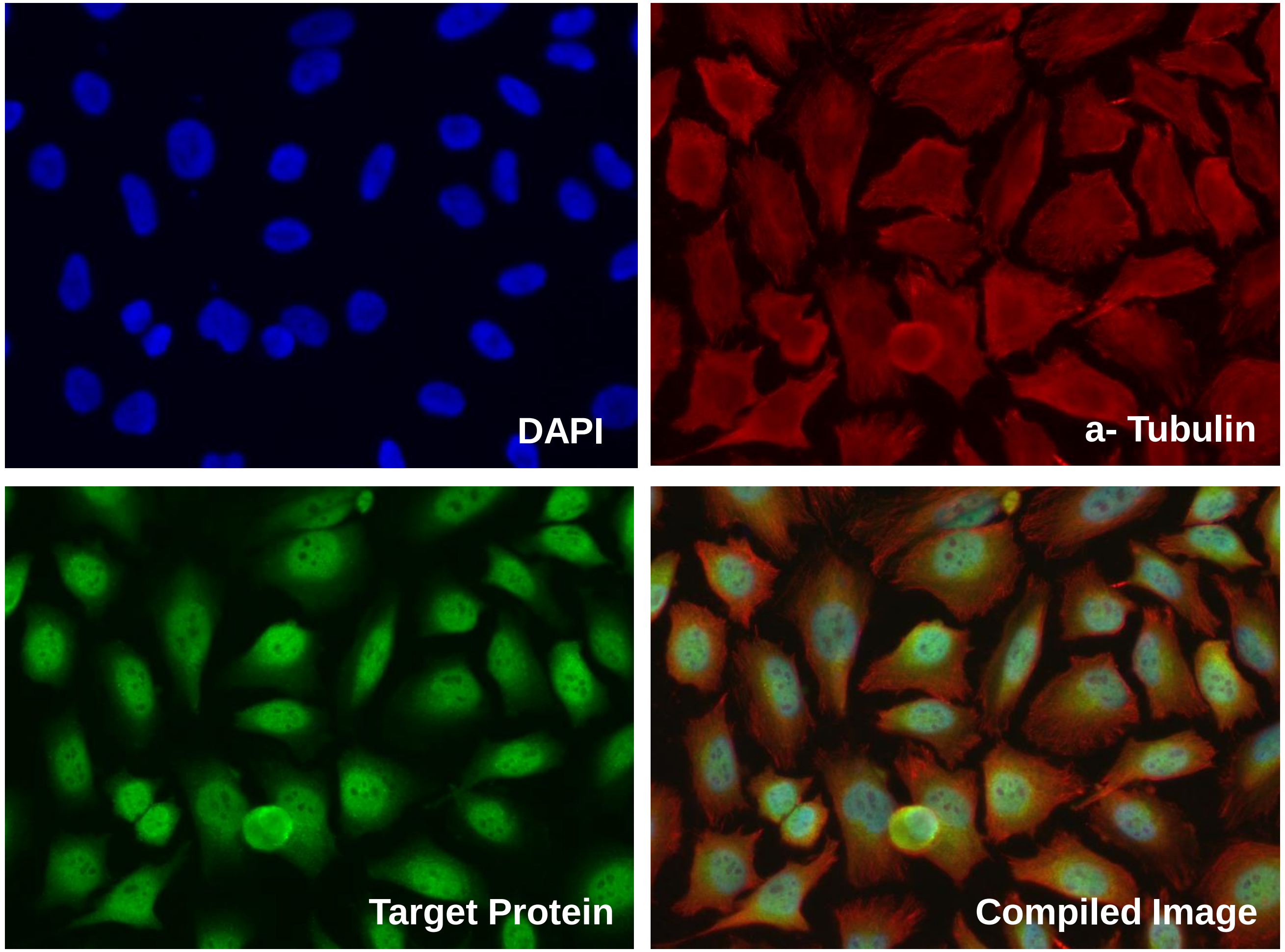

Subcellular Location: Nucleus, Other

Immunofluorescence - MCF10A

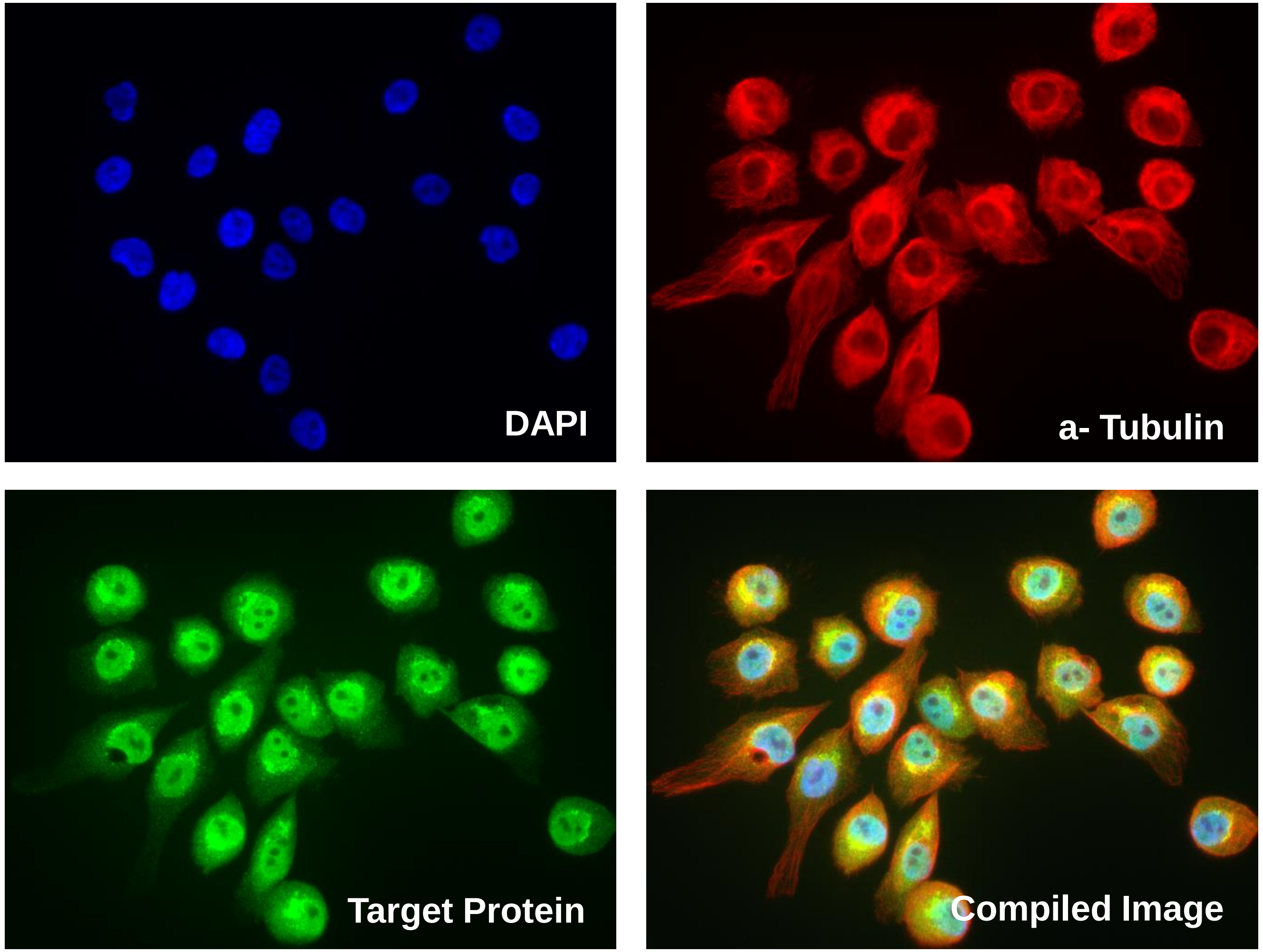

Subcellular Location: Nucleus, Other

Immunofluorescence - LCL57

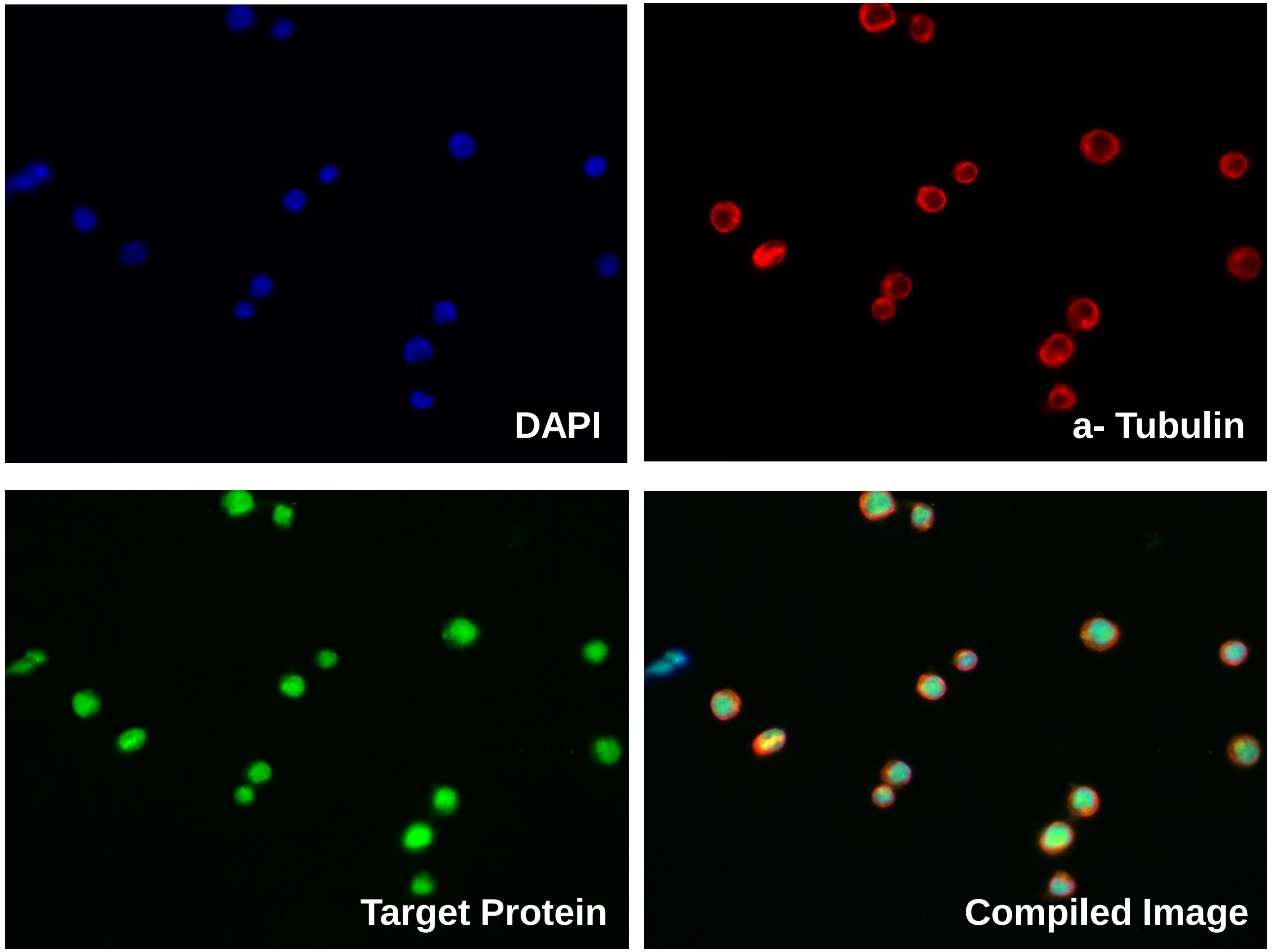

Subcellular Location: Nucleus, Other

Single Cell Western Blot

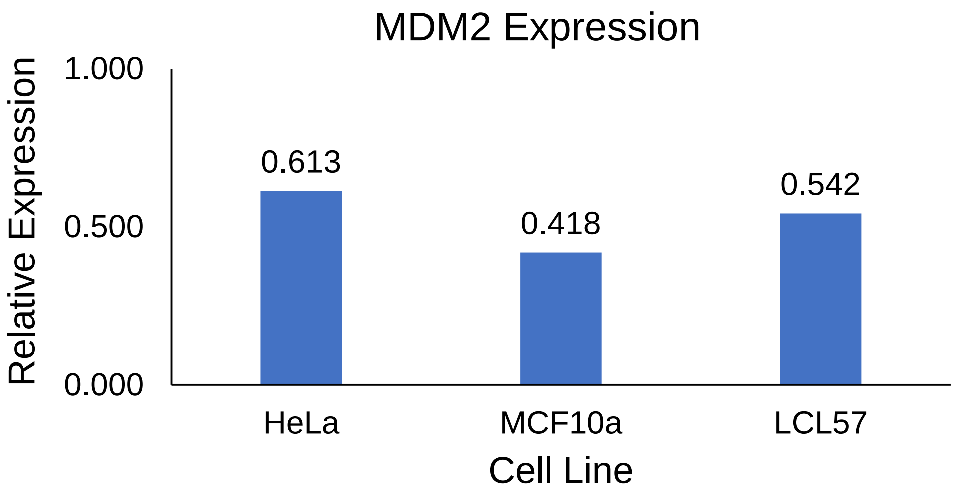

A.

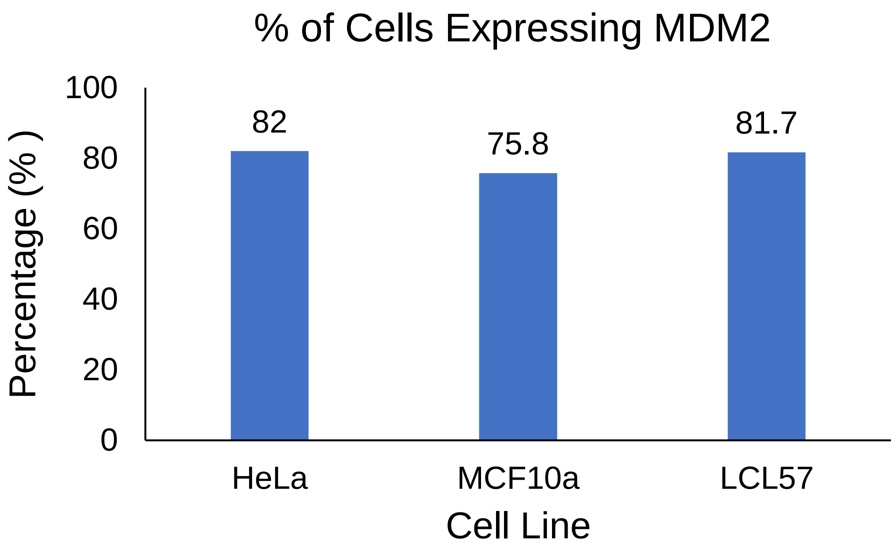

B.

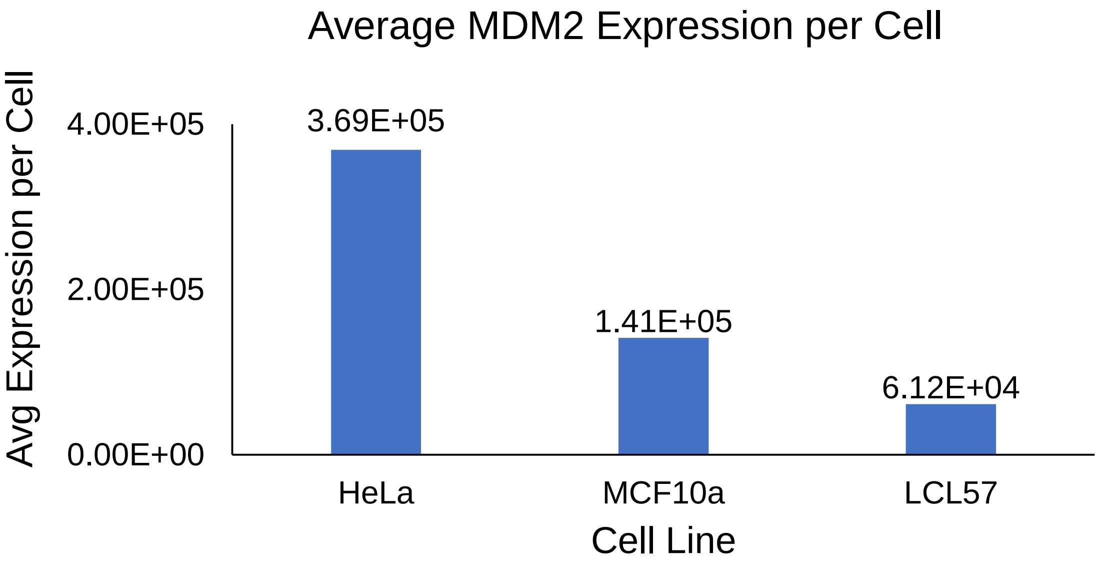

C.

Single cell western blot data for MDM2 (66 kDa band). Relative expression of total MDM2 (66 kDa) in HeLa, MCF10A, and LCL57 cells (A). Percentage of cells that express MDM2 (B). Average expression of MDM2 protein per cell (C). All data is normalized to β-tubulin expression.

22. CPTC-MKI67-3

Technique	Analyte	Result
WB	RecProt	N*
West†	HeLa	U
	MCF10A	U
	LCL57	N
WB§	HeLa	N
	MCF10A	N
	LCL57	N
IF	HeLa	NT
	MCF10A	NT
	LCL57	NT
SCWB	HeLa	NT
	MCF10A	NT
	LCL57	NT
IP	RecProt	NT
IP	HeLa	NT
	MCF10A	NT
	LCL57	NT
IHC		P
NCI60		P

P – Positive, N – Negative, NT – Not Tested, * Data not shown, U - Uncertain
† Data shown are from Phospho-specificity Experiment. § Data from Phospho-specificity Experiment are not shown.

Endogenous Wes

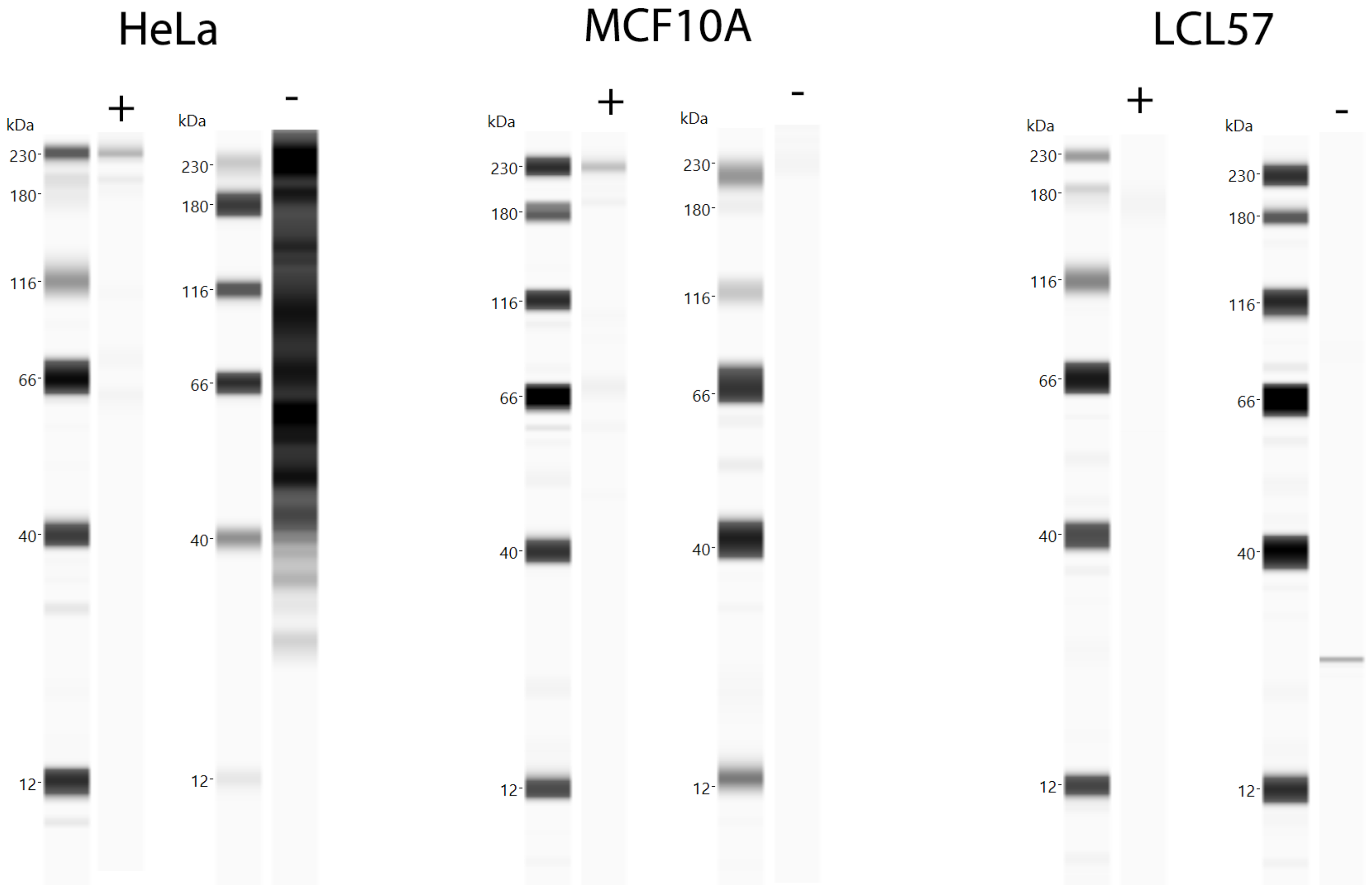

Expected molecular weight: 359, 319 kDa

+ : irradiation treated cells
- : not treated cells

CPTC-MKi67-3

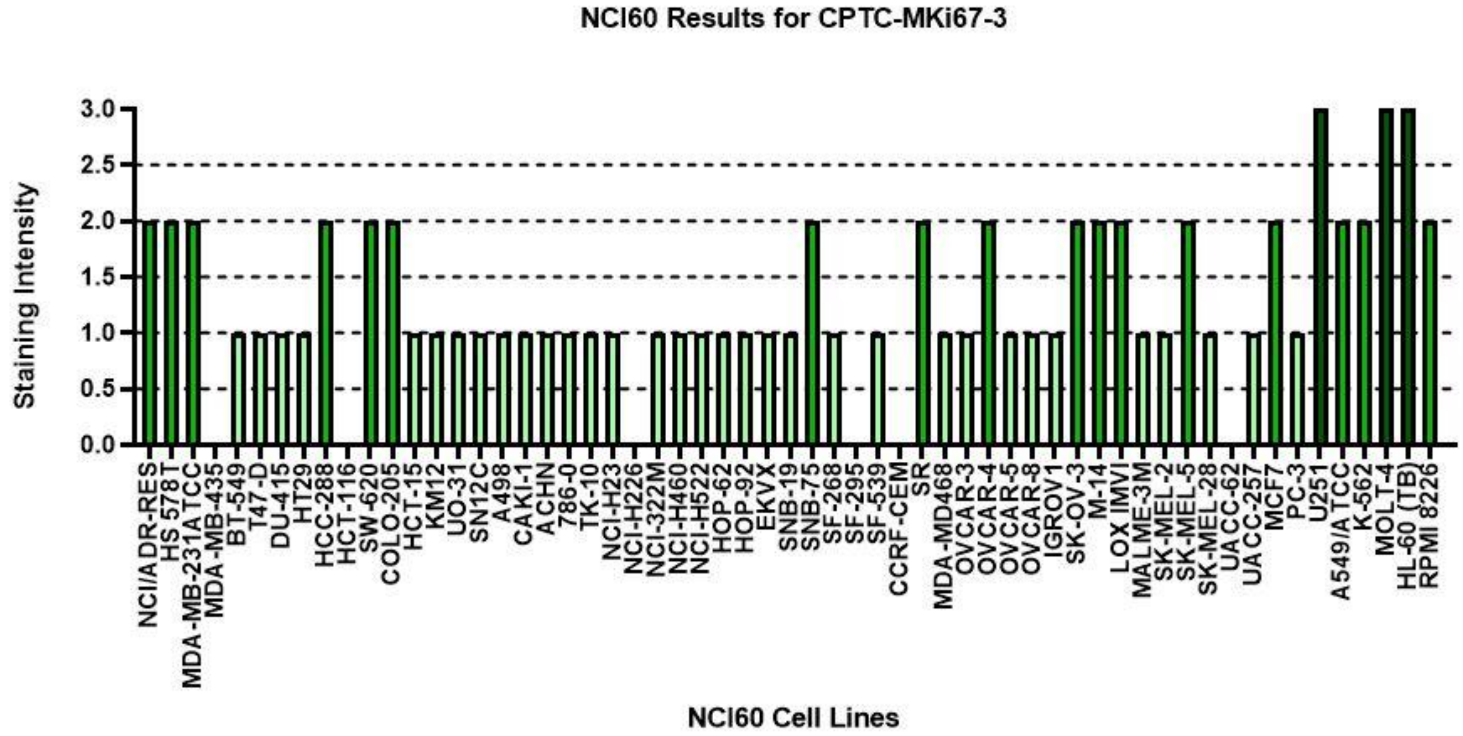
NCI60 Results for CPTC-MKi67-3
Staining Intensity
3.0
2.5
2.0
1.5
1.0
0.5
0.0
NCI/ADR-RES
HS 578T
MDA-MB-231ATCC
MDA-MB-435
BT-549
T47-D
DU-415
HT29
HCC-288
HCT-116
SW-620
COLO-205
HCT-15
KM12
UO-31
SN12C
A498
CAKI-1
ACHN
786-0
TK-10
NCI-H23
NCI-H226
NCI-322M
NCI-H460
NCI-H522
HOP-62
HOP-92
EKVX
SNB-19
SNB-75
SF-268
SF-295
SF-539
CCRF-CEM
SR
MDA-MD468
OVCAR-3
OVCAR-4
OVCAR-5
OVCAR-8
IGROV1
SK-OV-3
M-14
LOX IMVI
MALME-3M
SK-MEL-2
SK-MEL-5
SK-MEL-28
UACC-62
UACC-257
MCF7
PC-3
U251
A549/ATCC
K-562
MOLT-4
HL-60 (TB)
RPMI 8226
NCI60 Cell Lines

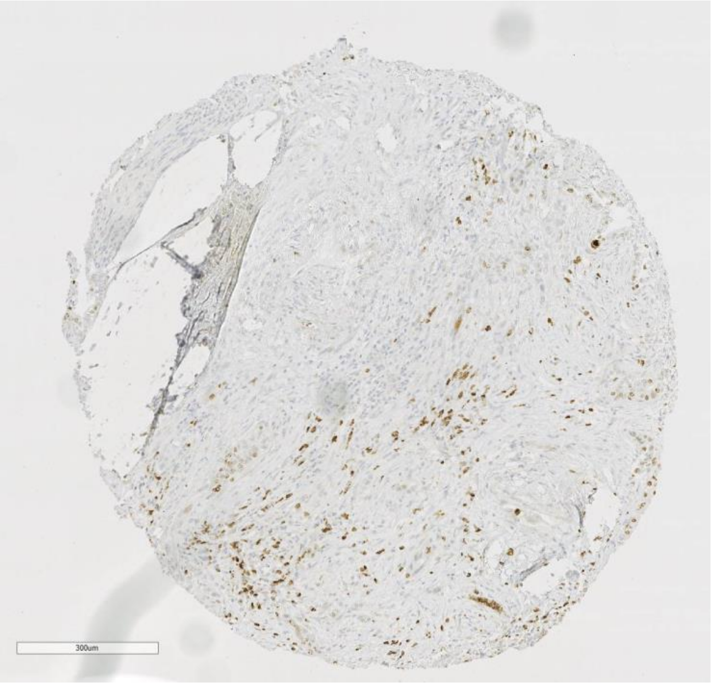

23. CPTC-MKI67-4

Technique	Analyte	Result
WB	RecProt	N*
West†	HeLa	N
	MCF10A	N
	LCL57	N
WB	HeLa	NT
	MCF10A	NT
	LCL57	NT
IF	HeLa	NT
	MCF10A	NT
	LCL57	NT
SCWB	HeLa	NT
	MCF10A	NT
	LCL57	NT
IP	RecProt	NT
IP	HeLa	NT
	MCF10A	NT
	LCL57	NT
IHC		P
NCI60		P

N – Negative, NT – Not Tested, * Data not shown
† Data shown are from Phospho-specificity Experiment.

Endogenous Wes

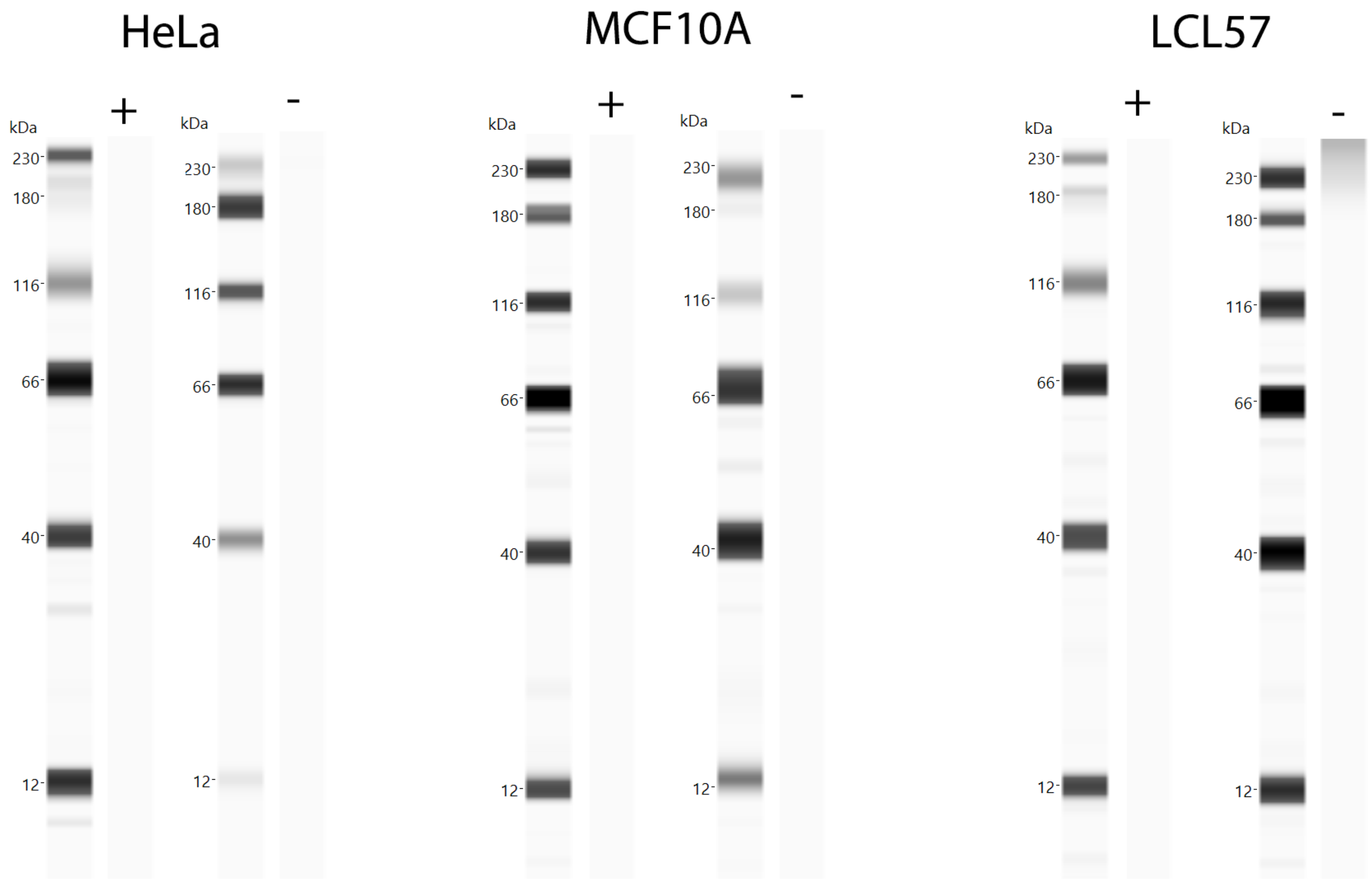

Expected molecular weight: 359, 319 kDa

+ : irradiation treated cells
- : not treated cells

CPTC-MKi67-4

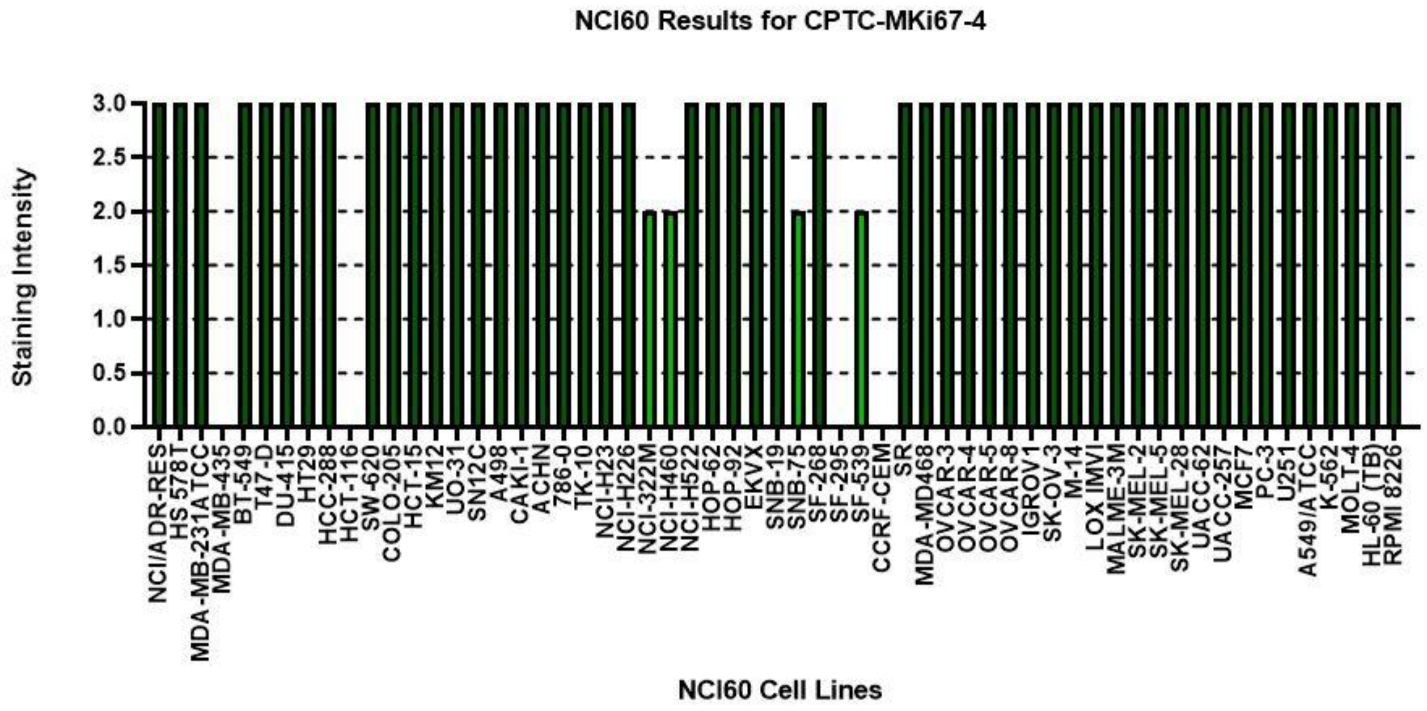
NCI60 Results for CPTC-MKi67-4
Staining Intensity
3.0
2.5
2.0
1.5
1.0
0.5
0.0
NCI/ADR-RES
HS 578T
MDA-MB-231ATCC
MDA-MB-435
BT-549
T47-D
DU-415
HT29
HCC-288
HCT-116
SW-620
COLO-205
HCT-15
KM12
UO-31
SN12C
A498
CAKI-1
ACHN
786-0
TK-10
NCI-H23
NCI-H226
NCI-322M
NCI-H460
NCI-H522
HOP-62
HOP-92
EKVX
SNB-19
SNB-75
SF-268
SF-295
SF-539
CCRF-CEM
SR
MDA-MD468
OVCAR-3
OVCAR-4
OVCAR-5
OVCAR-8
IGROV1
SK-OV-3
M-14
LOX IMVI
MALME-3M
SK-MEL-2
SK-MEL-5
SK-MEL-28
UACC-62
UACC-257
MCF7
PC-3
U251
A549/ATCC
K-562
MOLT-4
HL-60 (TB)
RPMI 8226
NCI60 Cell Lines

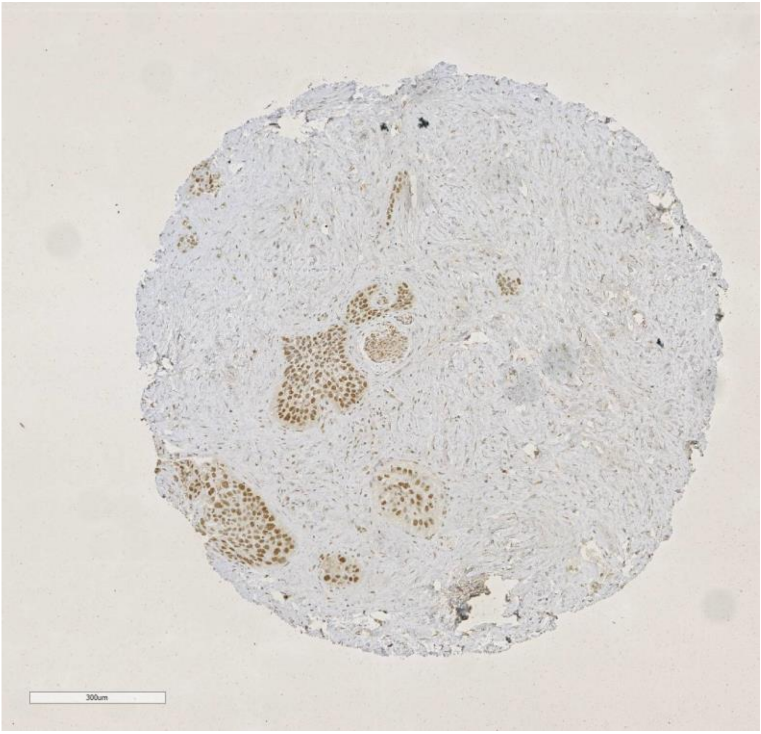

24. CPTC-MRE11A-1

Technique	Analyte	Result
WB	RecProt	**N***
West†	HeLa	**N**
	MCF10A	**N**
	LCL57	**N**
WB	HeLa	**NT**
	MCF10A	**NT**
	LCL57	**NT**
IF	HeLa	**NT**
	MCF10A	**NT**
	LCL57	**NT**
SCWB	HeLa	**NT**
	MCF10A	**NT**
	LCL57	**NT**
IP	RecProt	**NT**
IP	HeLa	**NT**
	MCF10A	**NT**
	LCL57	**NT**
IHC		**P**
NCI60		**P**

N – Negative, NT – Not Tested, * Data not shown
† Data shown are from Phospho-specificity Experiment.

Endogenous Wes

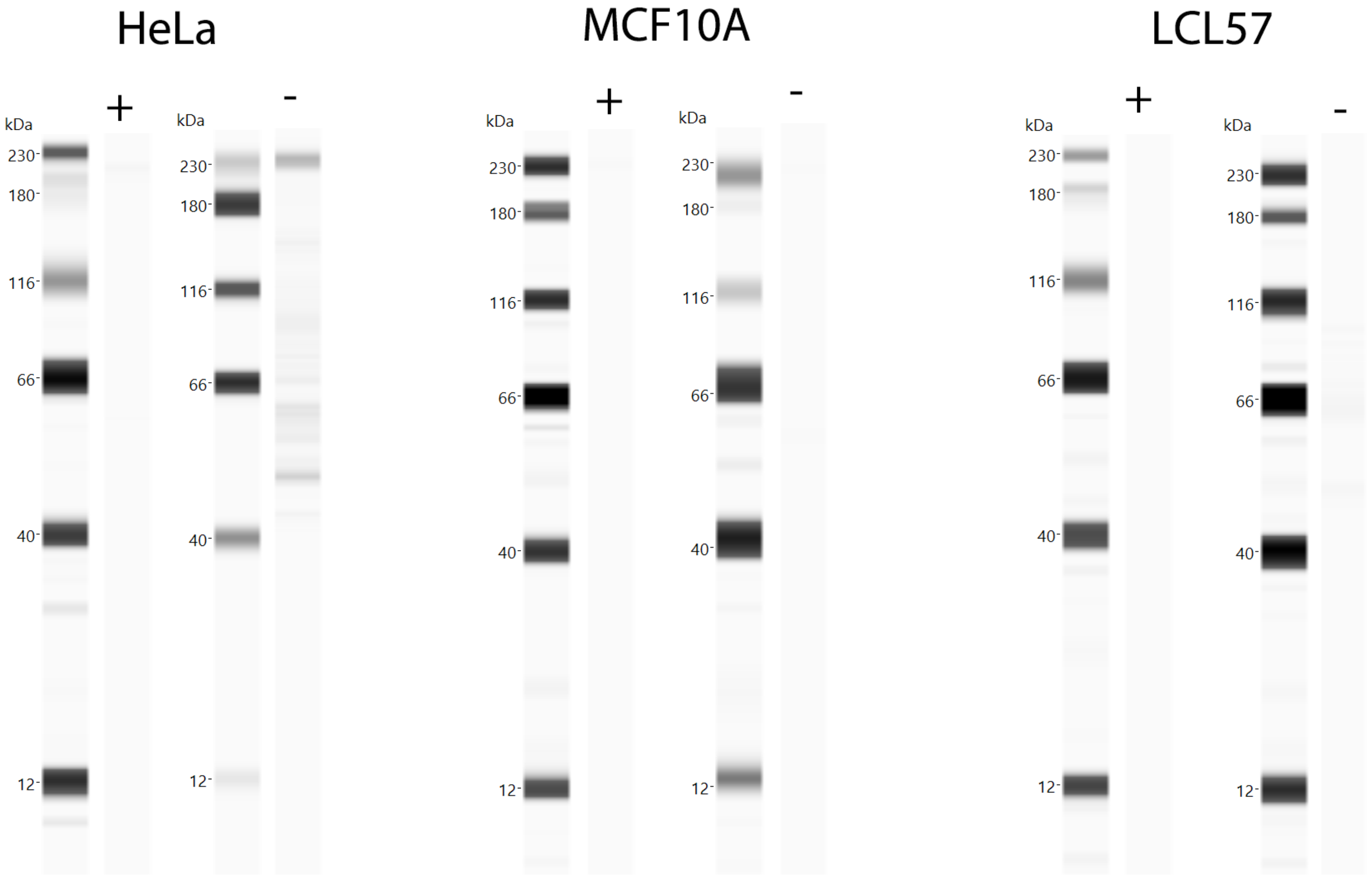

Expected molecular weight: 81, 78 kDa

+ : irradiation treated cells
- : not treated cells

CPTC-MRE11A-1

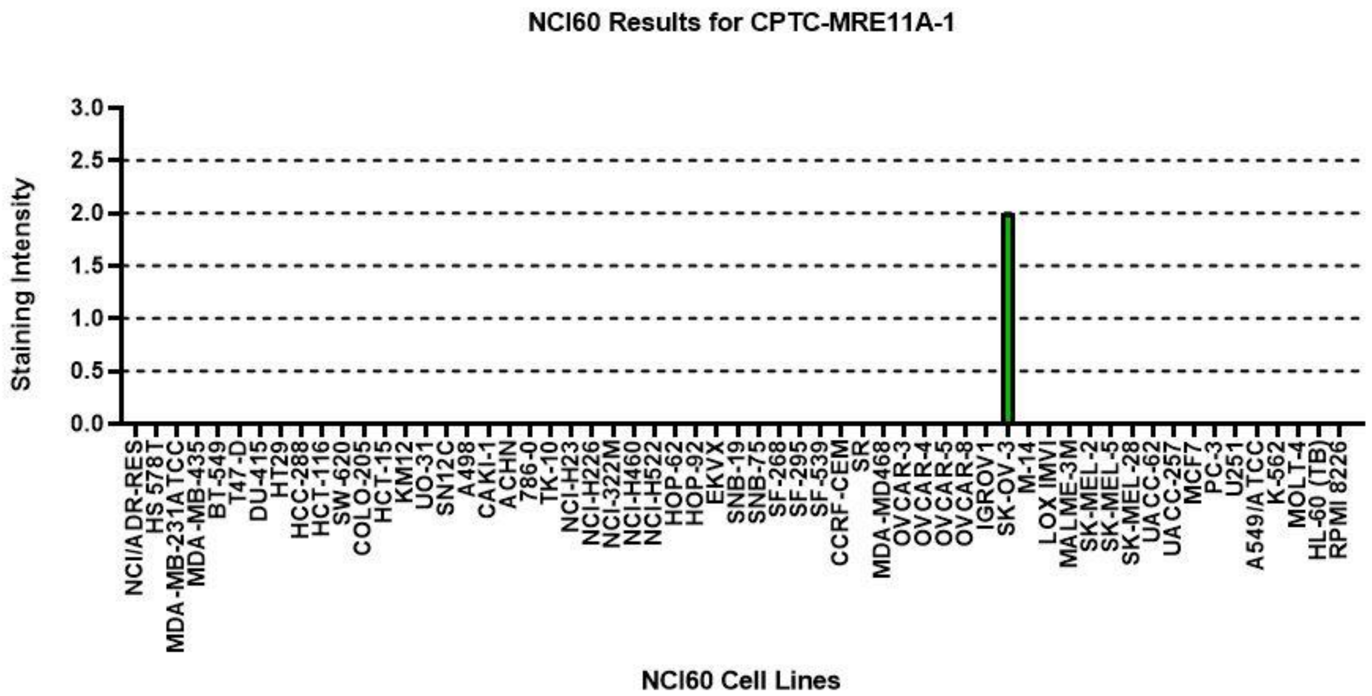

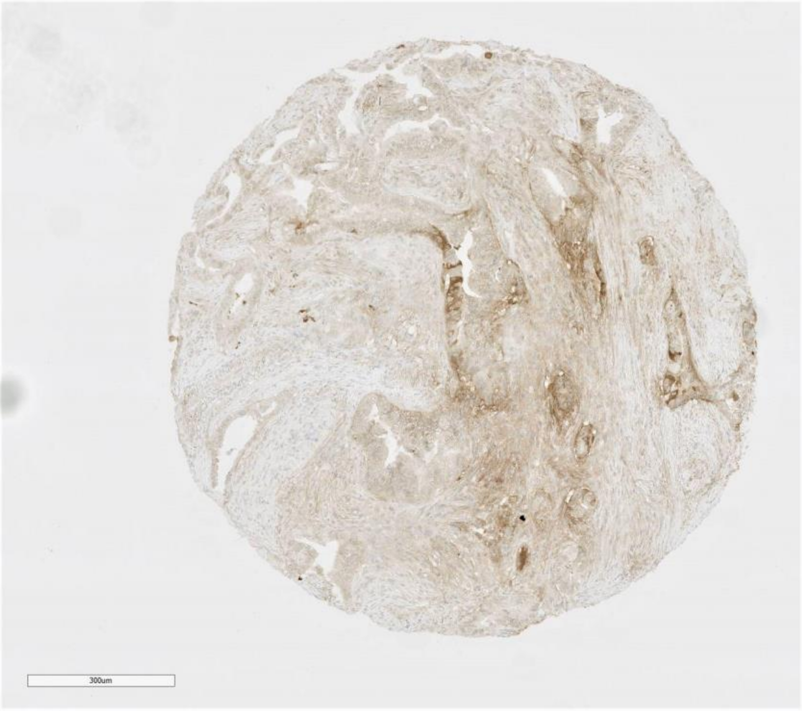

25. CPTC-NCAPH2-1

Technique	Analyte	Result
WB	RecProt	N*
West†	HeLa	N
	MCF10A	N
	LCL57	N
WB	HeLa	NT
	MCF10A	NT
	LCL57	NT
IF	HeLa	NT
	MCF10A	NT
	LCL57	NT
SCWB	HeLa	NT
	MCF10A	NT
	LCL57	NT
IP	RecProt	NT
IP	HeLa	NT
	MCF10A	NT
	LCL57	NT
IHC		P
NCI60		P

N – Negative, NT – Not Tested, * Data not shown
† Data shown are from Phospho-specificity Experiment.

Endogenous Wes

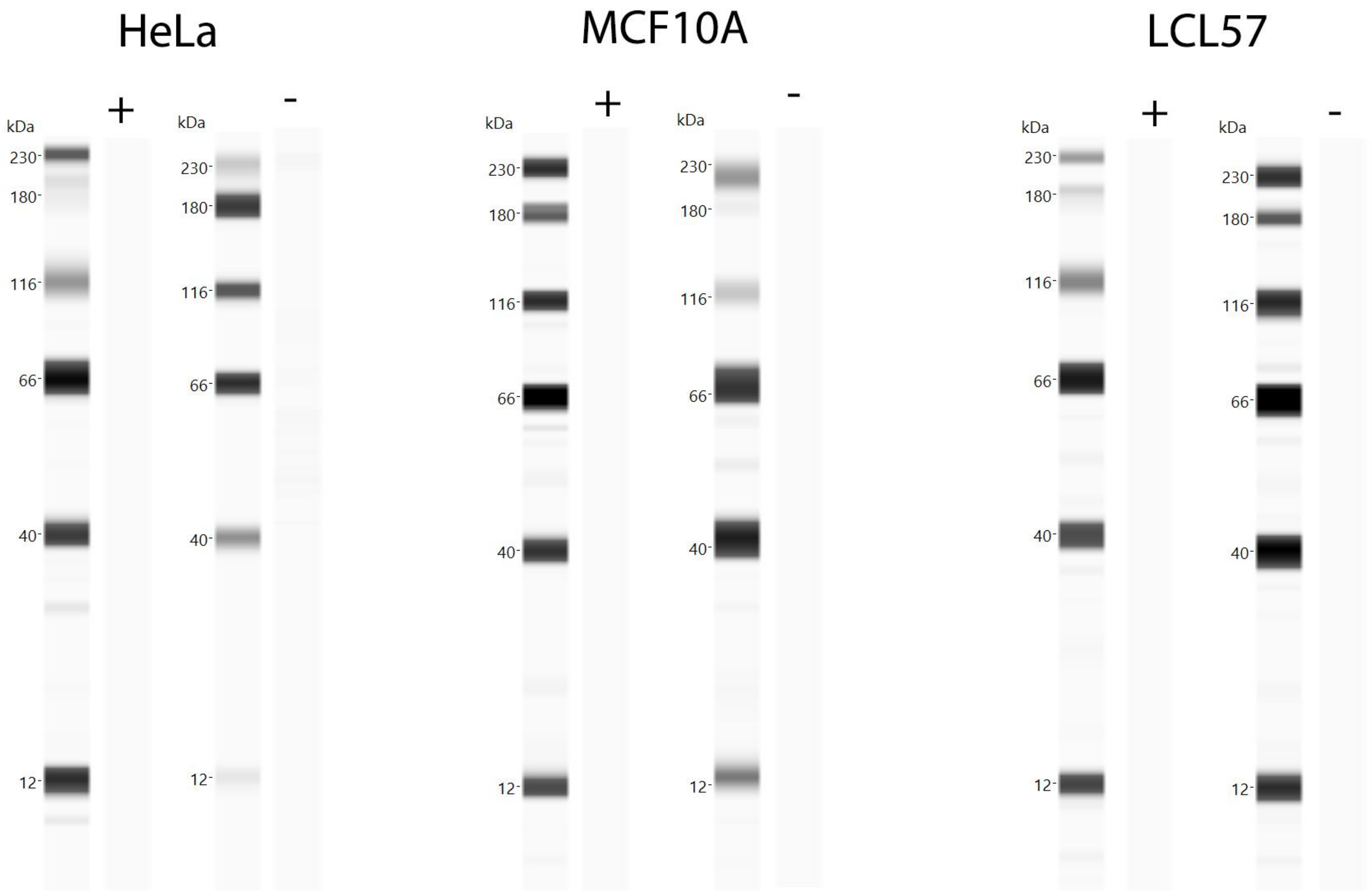

Expected molecular weight: 68, 66, 33 kDa

+ : irradiation treated cells
- : not treated cells

CPTC-NCAPH2-1

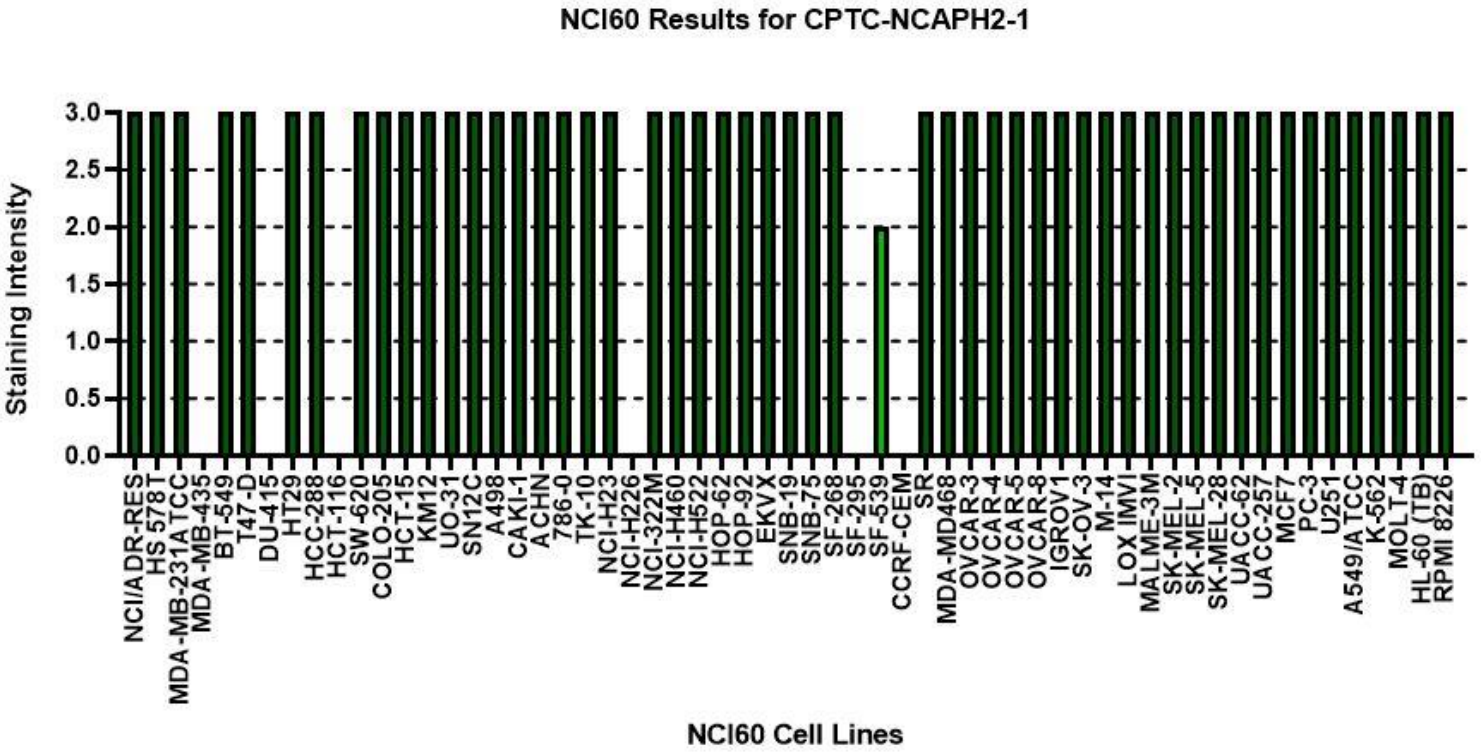

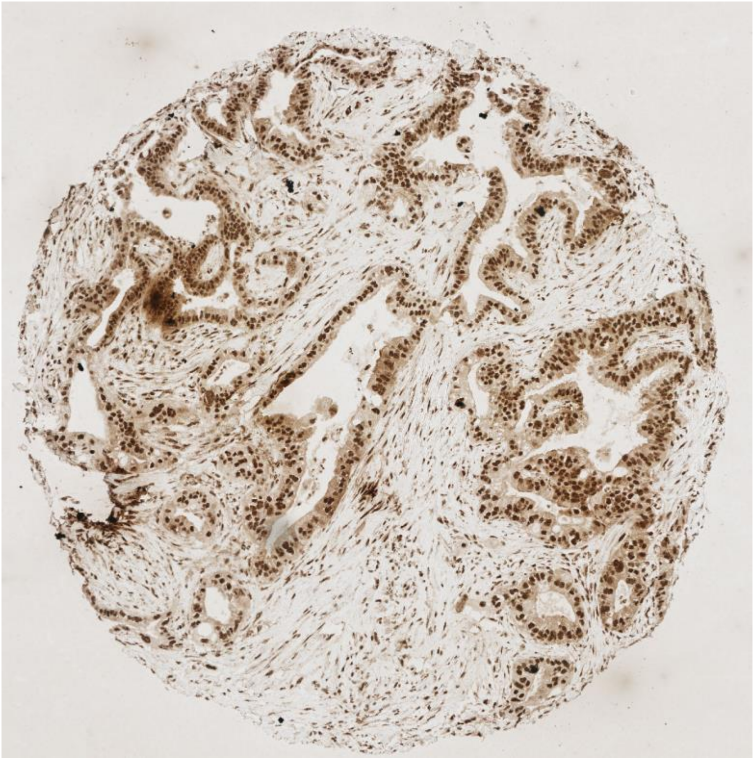

26. CPTC-NUMA1-1

Technique	Analyte	Result
WB	RecProt	N*
West†	HeLa	P
	MCF10A	P
	LCL57	P
WB§	HeLa	U*
	MCF10A	U*
	LCL57	P
IF	HeLa	NT
	MCF10A	NT
	LCL57	NT
SCWB	HeLa	NT
	MCF10A	NT
	LCL57	NT
IP	RecProt	NT
IP	HeLa	NT
	MCF10A	NT
	LCL57	NT
IHC		P
NCI60		P

P – Positive, N – Negative, NT – Not Tested, * Data not shown, U - Uncertain
† Data shown are from Phospho-specificity Experiment. § Data shown are from Phospho-specificity Experiment.

Endogenous Wes

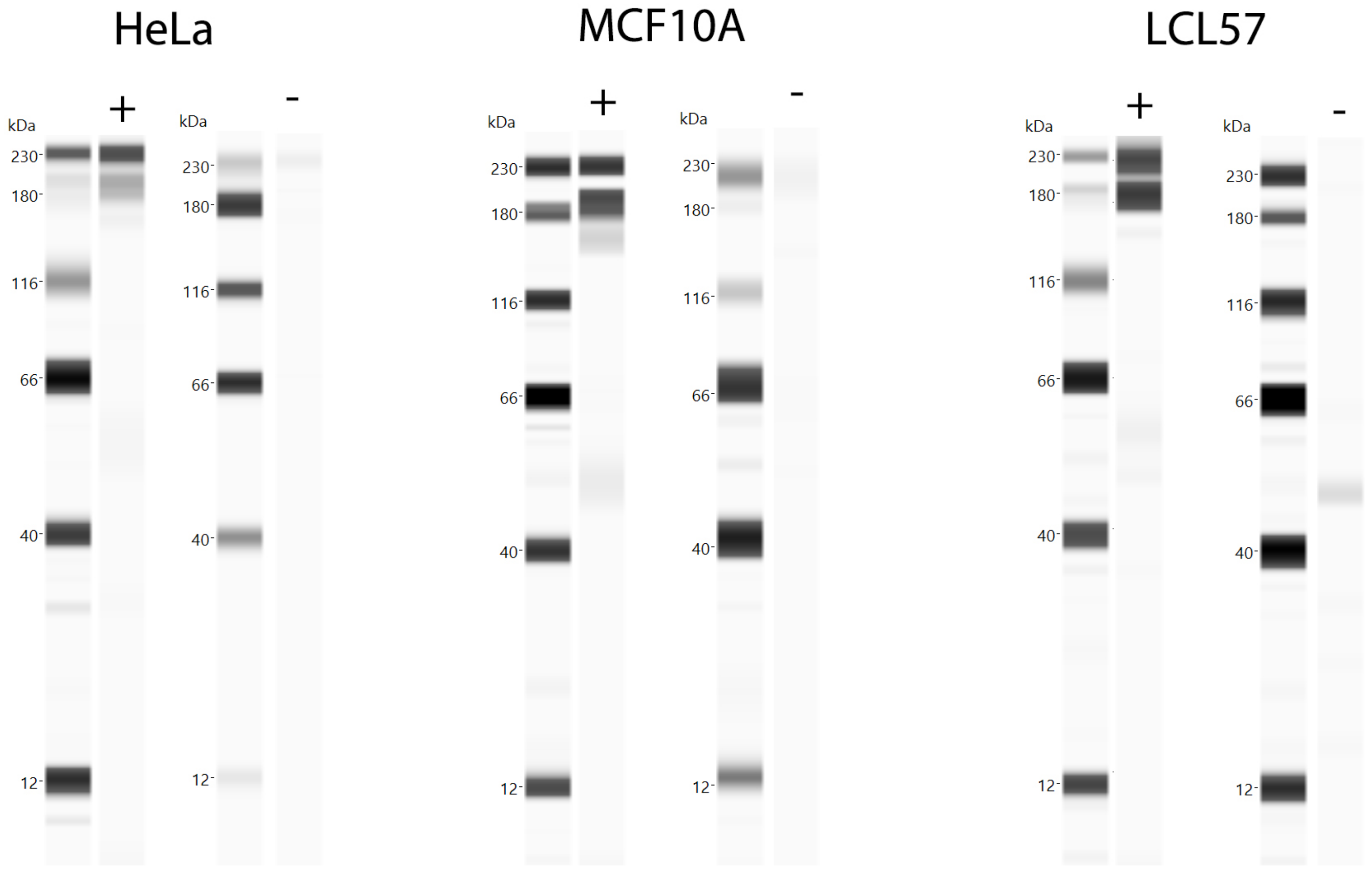

Expected molecular weight: 238, 237, 201, 200, 109 kDa

+ : irradiation treated cells
- : not treated cells

CPTC-NUMA1-1

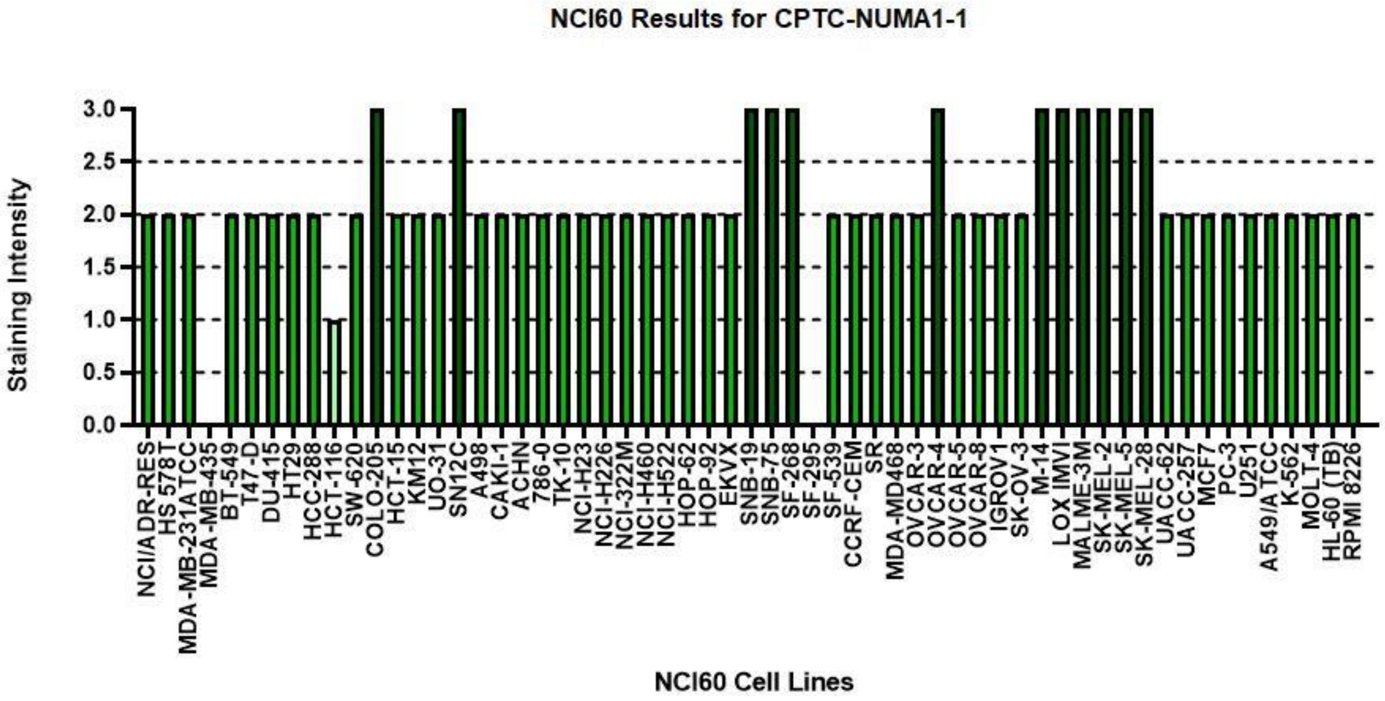

NCI60 Results for CPTC-NUMA1-1
Staining Intensity
3.0
2.5
2.0
1.5
1.0
0.5
0.0
NCI/ADR-RES
HS 578T
MDA-MB-231ATCC
MDA-MB-435
BT-549
T47-D
DU-415
HT29
HCC-288
HCT-116
SW-620
COLO-205
HCT-15
KM12
UO-31
SN12C
A498
CAKI-1
ACHN
786-0
TK-10
NCI-H23
NCI-H226
NCI-322M
NCI-H460
NCI-H522
HOP-62
HOP-92
EKVX
SNB-19
SNB-75
SF-268
SF-295
SF-539
CCRF-CEM
SR
MDA-MD468
OVCAR-3
OVCAR-4
OVCAR-5
OVCAR-8
IGROV1
SK-OV-3
M-14
LOX IMVI
MALME-3M
SK-MEL-2
SK-MEL-5
SK-MEL-28
UACC-62
UACC-257
MCF7
PC-3
U251
A549/ATCC
K-562
MOLT-4
HL-60 (TB)
RPMI 8226
NCI60 Cell Lines

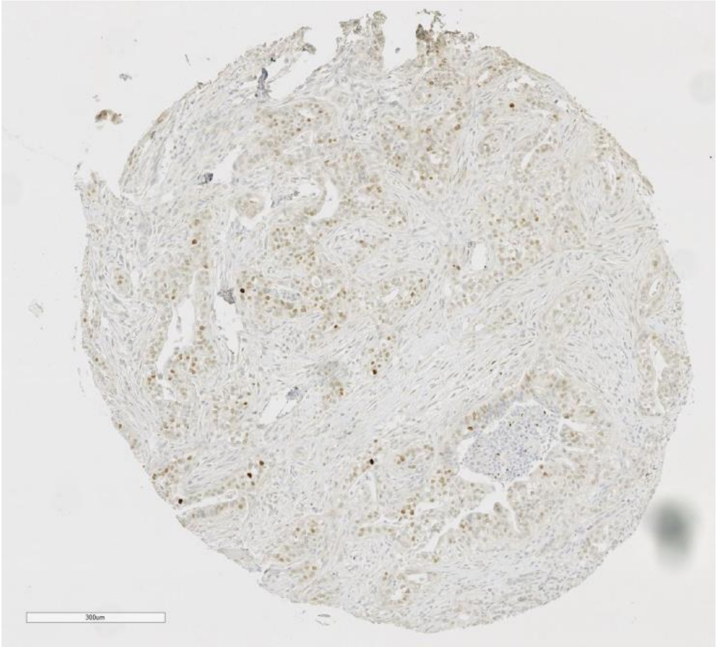

Traditional Western Blot

Expected molecular weight:
238, 237, 201, 200, 109 kDa

+ : Irradiation treated cells
 - : Non-treated cells

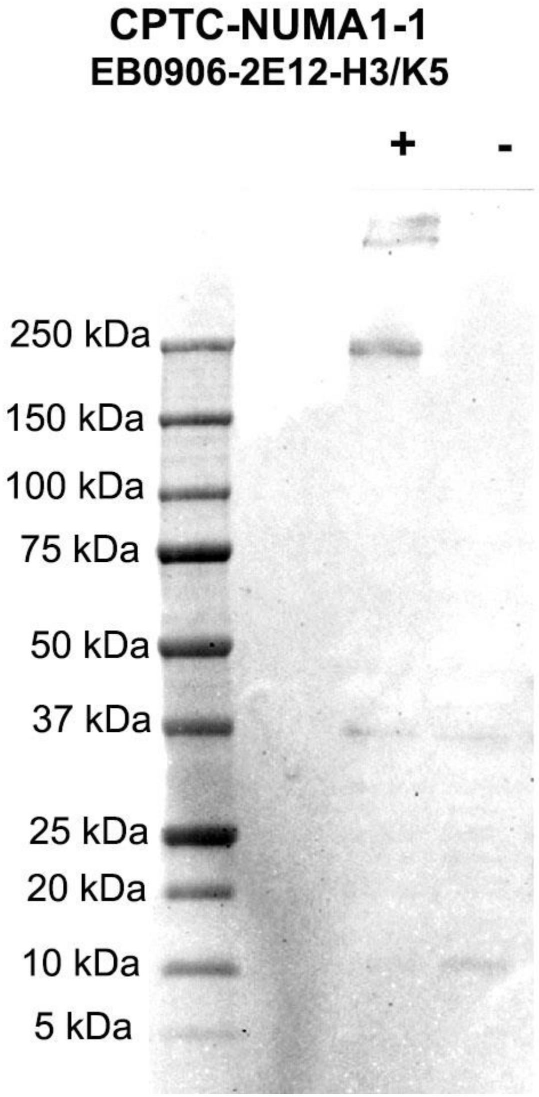

27. CPTC-PAK4-1

Technique	Analyte	Result
WB	RecProt	P
Wes	HeLa	P
	MCF10A	P
	LCL57	P
WB	HeLa	N
	MCF10A	N
	LCL57	N
IF	HeLa	N
	MCF10A	N
	LCL57	P
SCWB†	HeLa	N
	MCF10A	N
	LCL57	N
IP*	RecProt	P
IP*	HeLa	N
	MCF10A	N
	LCL57	N
IHC		P
NCI60		P

P – Positive, N – Negative, * Please refer to tables for results, † Data not shown

WB against commercial recombinant protein

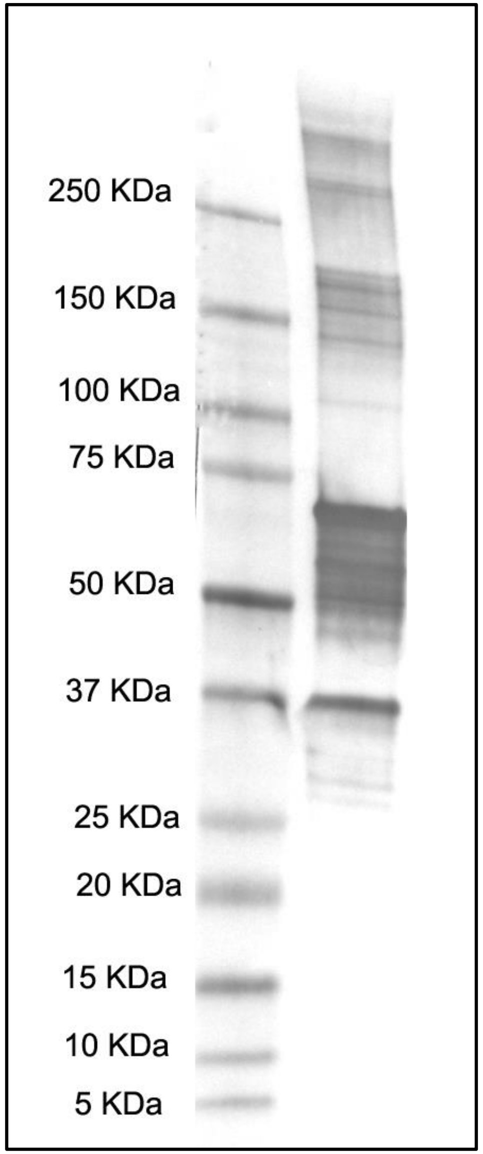

Ag Vendor: Origene

Cat #: TP302302

Description: Recombinant protein of human p21 protein (Cdc42/Rac)-activated kinase 4 (PAK4), transcript variant 1

Predicted MW 63.9 KDa

Endogenous WB

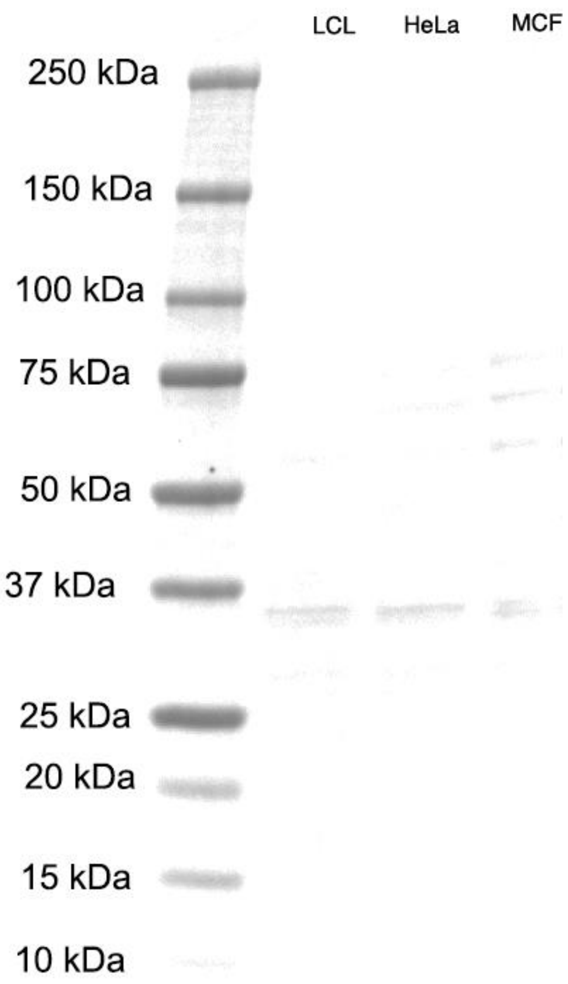

Endogenous Wes

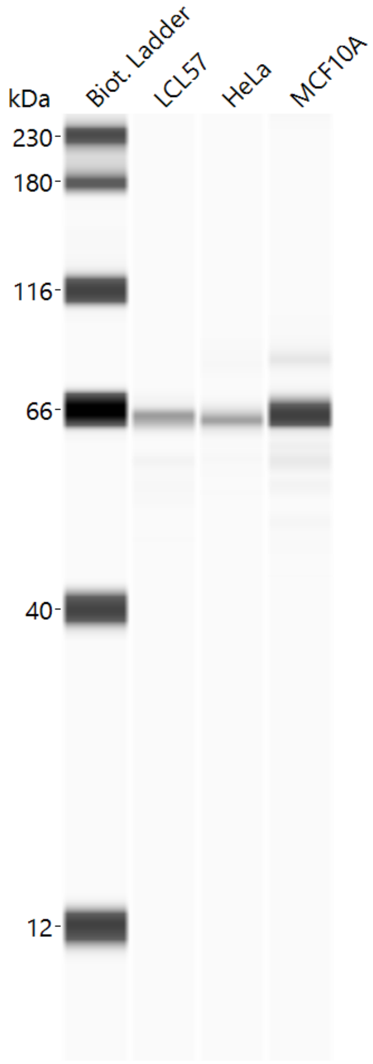

Immunofluorescence – LCL57

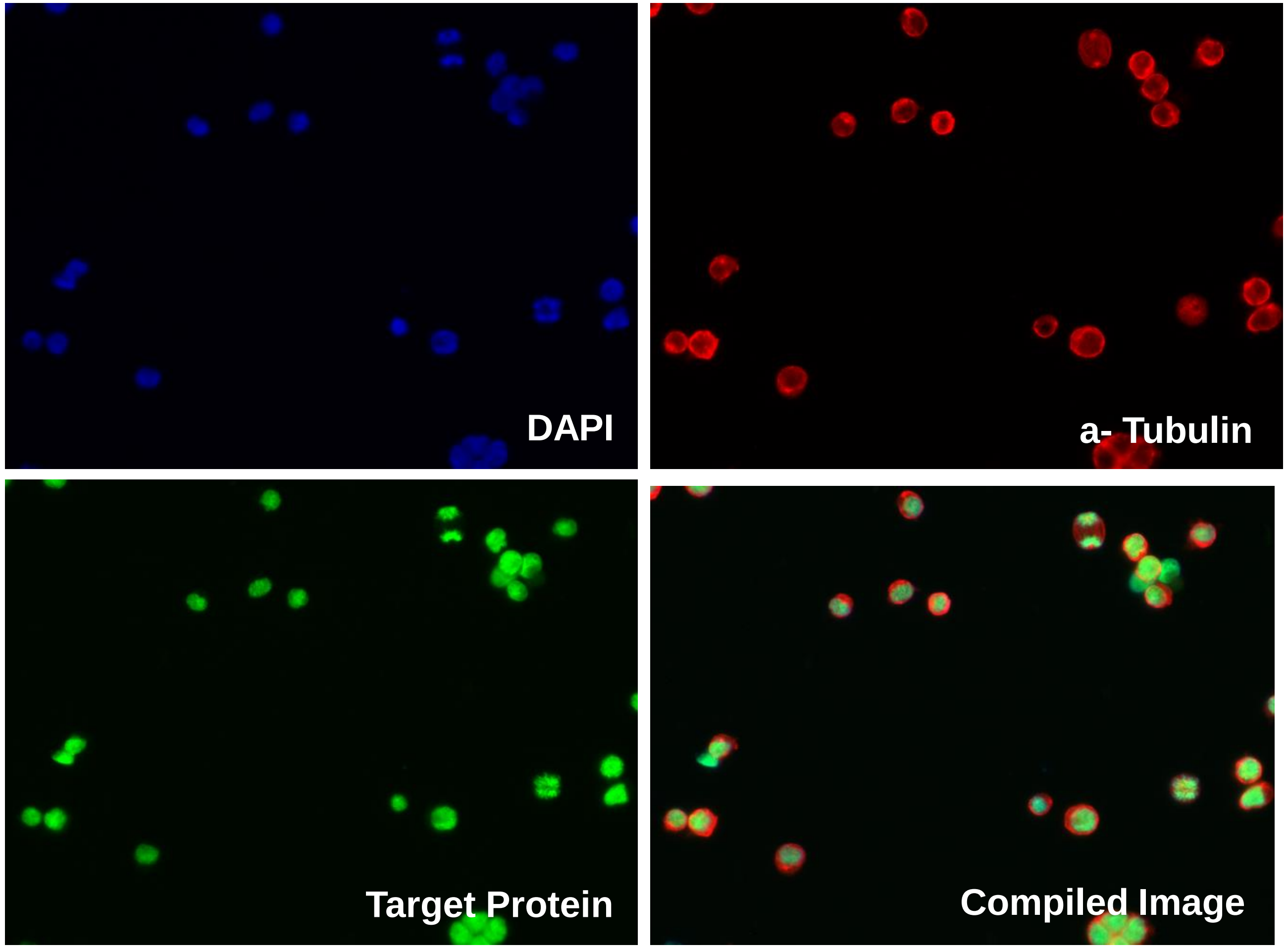

Subcellular location: Cytoplasm

CPTC-PAK4-1

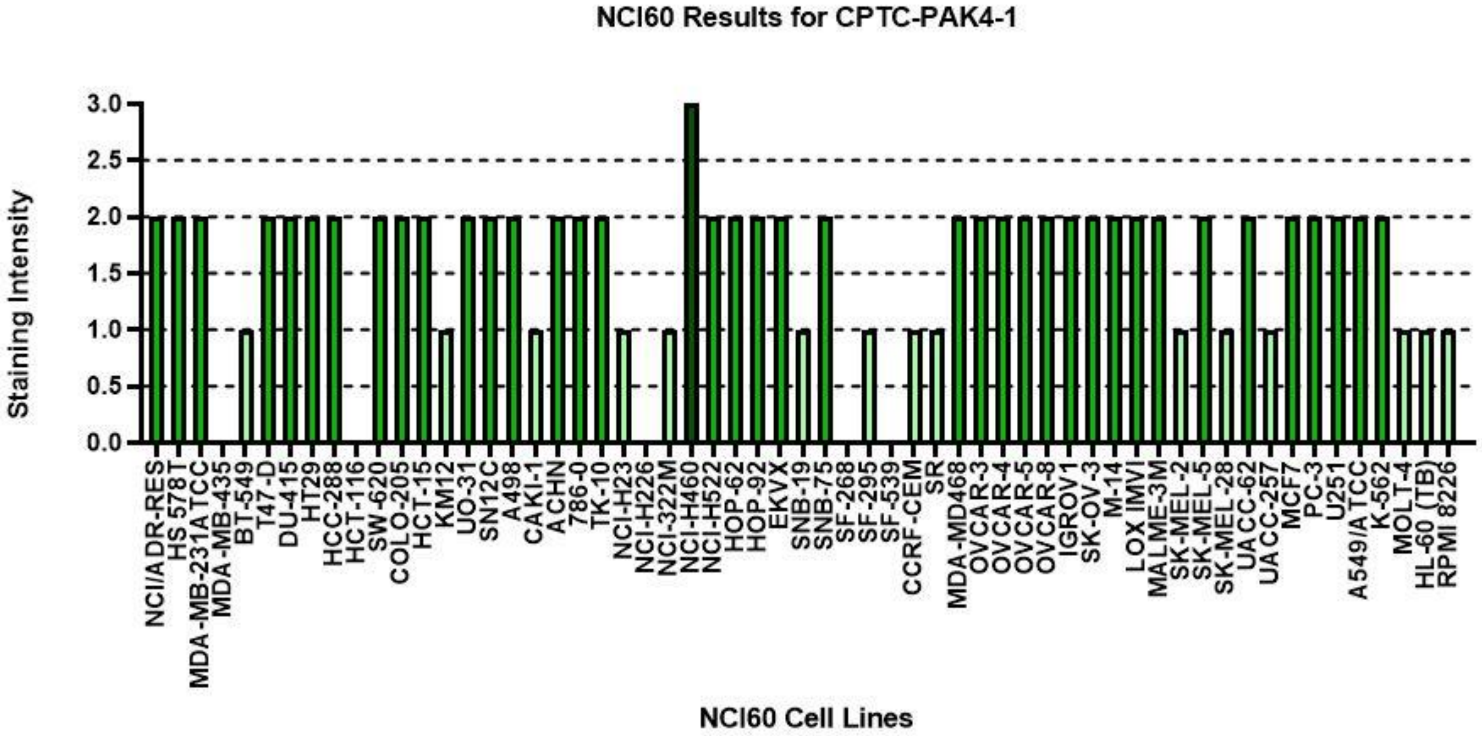
NCI60 Results for CPTC-PAK4-1
Staining Intensity
3.0
2.5
2.0
1.5
1.0
0.5
0.0
NCI/ADR-RES
HS 578T
MDA-MB-231ATCC
MDA-MB-435
BT-549
T47-D
DU-415
HT29
HCC-288
HCT-116
SW-620
COLO-205
HCT-15
KM12
UO-31
SN12C
A498
CAKI-1
ACHN
786-0
TK-10
NCI-H23
NCI-H226
NCI-322M
NCI-H460
NCI-H522
HOP-62
HOP-92
EKVX
SNB-19
SNB-75
SF-268
SF-295
SF-539
CCRF-CEM
SR
MDA-MD468
OVCAR-3
OVCAR-4
OVCAR-5
OVCAR-8
IGROV1
SK-OV-3
M-14
LOX IMVI
MALME-3M
SK-MEL-2
SK-MEL-5
SK-MEL-28
UACC-62
UACC-257
MCF7
PC-3
U251
A549/ATCC
K-562
MOLT-4
HL-60 (TB)
RPMI 8226
NCI60 Cell Lines

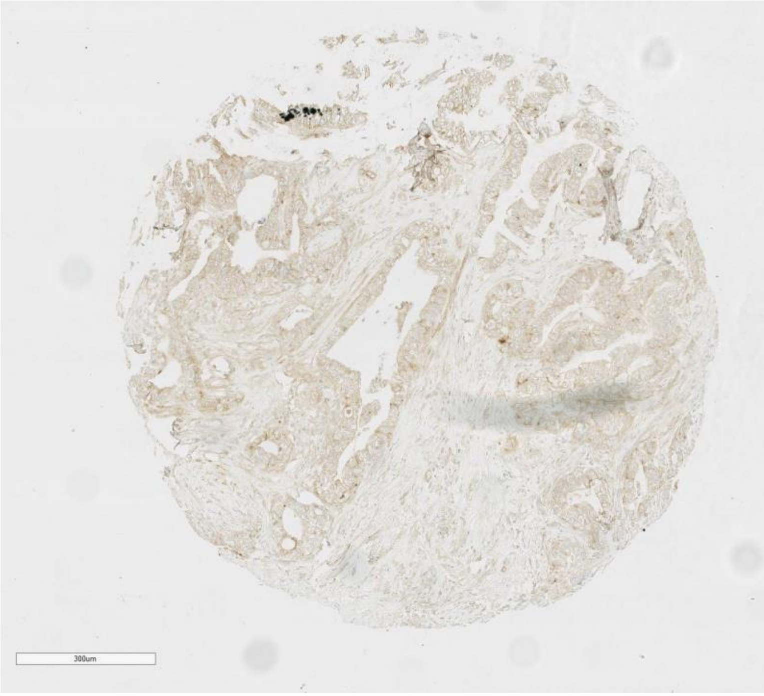

28. CPTC-PARP1-2

Technique	Analyte	Result
WB	RecProt	**N***
West†	HeLa	**N**
	MCF10A	**N**
	LCL57	**N**
WB	HeLa	**NT**
	MCF10A	**NT**
	LCL57	**NT**
IF	HeLa	**NT**
	MCF10A	**NT**
	LCL57	**NT**
SCWB	HeLa	**NT**
	MCF10A	**NT**
	LCL57	**NT**
IP	RecProt	**NT**
IP	HeLa	**NT**
	MCF10A	**NT**
	LCL57	**NT**
IHC		**N***
NCI60		**NT**

N – Negative, NT – Not Tested, * Data not shown
† Data shown are from Phospho-specificity Experiment

Endogenous Wes

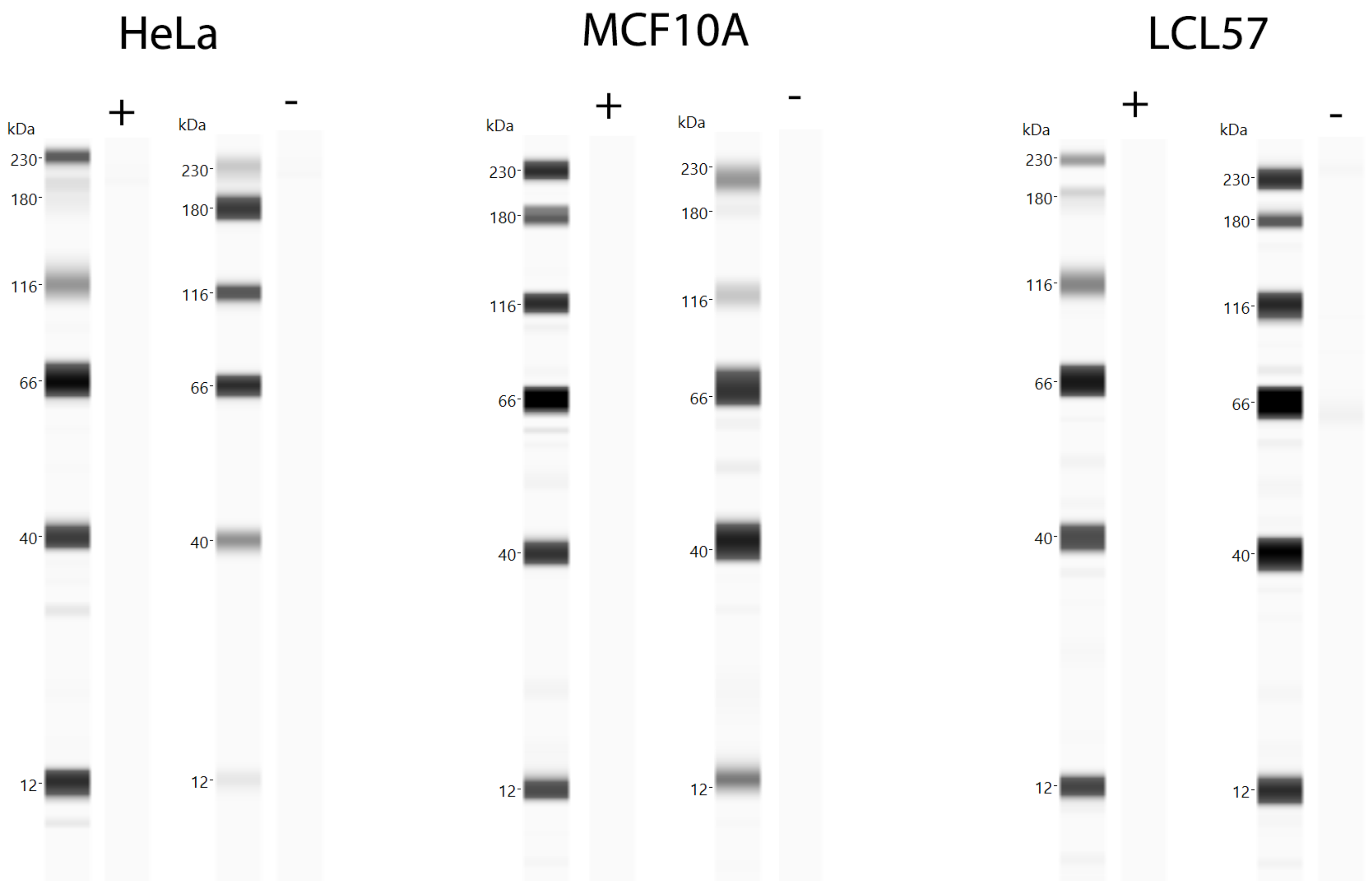

Expected molecular weight: 113 kDa

+ : irradiation treated cells
- : not treated cells

29. CPTC-RAD50-2

Technique	Analyte	Result
WB	RecProt	N*
West†	HeLa	N
	MCF10A	N
	LCL57	N
WB	HeLa	NT
	MCF10A	NT
	LCL57	NT
IF	HeLa	NT
	MCF10A	NT
	LCL57	NT
SCWB	HeLa	NT
	MCF10A	NT
	LCL57	NT
IP	RecProt	NT
IP	HeLa	NT
	MCF10A	NT
	LCL57	NT
IHC		N*
NCI60		NT

N – Negative, NT – Not Tested, * Data not shown
† Data shown are from Phospho-specificity Experiment.

Endogenous Wes

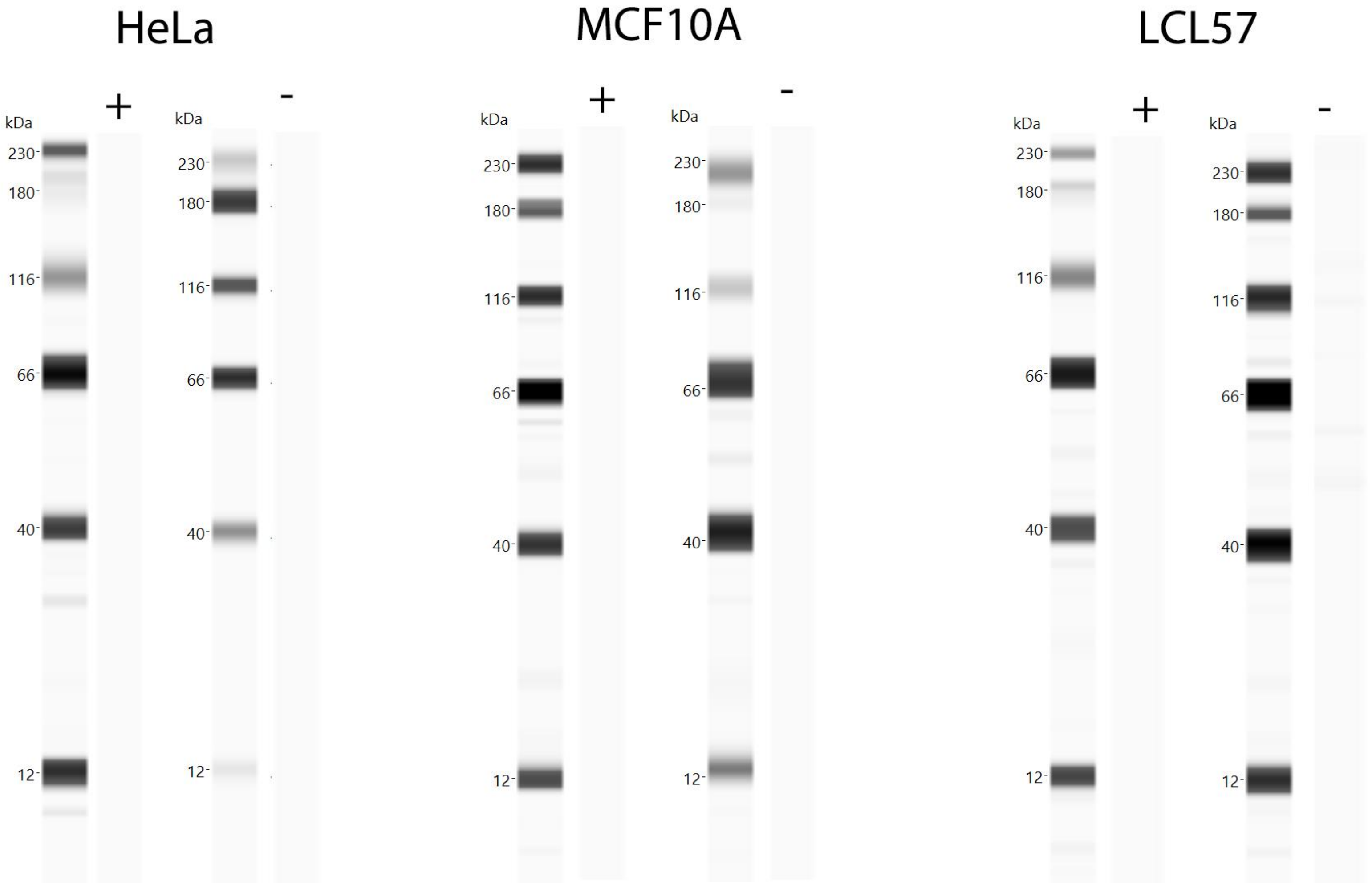

Expected molecular weight: 154, 155, 138 kDa

+ : irradiation treated cells
- : not treated cells

30. CPTC-RAD9A-1

Technique	Analyte	Result
WB	RecProt	**N***
West†	HeLa	**P**
	MCF10A	**N**
	LCL57	**N**
WB§	HeLa	**U**
	MCF10A	**N**
	LCL57	**U**
IF	HeLa	**NT**
	MCF10A	**NT**
	LCL57	**NT**
SCWB	HeLa	**NT**
	MCF10A	**NT**
	LCL57	**NT**
IP	RecProt	**NT**
IP	HeLa	**NT**
	MCF10A	**NT**
	LCL57	**NT**
IHC		**N***
NCI60		**NT**

P – Positive, N – Negative, NT – Not Tested, U – Undetermined, * Data not shown
† Data shown are from Phospho-specificity Experiment. § Data from Phospho-specificity Experiment are not shown.

Endogenous Wes

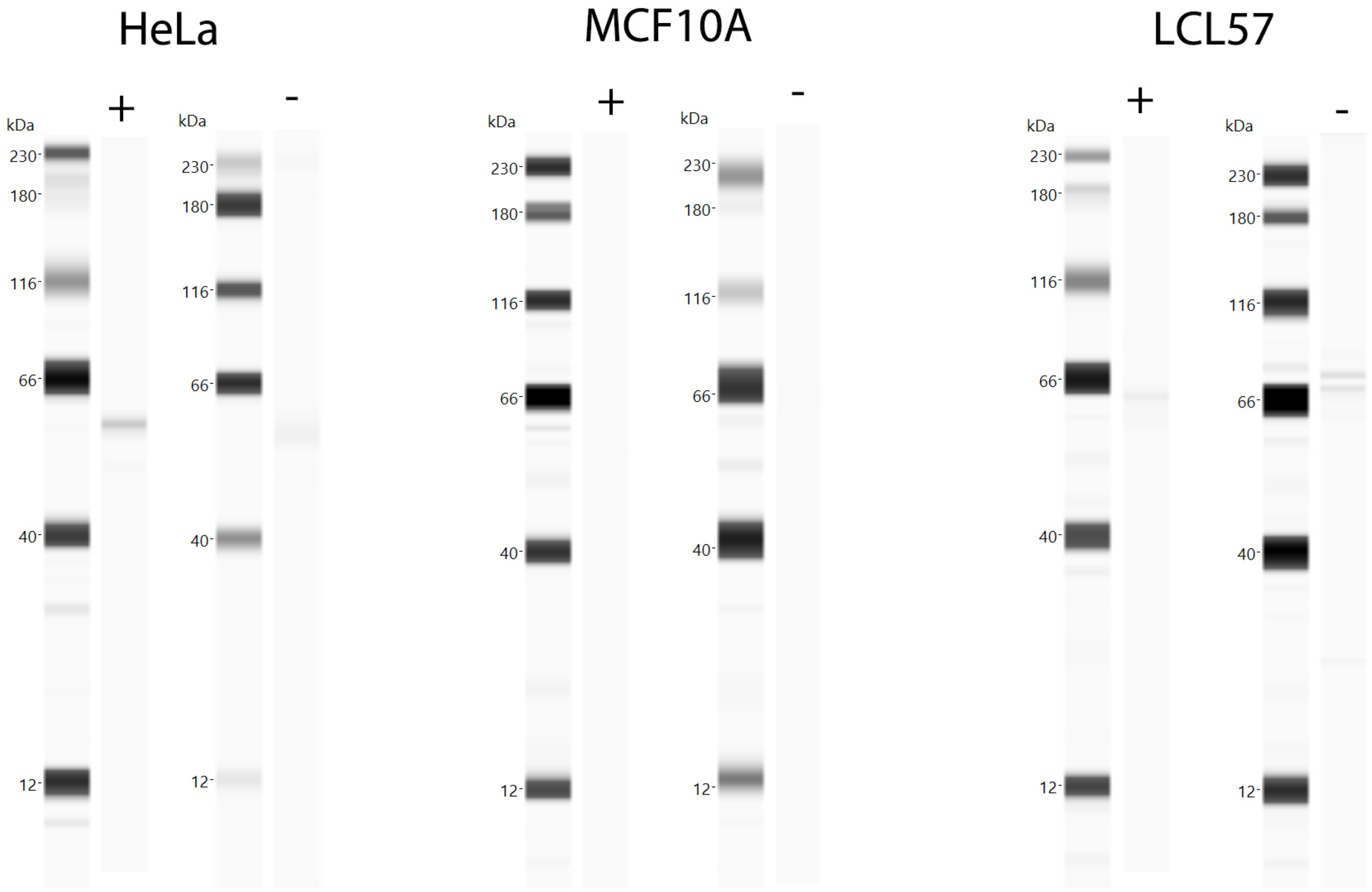

Expected molecular weight: 43 kDa

+ : irradiation treated cells
- : not treated cells

31. CPTC-RTF1-1

Technique	Analyte	Result
WB	RecProt	**N***
West†	HeLa	**N**
	MCF10A	**N**
	LCL57	**N**
WB	HeLa	**NT**
	MCF10A	**NT**
	LCL57	**NT**
IF	HeLa	**NT**
	MCF10A	**NT**
	LCL57	**NT**
SCWB	HeLa	**NT**
	MCF10A	**NT**
	LCL57	**NT**
IP	RecProt	**NT**
IP	HeLa	**NT**
	MCF10A	**NT**
	LCL57	**NT**
IHC		**P**
NCI60		**P**

N – Negative, NT – Not Tested, * Data not shown
† Data shown are from Phospho-specificity Experiment.

Endogenous Wes

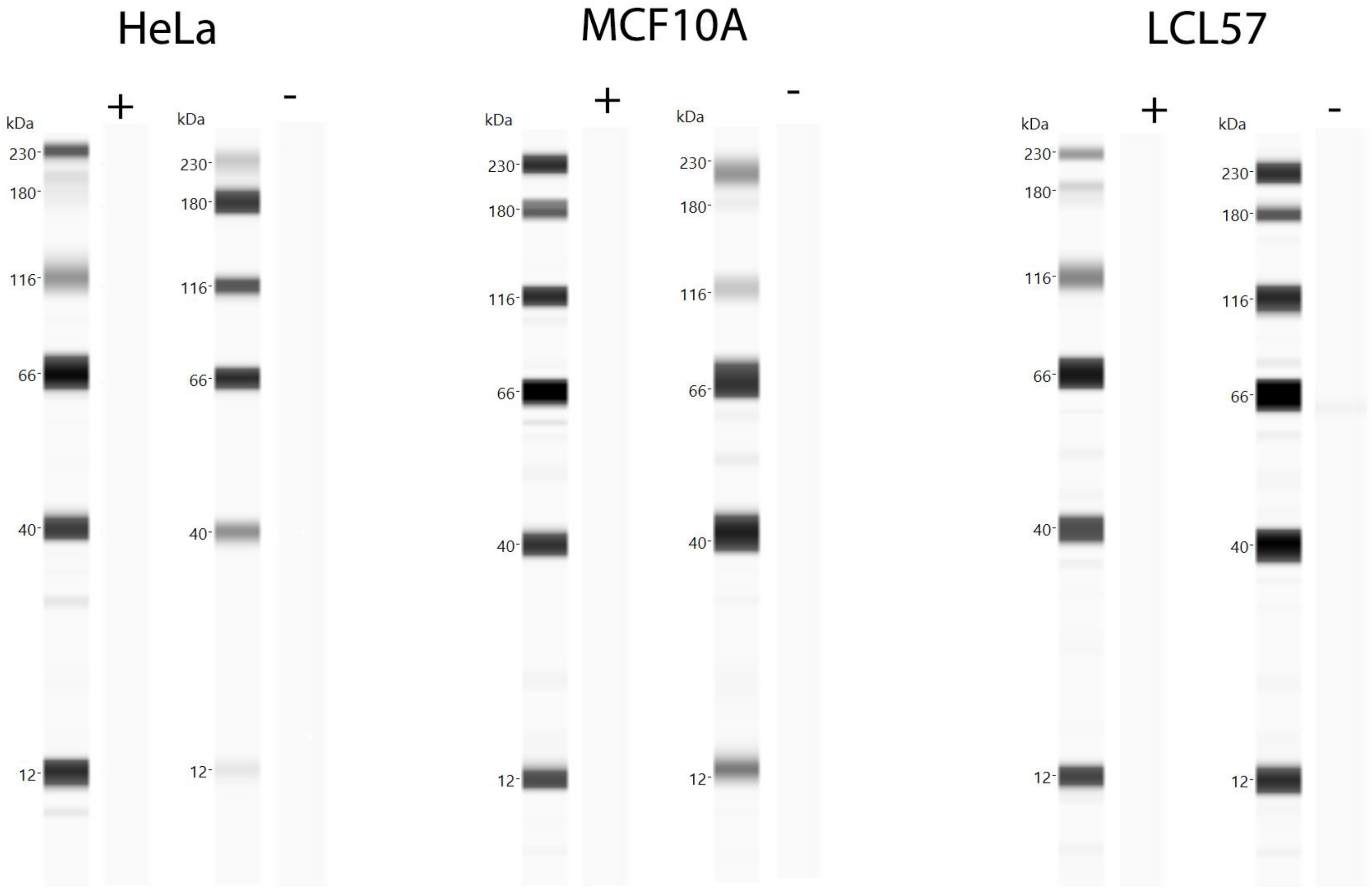

Expected molecular weight: 66 kDa

+ : irradiation treated cells
- : not treated cells

CPTC- RTF1-1

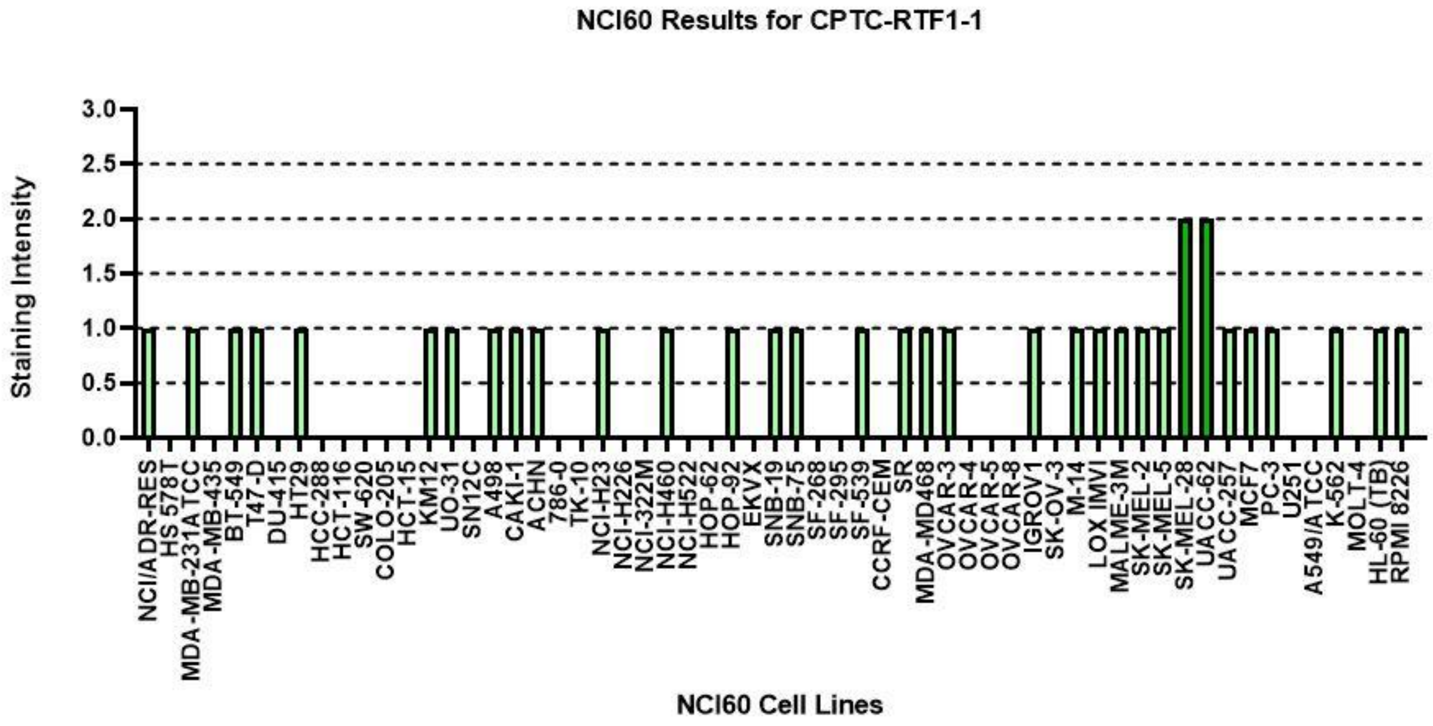
NCI60 Results for CPTC-RTF1-1
Staining Intensity
3.0
2.5
2.0
1.5
1.0
0.5
0.0
NCI/ADR-RES
HS 578T
MDA-MB-231ATCC
MDA-MB-435
BT-549
T47-D
DU-415
HT29
HCC-288
HCT-116
SW-620
COLO-205
HCT-15
KM12
UO-31
SN12C
A498
CAKI-1
ACHN
786-0
TK-10
NCI-H23
NCI-H226
NCI-322M
NCI-H460
NCI-H522
HOP-62
HOP-92
EKVX
SNB-19
SNB-75
SF-268
SF-295
SF-539
CCRF-CEM
SR
MDA-MD468
OVCAR-3
OVCAR-4
OVCAR-5
OVCAR-8
IGROV1
SK-OV-3
M-14
LOX IMVI
MALME-3M
SK-MEL-2
SK-MEL-5
SK-MEL-28
UACC-62
UACC-257
MCF7
PC-3
U251
A549/ATCC
K-562
MOLT-4
HL-60 (TB)
RPMI 8226
NCI60 Cell Lines

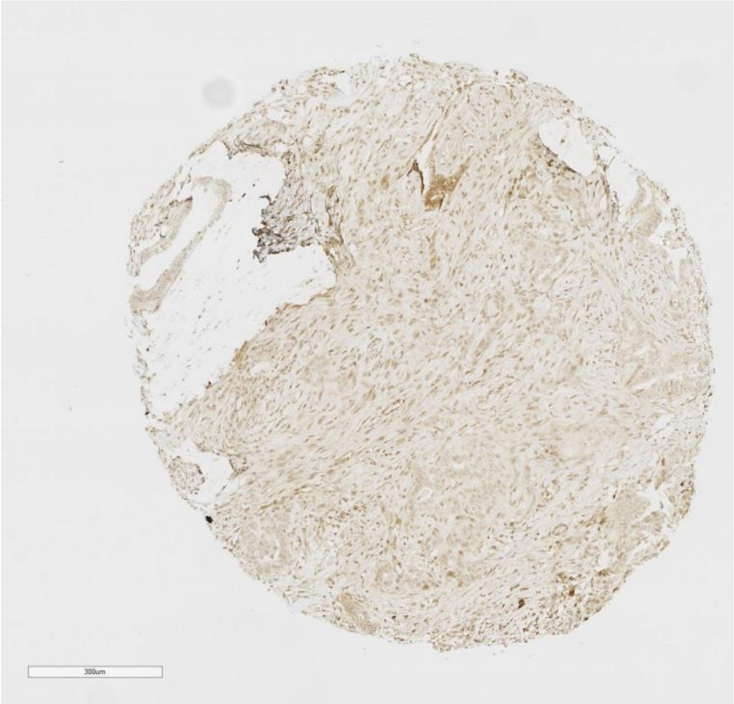

32. CPTC-RTF1-2

Technique	Analyte	Result
WB	RecProt	**N***
West†	HeLa	**N**
	MCF10A	**N**
	LCL57	**N**
WB	HeLa	**NT**
	MCF10A	**NT**
	LCL57	**NT**
IF	HeLa	**NT**
	MCF10A	**NT**
	LCL57	**NT**
SCWB	HeLa	**NT**
	MCF10A	**NT**
	LCL57	**NT**
IP	RecProt	**NT**
IP	HeLa	**NT**
	MCF10A	**NT**
	LCL57	**NT**
IHC		**P**
NCI60		**P**

N – Negative, NT – Not Tested, * Data not shown

† Data shown are from Phospho-specificity Experiment.

Endogenous Wes

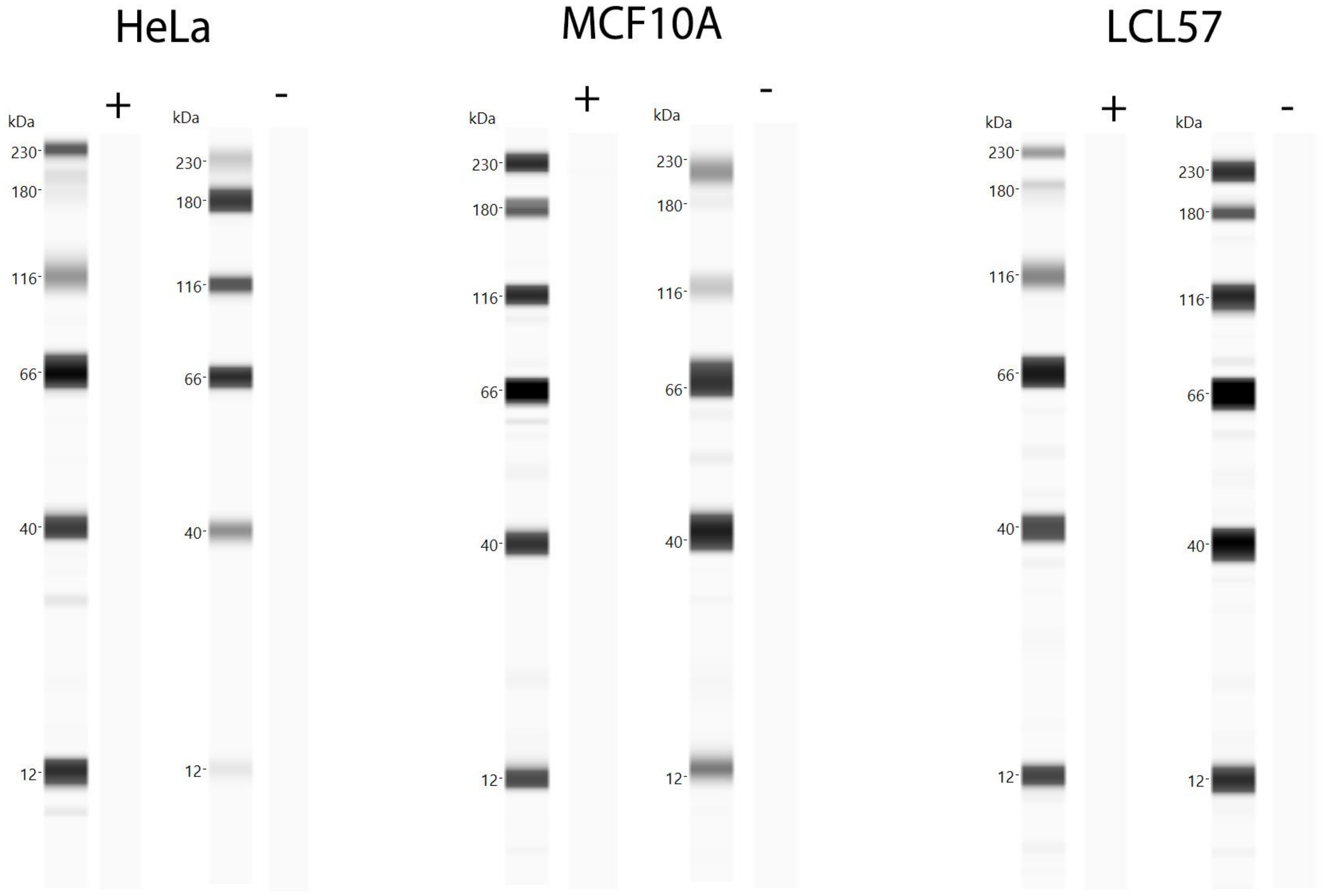

Expected molecular weight: 66 kDa

+ : irradiation treated cells
- : not treated cells

CPTC-RTF1-2

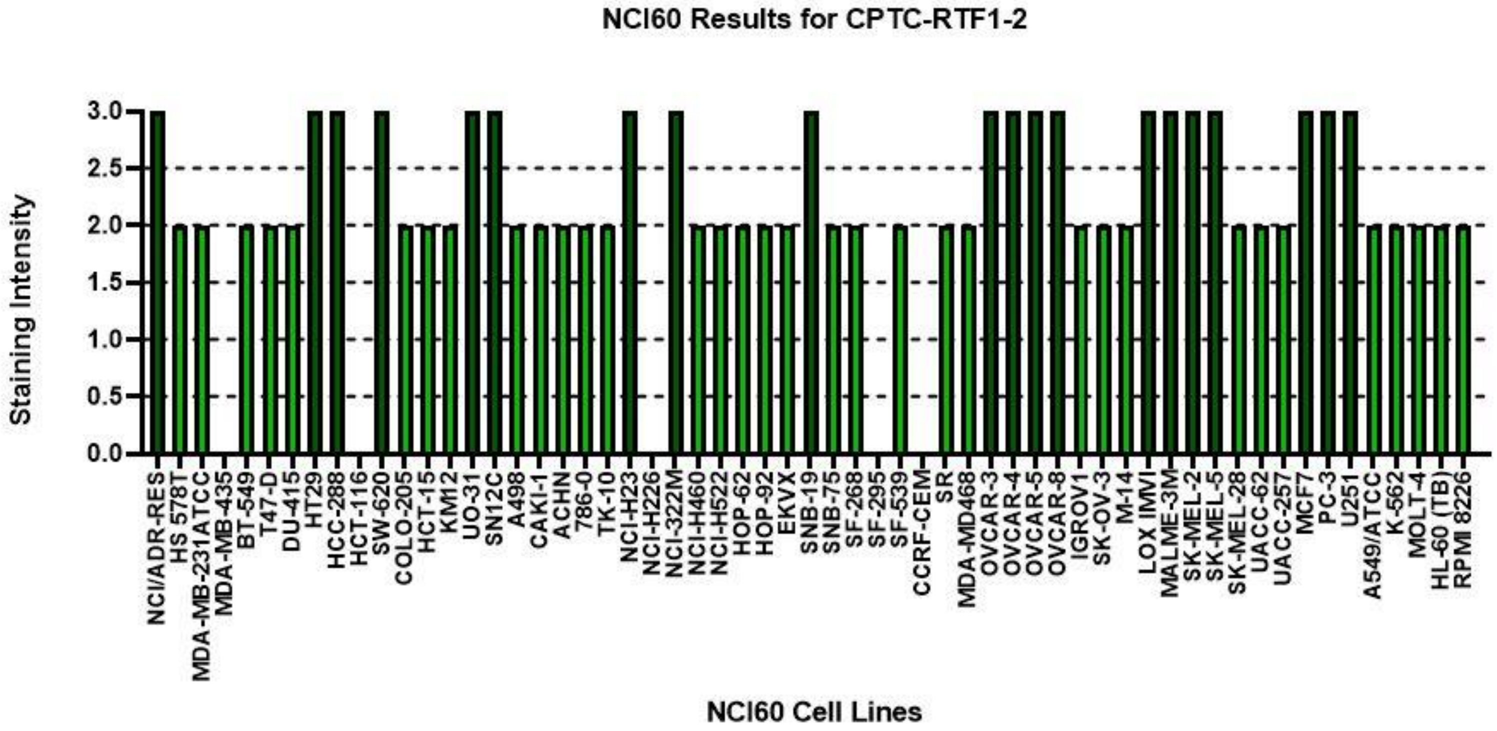
NCI60 Results for CPTC-RTF1-2
Staining Intensity
3.0
2.5
2.0
1.5
1.0
0.5
0.0
NCI/ADR-RES
HS 578T
MDA-MB-231ATCC
MDA-MB-435
BT-549
T47-D
DU-415
HT29
HCC-288
HCT-116
SW-620
COLO-205
HCT-15
KM12
UO-31
SN12C
A498
CAKI-1
ACHN
786-0
TK-10
NCI-H23
NCI-H226
NCI-322M
NCI-H460
NCI-H522
HOP-62
HOP-92
EKVX
SNB-19
SNB-75
SF-268
SF-295
SF-539
CCRF-CEM
SR
MDA-MD468
OVCAR-3
OVCAR-4
OVCAR-5
OVCAR-8
IGROV1
SK-OV-3
M-14
LOX IMVI
MALME-3M
SK-MEL-2
SK-MEL-5
SK-MEL-28
UACC-62
UACC-257
MCF7
PC-3
U251
A549/ATCC
K-562
MOLT-4
HL-60 (TB)
RPMI 8226
NCI60 Cell Lines

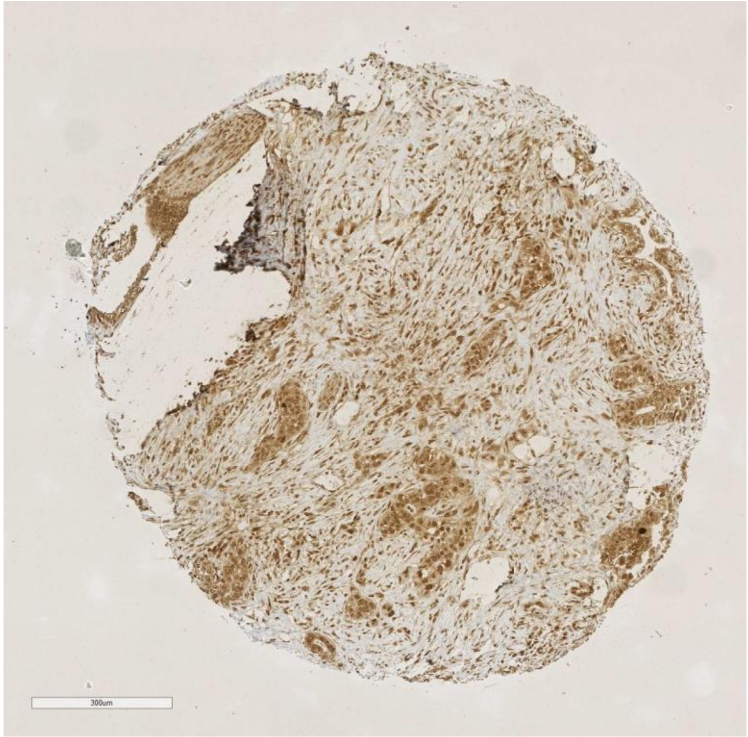

33. CPTC-SAAL1-1

Technique	Analyte	Result
WB	RecProt	**P**
Wes	HeLa	**U**
	MCF10A	**P**
	LCL57	**P**
WB	HeLa	**N**
	MCF10A	**N**
	LCL57	**P**
IF	HeLa	**N†**
	MCF10A	**N†**
	LCL57	**P**
SCWB†	HeLa	**N**
	MCF10A	**N**
	LCL57	**N**
IP*	RecProt	**P**
IP*	HeLa	**N**
	MCF10A	**N**
	LCL57	**N**
IHC		**N†**
NCI60		**NT**

P – Positive, N – Negative, U – Undetermined, *Please refer to tables for results, † Data not shown

WB against commercial recombinant protein

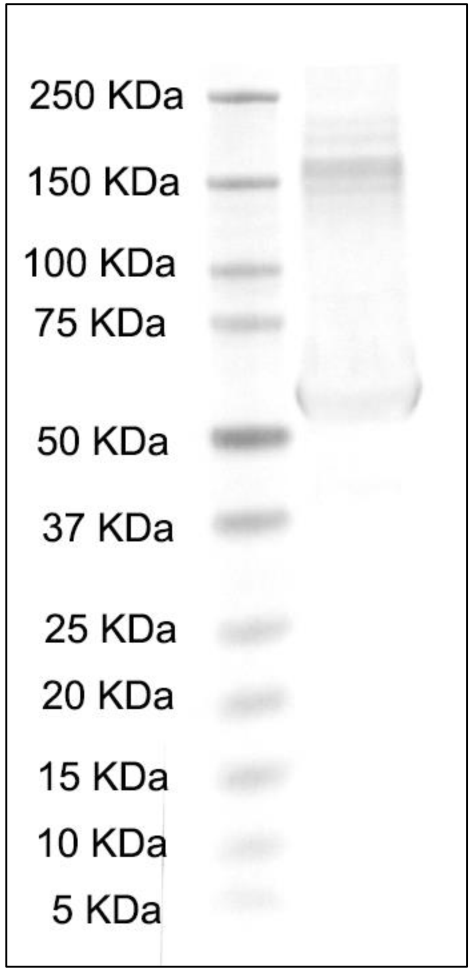

Ag Vendor: Origene

Cat #: TP304551

Description: Recombinant protein of human serum amyloid A-like 1 (SAAL1)

Predicted MW: 53.4 kDa

Endogenous WB

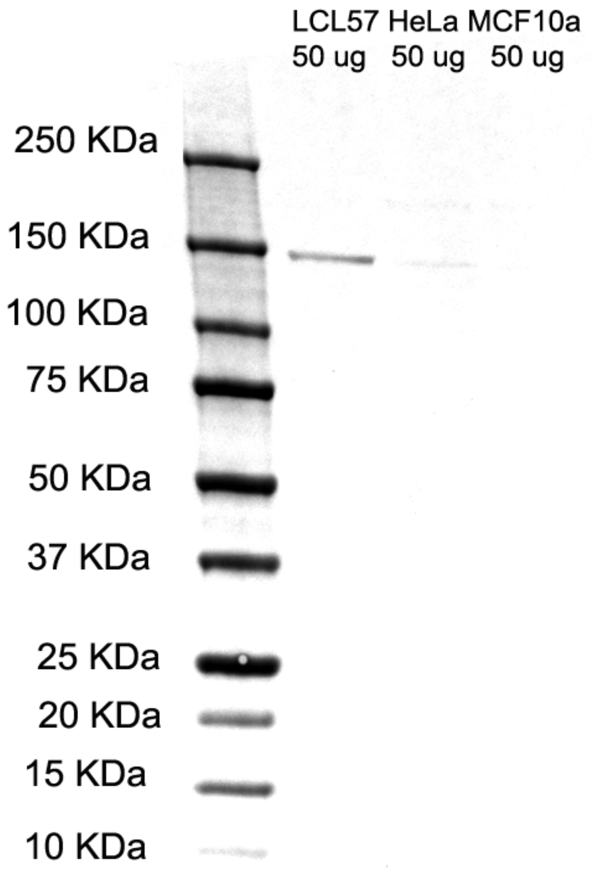

Endogenous Wes

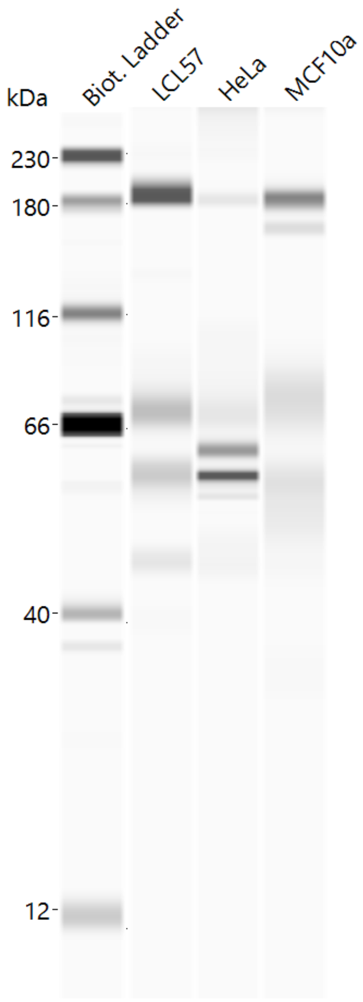

Immunofluorescence - LCL57

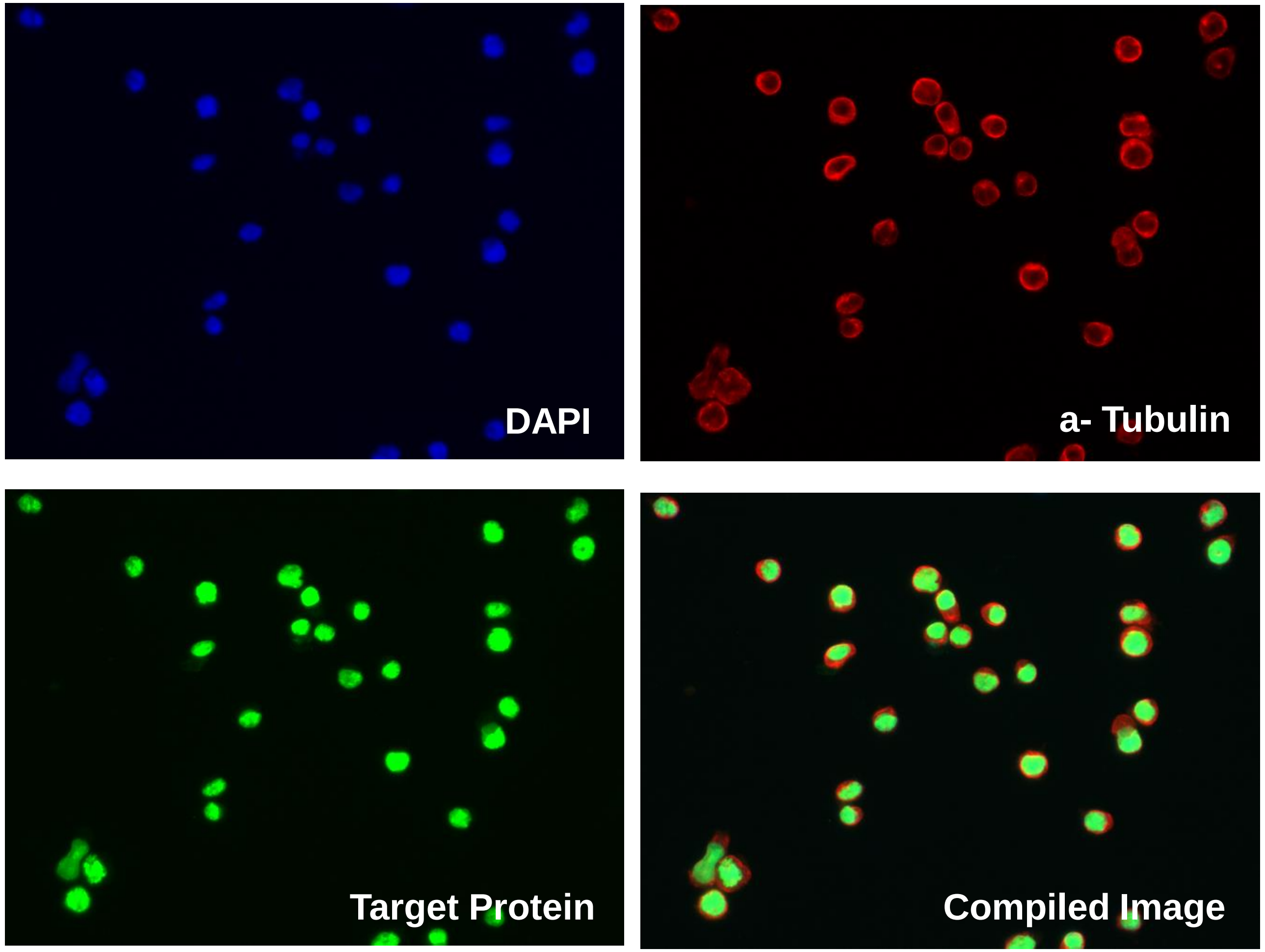

Subcellular Location: Nucleus

34. CPTC-TNFRSF17-1

Technique	Analyte	Result
WB	RecProt	N*
West†	HeLa	N
	MCF10A	U
	LCL57	N
WB§	HeLa	N
	MCF10A	N
	LCL57	N
IF	HeLa	NT
	MCF10A	NT
	LCL57	NT
SCWB	HeLa	NT
	MCF10A	NT
	LCL57	NT
IP	RecProt	NT
IP	HeLa	NT
	MCF10A	NT
	LCL57	NT
IHC		N*
NCI60		NT

U – Uncertain, N – Negative, NT – Not Tested, * Data not shown
† Data shown are from Phospho-specificity Experiment. § Data from Phospho-specificity Experiment are not shown.

Endogenous Wes

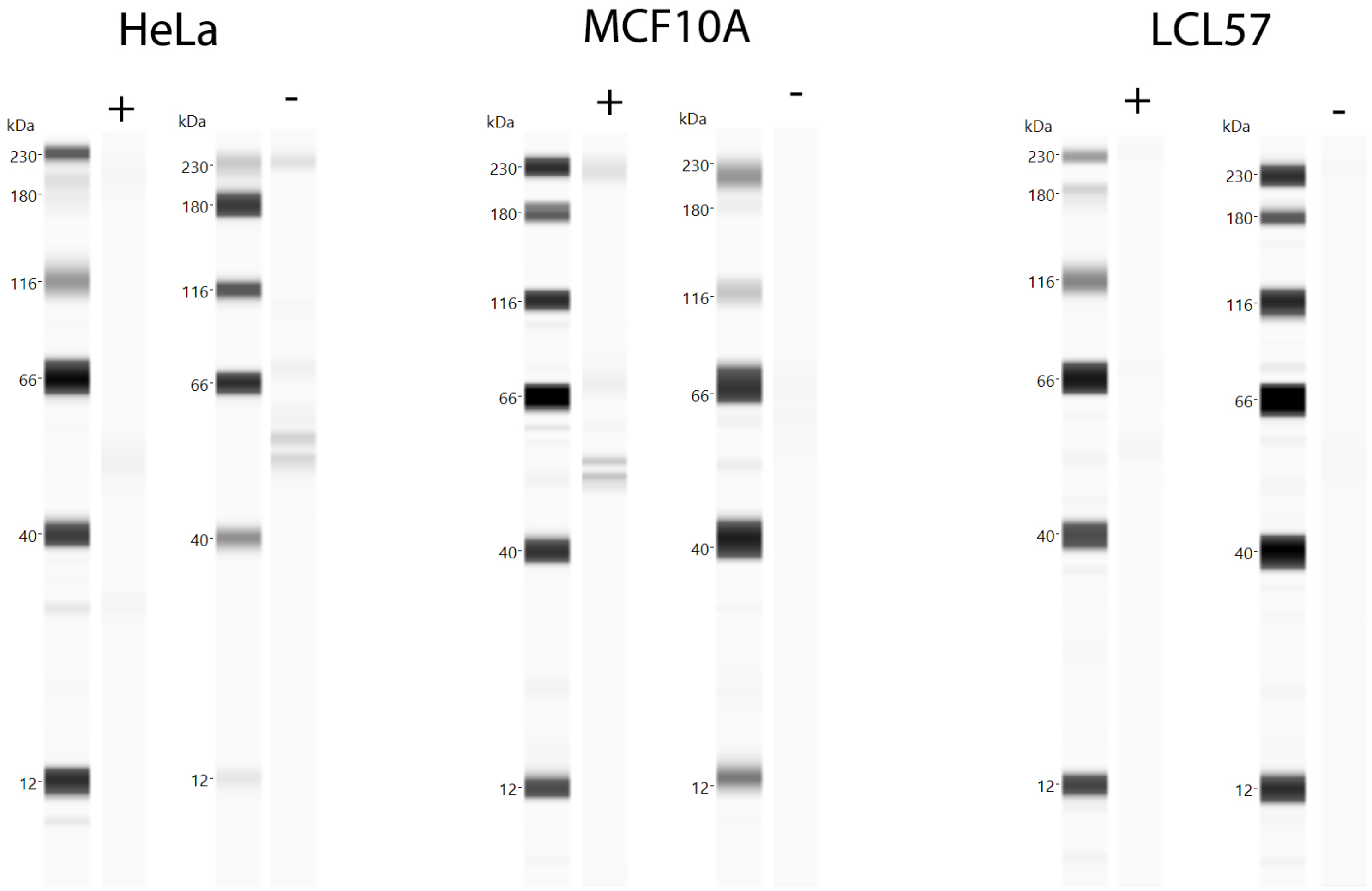

Expected molecular weight: 20, 15 kDa

+ : irradiation treated cells
- : not treated cells

35. CPTC-TP53-1

Technique	Analyte	Result
WB	RecProt	P
Wes	HeLa	P
	MCF10A	N
	LCL57	N
WB	HeLa	U
	MCF10A	U
	LCL57	U
IF	HeLa	P
	MCF10A	P
	LCL57	P
SCWB†	HeLa	N
	MCF10A	N
	LCL57	N
IP*	RecProt	P
IP*	HeLa	N
	MCF10A	N
	LCL57	N
IHC		N†
NCI60		NT

P – Positive, N – Negative, U – Undetermined, * Please refer to tables for results, † Data not shown

WB against commercial recombinant protein

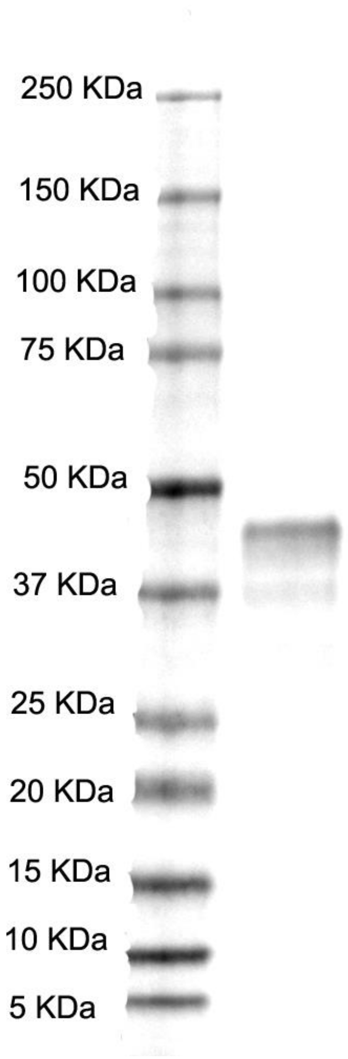

Ag Vendor: Origene

Cat #: TP300003

Description: Recombinant protein of human tumor protein p53 (TP53), transcript variant 1

Predicted MW 43.5 KDa

Endogenous WB

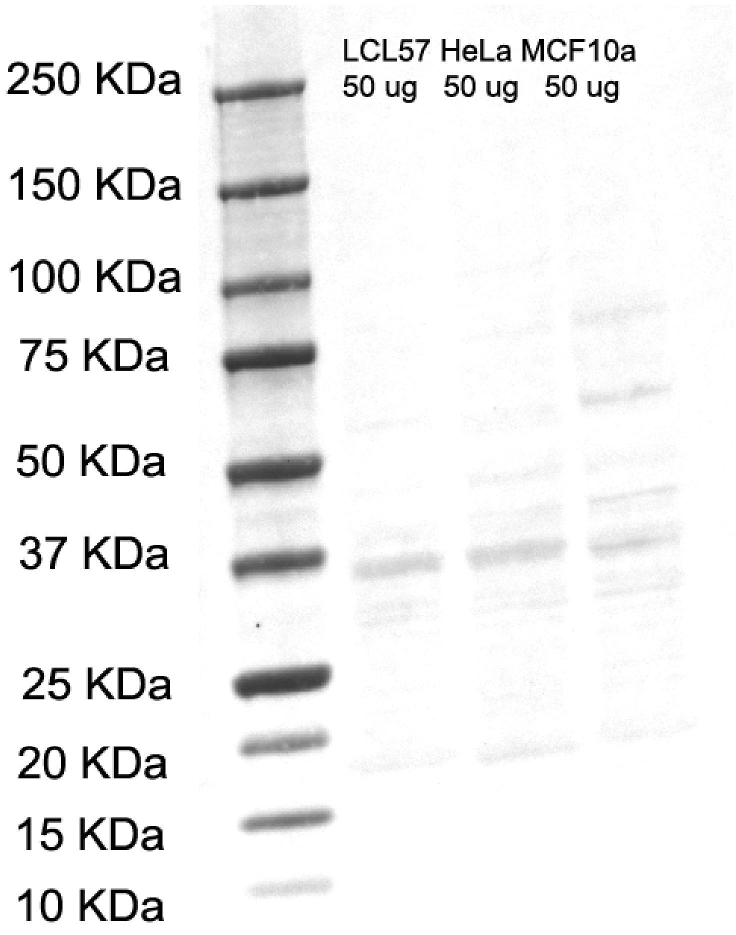

Endogenous Wes

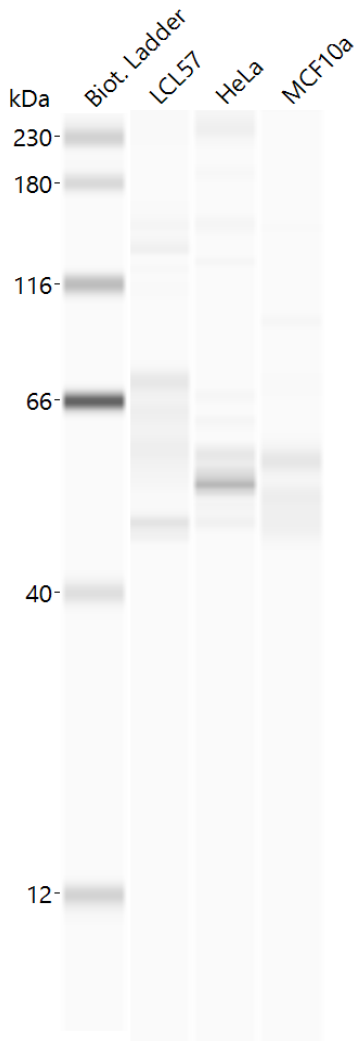

Immunofluorescence – HeLa

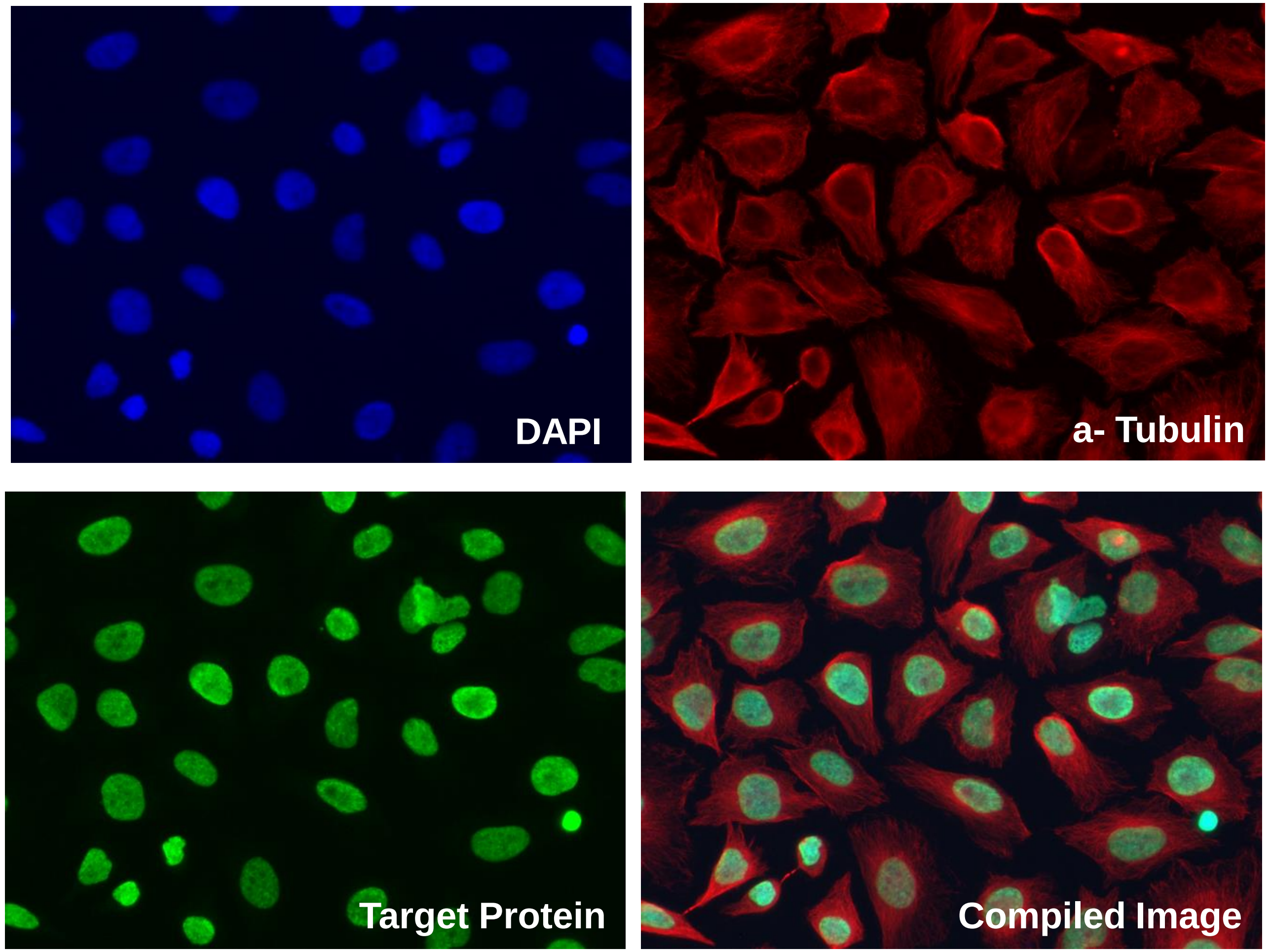

Subcellular location: Cytoskeleton, Mitochondrion, Nucleus, Endoplasmic reticulum, Other

Immunofluorescence – MCF10A

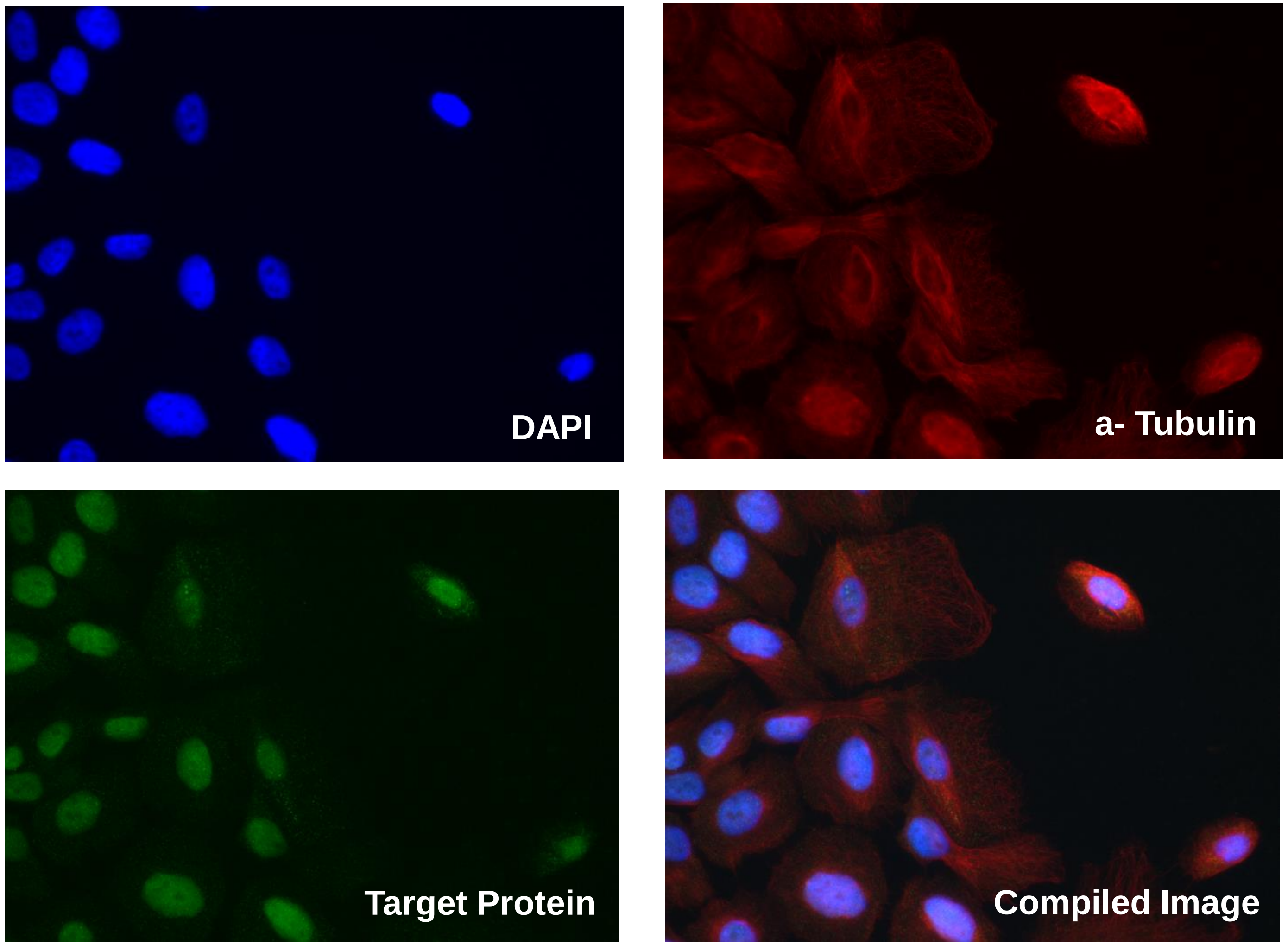

Subcellular location: Cytoskeleton, Mitochondrion, Nucleus, Endoplasmic reticulum, Other

Immunofluorescence – LCL57

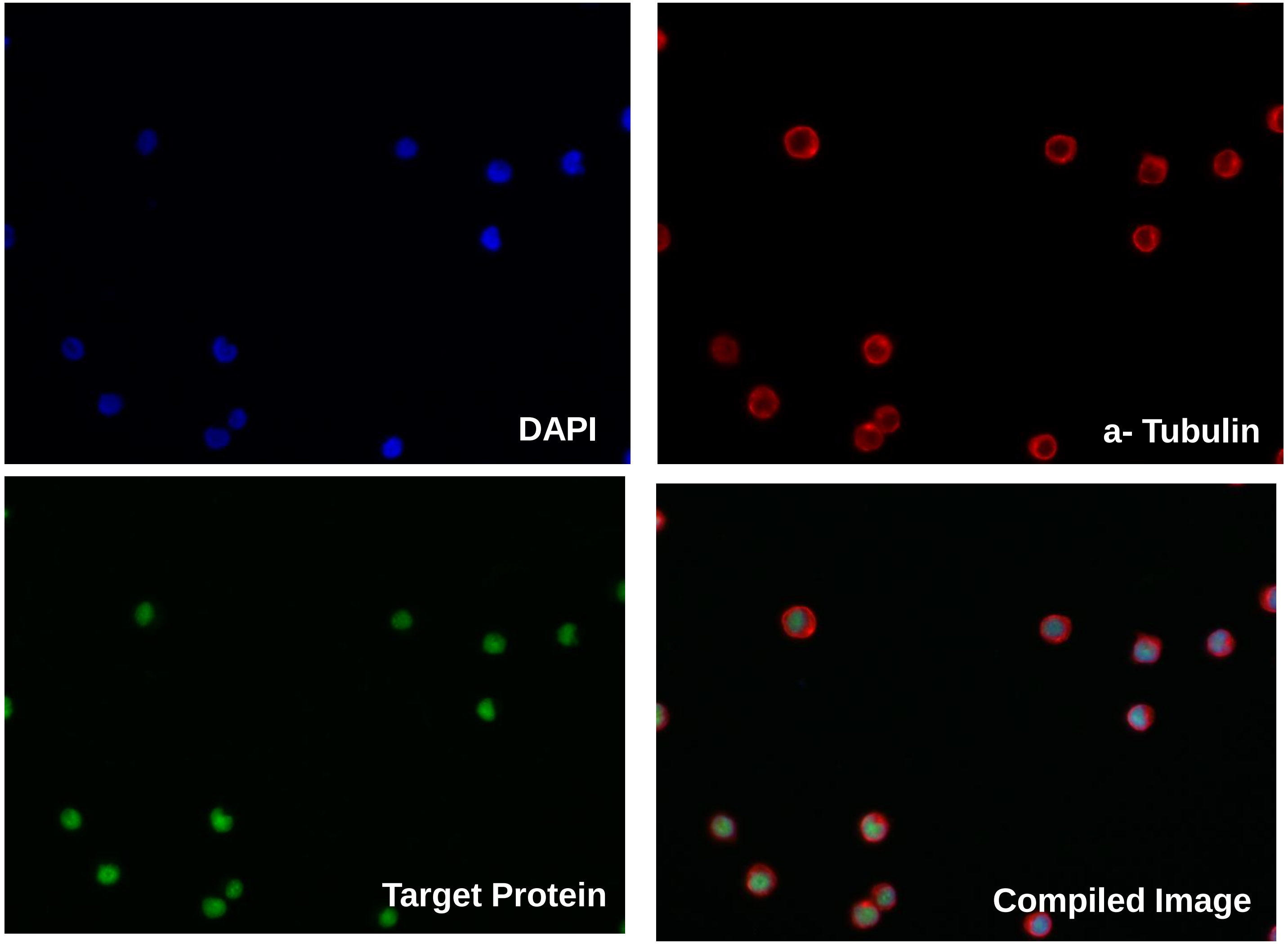

Subcellular location: Cytoskeleton, Mitochondrion, Nucleus, Endoplasmic reticulum, Other

36. CPTC-TP53-2

Technique	Analyte	Result
WB	RecProt	P
Wes	HeLa	P
	MCF10A	P
	LCL57	P
WB	HeLa	P
	MCF10A	P
	LCL57	P
IF	HeLa	P
	MCF10A	P
	LCL57	P
SCWB	HeLa	P
	MCF10A	P
	LCL57	P
IP*	RecProt	P
IP*	HeLa	N
	MCF10A	N
	LCL57	N
IHC		P
NCI60		P

P – Positive, N – Negative, *Please refer to tables for results

WB against commercial recombinant protein

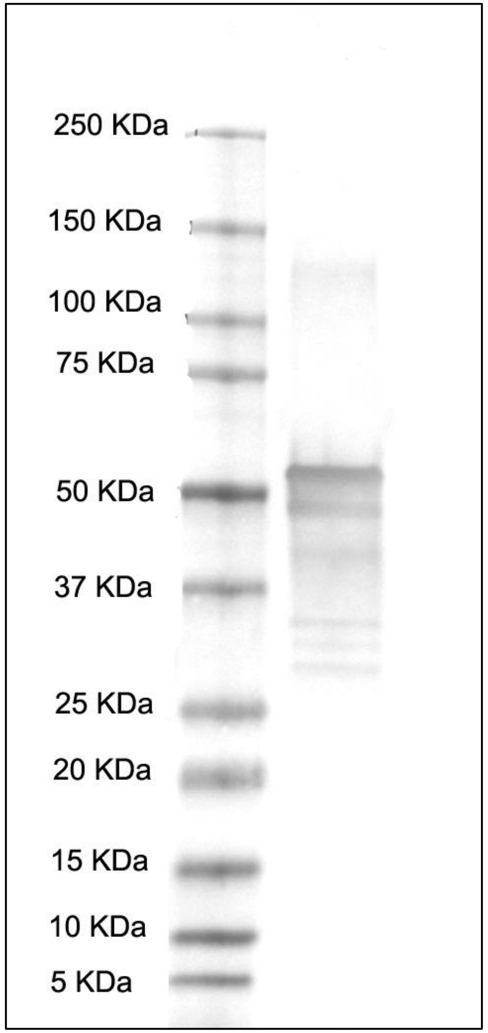

Ag Vendor: Origene

Cat #: TP300003

Description: Recombinant protein of human tumor protein p53 (TP53), transcript variant 1

Predicted MW 43.5 KDa

Endogenous WB

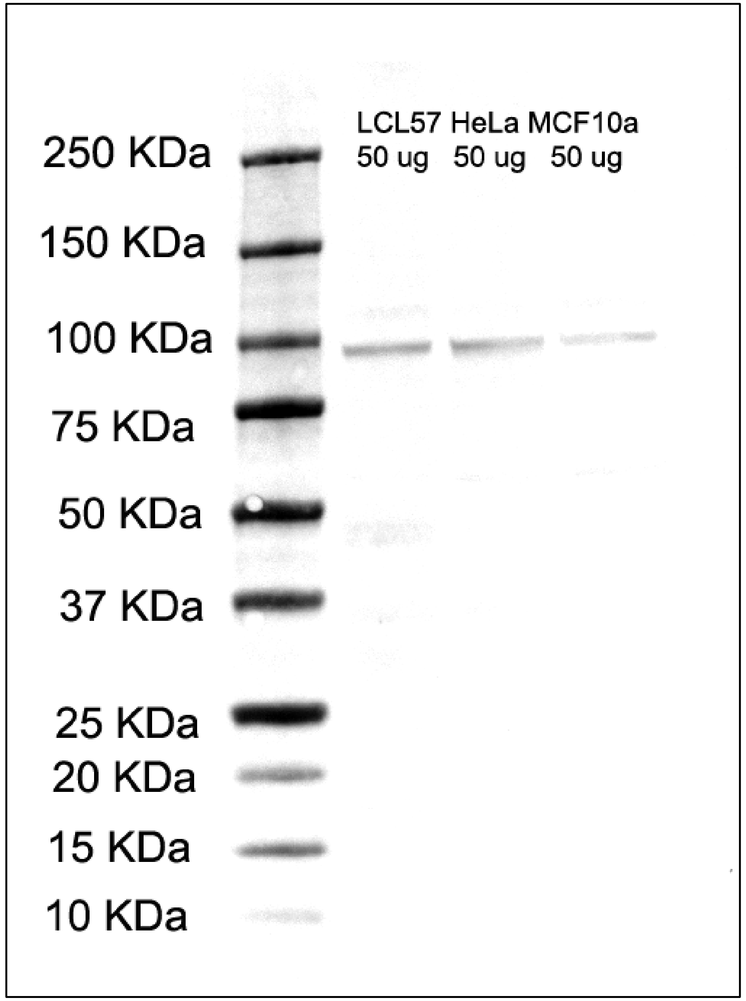

Endogenous Wes

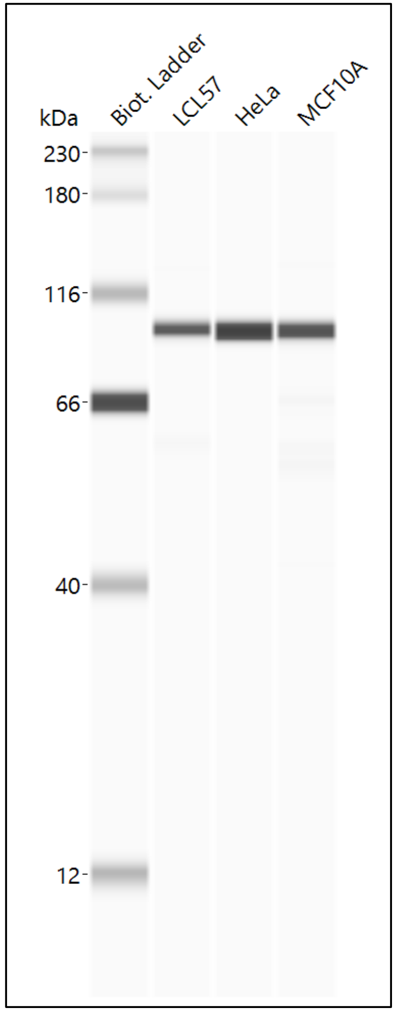

Immunofluorescence – HeLa

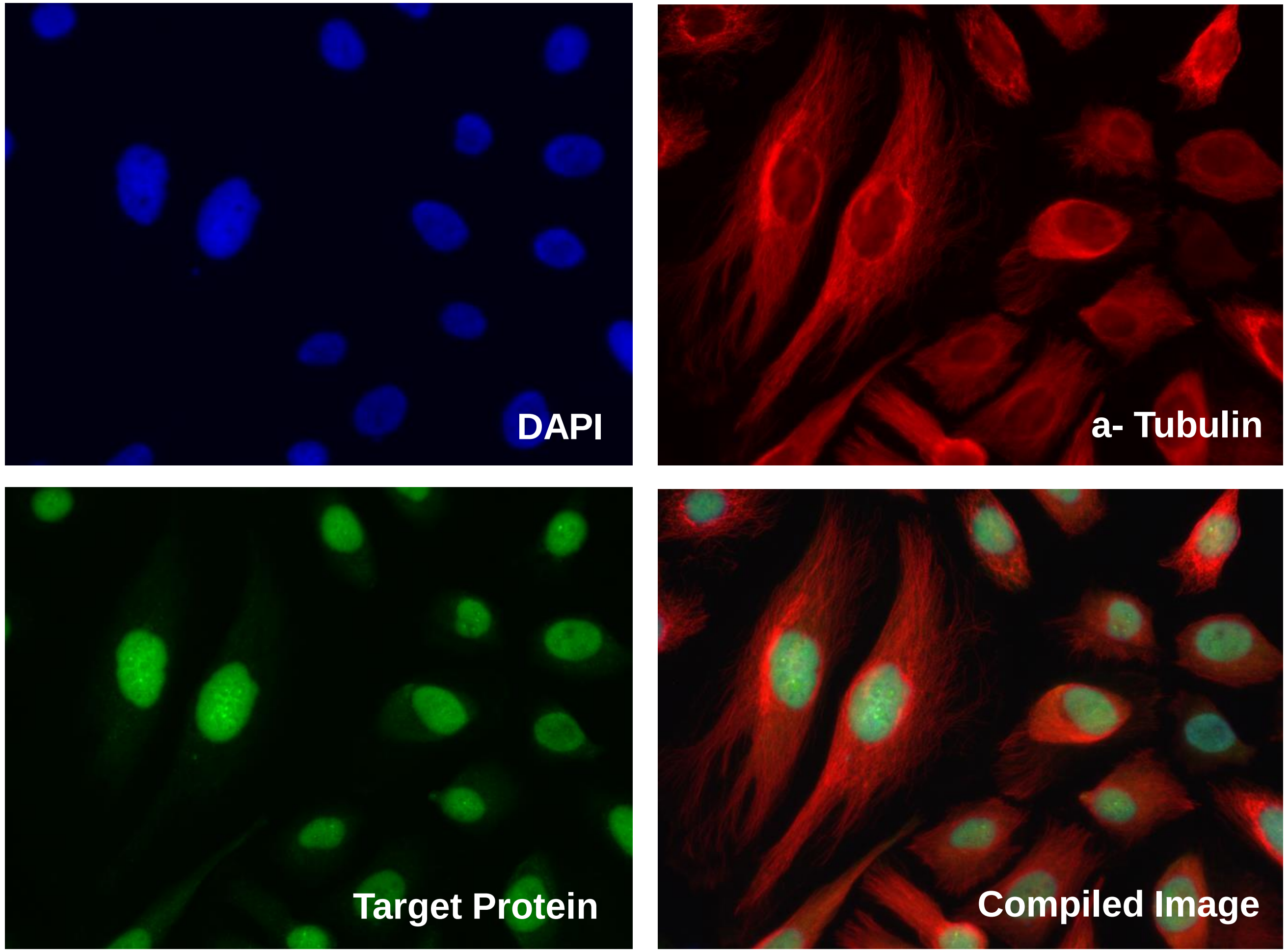

Subcellular location: Cytoskeleton, Mitochondrion, Nucleus, Endoplasmic reticulum, Other

Immunofluorescence – MCF10A

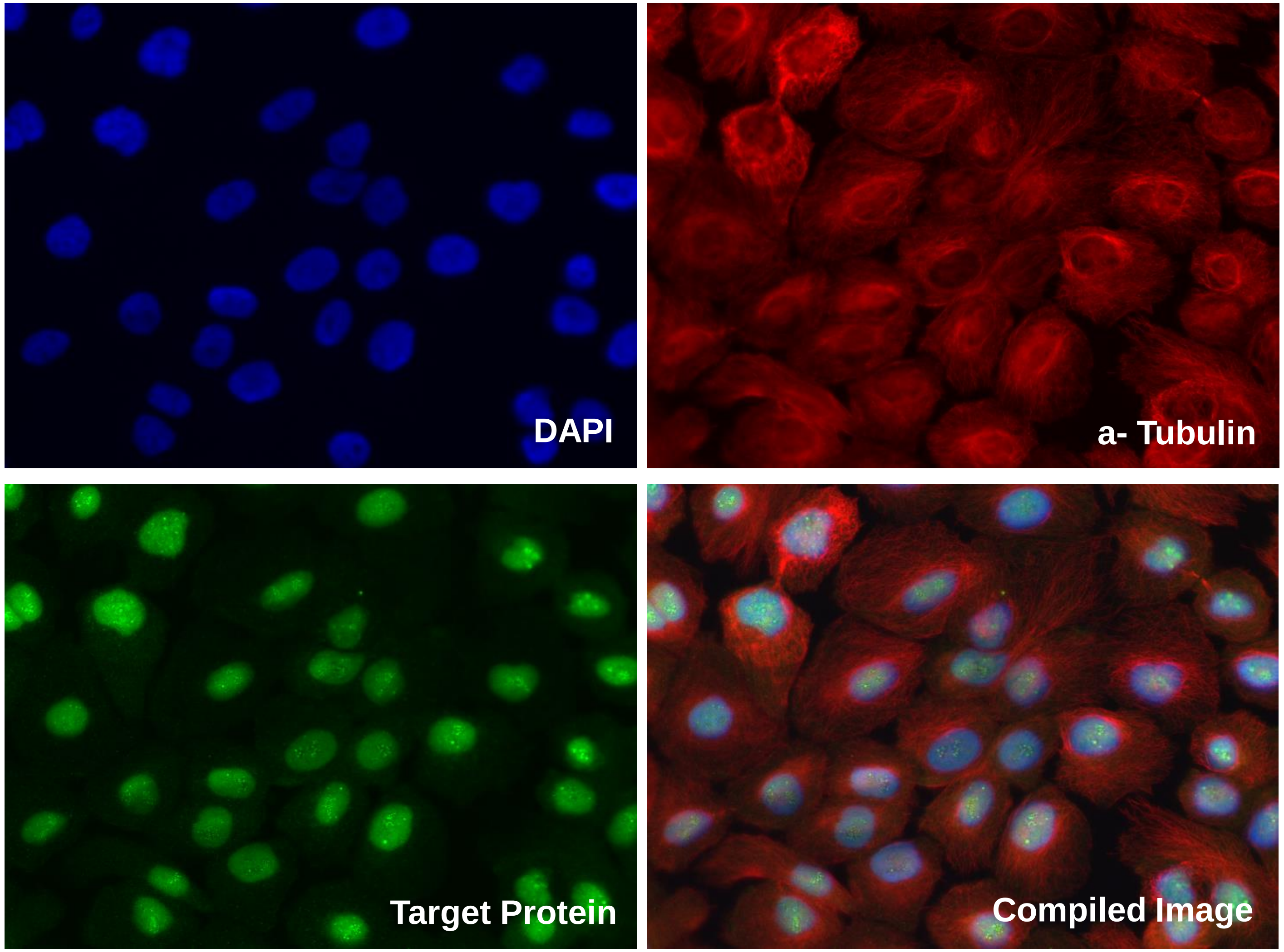

Subcellular location: Cytoskeleton, Mitochondrion, Nucleus, Endoplasmic reticulum, Other

Immunofluorescence – LCL57

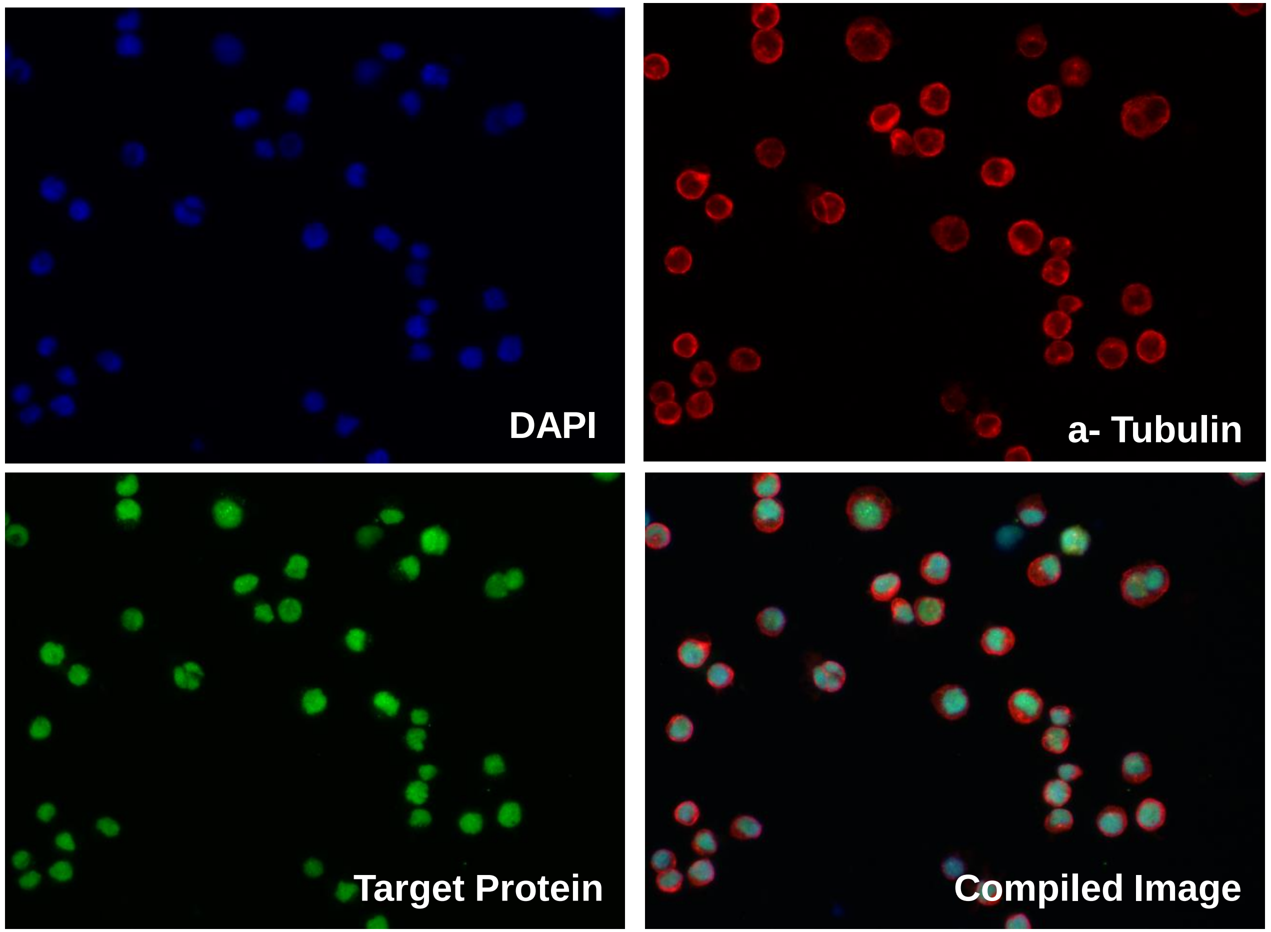

Subcellular location: Cytoskeleton, Mitochondrion, Nucleus, Endoplasmic reticulum, Other

Single Cell Western Blot

A.

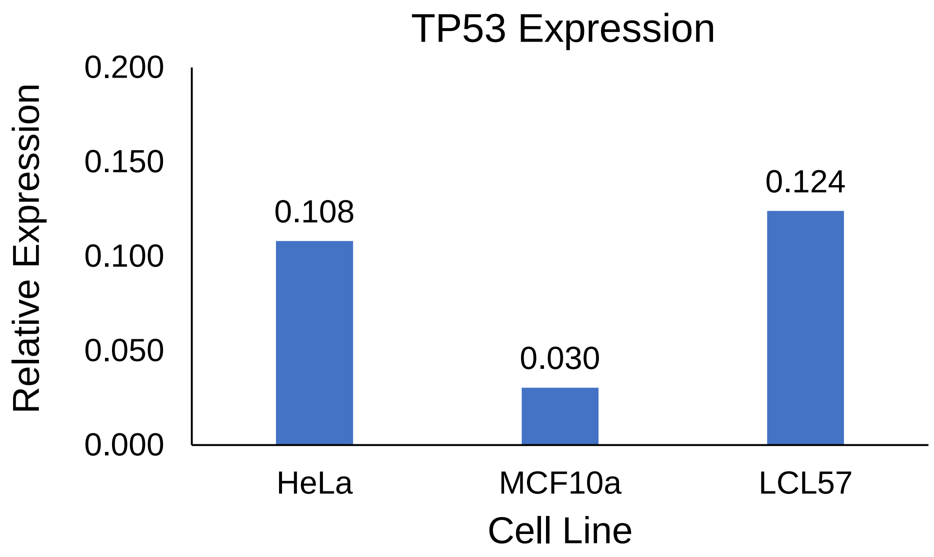

B.

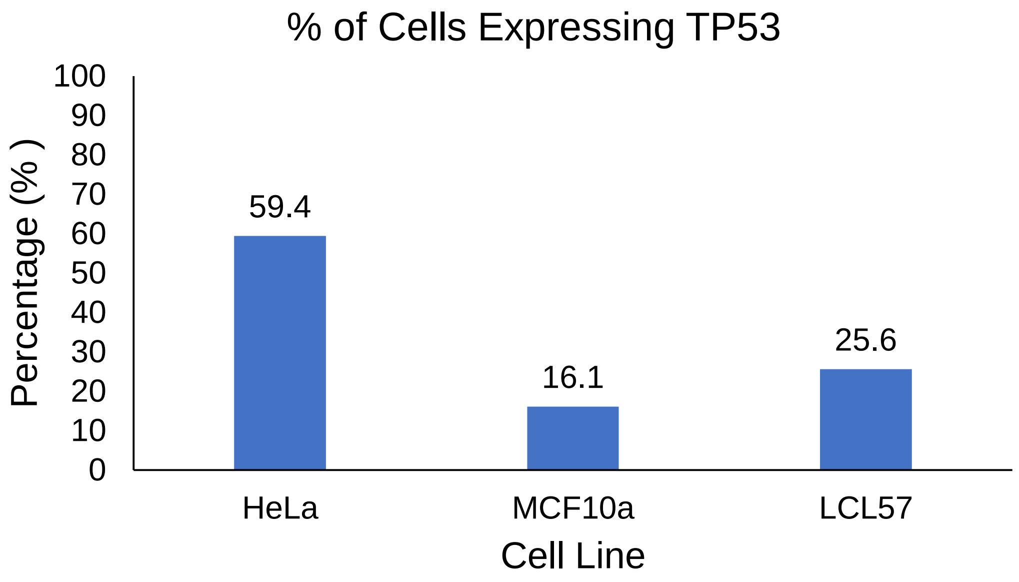

C.

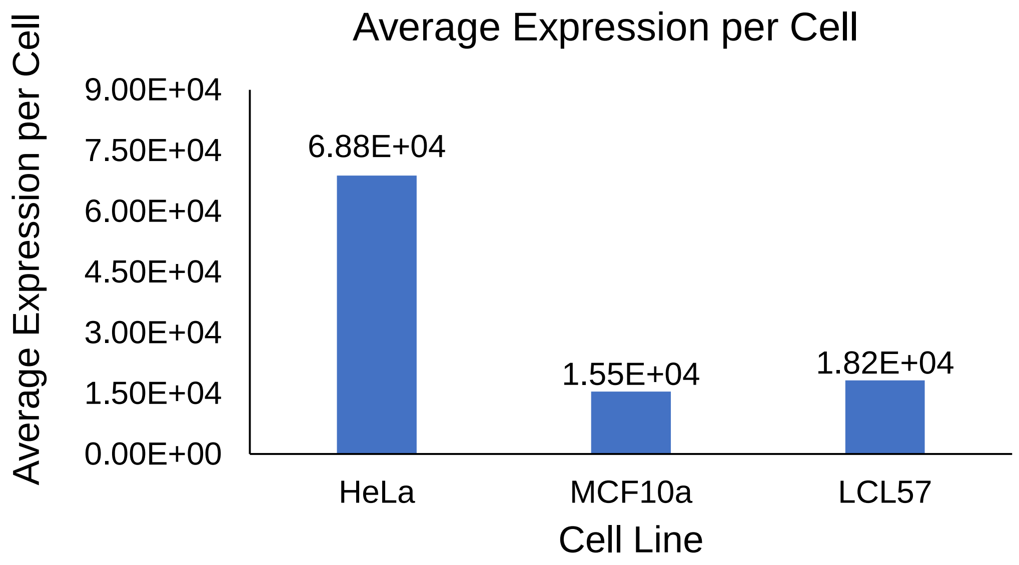

Single cell western blot data for TP53 (44 kDa band). Relative expression of total TP53 (44 kDa) in HeLa, MCF10A, and LCL57 cells (A). Percentage of cells that express TP53 (B). Average expression of TP53 protein per cell (C). All data is normalized to β-tubulin expression.

CPTC-TP53-2

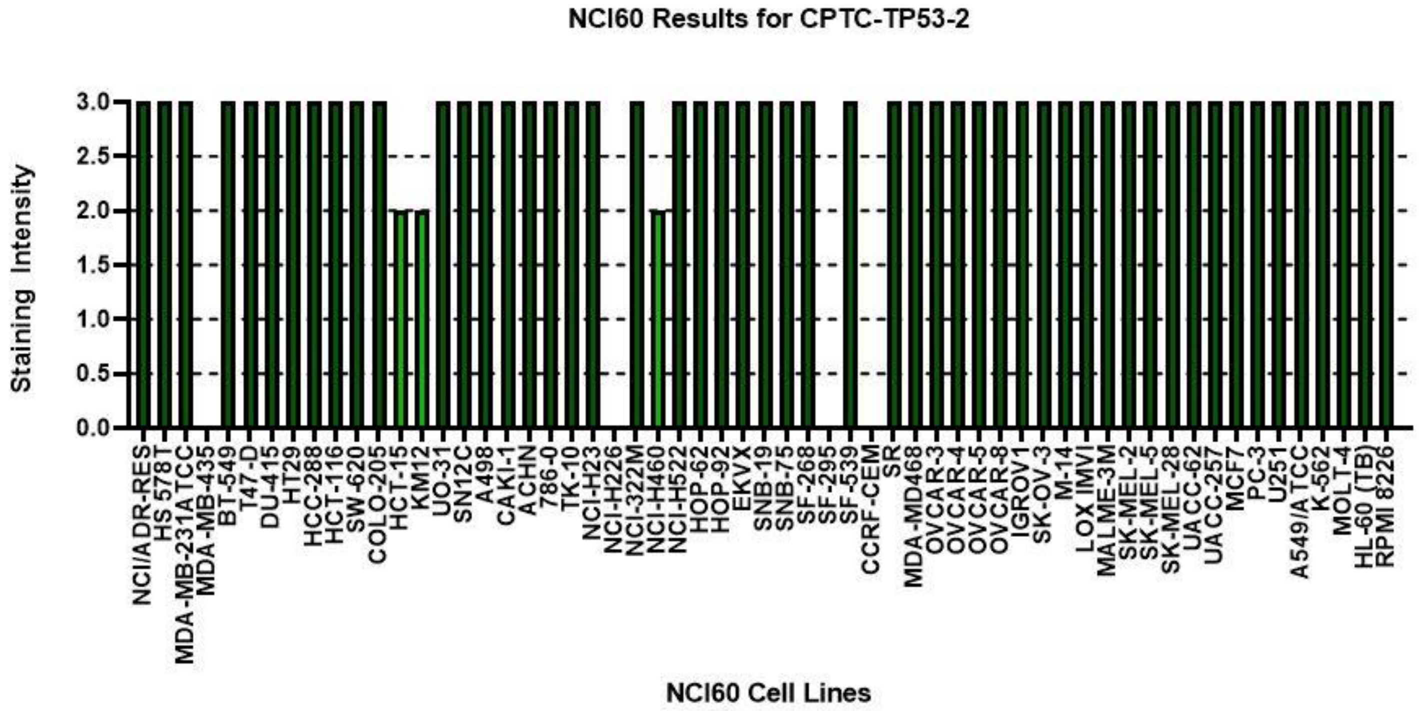

NCI60 Results for CPTC-TP53-2
Staining Intensity
3.0
2.5
2.0
1.5
1.0
0.5
0.0
NCI/ADR-RES
HS 578T
MDA-MB-231ATCC
MDA-MB-435
BT-549
T47-D
DU-415
HT29
HCC-288
HCT-116
SW-620
COLO-205
HCT-15
KM12
UO-31
SN12C
A498
CAKI-1
ACHN
786-0
TK-10
NCI-H23
NCI-H226
NCI-322M
NCI-H460
NCI-H522
HOP-62
HOP-92
EKVX
SNB-19
SNB-75
SF-268
SF-295
SF-539
CCRF-CEM
SR
MDA-MD468
OVCAR-3
OVCAR-4
OVCAR-5
OVCAR-8
IGROV1
SK-OV-3
M-14
LOX IMVI
MALME-3M
SK-MEL-2
SK-MEL-5
SK-MEL-28
UACC-62
UACC-257
MCF7
PC-3
U251
A549/ATCC
K-562
MOLT-4
HL-60 (TB)
RPMI 8226
NCI60 Cell Lines

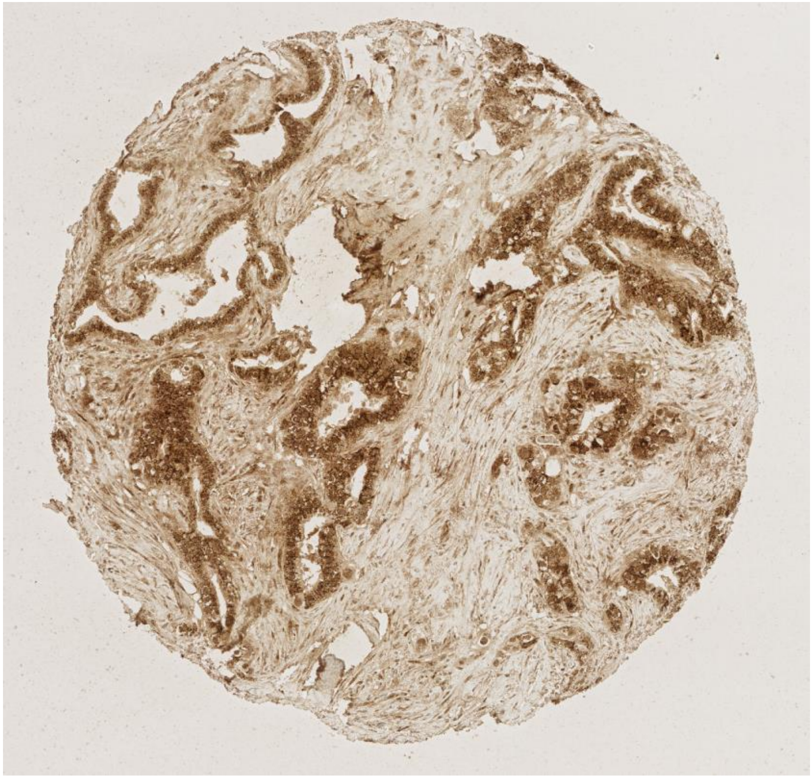

37. CPTC-TP53BP1-1

Technique	Analyte	Result
WB	RecProt	P
Wes	HeLa	NT
	MCF10A	NT
	LCL57	NT
WB	HeLa	U
	MCF10A	U
	LCL57	U
IF	HeLa	P
	MCF10A	P
	LCL57	P
SCWB	HeLa	NT
	MCF10A	NT
	LCL57	NT
IP*	RecProt	NT
IP*	HeLa	W
	MCF10A	N
	LCL57	P
IHC		P
NCI60		P

P – Positive, N – Negative, U – Undetermined, W – Weak, NT – Not Tested, * Please refer to tables for results

WB against commercial recombinant protein

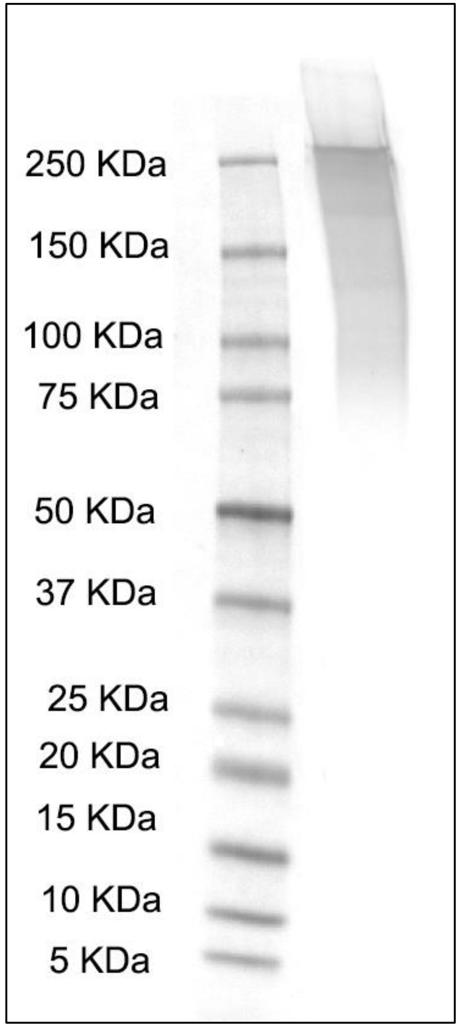

Ag Vendor: Origene

Cat #: TP318016

Description: Recombinant protein of human tumor protein p53 binding protein 1 (TP53BP1), transcript variant 3

Predicted MW: 213.4 kDa

Endogenous WB

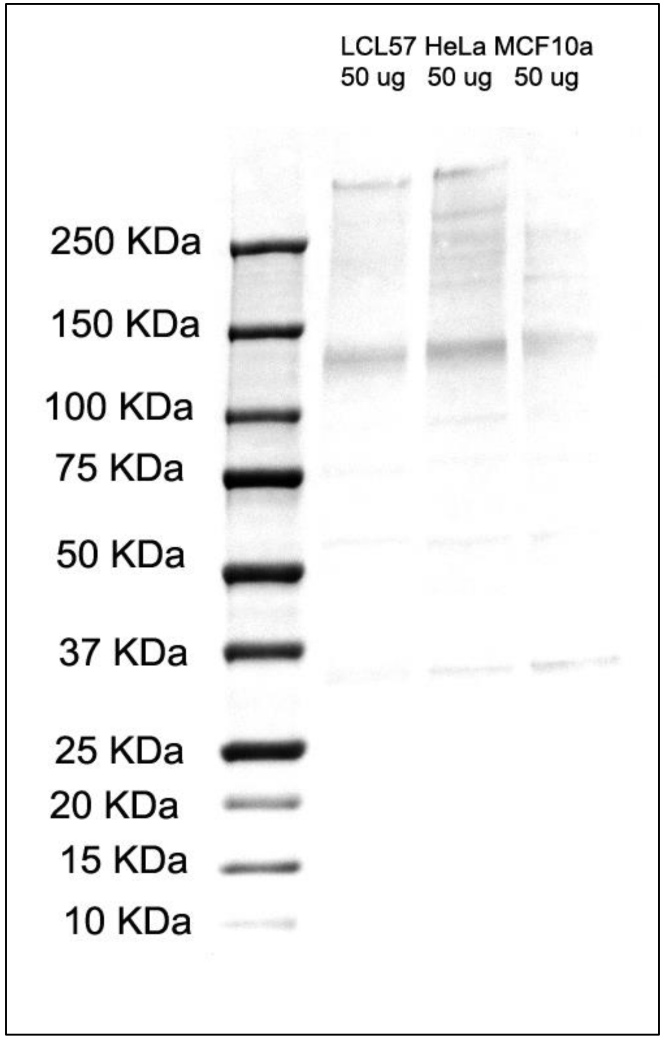

Immunofluorescence – HeLa

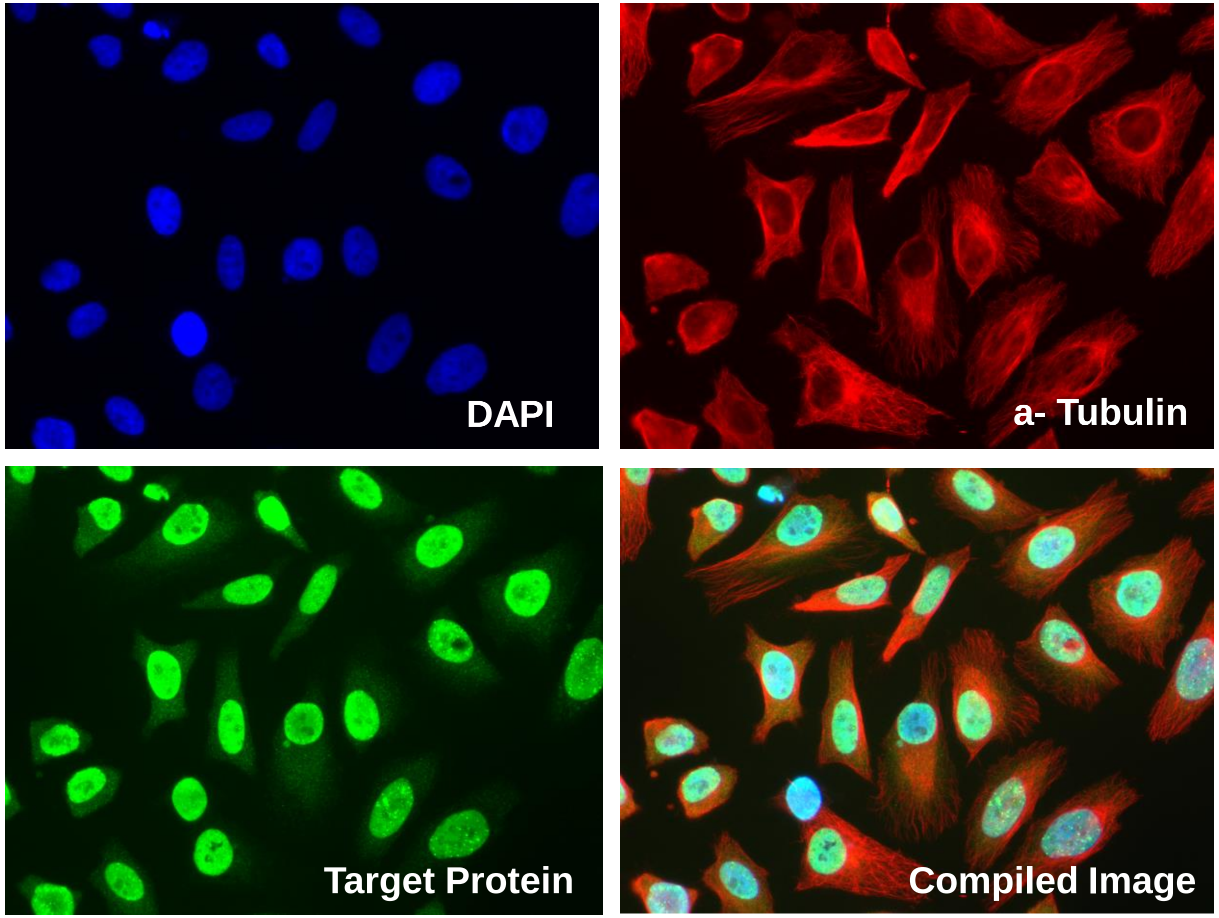

Subcellular location: Nucleus, Other
Primary Antibody concentration – 0.5 ug/mL

Immunofluorescence – MCF10A

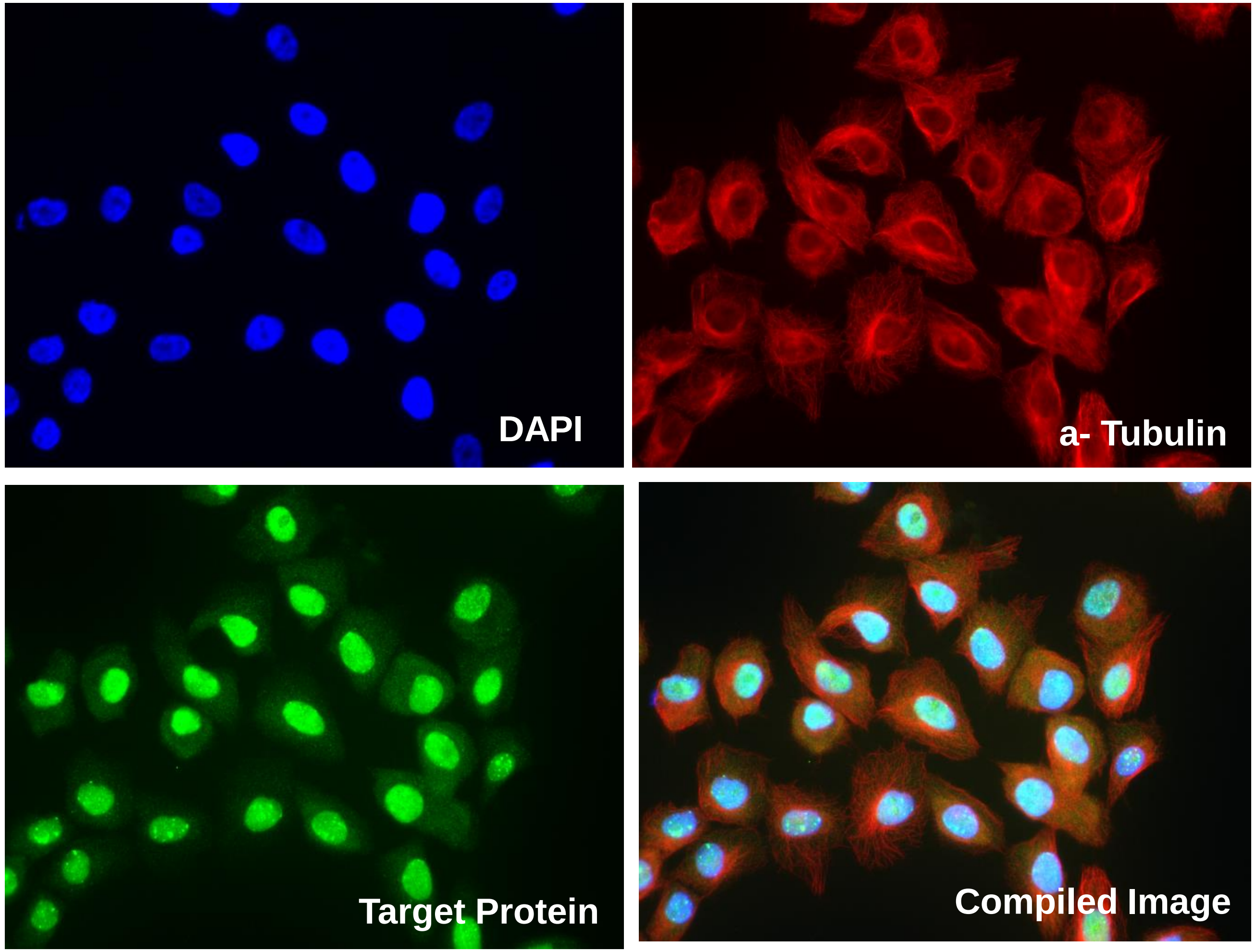

Subcellular location: Nucleus, Other
Primary Antibody concentration – 0.5 ug/mL

Immunofluorescence – LCL57

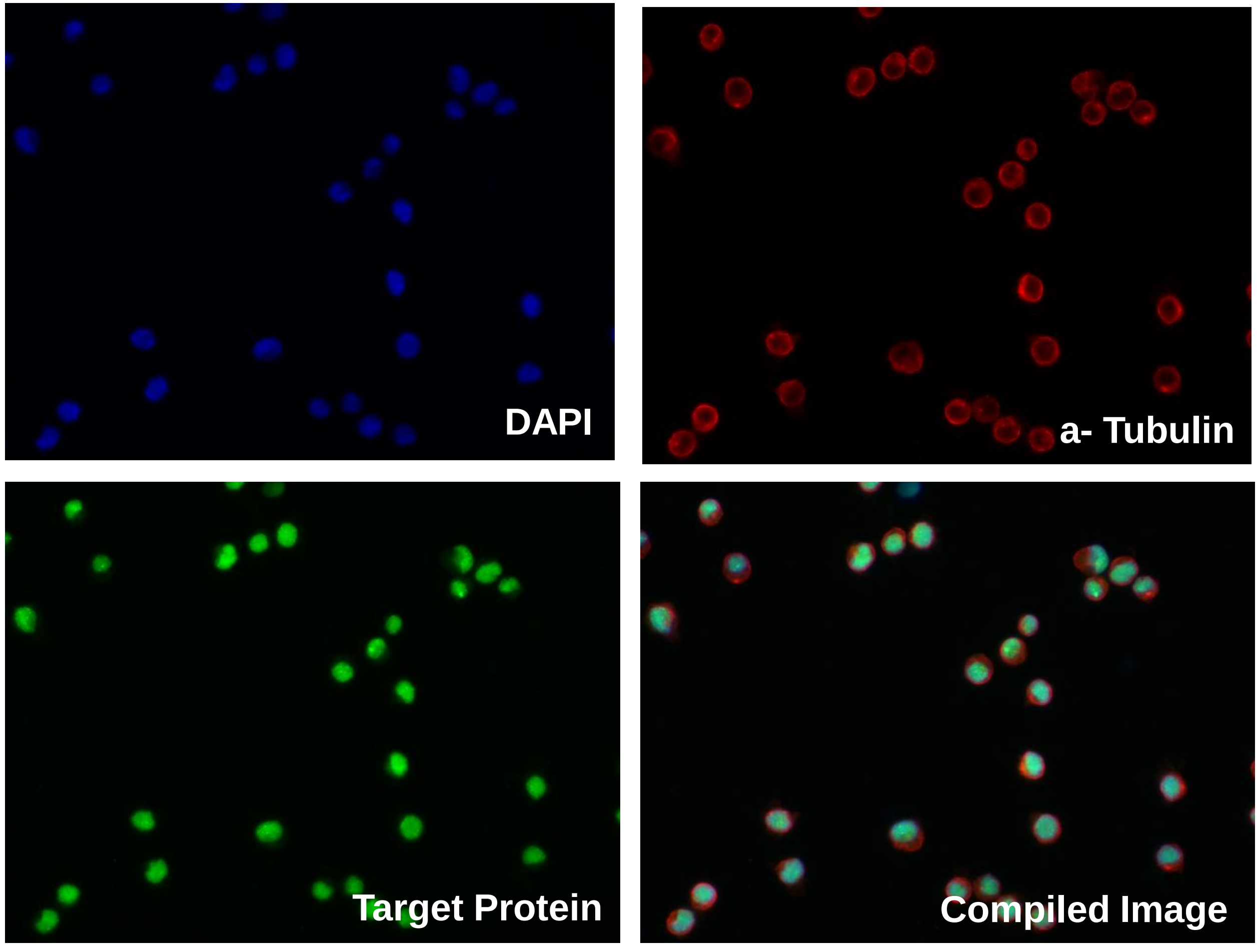

Subcellular location: Nucleus, Other

CPTC-TP53BP-1

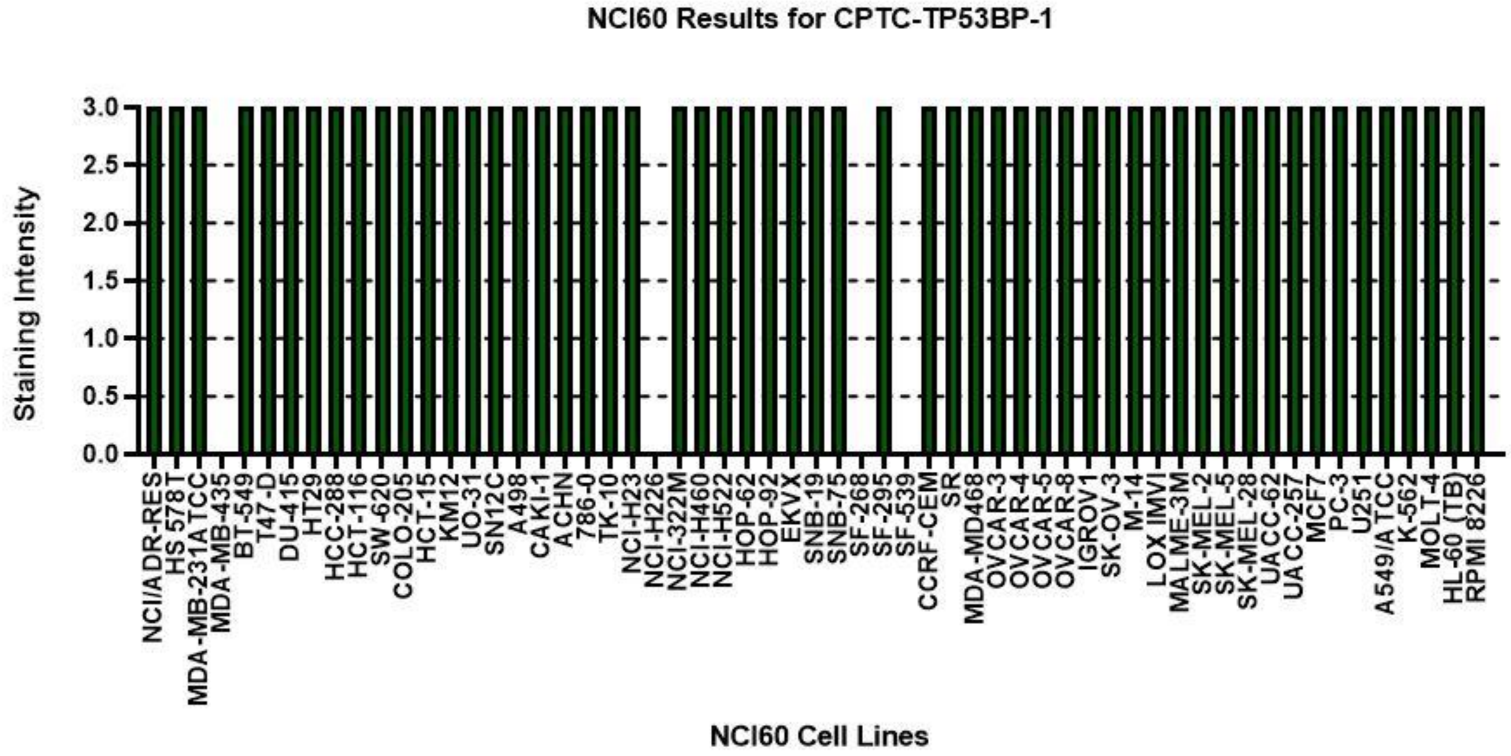

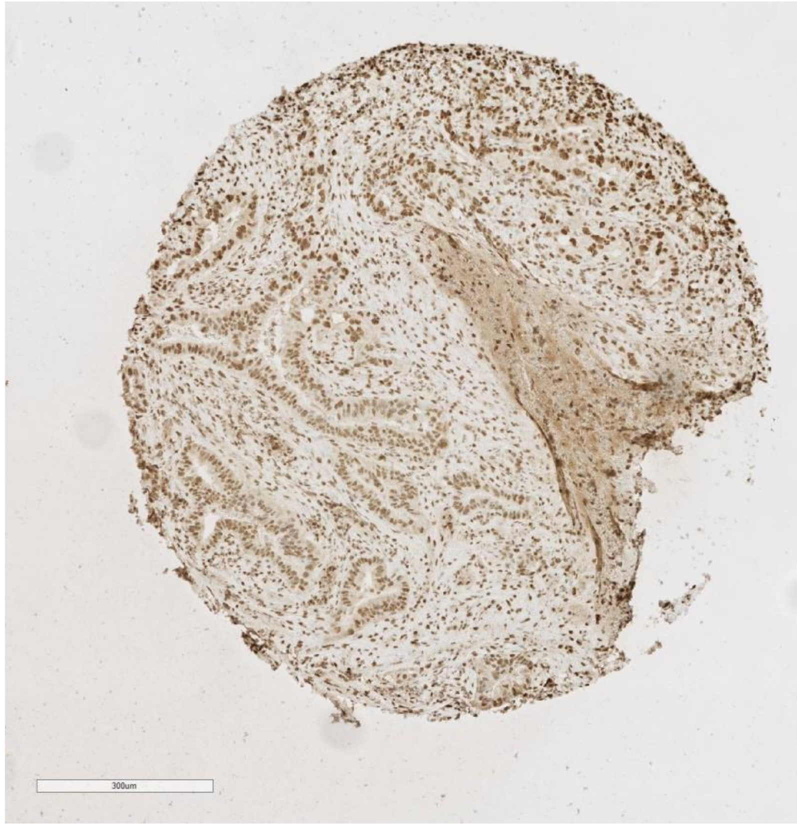

38. CPTC-TUBB-1

Technique	Analyte	Result
WB	RecProt	**N***
Wes	HeLa	**NT**
	MCF10A	**NT**
	LCL57	**NT**
WB	HeLa	**NT**
	MCF10A	**NT**
	LCL57	**NT**
IF	HeLa	**NT**
	MCF10A	**NT**
	LCL57	**NT**
SCWB	HeLa	**NT**
	MCF10A	**NT**
	LCL57	**NT**
IP	RecProt	**NT**
IP	HeLa	**NT**
	MCF10A	**NT**
	LCL57	**NT**
IHC		**N***
NCI60		**NT**

N – Negative, NT – Not tested, * Data not shown

39. CPTC-UTP14A-1

Technique	Analyte	Result
WB	RecProt	N*
West†	HeLa	N
	MCF10A	U
	LCL57	N
WB§	HeLa	N
	MCF10A	N
	LCL57	N
IF	HeLa	NT
	MCF10A	NT
	LCL57	NT
SCWB	HeLa	NT
	MCF10A	NT
	LCL57	NT
IP	RecProt	NT
IP	HeLa	NT
	MCF10A	NT
	LCL57	NT
IHC		P
NCI60		P

U – Uncertain, N – Negative, NT – Not Tested, * Data not shown
† Data shown are from Phospho-specificity Experiment. § Data from Phospho-specificity Experiment are not shown.

Endogenous Wes

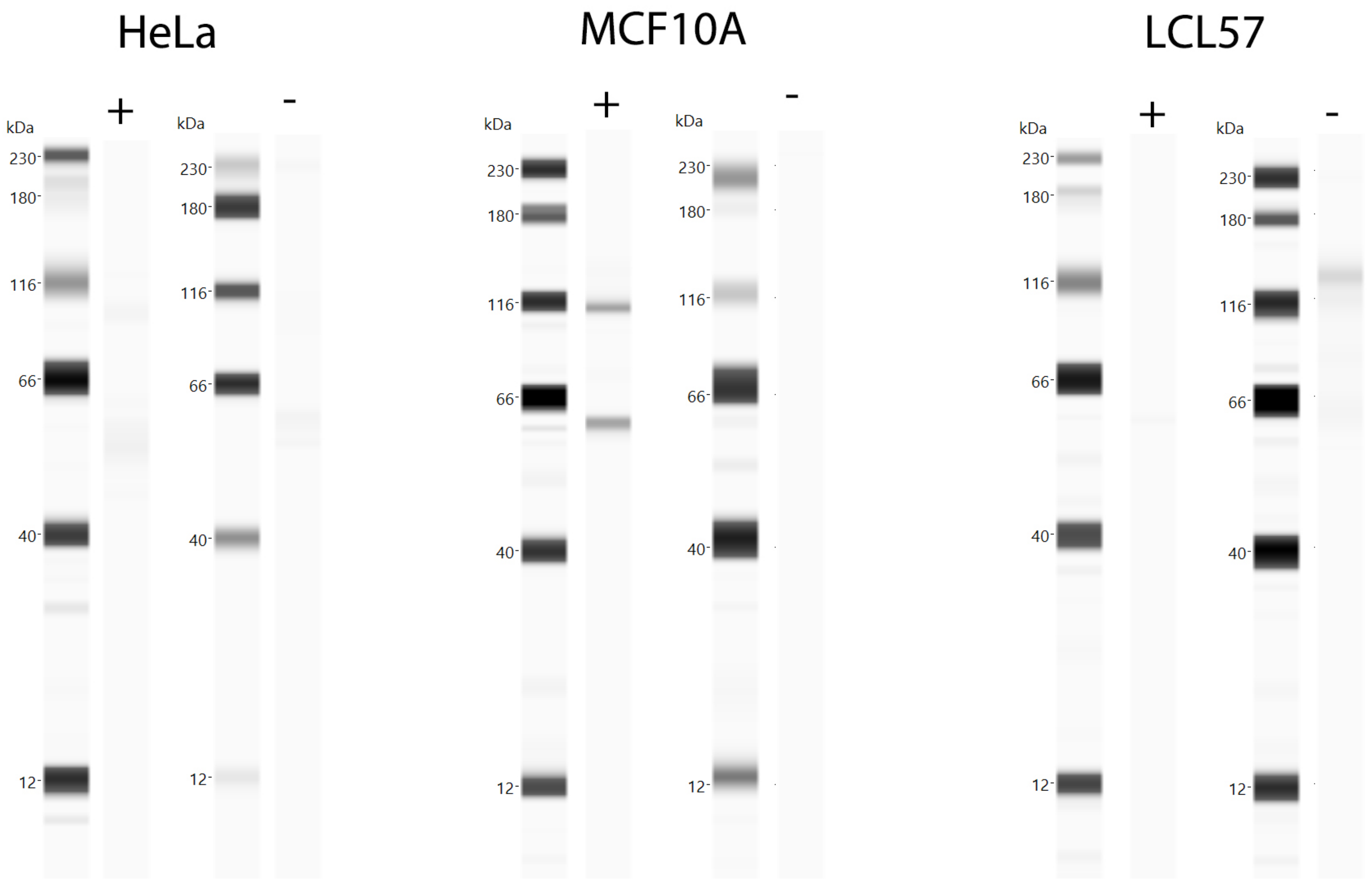

Expected molecular weight: 88, 69, 82 kDa

+ : irradiation treated cells
- : not treated cells

CPTC-UTP14A-1

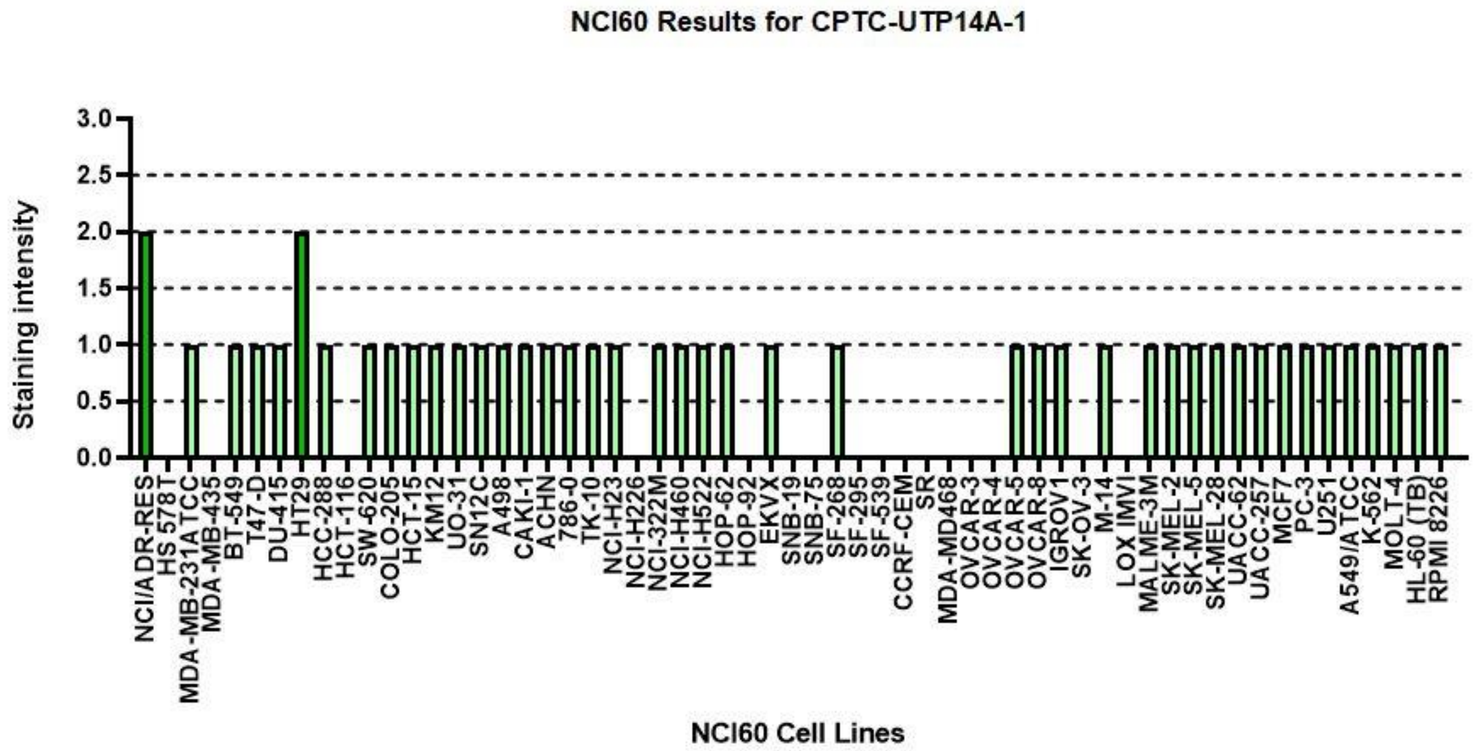

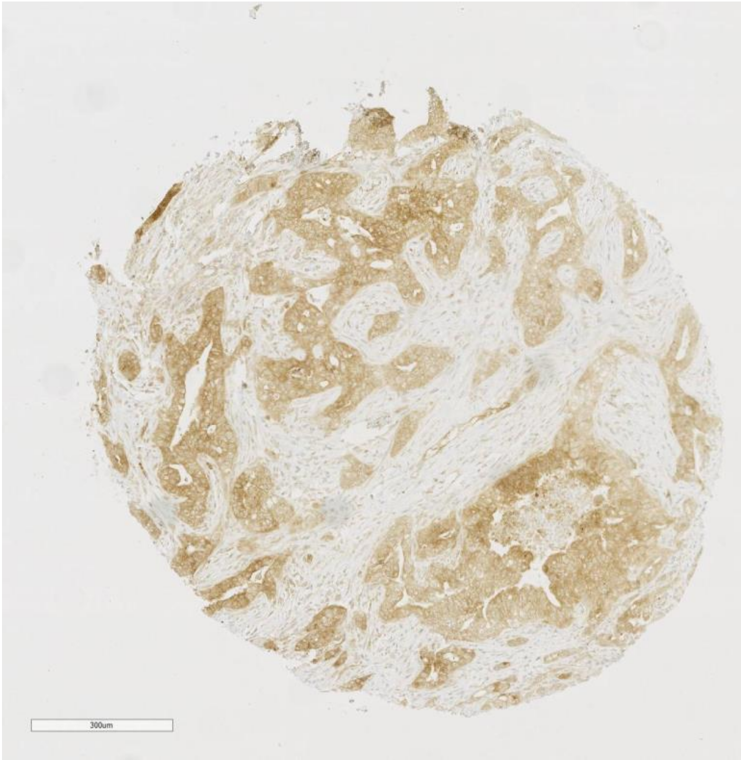

E. Summary

A panel of 39 rabbit anti-human antibodies were characterized in the Antibody Characterization Laboratory (ACL) at the Frederick National Laboratory for Cancer Research facility in Frederick, Md. A summary of the test results for the primary screen against recombinant protein is seen in Figure 3. The number of individual applications is seen in Figure 4. Figure 5 shows the global performance across all tested applications. One of the 21 clones negative against recombinant protein was shown to have phospho-specificity.

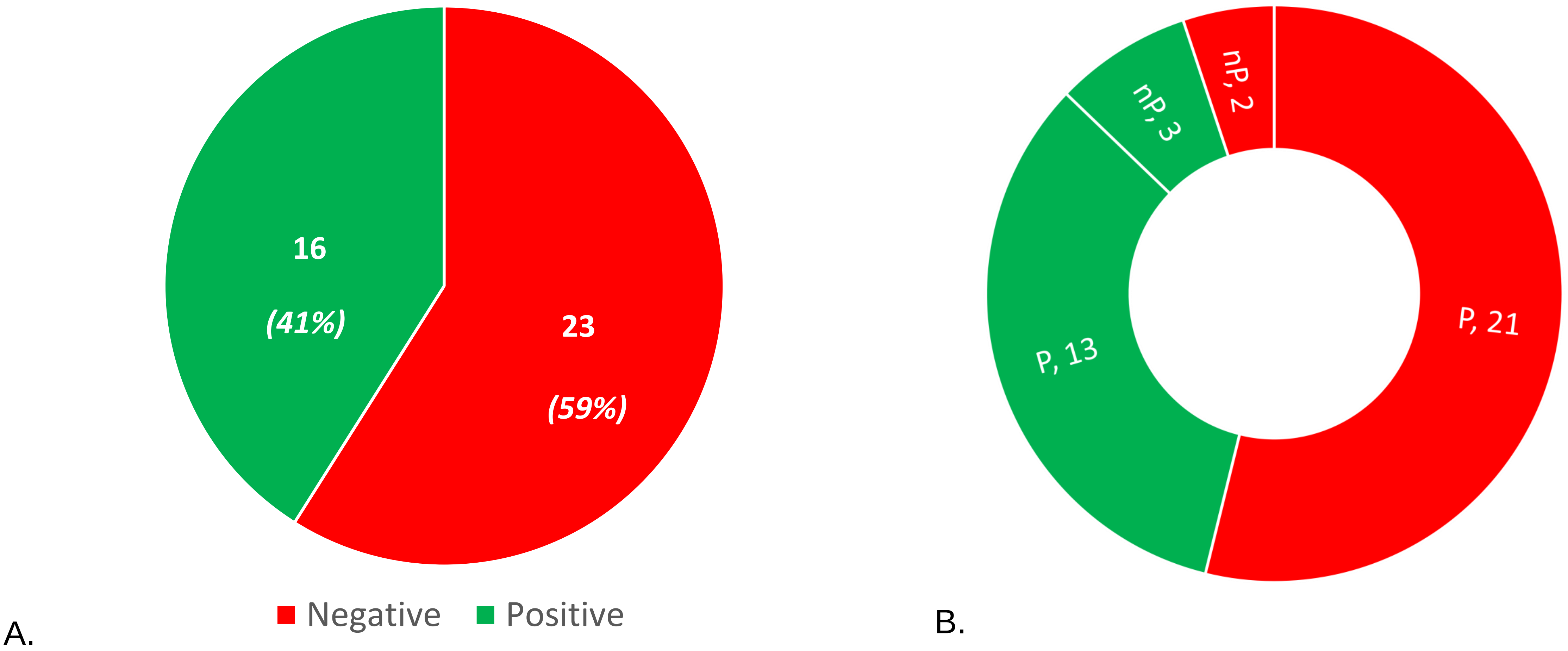

Figure 3. Western Blot against Recombinant Protein. Test results for the 39 antibodies that were characterized (A). Test results for antibodies based upon phosphorylation status of target (5 non-phospho-targets (nP) and 34 phospho-targets (P)) (B).

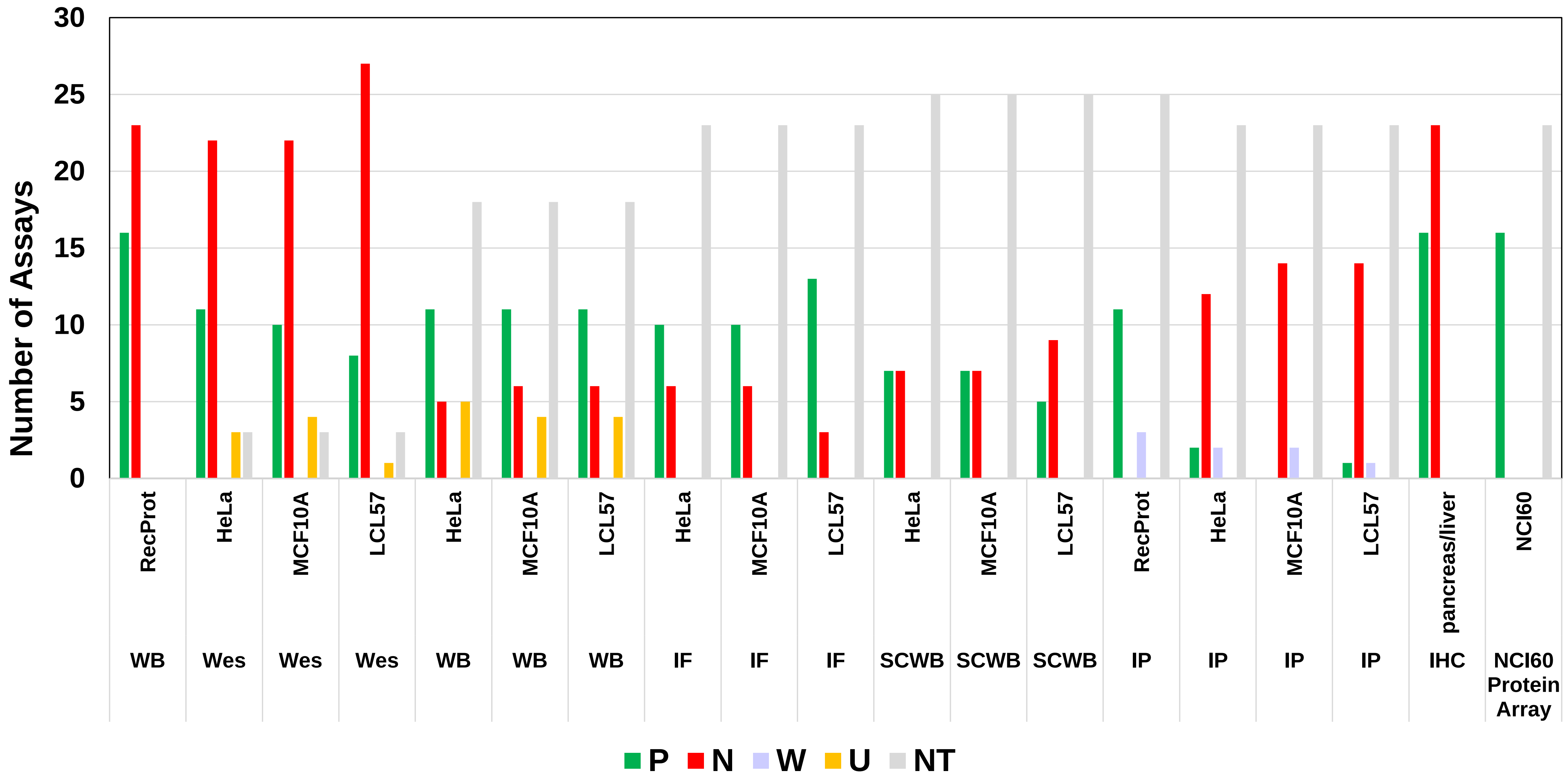

Figure 4. Number of individual applications.

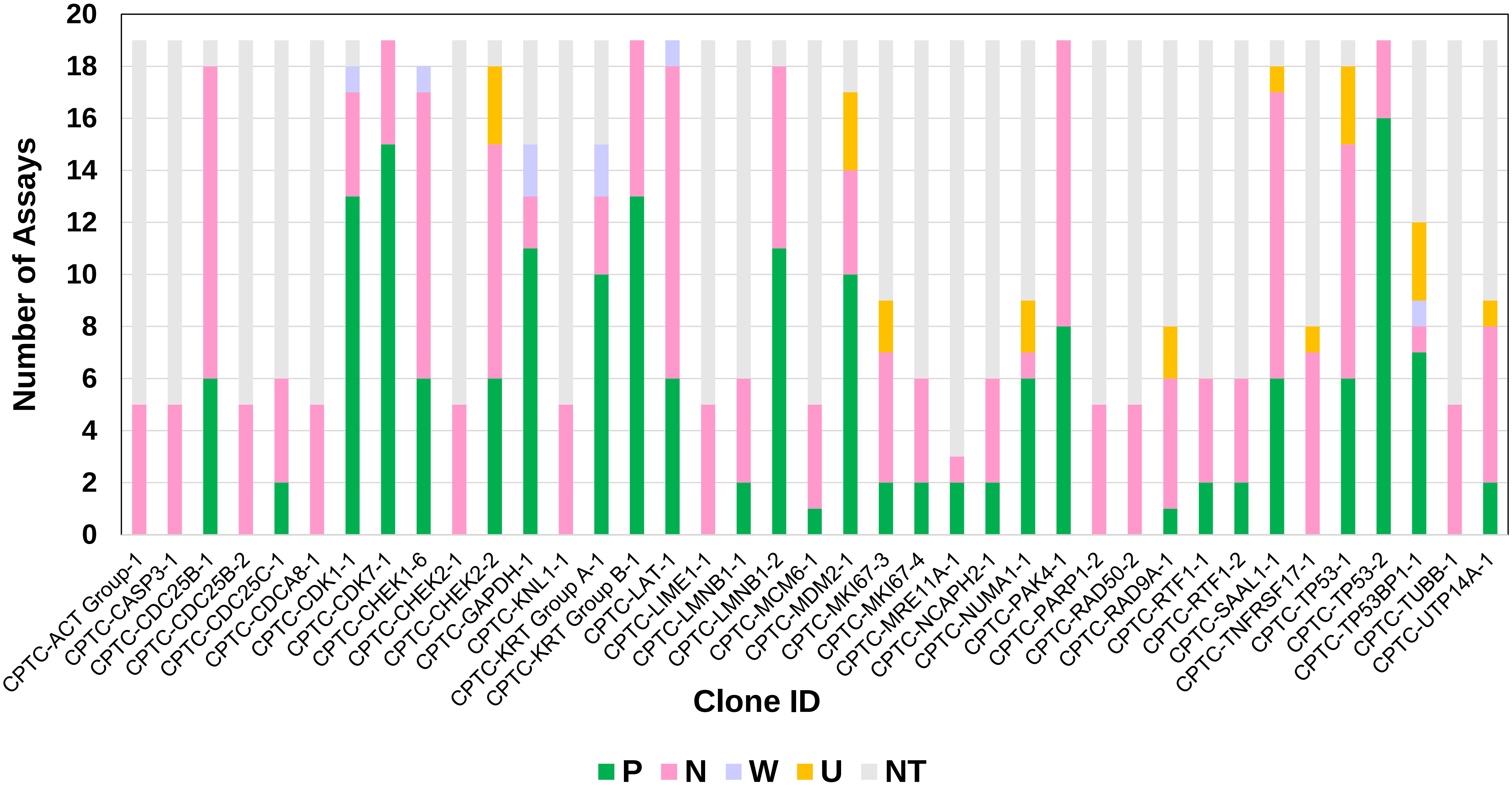

Figure 5. Global performance across all tested assays.

F. References

Whiteaker, J.R., Zhao, L., Saul, R., et al. A Multiplexed Mass Spectrometry-Based Assay for Robust Quantification of Phosphosignaling in Response to DNA Damage. *Radiation Research*. 2018;189(5):505-518.
